# Dual Inhibitors of p38α Mitogen-Activated Protein Kinase and Butyrylcholinesterase for the Modulation of Neuroinflammation and Cognitive Dysfunction

**DOI:** 10.1021/acs.jmedchem.6c01461

**Published:** 2026-08-10

**Authors:** Damijan Knez, David Malinak, Anže Meden, Svit Ferjančič Benetik, Anja Pišlar, Zuzana Kohoutova, Iveta Vondrackova, Miroslav Psotka, Antonin Lycka, Lenka Pulkrabkova, Ondrej Soukup, Adam Skarka, Rudolf Andrys, Xavier Brazzolotto, Alexandre Igert, José Dias, Florian Nachon, Jan Detka, Filip Targosiński, Dziyana Hliabovich, Joseph S. Brunzelle, Ludmilla Shuvalova, Kinga Sałat, Saktimayee Mitra Roy, Daniel Martin Watterson, Kamil Musílek, Stanislav Gobec

**Affiliations:** † 37663 University of Ljubljana, Faculty of Pharmacy, 1000 Ljubljana, Slovenia; ‡ University Hospital in Hradec Kralove, Biomedical Research Centre, 500 05 Hradec Kralove, Czech Republic; § University of Hradec Kralove, Faculty of Science, Department of Chemistry, 500 03 Hradec Kralove, Czech Republic; ∥ Département de Toxicologie et Risques Chimiques, Institut de Recherche Biomédicale des Armées, Brétigny-sur-Orge 91220, France; ⊥ Department of Pharmacodynamics, Chair of Pharmacodynamics, Faculty of Pharmacy, 49573 Jagiellonian University Medical College, Krakow 30-688, Poland; # Department of Pharmacology, 3270 Northwestern University, Chicago, Illinois 60611, United States

## Abstract

Chronic neuroinflammation and cholinergic dysfunction are major contributors to cognitive decline in Alzheimer’s disease. Here, we report a series of pyridazine-based multitarget-directed ligands that simultaneously inhibit human butyrylcholinesterase (hBChE) and p38α mitogen-activated protein kinase (p38α MAPK). Pyridazines **8** and **21** were identified as potent dual inhibitors with submicromolar inhibitory potencies against target enzymes and high selectivity over acetylcholinesterase. X-ray crystallographic analyses revealed the experimental binding modes of **8** and **21** in hBChE and p38α MAPK, and identified conserved π–π, π–cation, and hinge region interactions that rationalize dual-target engagement. Both compounds were blood-brain barrier permeable, exhibited low cytotoxicity, and significantly attenuated lipopolysaccharide-induced proinflammatory responses and apoptosis in BV2 microglia. *In vivo*, compound **21** enhanced cognitive performance in mouse models of scopolamine-induced amnesia and LPS-stimulated neuroinflammatory cognitive impairment. Collectively, these results document the potential of structure-assisted design to achieve selective dual-target pharmacology within a single molecular entity.

## Graphical abstract

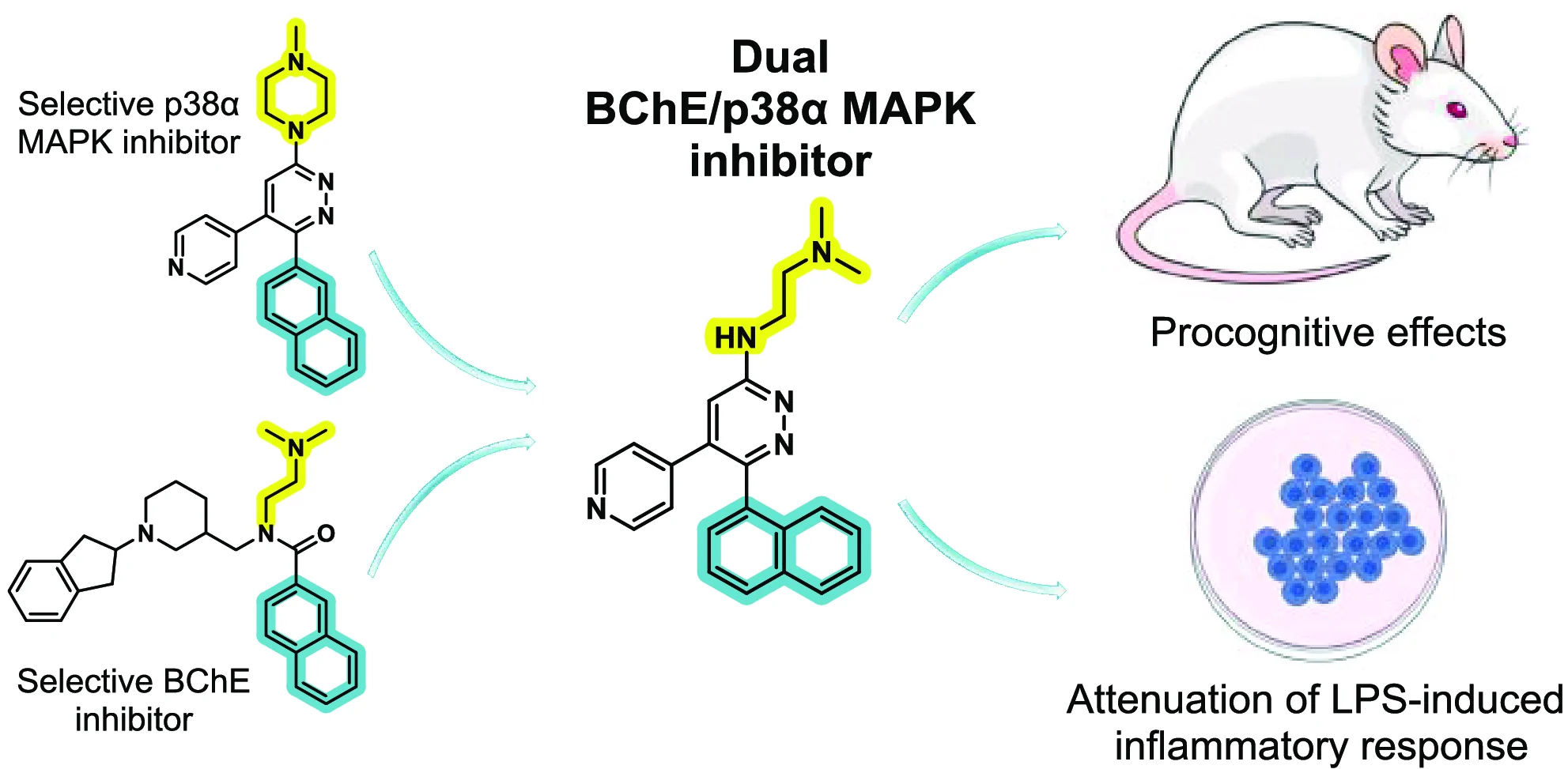

## Introduction

Neurodegenerative disorders now represent the leading cause of dementia worldwide. Mounting evidence places the neuroinflammation-synaptic dysfunction pathophysiology process as a common feature of their pathogenesis, offering a compelling rationale for therapeutic intervention.^1^ Neuroinflammation, driven predominantly by microglial and astrocytic activation is increasingly recognized as a convergent mechanism underlying neuronal damage in diverse neurodegenerative disorders. While the glial response is beneficial in the early stages by clearing the cellular debris and protein aggregates, its sustained activation creates a self-perpetuating inflammatory environment that amplifies oxidative stress, cytokine production, and protease release, ultimately exacerbating neuronal injury.^2,3^

A plethora of signaling cascades are involved in neuroinflammation of AD. In particular, p38α mitogen-activated protein kinase (MAPK) signaling has been implicated in the cellular response to stress at several levels. Activation of p38α MAPK is observed in neurons, microglia, and astrocytes within the AD brain.^4–7^ In microglia and astrocytes, activation of p38α MAPK leads to the production of pro-inflammatory mediators and cytokines, such as IL-1β, IL-6, TNF-α, COX-2, and iNOS. In the neuron, p38α MAPK modulates synaptic plasticity and genetic deletion or pharmacological inhibition protects against synaptic and memory dysfunction.^8,9^ Pharmacological inhibition by selective p38MAPK inhibitors in clinical development, such as neflamapimod (VX-745) and **MW150** (Figure 1), paves the way for novel disease-modifying therapeutic strategies targeting the neuroinflammatory pathways in AD, Lewy body dementia, Parkinson’s disease, autism spectrum disorder and beyond.^10–14^

**Figure 1:**
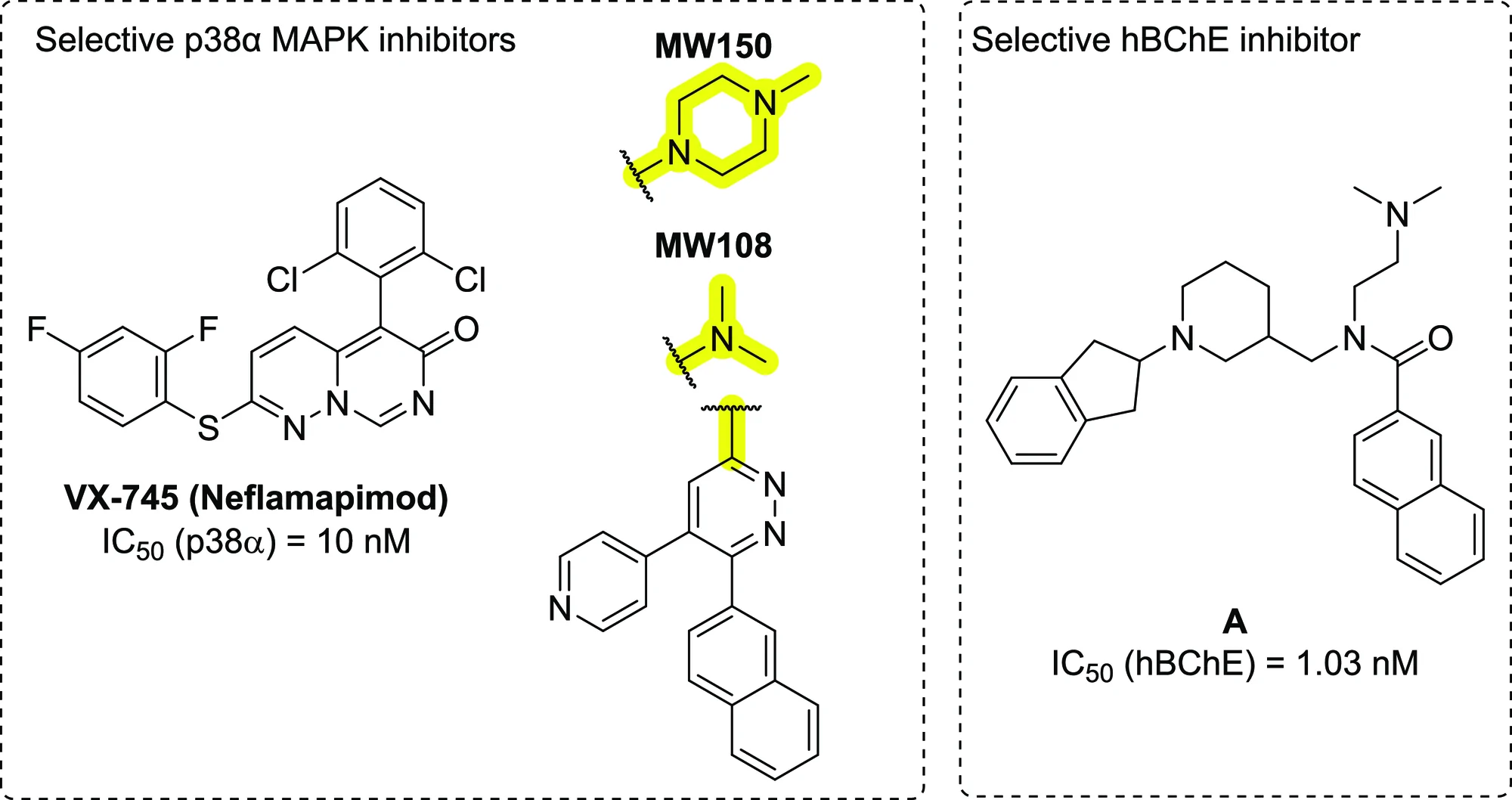
Selective p38α MAPK inhibitors VX-745 (neflamapimod, PDB: 3FC1), and **MW150 (**PDB: 4R3C**)**,^16^ and selective human (h)­BChE inhibitor **A**.^18^

**MW150** is a third-generation, orally bioavailable, brain penetrant, isoform-specific, high-affinity (Michaelis–Menten *K*_*i*_ = 54 nM) p38α MAPK inhibitor that is clinically safe and well-tolerated. **MW150** was generated by targeted chemical diversification of **MW081a**, an inactive fragment that engages a conserved cross-kinase architectural feature. Site- specific diversification with an aryl substituent allowed engagement of selective p38α MAPK space to generate *in vivo* active p38α MAPK inhibitor. Pharmacoinformatics-driven optimization identified the 2-naphthyl substituent as a therapeutic candidate *versus* phenyl or 1-naphthyl analogues. **MW150** explicitly avoids the off-target and hepatotoxicity risks of earlier pan-kinase and second generation p38MAPK inhibitors.^15,16^ **VX-745**, rebranded as neflamapimod, is a second-generation inhibitor of p38α MAPK and p38β MAPK with limited off-target liabilities, developed by classical medicinal chemistry optimization of hits from small molecule library activity screens, and clinically safe.^17^ The molecular recognition of human p38α MAPK by **MW150** and **VX-745** are distinct and differ in discrete pharmacological properties. These two selective p38 MAPK inhibitor therapeutic candidates provide the foundation for pursuit of new generation p38α MAPK and p38β MAPK inhibitors, including the pursuit of dual target inhibitors that retain the key p38 MAPK recognition features but add additional target activities with potential clinical impact.

Cholinergic signaling is indispensable for higher-order cognition, including attention, learning, and memory, whereas degeneration of the cholinergic system is a consistent feature of AD progression. The loss of basal cholinergic neurons projecting into the hippocampus and cortex results in reduced acetylcholine (ACh) levels and impaired synaptic transmission, directly contributing to cognitive decline.^19–21^ Cholinesterase inhibitors, namely donepezil, galantamine, and rivastigmine, improve cholinergic neurotransmission by preventing ACh breakdown, producing modest but clinically significant symptomatic benefits in mild to moderate AD.^22,23^ While the activity of acetylcholinesterase (AChE) remains unchanged or drops by 45% in some regions of the AD brain, the activity of its structural homologue, the butyrylcholinesterase (BChE), increases up to 90%.^24–26^ Notably, BChE colocalizes with amyloid plaques, and its inhibition reduces Aβ levels in transgenic AD rodent models.^27^ Therefore, BChE could also serve as a biomarker for incipient AD.^28–30^ The absence of BChE in peripheral cholinergic neurons, *i*.*e*., in the motor end plate and peripheral nervous center, suggests that selective BChE inhibition should spare peripheral cholinergic neurotransmission and is unlikely to provoke the parasympathetic adverse effects typical of AChE inhibitors.^31^ Selective BChE inhibition enhances memory and learning in AD models without the peripheral side effects of typical AChE inhibitors.^32–34^

AD pathogenesis arises from intersecting processes—amyloid deposition, tau hyperphosphorylation, synaptic failure, and chronic neuroinflammation. This raised the hypothesis that multitarget-directed ligands (MTDLs), also referred to as multifunctional ligands, could safely engage two or more AD-associated pathological changes. MTDLs offer the potential of therapeutic breadth with reduced drug-drug interaction risk of drug combinations.^35,36^ MTDL design couples two or more pharmacophores by merging them into one scaffold, fusing them contiguously, or tethering them with a linker.^37^ As proposed in our previous publication,^38^ designed MTDLs with dual BChE and p38α MAPK could potentially alleviate symptoms of AD through BChE inhibition and address the underlying neuroinflammatory processes that would ultimately lead to disease-modifying effects. Building on a ligand-centric comparison of the experimentally determined binding poses of selective hBChE inhibitor **A** ^18^ (Figures 1 and 2) and the p38α MAPK inhibitor **MW150**,^38^ we designed dual, pyridazine-based hBChE/p38α MAPK inhibitors. Evaluation of the pyridazine series, supported by structural biology, and subsequent biological characterization of dual inhibitors led to the identification of a hBChE/p38α MAPK inhibitor **21**, which demonstrated anti-inflammatory and precognitive effects in cellular and *in vivo* models of AD.

**Figure 2:**
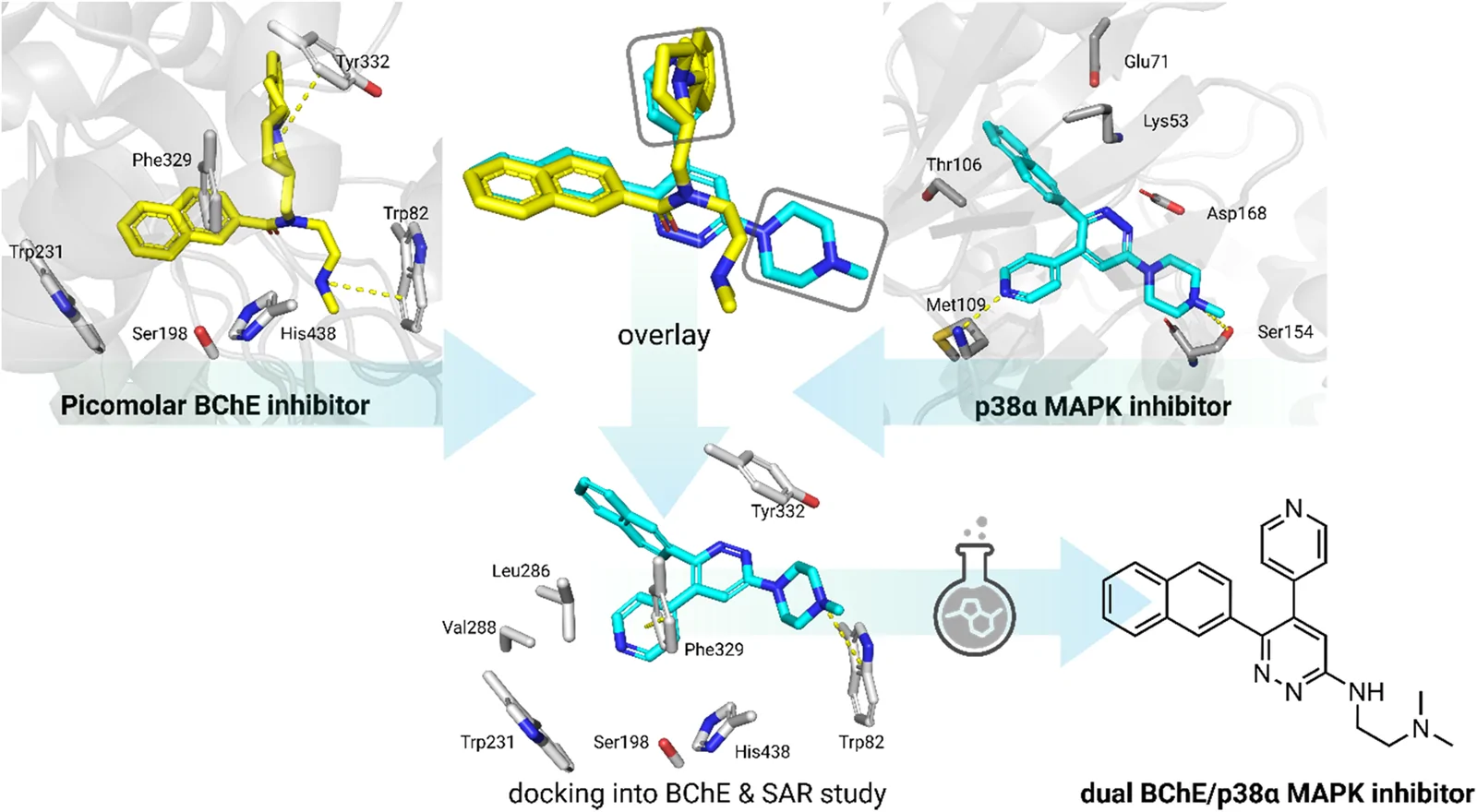
Rational design of dual BChE/p38α MAPK inhibitors. Previously reported crystal structures of inhibitor A bound to hBChE (top left, in yellow; PDB: 5NN0)^18^ and **MW150** bound to p38α MAPK (top right, in cyan; PDB: 4R3C)^16^ were used for a ligand-centric superposition (top middle). The bottom panel shows the prospective docking model of MW150 in hBChE (PDB 6QAA) generated in the present study. The comparison concerns the spatial arrangement of ligand features and does not imply structural similarity between the two protein active sites.

## Results and Discussion

### Design and Synthesis of Dual hBChE/p38α MAPK Inhibitors

The design of the dual inhibitors was based on a ligand-centric comparison of two previously reported crystal structures: **MW150** bound to human p38α MAPK (PDB 4R3C)^16^ and inhibitor **A** bound to hBChE (PDB 5NN0).^18^ It should be emphasized that hBChE and p38α MAPK are structurally and evolutionarily unrelated enzymes with fundamentally different folds, catalytic mechanisms, and active-site architectures. Accordingly, our design strategy did not assume similarity between the protein active sites themselves. Instead, the rationale was based on a ligand-centric comparison of experimentally determined inhibitor binding modes as explained in detail in our recent publication.^38^ Superposition of the experimentally determined ligand conformations revealed common spatial arrangements of several molecular features, despite the fundamentally different structures of the two target proteins (Figure 2). No experimental crystal structure of **MW150** bound to hBChE is available. Therefore, to investigate whether **MW150** could be accommodated within the hBChE active-site gorge, we prospectively docked **MW150** into hBChE using Schrödinger Glide and the hBChE crystal structure (PDB 6QAA).^39^ Upon docking of **MW150** into hBChE using Schrödinger Glide software (Figure 2, bottom), the naphthalene of **MW150** points toward the entrance of the hBChE’s active site, forming π–π interactions with Tyr332, whereas the pyridine protrudes toward the voluminous acyl-binding pocket of hBChE delineated by Trp231, Phe398, Leu286, and Val288. The *N*,*N*-dimethylamine forms a π–cation interaction with Trp82 of the choline-binding pocket. This simple *in silico* exercise shows that the *N*-methylpiperazine of **MW150** could be neatly replaced with various amines. Experimental data in support of this hypothesis are evident in various PDB deposits of selective p38α MAPK inhibitors of the MW-series in the complex with the kinase.^15,16^ This part of the inhibitor is solvent-exposed and does not form any crucial interactions with the p38α MAPK ATP-binding site.^38^ Based on our previous work, interaction with Trp82 is pivotal for potent inhibition of hBChE.^18^ Accordingly, *N*-methylpiperazine of **MW150** was replaced with the more flexible and less sterically demanding *N*,*N*-dimethylethylamine side chain. This modification retains a protonatable tertiary amine capable of forming a cation−π interaction with Trp82 while allowing improved accommodation within the hBChE active-site gorge.

Reference selective p38α MAPK inhibitor **MW150** ^16^ and its derivatives were synthesized according to the procedure outlined in Scheme 1. Commercially available naphthoyl chlorides were reacted with *N*,*O*-dimethylhydroxylamine hydrochloride in the presence of triethylamine to obtain the corresponding electrophilic Weinreb-Nahm amides. The latter were further subjected to nucleophilic addition of (pyridin-4-ylmethyl)­lithium, which was prepared *in situ* by deprotonating the weakly acidic 4-picoline methyl group with lithium diisopropylamide, to furnish corresponding ketones **2a**–**b**. Alpha deprotonation of ketones with sodium hydride and subsequent alkylation of enolates with ethyl bromoacetate yielded γ-ketoesters **3a**–**b** that were cyclized into dihydropyridazines in the presence of hydrazine. Dihydropyridazine **4a** was facilely oxidized to aromatic pyridazine-2-one using bromine in acetic acid, whereas **4b** was transformed into the corresponding pyridazine-2-one using oxidative dehydrogenation in the presence of palladium on carbon as catalyst.^40^ Both pyridazine-2-ones were further transformed into corresponding 2-chloropyridazines **5a**–**b** using phosphorus­(V) oxychloride. Nucleophilic aromatic substitution with an excess of various amines, hydrazines, or hydroxylamines yielded **MW150** and 3-substituted pyridazines. Suzuki-Miyaura palladium-catalyzed cross-coupling^41^ of 2-chloropyridazine **5a** and potassium vinyltrifluoroborate led to 6-vinylpyridazine **19**,^42^ and subsequent catalyst-free hydroamination of vinyl heteroarene^43^ yielded 6-alkylated pyridazine **20**.

**Scheme 1:**
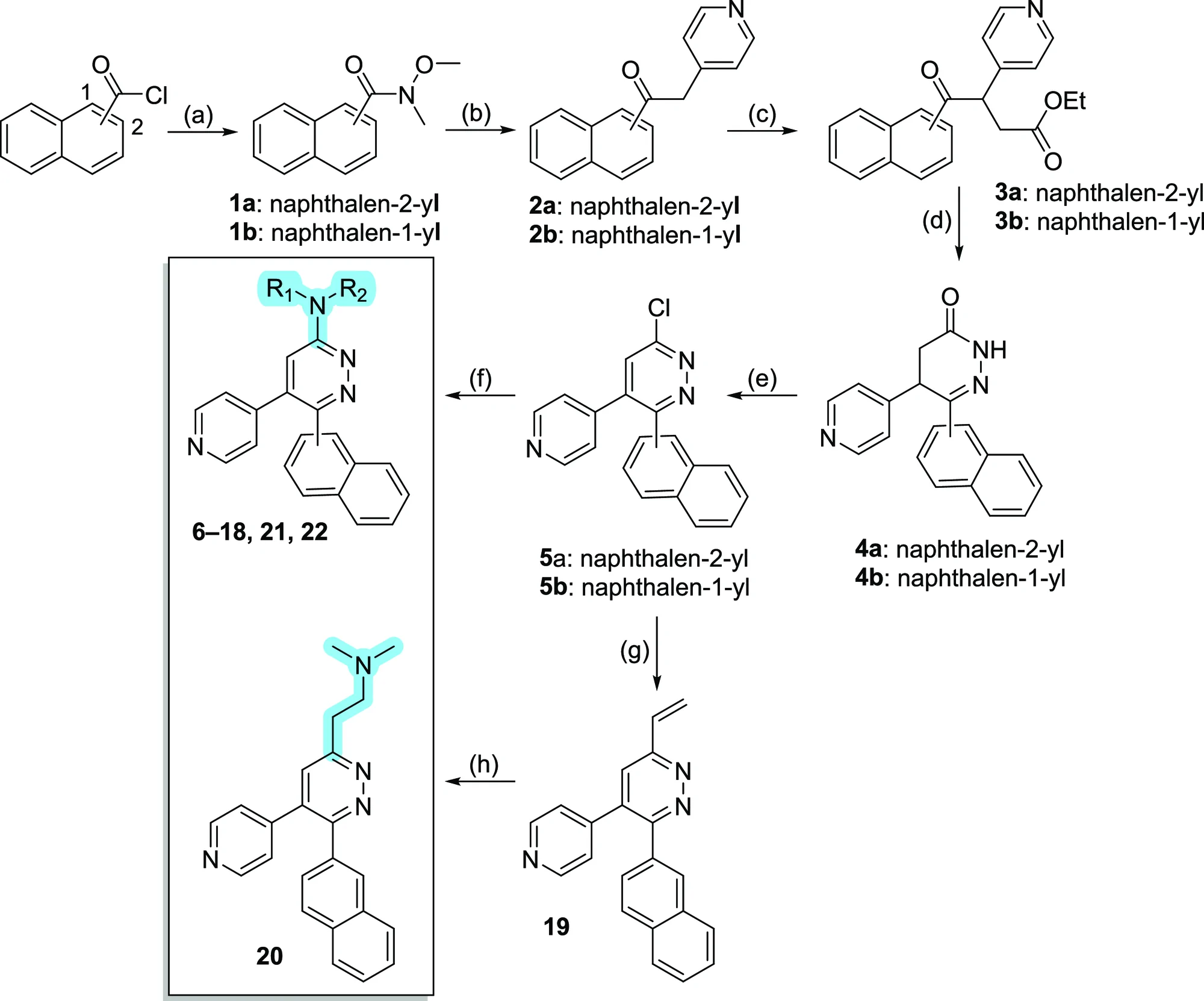
General Synthetic Routes to Pyridazines **6–22** ^a^

a Reagents and conditions: (a) *N*,*O*-dimethylhydroxylamine hydrochloride, triethylamine, THF, 0 °C to rt; (b) i. 4-picoline, lithium diisopropylamide, THF, −70 °C, 30 min, ii. corresponding Weinreb-Nahm amide **1a**–**b**, 0 °C; (c) ethyl bromoacetate, DMF, sodium hydride, 0 °C to rt; (d) hydrazine, EtOH, 95 °C; (e) i. bromine, AcOH, 70 °C for **5ai** (or Pd/C, toluene, 110 °C for **5bi**), ii. phosphorus­(V) oxychloride, MeCN, 85 °C; (f) corresponding amine, hydrazine or hydroxylamine, EtOH, 95 °C; (g) potassium vinyltrifluoroborate, triphenylphosphine, palladium­(II) chloride, caesium carbonate, THF/H_2_O, 80 °C; (h) dimethylamine, THF, 75 °C.

The synthesis of 1-naphthyl derivatives **21** and **22** was burdened by low yields due to copious side products, despite several optimization attempts (see Supporting Information for details); thus an alternative synthetic route were used to provide sufficient amounts of **21** needed for the *in vivo* experiments (*vide infra*) (Scheme 2).^44^ Briefly, a nucleophilic acyl substitution reaction and subsequent intramolecular cyclization of starting bromomaleic anhydride with hydrazine led to 4-bromo-1,2-dihydropyridazine-3,6-dione **23**. Suzuki–Miyaura cross-coupling^41^ using Pd­(PPh_3_)_4_ as the catalyst, and chlorination with phosphorus­(V) oxychloride was used to obtain dichloropyridazine **24**, which was transformed into **25** by nucleophilic aromatic substitution with *N*,*N*-dimethylethylenediamine. Final compound **21** was obtained by Suzuki-Miyaura cross-coupling of **25** with 1-naphthaleneboronic acid. Derivatives **6** (**MW150**) and **8**, and selective hBChE inhibitor **A** (Figure 1) used for *in vivo* studies were prepared according to the pathway outlined in Scheme 1 or according to our previously reported procedure,^18^ and used in the form of their tosylate salts for **6** (**MW150**) and **8**, or mono- for **21**, and dihydrochloride salts for **A**, respectively.^18^

**Scheme 2:**
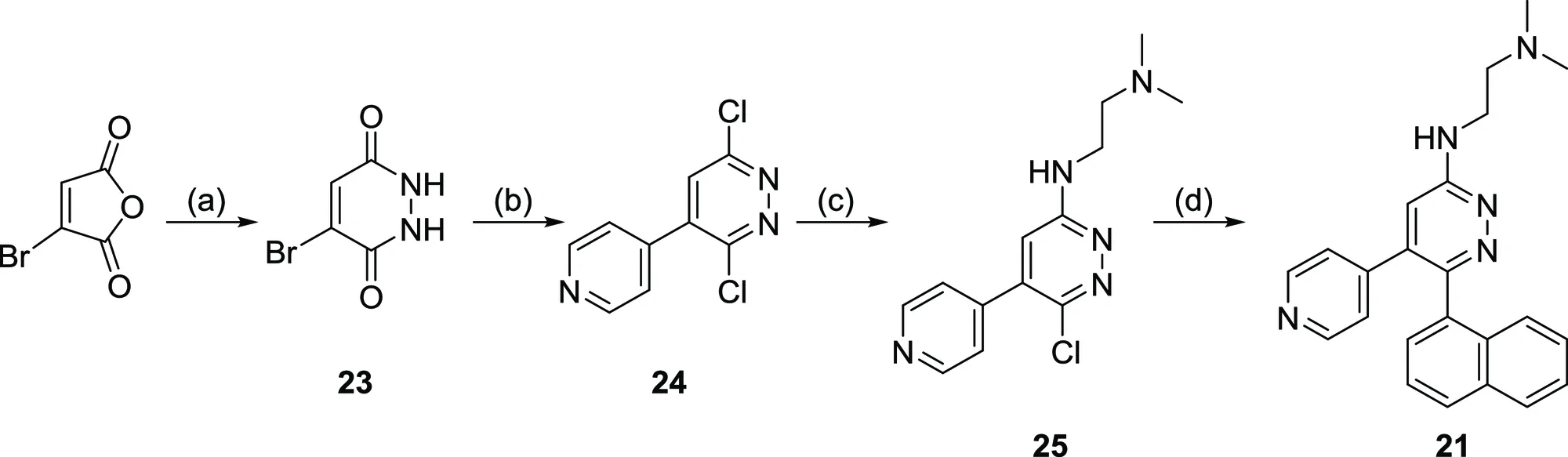
Optimized Synthetic Route to Pyridazine Analogue **21** ^a^

a Reagents and conditions: (a) hydrazine sulphate, H_2_O, 100 °C; (b) i. 4-pyridineboronic acid pinacol ester, Pd­(PPh_3_)_4_, sodium carbonate, DME/H_2_O (10:1), 110 °C, ii. phosphorus­(V) oxychloride, MeCN, 90 °C; (c) *N*,*N*-dimethylethylenediamine, EtOH, 90 °C; (d) 1-naphthaleneboronic acid, Pd­(PPh_3_)_4_, sodium carbonate, DME/H_2_O (10:1), 110 °C.

### Structure–Activity Relationships and Crystal Structures of Pyridazine-Based Dual hBChE/p38α MAPK Inhibitors

Pyridazines **6–22** were assayed *in vitro* against recombinant enzymes—human (h)­AChE, hBChE, and human p38α MAPK (Table 1) as described previously.^45^ Compounds retaining >50% residual enzyme activity (RA) at 100 μM were classified as inactive. IC_50_ values against p38α MAPK were determined for compounds inhibiting hBChE with IC_50_ values below 10 μM, as well as for the reference p38α MAPK inhibitors **MW150** (**6**) and **MW108** (**7**), which were included for comparison. All p38α MAPK inhibitory values reported in Table 1 represent IC_50_ values determined under a single, internally consistent assay format and are therefore intended for within-study comparison only; they should not be directly compared across different assays.^46^

**Table 1:**
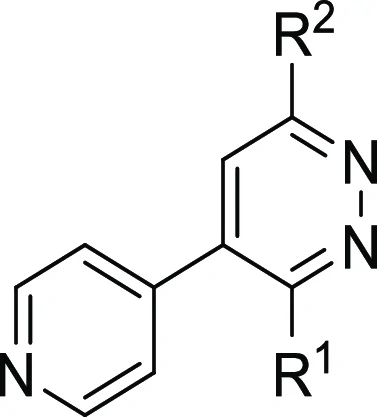

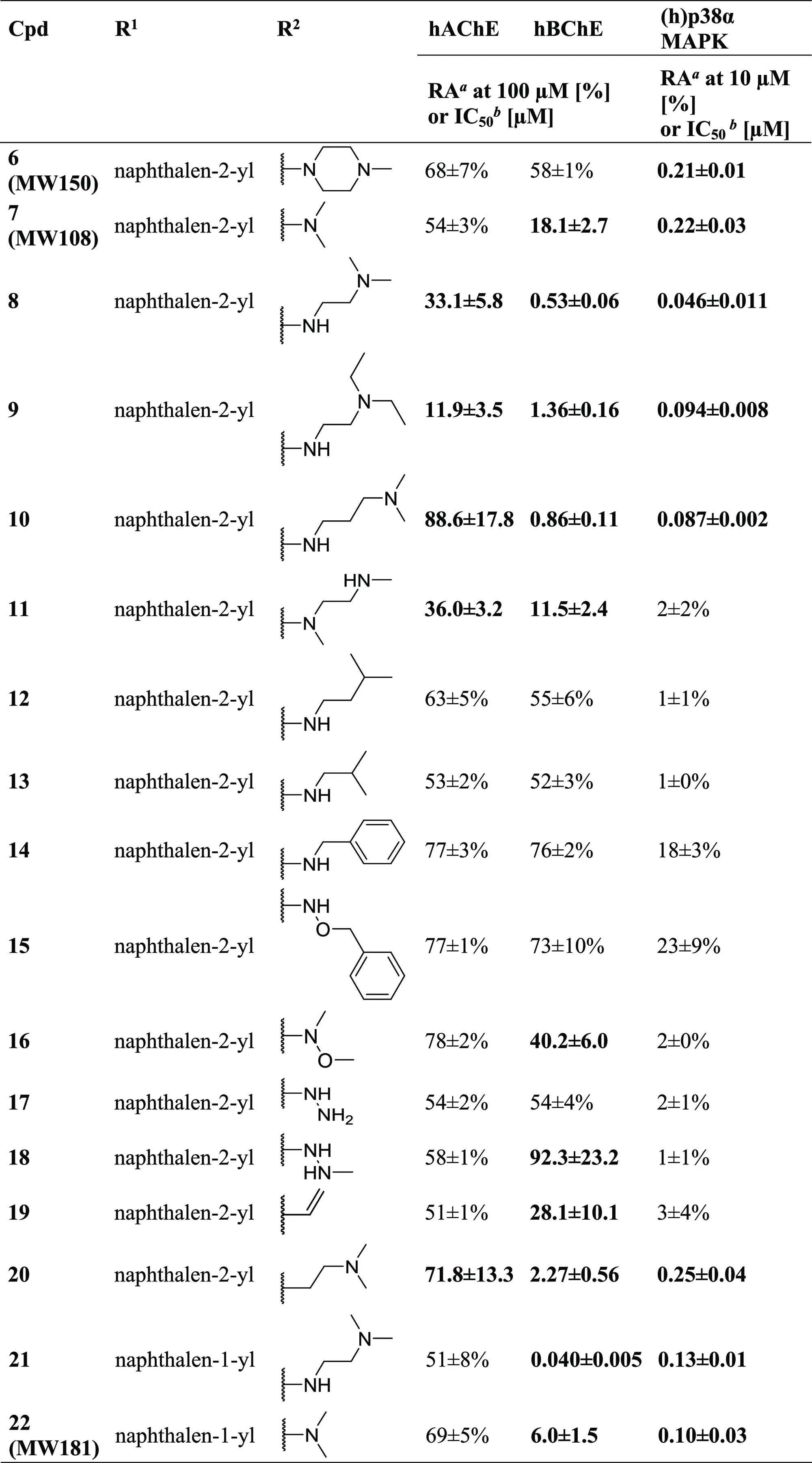
*In Vitro* ChE and p38α MAPK Inhibitory Potencies of Pyridazines **6–22**

a RA—residual activity (mean ± standard deviation of one independent experiment performed in triplicate for ChEs and in duplicate for p38α MAPK).

b SEM—standard error of the mean, data are the average of two to three independent experiments.

Our initial assumption that **MW150**, a selective p38α MAPK inhibitor, could interact with the active site of hBChE (Figure 2) was disproven by an *in vitro* assay—compound **6** (**MW150**) did not inhibit hBChE (Table 1). In contrast, replacing the *N*-methylpiperazine moiety of **MW150** with smaller and more flexible aliphatic amines, while retaining the key structural features of selective p38α MAPK inhibitors—the pyridine and naphthalene substituents on the pyridazine core^15,16^ —led to the anticipated hBChE inhibition. The *N* ^1^,*N* ^1^ *-*dimethylethane-1,2-diamine **8** and *N* ^1^,*N* ^1^ *-*dimethylpropane-1,3-diamine **10** analogues inhibited hBChE in the submicromolar range, showing at least 60-fold selectivity over hAChE. Increasing the size of the pyridazine substituent R_2_ (*e*.*g*., *N*,*N*-diethylamine **9** *vs* *N*,*N*-dimethylamine **8**), removing the distal amine from the side chain (*e*.*g*., isoamylamine **12**), or introducing an aromatic group (benzylamine **14**) diminished hBChE inhibition. Simple hydroxylamines or hydrazines (**15**–**18**) were not tolerated in terms of hBChE inhibition. In contrast, direct C-C attachment of the side chain to the pyridazine core (*N*,*N-*dimethylethane **20**) preserved hBChE activity in the low-micromolar range. Compound **10** inhibited hBChE and p38α MAPK with IC_50_ values of 0.86 and 0.087 μM, respectively, corresponding to an approximately 10-fold difference. It also showed approximately 100-fold selectivity for hBChE over hAChE, exceeding the approximately 62-fold selectivity of compound **8**. Compound **20** similarly displayed dual activity, although it was less potent against both intended targets and showed approximately 30-fold hBChE/hAChE selectivity. Interestingly, under the assay conditions used here, p38α MAPK inhibition was broadly comparable across the derivatives regardless of the nature of the R_2_ substituent, consistent with prior reports that this region is solvent-exposed.^15,16,44^ Among the naphthalen-2-yl derivatives, compound **8** displayed the most promising dual-target profile, inhibiting p38α MAPK and hBChE with IC_50_ values of 0.046 and 0.53 μM, respectively. Thus, although both activities were submicromolar, compound **8** was approximately 10-fold more potent against p38α MAPK and cannot be considered fully balanced. This observation highlights a central challenge in optimization of MTDLs: achieving a balanced activity on both targets.^47,48^ Since alternative aryl substituents, including naphthalen-1-yl and phenyl groups, are tolerated in p38α MAPK inhibitors of the MW series,^44,49^ the closely matched naphthalen-1-yl analogue **21**, which retained the dimethylethanediamine side chain of **8**, was prepared. The matched pair **8**/**21** enabled a direct assessment of the effect of naphthyl orientation on dual-target activity and binding. Introduction of the naphthalen-1-yl in **21** improved the balance of activity, affording IC_50_ values of 0.040 μM against hBChE and 0.13 μM against p38α MAPK, corresponding to an approximately 3-fold difference in the opposite direction. Direct comparison of these ratios should nevertheless be interpreted cautiously because the IC_50_ values were determined using different assay formats and do not directly represent relative target occupancy under cellular or *in vivo* conditions. For MTDLs, the pharmacologically optimal activity ratio may also depend on target abundance, accessibility, and the degree of inhibition required to elicit the desired biological response. Notably, compound **21** did not inhibit hAChE at 100 μM (Table 1), supporting the strategy to limit off-target cholinergic interactions that could contribute to adverse effects.^50^ Compound **22** (**MW181**) was previously reported to inhibit p38α MAPK with a *K*_*i*_ of 0.184 μM, compared with an IC_50_ of 0.10 ± 0.03 μM determined here. Although the values were obtained using different assay formats and are not directly interchangeable, both measurements place compound **22** within the same submicromolar potency range.

To fully characterize the activity discrepancy for hBChE inhibition between **8** and **21**, an X-ray crystallography study was undertaken by soaking the ligands into crystals of recombinant hBChE. This allowed the determination of the structures of the complexes at 2.62 and 2.84 Å resolution for **8** and **21**, respectively. The electron densities observed in the active site gorge of **8**- and **21**-soaked hBChE allowed for the unambiguous fitting of the ligands, which are flipped in comparison to those predicted by molecular modeling for **MW150** (Figure 3). This serves as a reminder that molecular modeling, while invaluable for generating hypotheses about binding modes, may not always accurately capture the proper orientation, highlighting the necessity of experimental validation through structural studies.^51^ The ligands arrange quite similarly into hBChE, with the naphthalene rings pointing toward Trp231 in the acyl pocket, the pyridine rings engage π–stacking with Tyr332 that forms the BChE peripheral aromatic site together with Asp70, while the dimethylethanediamine moiety resides in the choline pocket, interacting with Trp82. More specifically, the distal naphthalene ring lies at 5.0 and 5.1 Å from the pyrrole ring of Trp231 for **8** and **21**, respectively. In contrast, the proximal naphthalene rings exhibit a weaker π–π interaction with Phe329, characterized by respective distances of 5.8 and 6.5 Å for **8** and **21**. The same Phe329 interacts with the pyridazine core of the inhibitor, with distances of 5.7 Å for **8** and 5.6 Å for **21**. Stronger interaction between the pyridine ring and Tyr332 translates into distances of 3.9 Å and 4.4 Å for **8** and **21**. Finally, the terminal dimethylamine engages the benzyl ring of Trp82 with distances of 4.1 and 4.3 Å for **8** and **21**, respectively. Additionally, the two structures are distinguished by the presence of water molecules near the active site, close to the oxyanion hole formed by the amide nitrogen atoms of Gly116, Gly117, and Ala199, together with the O^γ^ atom of catalytic Ser198. For **21**, only one water molecule could be modeled between the protein and the ligand, while for **8**, two water molecules were modeled (Figure S2). All these minute differences add up to improved inhibition of hBChE by the naphthalen-1-yl analogue **21**.

**Figure 3:**
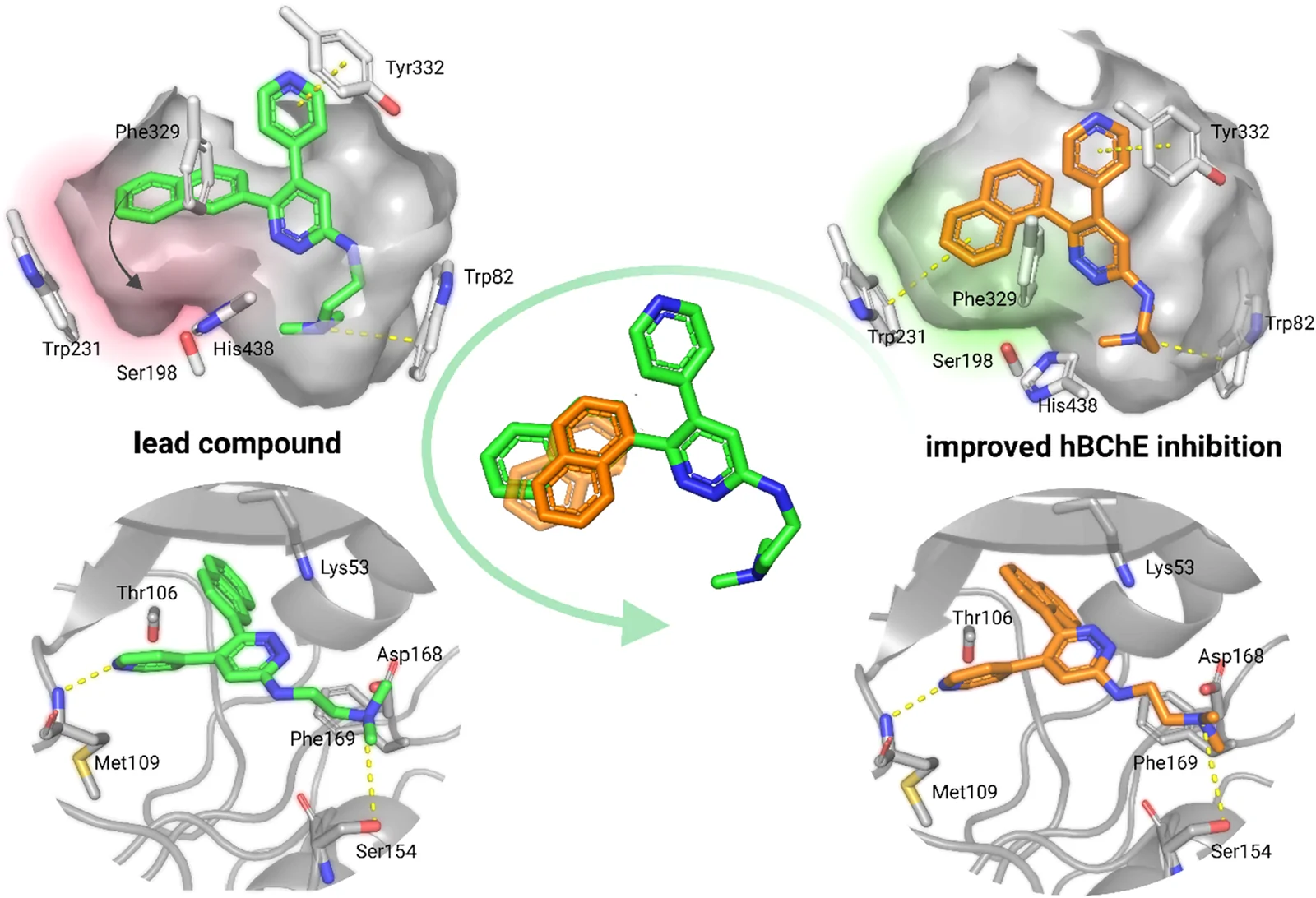
Crystal structures of dual hBChE/p38α MAPK inhibitors **8** (left, carbon atoms in green) and **21** (right, carbon atoms in orange) in the hBChE active site (top, PDB 9GCQ and 9GCR, respectively), and in the ATP-binding pocket of human p38α MAPK (bottom, PDB 9D8G and 9D6P, respectively). Ligands and notable residues are represented as sticks; the gray semitransparent surface and ribbons represent the active site gorge of the enzymes. Yellow dashed lines represent the π-stacking interactions and H-bonds.

The binding of compounds **8** and **21** to p38α MAPK was determined and compared with the binding characteristics of p38α MAPK inhibitors previously reported by the Watterson group (**MW181**, PDB 4F9Y; **MW108**, PDB 4F9W).^16,44^ The resulting crystal structures of complexes (PDB 9D8G and 9D6P for **8** and **21**, respectively) allow a residue-level comparison of the naphthyl orientation (2-naphthyl in **8** *vs* 1-naphthyl in **21**) within the hydrophobic pocket and the solvent-exposed amine region relative to **MW150** and **MW181**. Both **8** and **21** bind in the canonical ATP site of p38α MAPK and preserve the hallmark **MW**-series binding topology: the pyridine nitrogen engages the hinge at the Met109–Gly110 peptide bond (single hinge H-bond, 2.9 Å and 2.8 Å for **8** and **21**, respectively), and the activation loop remains in a DFG-in state without evidence of a hinge ‘glycine flip’ or other catalytic-core rearrangements (Figure 3). This binding mode is consistent with MW-series inhibitors, where a single hinge contact to Met109 is a defining interaction and the small gatekeeper Thr106 enables productive occupation of the adjacent hydrophobic pocket.^16,44^ A key differentiator between **8** and **21** is the orientation of the naphthyl substituent within the hydrophobic pocket: the 1-naphthyl *vs* 2-naphthyl substitution is known (from MW-series structures) to produce a measurable change in the aryl-vector and packing in this region, and the electron density in Watterson’s p38α MAPK structures directly captures this difference in substituent orientation. In our structures, the naphthyl group remains anchored against the same hydrophobic region (HR-I) delineated by Ala51, Lys53, Leu75, Ile84, Leu86, and Thr106, supporting the observation that the aryl–HR-I contact set is conserved across the series and helps explain why p38α MAPK potencies remain in the nanomolar range despite substantial variation at the solvent edge.^44^ The dimethylethanediamine moiety of **8** and **21** adopts a well-defined conformation extending toward the solvent-exposed region, replacing the dimethylamine (in **MW181**) or *N*-methylpiperazine (in **MW150**) groups. Notably, the solvent-edge substituent does not appear to induce compensatory rearrangements in the hinge region or HR-I pocket, consistent with the MW-series concept that potency and selectivity are dominated by the conserved hinge contact (Met109) and hydrophobic-pocket complementarity enabled by the Thr106 gatekeeper.^16,44^ Thus, compared with the reference p38α MAPK structures of **MW150** and **MW181**, compounds **8** and **21** retain the same ATP-site anchoring interactions while introducing a solvent-exposed diamine selected to improve hBChE binding without compromising p38α MAPK engagement. These crystallographically observed interactions provide a structural rationale for the submicromolar p38α MAPK potencies of compounds **8** and **21**. However, because crystal structures were determined only for these two compounds, the results do not establish that the same binding features account for differences in activity across the entire pyridazine series, particularly among the less active analogues.

### Kinase Selectivity and *In Vitro* Pharmacokinetics

Based on their *in vitro* activities, compounds **8** and **21** were analyzed for their kinase selectivity against p38α/β/γ/δ MAPKs as well as 17 other phylogenetically and structurally related kinases using the 33PanQuinase Activity Assay (Tables S4 and S5).^52,53^ Compounds **8** and **21** inhibited both p38α and p38β MAPK at the tested concentrations. At 1 μM, compound **21** produced residual activities of 22% and 47% for p38α and p38β MAPK, respectively, indicating greater inhibition of the α isoform under these assay conditions. However, these single-concentration measurements do not permit a quantitative assessment of isoform selectivity. Neither compound showed appreciable inhibition of p38γ or p38δ MAPK under the tested conditions (Table S4). Both compounds also inhibited the atypical MAPK-related kinase NLK.^16^ PKD3, RIPK2, and TNIK, which were reported as off-targets of an early MW-series hit,^15^ were not included in the present panel. Their inhibition by compound **21** therefore cannot be excluded, and broader kinase profiling will be required to determine both its selectivity and any potentially relevant polypharmacology. To predict the brain penetration of compounds **8** [experimentally determined log *D*_7.4_ = 1.57] and **21** [experimentally determined log *D*_7.4_ = 0.81], the BBB Parallel Artificial Membrane Permeability Assay (BBB-PAMPA) was used. High effective permeability coefficients (*P*_*e*_) were determined for **8** and **21** ([5.5 ± 0.9]×10^–6^ cm × s^–1^ and [4.3 ± 0.03] × 10^–6^ cm × s^–1^, respectively), exceeding the benchmark value for the BBB permeable drugs (Table S6).^54^ Phase I metabolic stability of compounds **8** and **21** using human liver microsomes revealed that the most prominent metabolites were the hydroxylated, *N*-monodemethylated, and *N*-dealkylated products (Table S7). Such metabolic profile is characteristic of naphthalene-substituted scaffolds, reflecting the ring’s susceptibility to epoxidation and subsequent hydroxylation by P450 enzymes, in parallel with oxidative demethylation of alkylamino substituents.^55,56^ The biological activities of the identified metabolites were not determined. Previous studies of the MW-series focused primarily on microsomal stability and CYP liability and did not report the target activities of individual metabolites. Since the identified metabolites (Table S7) retain the pyridine-pyridazine-naphthyl core structure, p38α MAPK activity cannot be excluded, particularly for the *N*-monodemethylated metabolite. Conversely, metabolites lacking the terminal basic amine may show reduced hBChE inhibition due to loss of the interaction with Trp82; however, direct testing is required to confirm their activity.

### *In Vitro* Cytotoxicity and Anti-Inflammatory Effects

Based on the promising *in vitro* inhibitory potencies and *in vitro* pharmacokinetics, dual hBChE/p38α MAPK inhibitors **8** and **21** were subjected to further cell-based assays. The results were compared side-by-side with selective p38α MAPK inhibitor **MW150**, which entered a phase II clinical trial in January 2022 (not recruiting, status unknown),^57^ and selective hBChE inhibitor **A** (Figure 1), both of which were used as the starting point for the development of dual hBChE/p38α MAPK inhibitors reported herein. Cytotoxicity studies on neuroblastoma SH-SY5Y, hepatocellular carcinoma HepG2 and microglial BV2 cell lines using the MTS assay showed that compounds **8** and **21** did not affect cell metabolic activity at concentrations up to 10 μM or 20 μM, depending on the cell line. For comparison, the selective hBChE inhibitor **A** altered metabolic activity at 10 μM (Figure S3). Cell viability was further assessed by uptake of 7-aminoactinomycin D (7-AAD), a fluorescent DNA-intercalating dye that penetrates only cells with compromised membrane integrity, thereby quantifying nonviable cells. Unlike the MTS assay, which estimates viability based on metabolic activity, the 7-AAD assay provides a more direct measure of membrane integrity and cell death.^58,59^ The results of the 7-AAD-based cytotoxicity assay confirmed low cytotoxicity of **MW150**, **8** and **21** on SH-SY5Y and BV2 cells, and a substantial cytotoxic effect of the selective hBChE inhibitor **A** on the BV2 cell line at concentrations of 5 μM or higher (Figure S4).

As dual hBChE/p38α MAPK inhibitors **8** and **21** exhibited negligible cytotoxicity, we next examined their ability to modulate neuroinflammatory responses by assessing their gliaprotective effects in the LPS-stimulated BV2 microglial model of neuroinflammation.^60^ LPS stimulation of BV2 microglial cells leads to microglial activation, characterized by elevated nitrite production and engagement of the TLR4/p38α MAPK/NF-κB signaling cascade, which in turn increases the release of pro-inflammatory cytokines, such as interleukin-6 (IL-6) and tumor necrosis factor-α (TNF-α). These processes play a fundamental role in the propagation of neuroinflammatory responses and subsequent neuronal injury.^61^ Treatment with LPS markedly increased the secretion of inflammatory mediators, namely nitrite, IL-6, and TNF-α in BV2 cultures (Figure 4A–C), confirming the robust activation of microglial inflammatory signaling. Pretreatment with dual hBChE/p38α MAPK inhibitors **8** and **21** significantly attenuated the LPS-induced nitrite and cytokine release, with inhibition levels comparable to those achieved with the selective p38α MAPK inhibitor **MW150**. LPS activates p38α MAPK in microglia to promote inflammatory outputs, while inhibition of p38α MAPK diminishes LPS-evoked nitrite production by BV2 cells, supporting a causal link between p38α MAPK activity and nitrite production.^62^ Importantly, no reduction of LPS-induced cytokine release was detected by treatment with the selective hBChE inhibitor **A**. Selective hBChE inhibitor **A** was evaluated only at 1 and 2.5 μM because concentrations of 5 μM and higher substantially reduced BV2 cell viability (Figure S4), potentially confounding the interpretation of its effects on inflammatory markers. The reduction of IL-6 and TNF-α suggests that both dual inhibitors effectively modulate the p38α-dependent inflammatory pathway, thereby suppressing microglial activation. p38α MAPK signaling also contributes to microglial apoptotic pathways as evidenced by induced cytotoxicity and further activation of caspase-3, an executor of apoptosis.^63^ In LPS-stimulated BV2 microglia, **21** significantly reduced LPS-induced cytotoxicity (Figure 4D), whereas both **8** and **21** significantly decreased caspase-3 activity (Figure 4E), consistent with an attenuation of TLR4-driven p38α signaling.

**Figure 4:**
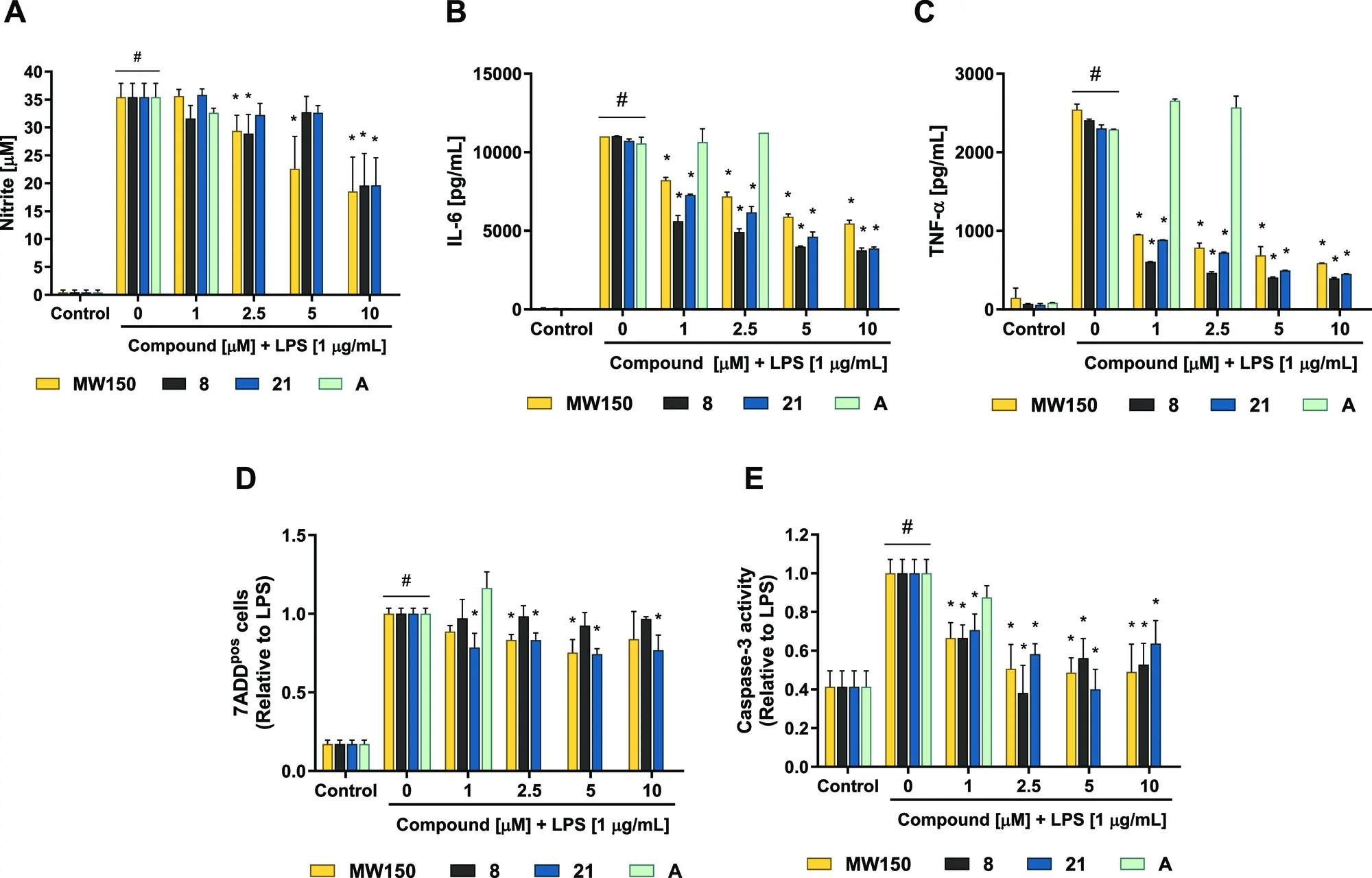
Gliaprotective activity of **MW150**, **8**, **21**, and the selective hBChE inhibitor **A** in the LPS-stimulated BV2 microglial model of neuroinflammation. BV2 cells were stimulated with LPS (1 μg/mL) in the absence or presence of compounds at concentrations 1, 2.5, 5, and 10 μM. Due to its cytotoxicity (Figure S4), selective hBChE inhibitor **A** was used only at concentrations of 1 and 2.5 μM. After 24 h, inflammatory markers—nitrite (A), IL-6 levels (B), and TNF-α levels (C), and cytotoxicity evaluated by flow cytometry analysis of 7-AAD staining (D) and caspase-3 activity (E) were measured. DMSO-treated cells were considered as control cells. Data are presented as means (±SEM) of at least three independent experiments. Statistical analysis: one-way ANOVA, *t* test; *p < 0.05 (compound/LPS *vs* LPS), ^#^ *p* < 0.05 (LPS *vs* control).

#### *In Vivo* Studies

To explore the procognitive benefits of dual hBChE/p38α MAPK inhibitors **8** and **21**, we utilized two complementary mouse models of cognitive impairment: the scopolamine model of cognitive deficit, mimicking cholinergic dysfunction as seen in AD,^64,65^ and the LPS-induced *in vivo* model of systemic inflammation^66,67^ that replicates neuroinflammation-associated cognitive decline characteristics of multiple neurodegenerative conditions. Three behavioral paradigms, the passive avoidance (PA), novel object recognition (NOR), and Morris water maze (MWM) tasks, were employed to evaluate the effects of compounds **8** and **21** on cognitive performance in adult male Albino Swiss (CD-1) and C57BL/6J mice. The *in vivo* performance of hBChE/p38α MAPK inhibitors **8** and **21** was compared to the *in vivo* activity of **MW150**, a selective p38α MAPK inhibitor, and the selective hBChE inhibitor **A**. To exclude false-positive results in the cognitive tests and to establish general effects of compounds on animal’s locomotion, rotarod and locomotor activity assays were first carried out to exclude motor deficits that could confound the results of *in vivo* assays. The rotarod assay revealed that the selective hBChE inhibitor **A** at 10 mg/kg reduced motor coordination at 24 rpm, while the remaining three compounds did not differ from the vehicle-treated group (Figure S6). In contrast, none of the assayed ligands altered spontaneous locomotor activity, indicating no generalized motor stimulation or suppression at the tested doses (Figure S7).

#### Scopolamine-Induced Amnesia

The procognitive effects of the selective p38α MAPK inhibitor **MW150**, dual hBChE/p38α MAPK inhibitors **8** and **21**, and the selective hBChE inhibitor **A** in the scopolamine-induced amnesia mouse model were first evaluated using the passive avoidance (PA) task, which measures long-term contextual memory by pairing a specific environment with an aversive foot shock. In this paradigm, cognition-enhancing compounds typically increase the step-through latency, reflecting improved memory retention as animals take longer to re-enter the shock-associated compartment. This outcome is interpreted as enhanced consolidation and recall of the aversive experience.^68^ In this fear-motivated task, the effect of three fixed doses of **MW150**, **8**, **21**, and **A** (5, 10, and 30 mg/kg) on scopolamine-induced cognitive dysfunction was assessed using adult male Albino Swiss (CD-1) mice. In the acquisition trial, no statistically significant differences in the step-through latencies were observed between the treatment groups (Figure 5A). In contrast to this, in the retention trial, a statistically significant prolonged step-through latency was observed between scopolamine-treated control mice *vs* vehicle-treated mice (*p* < 0.0001), and between scopolamine-treated control *vs* mice treated with a combination of scopolamine and dual hBChE/p38α MAPK inhibitors **8** at the dose of 5 mg/kg (*p* < 0.05) and **21** at the dose of 30 mg/kg (*p* < 0.01) (Figure 5A). **MW150**, a selective, brain-penetrant p38α MAPK inhibitor, has been previously evaluated in multiple *in vivo* models relevant to AD, neuroinflammation, and autism spectrum disorder,^15,69–71^ but not yet in the scopolamine-induced amnesia model. Neither **MW150** nor the selective hBChE inhibitor **A** prolonged the step-through latency in the PA task, showcasing the superior activity of dual ligands **8** and **21** in the scopolamine-induced amnesia model.

**Figure 5:**
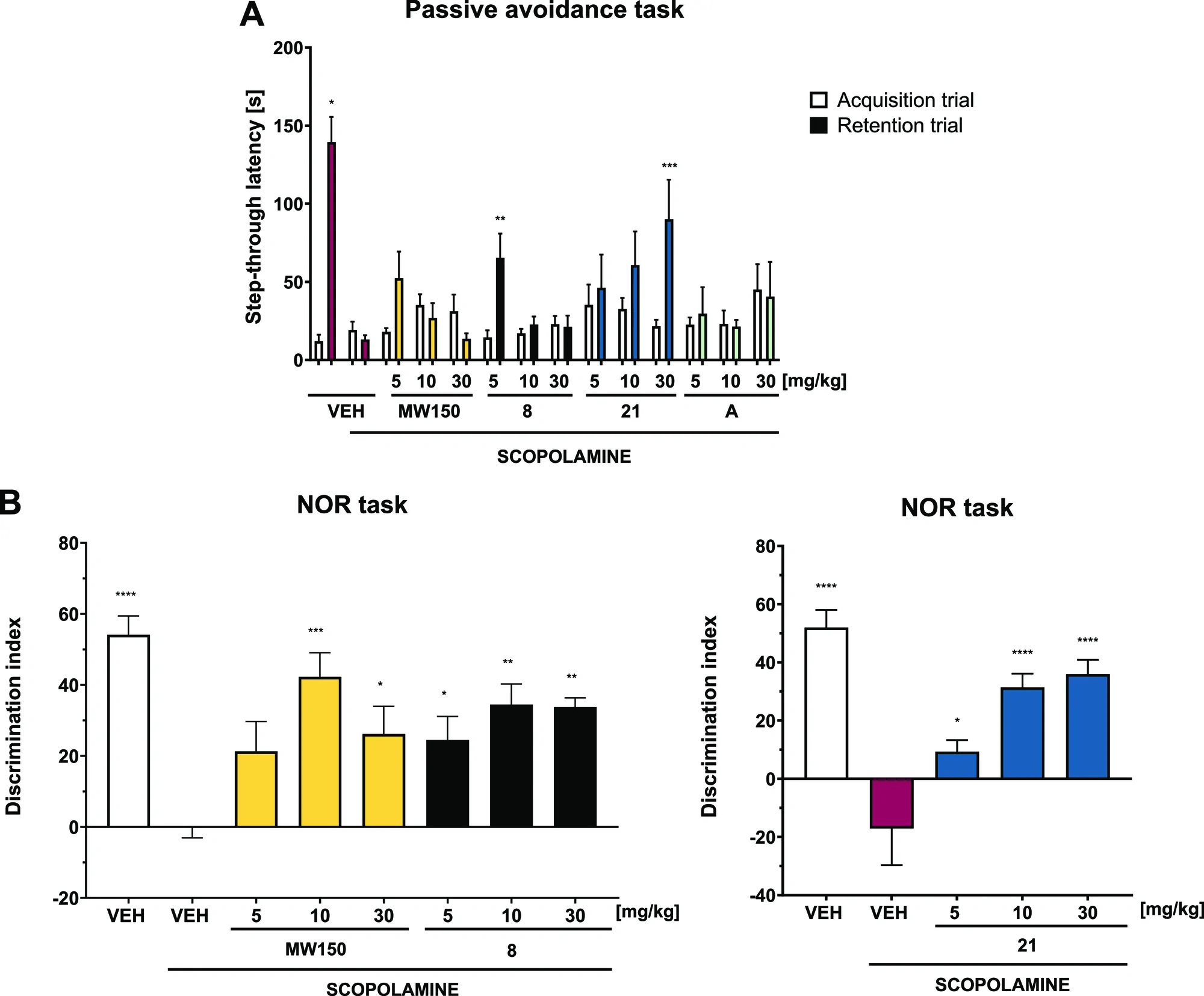
Effects of the selective p38α MAPK inhibitor **MW150**, dual hBChE/p38α MAPK inhibitors **8** and **21**, and the selective hBChE inhibitor **A** (doses 5, 10, and 30 mg/kg, i.p.) on scopolamine-induced cognitive deficits assessed in the (A) PA task and (B) NOR task. (A) PA task. Results are shown as mean step-through latency (±SEM) assessed in the acquisition trial and 24 h later (the retention trial) for *n* = 7–14 mice per group. Statistical analyses: repeated measures ANOVA followed by Dunnett’s *post hoc* comparison. Significance *vs* scopolamine-treated control mice in the respective trial: *p < 0.05, **p < 0.01, ***p < 0.001. (B) NOR task: scopolamine was administered s.c. Thirty min before the T1 trial, and **MW150**, **8** and **21** were administered i.p. 60 min before the T1 trial. Data represent mean discrimination index (±SEM) for *n* = 8–10 mice per group. Statistical analyses: one-way ANOVA followed by Dunnett’s *post hoc* comparison. Significance *vs* scopolamine-treated control: *p < 0.05, **p < 0.01, ***p < 0.001, ****p < 0.0001.

Next, the novel object recognition (NOR) task was used to assess recognition memory by measuring the animal’s preference for exploring a new object over a familiar one after a retention interval. Since the selective hBChE inhibitor **A** was not active in the PA task, it was not further assessed in the NOR task. Mice treated with scopolamine and pyridazines **MW150**, **8**, and **21** at doses of 10 and 30 mg/kg showed a statistically significant preference for novel objects compared to scopolamine-treated control (Figure 5B). At 5 mg/kg, significant effects were observed for dual hBChE/p38α MAPK inhibitors **8** and **21**, but not for selective p38α MAPK inhibitor **MW150**. However, the absence of statistical significance at 5 mg/kg for **MW150** does not establish a significant difference between the treatments. As compound **A** was not evaluated in the NOR task, the contribution of hBChE inhibition to the observed effects cannot be determined from these experiments. Overall, **8** and **21** demonstrated procognitive activity in the scopolamine model based on the NOR task at all the tested doses, although the relative contributions of p38α MAPK and hBChE inhibition require further investigation.

#### LPS Model of Neuroinflammation

Systemic intraperitoneal administration of lipopolysaccharide (LPS), which activates the TLR4 receptor complex on immune cells, subsequently initiates MyD88- and TRIF-dependent signaling that converges on the NF-κB and MAPK pathways, inducing robust cytokine release. Peripheral cytokines and vagal signaling subsequently activate microglial TLR4-NF-κB and p38α MAPK cascades within the CNS, leading to sustained neuroinflammation. This model reliably produces behavioral and cognitive alterations associated with neuroinflammatory processes, without requiring a direct CNS insult.^67,72–74^

The PA task was used to assess the effects of the selected compounds (*i*.*e*., **MW150**, **8** and **A**) on contextual learning and memory in the LPS systemic model in C57BL/6J mice. Each compound was administered at 5 mg/kg (the lowest dose of compound **8** shown to be active in both PA and NOR tasks in the scopolamine-induced amnesia model). This procedure was conducted in two phases (Figure S5): (1) before LPS administration—T1 (pre-LPS acquisition) and T2 (pre-LPS retention), and (2) following LPS administration—T3 (post-LPS acquisition) and T4 (post-LPS retention). In mice that did not receive LPS, no statistically significant differences were observed between treatment groups in step-through latency during either the acquisition (T1) or retention (T2) trial. This indicated that a single administration of **MW150**, dual hBChE/p38α MAPK inhibitor **8**, or the selective hBChE inhibitor **A** did not alter contextual, fear-motivated learning in memory-unimpaired mice. The 10-day administration of **MW150**, **8** or selective hBChE inhibitor **A** (5 mg/kg), together with a 6-day LPS treatment, significantly modulated contextual memory in mice. As shown in Figure 6, a single dose of these compounds did not alter cognition in memory-unimpaired mice in the PA task, whereas repeated administration led to significantly increased step-through latencies in the post-LPS acquisition (T3) trial [**MW150** and **A**: *p* < 0.05 *vs* LPS-treated control; **8**: *p* < 0.001 *vs* LPS-treated control], which indicated their positive effect on learning in mice. In contrast, no significant differences in step-through latencies were observed among groups in the post-LPS retention (T4) trial. Additional analyses comparing differences within each experimental group at trials before LPS (trials T1 and T2) and after LPS administration (trials T3 and T4) were also conducted (for details see Supporting Information, Figure S8), and ultimately revealed that 10-day treatment with **MW150** and **8** positively influenced the formation of long-term memory.

**Figure 6:**
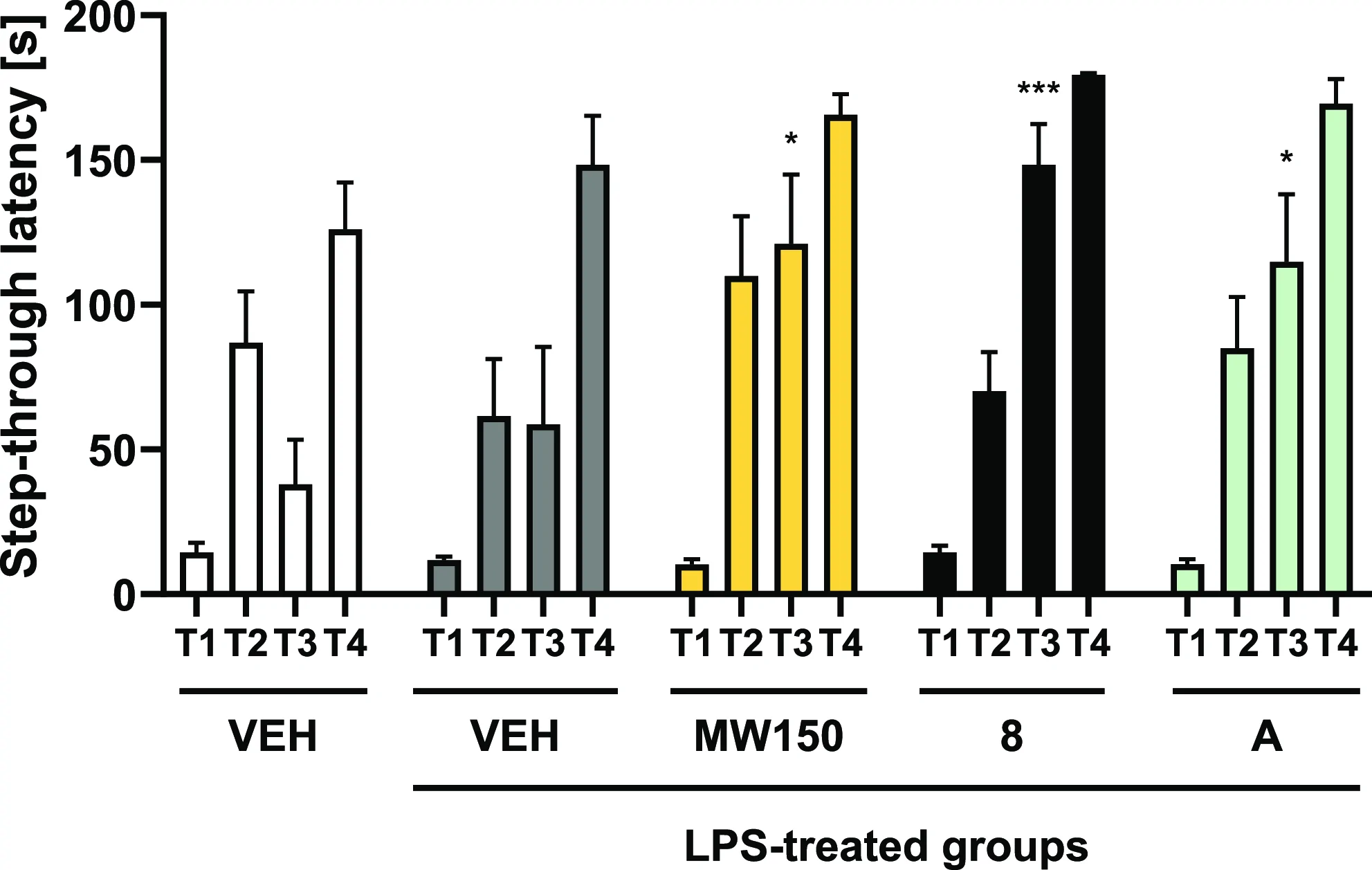
Effects of the selective p38α MAPK inhibitor **MW150**, dual hBChE/p38α MAPK inhibitor **8**, and the selective hBChE inhibitor **A** (5 mg/kg, i.p.) on fear-motivated contextual learning and memory measured using the passive avoidance (PA) task in mice not exposed to LPS (T1, T2 trials) and in mice exposed to LPS (T3 and T4 trials). A detailed protocol for drug administration is shown in Figure S5. Results are shown as the mean step-through latency (±SEM) assessed in the acquisition trials (T1, T3) and 24 h later (in the retention trials: T2, T4) for *n* = 8–10 mice. Statistical analysis: repeated measures ANOVA followed by Dunnett’s or Tukey’s *post hoc* comparisons. Significance *vs* LPS-treated control mice in the respective trial: *p < 0.05, ***p < 0.001.

The Morris water maze (MWM) task is a widely used assay for the evaluation of spatial learning and memory in rodents.^75,76^ To assess the impact of **MW150**, dual inhibitors **8** and **21**, and the selective hBChE ligand **A** on spatial memory in the MWM task, we used C57BL/6 mice, which outperform CD-1 mice in this task due to the visual impairments observed in the latter strain.^77^ Of note, the experiments were done in two series, which slightly differed from each other (discussed below), but importantly, the cumulative dose of LPS administered to mice was the same (*i*.*e*., 12 mg/kg). In the cellular LPS model, **8** and **21** were evaluated at the same concentrations. However, the *in vivo* LPS experiments were conducted at different doses: **8** was administered at 5 mg/kg, whereas **21** was tested at 10 and 30 mg/kg. These doses were selected based on the preceding scopolamine experiments—the PA task (Figure 5A), where **8** was active at lower concentration compared to **21**. Consequently, the results demonstrate the activity of each compound under its respective experimental conditions but do not allow their relative *in vivo* potency or efficacy to be directly compared.

In the MWM task (series 1: **MW150**, **8**, **A**), repeated administration of LPS, compared to vehicle, did not significantly influence escape latency in mice on each test day (Figure 7). Of note, on day 1 of the acquisition trial, the escape latencies in mice treated with LPS + **A**, LPS + **MW150**, and LPS + **8** (all at 5 mg/kg) were significantly reduced as compared to LPS-treated control (*p* < 0.05, *p* < 0.05, and *p* < 0.001, respectively). This effect was still maintained on day 2 only in **8**-treated mice (*p* < 0.05 *vs* LPS-treated control) (Figure 7). As shown in Figure 7 (series 2 of the MWM task: **21**), escape latencies did not differ significantly among groups on day 1. However, on day 2, escape latencies in the LPS-treated control group were significantly longer (*p* < 0.01) than in vehicle-treated mice (*p* < 0.01) and in the LPS + **21**-treated groups (both doses, *p* < 0.05). Statistically significantly reduced escape latencies were also noted on day 3 in vehicle-treated mice (*p* < 0.0001) and LPS + **21**-treated mice (10 mg/kg, *p* < 0.001 and 30 mg/kg, *p* < 0.05) compared to LPS-treated control (Figure 7). The results show that **21** outperformed selective p38α MAPK inhibitor **MW150**, and also neflamapimod (at the same doses—10 mg/kg and 30 mg/kg)—this selective p38α MAPK inhibitor was effective in improving spatial learning only on day 3 of the acquisition phase of the MWM task.^45^

**Figure 7:**
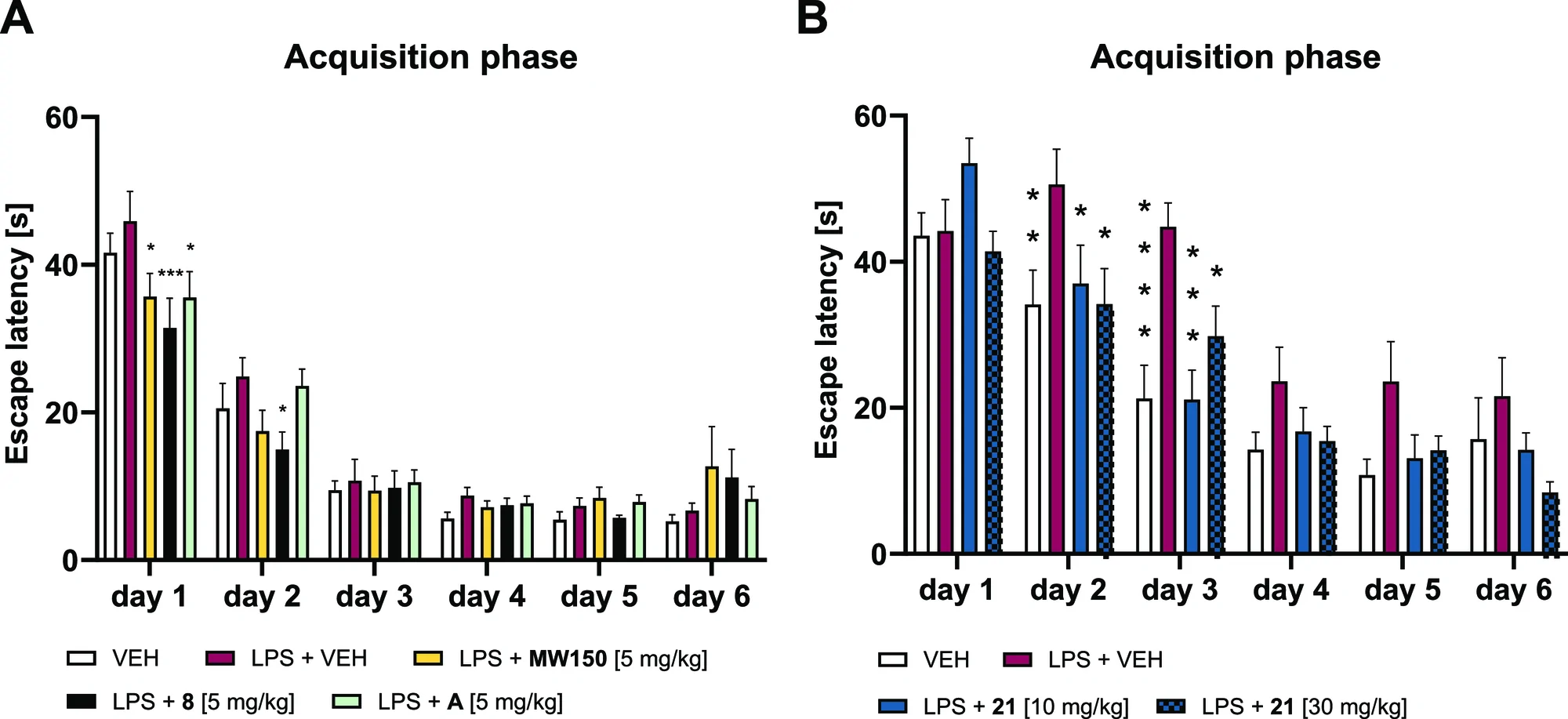
Effects of the selective p38α MAPK inhibitor **MW150**, dual hBChE/p38α MAPK inhibitors **8** and **21**, and the selective hBChE inhibitor **A** (dose: 5 mg/kg i.p., unless indicated otherwise) on spatial learning measured as escape latency assessed in the Morris water maze (MWM) task. LPS was administered at a cumulative dose of 12 mg/kg intraperitoneally 5 h before and the tested compounds were administered 60 min before the first daily trial of the acquisition phase (days 1–6). Results are shown as mean latency [s] to reach the hidden platform ± SEM for *n* = 7–10 mice per group. Statistical analysis: repeated measures ANOVA followed by Dunnett’s *post hoc* comparison. Significance *vs* LPS-treated control on the respective test day: *p < 0.05, **p < 0.01, ***p < 0.001, ****p < 0.0001.

The effect on memory retention was evaluated on day 7—the drug-off retention trial of the MWM task—where the following parameters were measured: (i) latency to northwest (NW) zone entry, (ii) time in NW zone, (iii) time spent at the target zone (*i*.*e*., the former platform location), and (iv) the number of NW zone entries. *Post hoc* analysis showed a statistically significant difference in the time spent at the target zone between the LPS-treated control group and **LPS+8**-treated group (*p* < 0.05, Figure 8D), and the LPS-treated control group and LPS**+21**-treated groups (at both doses, *p* < 0.001, Figure 8H). Considering this, it can be concluded that dual hBChE/p38α MAPK inhibitors **8** and **21** positively affected certain aspects of spatial memory retention, as measured in the MWM task. Specifically, during the drug-off retention trial on day 7 of the MWM task, both increased the time spent in the target NW zone. It should also be noted that the reference drug, rivastigmine, previously tested under the same laboratory conditions, was not effective in the LPS-induced neuroinflammation model accompanied by memory decline.^45^

**Figure 8:**
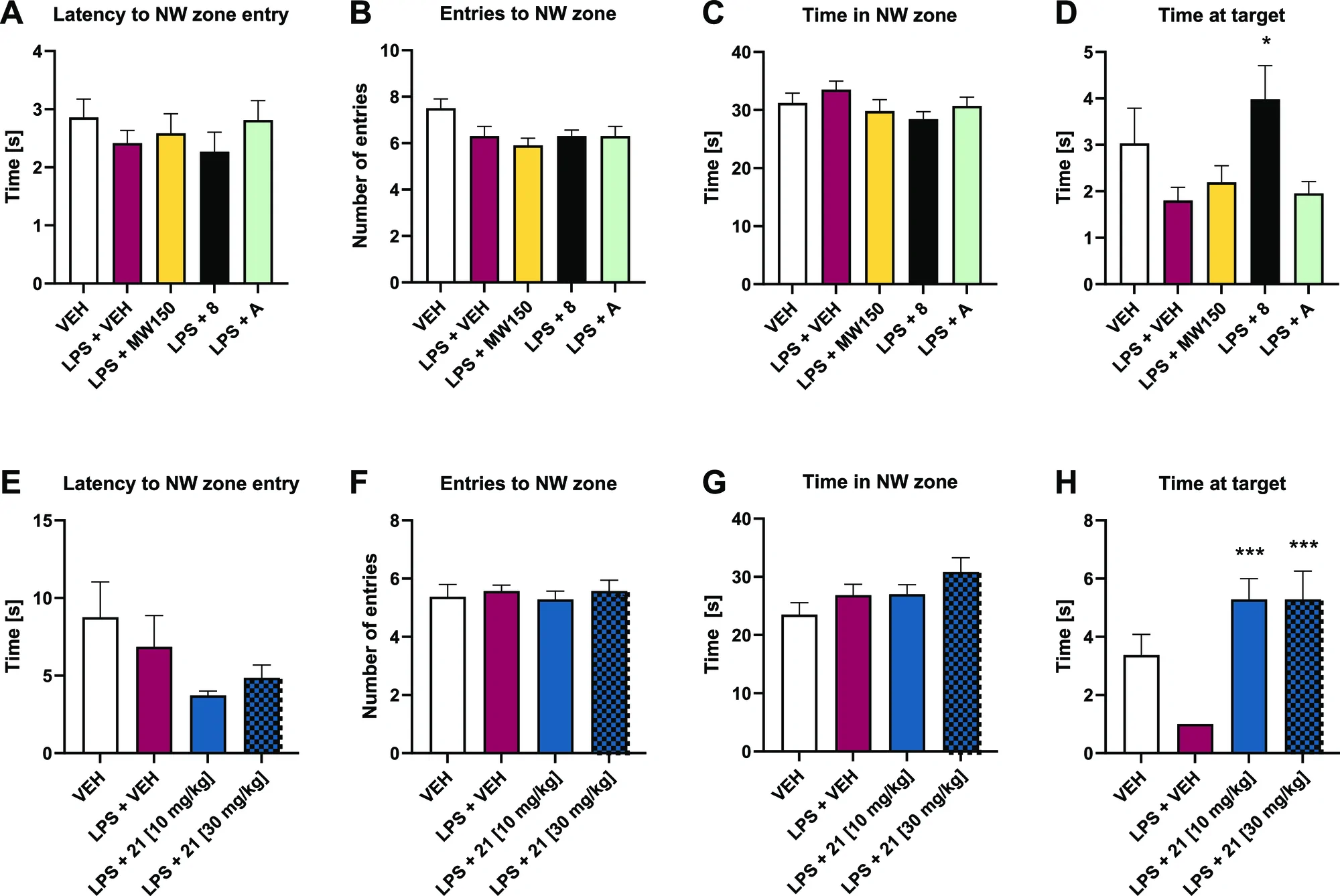
Effects of the selective p38α MAPK inhibitor **MW150**, dual hBChE/p38α MAPK inhibitors **8** and **21**, and the selective hBChE inhibitor **A** (dose: 5 mg/kg i.p., unless indicated otherwise) on memory retention measured in the mouse MWM task on day 7 of testing. Results are shown as (A, E) mean latency to NW zone entry ± SEM, (B, F) mean number of NW zone entries ± SEM, (C, G) mean time spent in NW zone ± SEM, and (D, H) mean time spent at target zone (*i*.*e*., the former platform location) ± SEM for *n* = 7–10. Statistical analysis: one-way ANOVA followed by Dunnett’s (A–C, E–H) or Sidak’s (D) *post hoc* comparison. Significance *vs* LPS-treated control group: *p < 0.05, ***p < 0.001.

In the two series of MWM experiments, we used two distinct protocols of LPS administration, but the total dose of LPS was the same (12 mg/kg). In experiment from series 1, LPS was administered in a relatively front-loaded scheme (three consecutive 2.5 mg injections followed by three 2 mg injections), resulting in a more abrupt early peak in behavioral alterations. In contrast, the experiment in series 2 used a tapered scheme (2.5 mg × 2 days, 2 mg × 2 days, 1.5 mg × 2 days), generating a more sustained change of animals’ behavior. Although these two LPS paradigms were matched for cumulative dose, they differed in temporal distribution, which influenced the magnitude and the onset of behavioral changes. This factor also contributed to the variability observed between the control groups in both series of experiments. This distinction is relevant because neuroinflammation and the action of LPS are strongly time-dependent and nonlinear. Previous studies have shown that equal cumulative LPS doses administered with different spacing or intensity can yield different CNS outcomes (*e*.*g*., variability in cytokine profiles, microglial priming, sickness behavior, and learning deficits).^78,79^ Moreover, lower repeated LPS exposures may paradoxically elicit stronger long-term microglial reactivity than higher-intensity peaks due to TLR4 signaling dynamics and transcriptional sensitization.^80^

Taken together, the first experiment produced a rapid, short-lasting behavioral effect in the MWM task, whereas in the second experiment the doses were gradually tapered, leading to a more prolonged behavioral effect. Numerous studies show that even when the total dose is identical, differences in timing can lead to distinct inflammatory responses, cytokine profiles, and degrees of learning and memory impairment. Thus, the more severe deficit in the MWM task in the second experiment is most likely attributable to differences in the kinetics of inflammation rather than procedural errors or differences in animals. Importantly, some variability between control groups (vehicle *vs* LPS) across independent experiments is expected due to intrinsic biological variability, housing microenvironment, LPS batch-to-batch differences, and baseline immune state differences across cohorts. Nevertheless, in both experiments the direction of the observed effect (LPS-induced learning deficits) remained consistent, supporting the reproducibility of the model.

Forty-eight hours after completion of the last LPS injection, mice from the LPS+**21** group and the respective vehicle and LPS control groups were euthanized, and their brain structures, *i*.*e*., hippocampus and frontal cortex, were collected and analyzed for inflammation-related markers. Treatment with **21** did not change the expression of these inflammatory markers 48 h after the last administration of LPS compared to the vehicle and the LPS control groups (Supporting Information, Figure S9). The absence of significant changes at this delayed end point does not exclude transient attenuation of p38α MAPK-dependent signaling during the peak inflammatory phase. Such early modulation may have limited downstream synaptic dysfunction and preserved memory-related neuronal circuits, resulting in behavioral benefits that persisted after the acute inflammatory response had subsided.^69,81^

## Conclusions

We report the rational design and optimization of pyridazine-based MTDLs that concurrently inhibit p38α MAPK and hBChE, two mechanistically complementary targets implicated in neuroinflammation and cholinergic imbalance in AD. Evaluation of the pyridazine series identified dual hBChE/p38α MAPK inhibitors **8** and **21** as lead compounds with submicromolar potencies against both enzymes. X-ray crystallography revealed their binding modes, including ATP-site engagement in p38α MAPK and key π–cation and π–stacking interactions within the hBChE active-site gorge. Both compounds exhibited high blood–brain barrier permeability and low intrinsic cytotoxicity and attenuated LPS-induced microglial responses by decreasing nitrite release, proinflammatory cytokine production, and caspase-3 activity. However, only **21** significantly reduced LPS-induced cytotoxicity in BV2 microglial cells. *In vivo*, dual inhibitor **21**, in particular, improved cognitive performance in mouse models of scopolamine-induced amnesia and LPS-evoked neuroinflammation. These findings support the feasibility of simultaneously modulating neuroinflammatory and cholinergic pathways with a single molecular entity. The dual inhibitor of hBChE and p38α MAPK **21** thus represents a promising lead for further optimization and preclinical evaluation as a therapeutic candidate for neurodegenerative conditions characterized by neuroinflammation and cognitive decline.

## Experimental Section

### Chemistry—General Information

The reagents and solvents were used as received from commercial suppliers (Sigma Aldrich, Merck, TCI Europe, Carlo Erba, BLDPharm, Fluorochem, Acros, Merck, Fluka, Abcr, Penta Chemicals United). Tetrahydrofuran (THF) was distilled from sodium/benzophenone and stored under Ar over 4 Å molecular sieves prior to use. After extraction, organic phases were dried over anhydrous sodium sulfate. Reactions were monitored using analytical thin-layer chromatography (TLC) on silica gel 60 F_254_ Al plates and Merck Silica gel 60 RP-18 F_254_. Developed plates were inspected under UV light and, if necessary, visualized with ninhydrin or 2,4-dinitrophenylhydrazine stains. Nuclear magnetic resonance spectra were recorded with a Bruker Avance III 400 MHz and Avance NEO Ascend 500 MHz spectrometers (Bruker, Billerica, MA, USA) at 400 or 500 MHz for ^1^H, 100 or 126 MHz for ^13^C, respectively, using DMSO-*d*_6_, CDCl_3_, acetone-*d*_6_, or MeOH-*d*_4_ with TMS as the internal standard, as solvents. Chemical shifts are reported in *parts per million* (ppm), ^1^H spectra are calibrated to the TMS peak (0 ppm) or the residual nonperdeuterated solvent peak, ^13^C spectra to the central peak of the deuterated solvent. The multiplicities are reported as follows: s (singlet), d (doublet), t (triplet), q (quartet), m (multiplet), dd (doublet of doublets), ddd (doublet doublet of doublets), td (triplet of doublets), qd (quartet of doublets), and br (broad). Coupling constants (*J*) are reported in Hertz (Hz). Mass spectra were recorded on Thermo Scientific Q Exactive Plus LC-MS/MS spectrometers (Thermo Fisher Scientific Inc., Waltham, MA, USA**)** and processed in Xcalibur software (Thermo Fisher Scientific, Waltham, Mass, USA). IR spectra were recorded on a Thermo Nicolet FT-IR spectrophotometer (Thermo Fisher Scientific Inc., Waltham, MA, USA**)**. Column chromatography was performed on silica gel (Silica gel 60, particle size: 0.035–0.070 mm, Merck KGaA) or using the Reveleris Prep purification system (Donau Lab, Czech Republic). UHPLC analyses were performed on Thermo Scientific Dionex UltiMate 3000 modular system (Thermo Fisher Scientific Inc., Waltham, MA, USA). The general method (method I) used a Waters Acquity UPLC HSS C18 SB column (2.1 × 50 mm^2^, 1.8 μm; Waters Corporation, Milford, MA, USA) thermostatted at 40 °C, with: injection volume, 1 μL; sample, 0.2–0.5 mg/mL in MeOH or MeCN; flow rate, 0.4 mL/min; detector λ, 254 nm; mobile phase A: 0.1% TFA (v/v) in water; mobile phase B: MeCN. Gradient: 0–2 min, 10% B; 2–8 min, 10%–90% B; 8–10 min, 90% B. The purities of the tested compounds were established to be ≥95%, as determined by UHPLC.

#### General Procedure 1 (GP1)—Nucleophilic Aromatic Substitution

To a solution of 2-chloropyridazine (1 equiv) in absolute EtOH (10–15 mL), the corresponding amine, hydroxylamine hydrochloride, or hydrazine (5–10 equiv) was added, and the reaction mixture was stirred at 95 °C for 24 h. The volatiles were removed under reduced pressure, and the product was purified according to *GP2*.

#### General Procedure 2 (GP2)—RP-CC Purification

Compounds were purified by reversed-phase column chromatography (RP-CC) (Isolera One Flash Chromatography from Biotage or puriFlash 5.250 LC system from Advion Interchim Scientific) using Biotage Sfär C18 Duo 100 Å 30 μm, 30 g column and a gradient of 0.1% TFA in deionized water and MeCN as eluent (gradient 0–100%) MeCN in 6 column volumes (300 mL); 100% MeCN for 2 column volumes (100 mL). Following the RP-CC, fractions containing the product were combined, and organic volatiles were evaporated *in vacuo*. The remaining aqueous solution was made alkaline (pH = 13–14) with 1 M NaOH and extracted with DCM (2 × 20 mL). The combined organic phase was dried over anhydrous sodium sulfate, filtered, and the volatiles were evaporated *in vacuo* to afford the pure product.

### Synthesis

#### 6-Chloro-3-(naphthalen-2-yl)-4-(pyridin-4-yl)­pyridazine **5a**

To an ice bath cooled (0 °C) solution of 2-naphthoyl chloride (10.0 g, 52.46 mmol) in THF (40 mL), Et_3_N (21.9 mL, 157.38 mmol) and *N*,*O*-dimethylhydroxylamine hydrochloride (6.14 g, 62.95 mmol) were added, and stirred at rt for 24 h. The solvent was removed *in vacuo*, the residue partitioned between EtOAc (200 mL) and water (150 mL), the organic phase washed with saturated sodium bicarbonate (50 mL), dried over anhydrous sodium sulfate, filtered, and volatiles evaporated *in vacuo* to afford crude *N*-methoxy-*N*-methyl-2-naphthamide **1a**. Yield: 10.4 g (48.32 mmol, 92%) of beige semisolid. ESI-HRMS: *m*/*z* = 216.10181 (MH^+^); C_13_H_14_NO_2_ requires: *m*/*z* = 216.10191 (MH^+^). ^1^H NMR (400 MHz, CDCl_3_) δ = 3.41 (s, 3H), 3.56 (s, 3H), 7.49–7.57 (m, 2H), 7.75 (dd, *J* = 8.5, 1.7 Hz, 1H), 7.83–7.87 (m, 2H), 7.88–7.92 (m, 1H), 8.21–8.24 (m, 1H).

(Pyridin-4-ylmethyl)lithium was prepared by the addition of lithium diisopropylamide (1.8 M in THF/heptane/ethylbenzene; 25.8 mL, 46.46 mmol) to 4-picoline (4.53 mL, 46.46 mmol) in THF (20 mL) under Ar at −70 °C. After the addition was complete, the reaction mixture was stirred for an additional 30 min. Then, *N*-methoxy-*N*-methyl-2-naphthamide **1a** (5.0 g, 23.23 mmol) was dissolved in THF (15 mL) and added dropwise. The reaction mixture was stirred on an ice bath (0 °C) for 3 h before being quenched by the addition of 10% citric acid (15 mL). The volatiles were removed *in vacuo*, the residue partitioned between EtOAc (150 mL) and 4 M NaOH (20 mL), the organic phase separated, dried over anhydrous sodium sulfate, filtered, and volatiles evaporated under oil pump vaccuum to afford crude 1-(naphthalen-2-yl)-2-(pyridin-4-yl)­ethan-1-one **2a**. Yield: 4751 mg (19.21 mmol, 83%) of yellow crystals. ESI-HRMS: *m*/*z* = 248.10689 (MH^+^); C_17_H_14_NO requires: *m*/*z* = 248.10699 (MH^+^). ^1^H NMR (400 MHz, CDCl_3_) δ = 4.43 (s, 2H), 7.24–7.27 (m, 2H), 7.55–7.66 (m, 2H), 7.87–7.94 (m, 2H), 7.98 (ddd, *J* = 8.0, 1.5, 0.7 Hz, 1H), 8.05 (dd, *J* = 8.6, 1.8 Hz, 1H), 8.52–8.54 (m, 1H), 8.56–8.59 (m, 2H).

To an ice-bath cooled (0 °C) solution of 1-(naphthalen-2-yl)-2-(pyridin-4-yl)­ethan-1-one **2a** (6.13 g, 24.79 mmol) in anhydrous DMF (20 mL), sodium hydride (60 wt% dispersion in mineral oil; 1.09 g, 27.27 mmol) was slowly added under Ar, stirred for 30 min at 0 °C, then ethyl 2-bromoacetate (3.02 mL, 27.27 mmol) was added and the mixture stirred at rt for 24 h. Water (150 mL) and EtOAc (100 mL) were added, the organic phase separated, dried over anhydrous sodium sulfate, filtered, volatiles evaporated *in vacuo* and ethyl 4-(naphthalen-2-yl)-4-oxo-3-(pyridin-4-yl)­butanoate **3a** isolated by column chromatography on silica (EtOAc/*n*-hex = 4:1). Yield: 5.78 g (17.35 mmol, 70%) of yellow solid. ESI-HRMS: *m*/*z* = 334.14341 (MH^+^); C_21_H_20_NO_3_ requires: *m*/*z* = 334.14377 (MH^+^). ^1^H NMR (400 MHz, CDCl_3_) δ = 1.18 (t, *J* = 7.1 Hz, 3H), 2.77 (dd, *J* = 17.0, 5.3, Hz, 1H), 3.42 (dd, *J* = 17.0, 9.4 Hz, 1H), 4.10 (q, *J* = 7.1 Hz, 2H), 5.27 (dd, *J* = 9.4, 5.3 Hz, 1H), 7.28–7.33 (m, 2H), 7.46–7.58 (m, 2H), 7.74–7.84 (m, 2H), 7.89 (d, *J* = 8.0 Hz, 1H), 7.98 (dd, *J* = 8.6, 1.8 Hz, 1H), 8.49 (d, *J* = 1.7 Hz, 1H), 8.51–8.55 (m, 2H). ^13^C NMR (101 MHz, CDCl_3_) δ = 14.14, 38.05, 48.70, 61.04, 123.42, 124.22, 126.93, 127.75, 128.70, 128.84, 129.70, 130.77, 132.38, 133.08, 135.69, 147.43, 150.25, 171.43, 197.43.

Ethyl 4-(naphthalen-2-yl)-4-oxo-3-(pyridin-4-yl)­butanoate **3a** (4.74 g, 14.22 mmol) and hydrazine (35 wt% in water; 6.51 mL, 71.10 mmol) in anhydrous EtOH (30 mL) were stirred in pressure tube at 95 °C for 24 h. The solvent was removed *in vacuo*, and the residue was partitioned between EtOAc (2 × 50 mL) and water (50 mL). The organic phase was separated, dried over anhydrous sodium sulfate, filtered, and volatiles evaporated *in vacuo*. The product, 6-(naphthalen-2-yl)-5-(pyridin-4-yl)-4,5-dihydropyridazin-3­(2*H*)-one **4a**, was isolated by recrystallization from EtOH. Yield: 3643 mg (12.09 mmol, 85%) of yellow solid. ESI-HRMS: *m*/*z* = 302.12830 (MH^+^); C_19_H_16_N_3_O requires: *m*/*z* = 302.12879 (MH^+^). ^1^H NMR (400 MHz, DMSO-*d*_*6*_) δ = 2.65 (dd, *J* = 16.9, 1.6 Hz, 1H), 3.19 (dd, *J* = 16.9, 7.8 Hz, 1H), 4.96 (d, *J* = 7.4 Hz, 1H), 7.22–7.27 (m, 2H), 7.49–7.57 (m, 2H), 7.89–7.95 (m, 3H), 8.04 (dd, *J* = 8.7, 1.8 Hz, 1H), 8.21 (d, *J* = 1.8 Hz, 1H), 8.48–8.54 (m, 2H), 11.33 (s, 1H). ^13^C NMR (101 MHz, DMSO-*d*_*6*_) δ = 33.96, 37.04, 122.39, 122.94, 126.04, 126.70, 127.15, 127.54, 128.28, 128.53, 132.41, 132.65, 133.23, 147.62, 148.91, 150.32, 165.65.

To a solution of 6-(naphthalen-2-yl)-5-(pyridin-4-yl)-4,5-dihydropyridazin-3­(2*H*)-one **4a** (2.51 g, 8.33 mmol) in acetic acid (20 mL), bromine (0.51 mL, 10.00 mmol) was added, and the mixture was stirred at 70 °C for 4 h. The volatiles were removed *in vacuo* and residue partitioned between EtOAc (4 × 30 mL) and saturated sodium bicarbonate (50 mL), the organic phase separated, dried over anhydrous sodium sulfate, filtered, volatiles evaporated *in vacuo* and 6-(naphthalen-2-yl)-5-(pyridin-4-yl)­pyridazin-3­(2*H*)-one **5ai** isolated by column chromatography on silica (DCM/MeOH = 20:1). Yield: 2368 mg (7.91 mmol, 95%) of off-brown solid. ESI-HRMS: *m*/*z* = 300.11267 (MH^+^); C_19_H_14_N_3_O requires: *m*/*z* = 300.11314 (MH^+^). ^1^H NMR (400 MHz, DMSO-*d*_*6*_) δ = 7.06 (s, 1H), 7.17–7.24 (m, 3H), 7.47–7.56 (m, 2H), 7.75–7.85 (m, 3H), 7.85–7.89 (m, 1H), 8.45–8.49 (m, 2H), 13.52 (s, 1H). ^13^C NMR (101 MHz, DMSO-*d*_*6*_) δ = 123.34, 126.45, 126.58, 126.87, 127.42, 127.55, 128.14, 128.47, 129.59, 132.32, 132.41, 132.63, 143.43, 143.75, 144.90, 149.63, 160.11.

6-(Naphthalen-2-yl)-5-(pyridin-4-yl)­pyridazin-3­(2*H*)-one **5ai** (1.17 g, 3.91 mmol) and POCl_3_ (1.01 mL, 10.75 mmol) in MeCN (30 mL) were stirred under argon at 85 °C for 3 h. The mixture was cooled on an ice-bath, water (50 mL) and solid K_2_CO_3_ added till the effervescence ceased, then the organics were extracted with EtOAc (2 × 50 mL), the organic phases were separated, dried over anhydrous sodium sulfate, filtered and volatiles evaporated *in vacuo* to afford crude 6-chloro-3-(naphthalen-2-yl)-4-(pyridin-4-yl)­pyridazine **5a**. Yield: 1068 mg (3.36 mmol, 86%) of viscous brown oil. ESI-HRMS: *m*/*z* = 318.07922 (MH^+^); C_19_H_13_ClN_3_ requires: *m*/*z* = 318.07925 (MH^+^). ^1^H NMR (400 MHz, CDCl_3_) δ = 7.13–7.15 (m, 2H), 7.29 (dd, *J* = 8.5, 1.8 Hz, 1H), 7.44–7.53 (m, 2H), 7.57 (*s*, 1H), 7.71 (d, *J* = 8.6 Hz, 1H), 7.73–7.81 (m, 2H), 7.99 (d, *J* = 1.9 Hz, 1H), 8.55–8.58 (m, 2H). ^13^C NMR (101 MHz, CDCl_3_) δ = 123.40, 126.57, 126.79, 127.49, 127.76, 128.25, 128.35, 128.67, 130.26, 132.15, 132.96, 133.47, 138.89, 143.66, 150.37, 155.65, 158.68.

#### 6-Chloro-3-(naphthalen-1-yl)-4-(pyridin-4-yl)­pyridazine **5b**

To an ice bath cooled (0 °C) solution of 1-naphthoyl chloride (15.71 mL, 104.25 mmol) in THF (80 mL), Et_3_N (43.47 mL, 312.75 mmol) and *N*,*O*-dimethylhydroxylamine hydrochloride (10.17 g, 104.25 mmol) were added, and stirred at rt for 24 h. The solvent was removed *in vacuo*, the residue partitioned between Et_2_O (100 mL) and water (30 mL), the organic phase washed with 1 M HCl (100 mL), 1 M NaOH (100 mL), dried over anhydrous sodium sulfate, filtered, volatiles evaporated *in vacuo* and *N*-methoxy-*N*-methyl-1-naphthamide **1b** isolated by column chromatography on silica (EtOAc/*n*-Hex = 1:3). Yield: 11230 mg (52.17 mmol, 50%) of colorles oil. ESI-HRMS: *m*/*z* = 216.10171 (MH^+^); C_13_H_14_NO_2_ requires: *m*/*z* = 216.10191 (MH^+^). ^1^H NMR (400 MHz, CDCl_3_) δ = 3.42 (bs, 6H), 7.40–7.58 (m, 4H), 7.78–7.94 (m, 3H). ^13^C NMR (101 MHz, CDCl_3_) δ = 32.79, 61.37, 124.37, 124.84, 124.94, 126.33, 126.94, 128.38, 129.65, 129.77, 133.17, 133.37, 170.21.

(Pyridin-4-ylmethyl)lithium was prepared by the addition of lithium diisopropylamide (1.8 M in THF/heptane/ethylbenzene; 58.0 mL, 104.40 mmol) to 4-picoline (10.16 mL, 104.40 mmol) in THF (35 mL) under Ar at −70 °C. After the addition was complete, the reaction mixture was stirred for an additional 45 min. Then, *N*-methoxy-*N*-methyl-1-naphthamide **1b** (11.23 g, 52.17 mmol) dissolved in THF (20 mL) was added dropwise and the reaction mixture was stirred on an ice bath (0 °C) for 3 h, before the reaction was quenched by the addition of 10% citric acid (15 mL). The volatiles were removed *in vacuo*, the residue partitioned between EtOAc (150 mL) and 1 M NaOH (20 mL), the organic phase separated, dried over anhydrous sodium sulfate, filtered, volatiles evaporated under oil pump vacuum and 1-(naphthalen-1-yl)-2-(pyridin-4-yl)­ethan-1-one **2b** isolated by column chromatography on silica (1. EtOAc/*n*-Hex = 1:4; 2. EtOAc/*n*-Hex = 1:1). Yield: 4817 mg (19.48 mmol, 37%) of brownish semisolid. ESI-HRMS: *m*/*z* = 248.10670 (MH^+^); C_17_H_14_NO requires: *m*/*z* = 248.10699 (MH^+^). ^1^H NMR (400 MHz, CDCl_3_) δ = 4.38 (s, 2H), 7.23–7.30 (m, 2H), 7.46–7.60 (m, 3H), 7.83–7.89 (m, 1H), 7.95 (dd, *J* = 7.3, 1.2 Hz, 1H), 8.01 (dd, *J* = 8.2, 1.0 Hz, 1H), 8.49–8.56 (m, 2H), 8.56–8.62 (m, 1H). ^13^C NMR (101 MHz, CDCl_3_) δ = 47.80, 124.34, 125.38, 125.63 (2 × Ar-C), 126.79, 128.45, 128.63, 130.33, 133.68, 134.05, 134.55, 145.07, 148.73, 199.09.

To an ice-bath cooled (0 °C) solution of 1-(naphthalen-1-yl)-2-(pyridin-4-yl)­ethan-1-one **2b** (4.64 g, 18.77 mmol) in anhydrous DMF (20 mL), sodium hydride (60 wt% dispersion in mineral oil; 0.83 g, 20.65 mmol) was added under Ar, stirred for 45 min at 0 °C, then ethyl 2-bromoacetate (2.29 mL, 20.65 mmol) was added and the mixture stirred at rt for 24 h. Water (100 mL) and EtOAc (50 mL) were added, the organic phase separated, dried over anhydrous sodium sulfate, filtered, volatiles evaporated *in vacuo* and ethyl 4-(naphthalen-1-yl)-4-oxo-3-(pyridin-4-yl)­butanoate **3b** isolated by column chromatography on silica (1. EtOAc/*n*-hex = 1:1; 2. EtOAc/*n*-hex = 4:1). Yield: 3880 mg (11.64 mmol, 62%) of brown oil. ESI-HRMS: *m*/*z* = 334.14306 (MH^+^); C_21_H_20_NO_3_ requires: *m*/*z* = 334.14377 (MH^+^). ^1^H NMR (400 MHz, CDCl_3_) δ = 1.23 (t, *J* = 7.1 Hz, 3H), 2.77 (dd, *J* = 17.0, 4.8 Hz, 1H), 3.52 (dd, *J* = 17.0, 10.0 Hz, 1H), 4.15 (qq, *J* = 6.9, 3.7 Hz, 2H), 5.12 (dd, *J* = 10.0, 4.7 Hz, 1H), 7.18–7.23 (m, 2H), 7.41–7.56 (m, 3H), 7.79–7.84 (m, 1H), 7.95 (dd, *J* = 8.2, 1.0 Hz, 1H), 7.99 (dd, *J* = 7.2, 1.2 Hz, 1H), 8.29–8.37 (m, 1H), 8.43–8.51 (m, 2H). ^13^C NMR (101 MHz, CDCl_3_) δ = 14.26, 37.60, 52.25, 61.21, 123.35, 124.36, 125.39, 126.69, 127.99, 128.22, 128.48, 130.57, 133.14, 133.93, 135.32, 146.40, 150.57, 171.66, 200.80.

Alternatively, ethyl 4-(naphthalen-1-yl)-4-oxo-3-(pyridin-4-yl)­butanoate **3b** was prepared as shown in Scheme S1 (Supporting Information). To a cooled (−78 °C) solution of 1-(naphthalen-1-yl)-2-(pyridin-4-yl)­ethan-1-one **2b** (2.00 g, 8.09 mmol) in anhydrous THF (64 mL), sodium bis­(trimethylsilyl)­amide NaHMDS (1 M solution in THF; 8.09 mL, 8.09 mmol) was added under Ar, stirred for 45 min at–78 °C, then ethyl 2-bromoacetate (0.89 mL, 8.09 mmol) was added and the mixture stirred at–78 °C for 24 h. Ethyl 4-(naphthalen-1-yl)-4-oxo-3-(pyridin-4-yl)­butanoate **3b** was isolated by column chromatography on silica (EtOAc/*n*-hex = 1:1). Yield: 728 mg (2.18 mmol, 27%) of yellow solid. Ethyl 2-(4-(2-(naphthalen-1-yl)-2-oxoethylidene)­pyridin-1­(4*H*)-yl)­acetate **3d** (Scheme S1B in Supporting Information) was isolated as the major side product. ESI-HRMS: *m*/*z* = 334.14355 (MH^+^); C_21_H_20_NO_3_ requires: *m*/*z* = 334.14377 (MH^+^). ^1^H NMR (500 MHz, DMSO-*d*_*6*_) δ = δ 1.16–1.29 (m, 3H), 4.19 (q, *J* = 6.5, 5.9 Hz, 2H), 4.86 (s, 2H), 5.60 (s, 1H), 6.45 (d, *J* = 7.4 Hz, 1H), 7.38–7.47 (m, 1H), 7.48 (d, *J* = 7.7 Hz, 3H), 7.54 (t, *J* = 5.1 Hz, 2H), 7.89 (dd, *J* = 15.7, 7.9 Hz, 2H), 8.30 (d, *J* = 8.0 Hz, 1H), 8.46 (d, *J* = 7.5 Hz, 1H). ^13^C NMR (126 MHz, DMSO-*d*_*6*_) δ = 13.95, 55.65, 61.37, 99.37, 112.17, 115.55, 123.92, 125.01, 125.60, 125.78, 126.10, 127.91, 128.13, 129.85, 133.23, 138.27, 139.55, 143.01, 149.44, 168.19, 188.42.

Ethyl 4-(naphthalen-1-yl)-4-oxo-3-(pyridin-4-yl)­butanoate **3b** (3.80 g, 11.40 mmol) and hydrazine (35 wt% in water; 5.22 mL, 57.00 mmol) in anhydrous EtOH (30 mL) were stirred at 95 °C for 24 h. The solvent was removed *in vacuo* and residue partitioned between EtOAc (1 × 50 mL) and water (50 mL), the organic phase separated, dried over anhydrous sodium sulfate, filtered, volatiles evaporated *in vacuo* to afford crude 6-(naphthalen-1-yl)-5-(pyridin-4-yl)-4,5-dihydropyridazin-3­(2*H*)-one **4b**, which was partially purified by column chromatography on silica (1. DCM/MeOH = 20:1; 2. DCM/MeOH = 15:1) and subsequently by RP-CC (*GP2*). Yield: 559 mg of orange semisolid (not pure). 6-(Naphthalen-1-yl)-5-(pyridin-4-yl)-4,5-dihydropyridazin-3­(2*H*)-one **4b** and Pd/C (50 mg) in toluene (20 mL) were stirred at 110 °C for 24 h, the mixture cooled to room temperature, filtered through a pad of Celite, filtrate evaporated and 6-(naphthalen-1-yl)-5-(pyridin-4-yl)­pyridazin-3­(2*H*)-one **5bi** isolated by column chromatography on silica (DCM/MeOH = 20:1). Yield: 157 mg (0.52 mmol, 5% over three steps) of pale orange solid. ESI-HRMS: *m*/*z* = 300.11286 (MH^+^); C_19_H_14_N_3_O requires: *m*/*z* = 300.11314 (MH^+^). ^1^H NMR (400 MHz, DMSO-*d*_*6*_) δ = 7.01–7.09 (m, 2H), 7.11 (s, 1H), 7.37–7.51 (m, 4H), 7.58–7.65 (m, 1H), 7.87–7.91 (m, 1H), 7.92 (ddd, *J* = 7.7, 1.8, 0.7 Hz, 1H), 8.22–8.31 (m, 2H), 13.53 (s, 1H). ^13^C NMR (101 MHz, DMSO-*d*_*6*_) δ = 122.67, 124.91, 125.10, 126.13, 126.61, 128.24, 128.36, 129.13, 129.18, 131.01, 132.64, 132.81, 143.38, 144.29, 144.40, 149.19, 160.31.

6-(Naphthalen-1-yl)-5-(pyridin-4-yl)­pyridazin-3­(2*H*)-one **5bi** (1.17 g, 3.91 mmol) and POCl_3_ (1.01 mL, 10.75 mmol) in MeCN (30 mL) were stirred under argon at 85 °C for 3 h. The mixture was cooled on an ice-bath, water (50 mL) and solid K_2_CO_3_ added till the effervescence ceased, then the organics were extracted with EtOAc (2 × 50 mL), the organic phases were separated, dried over anhydrous sodium sulfate, filtered, the volatiles evaporated *in vacuo* and 6-chloro-3-(naphthalen-2-yl)-4-(pyridin-4-yl)­pyridazine **5b** isolated by column chromatography on silica (EtOAc/*n*-hex = 1:1). Yield: 170 mg (0.53 mmol, 14%) of colorles oil. ESI-HRMS: *m*/*z* = 318.07906 (MH^+^); C_19_H_13_ClN_3_ requires: *m*/*z* = 318.07925 (MH^+^). ^1^H NMR (400 MHz, CDCl_3_) δ = 6.89–6.98 (m, 2H), 7.30 (dd, *J* = 7.1, 1.2 Hz, 1H), 7.35 (ddd, *J* = 8.3, 6.9, 1.4 Hz, 1H), 7.38–7.50 (m, 3H), 7.69 (s, 1H), 7.83–7.86 (m, 1H), 7.88 (dt, *J* = 8.3, 1.1 Hz, 1H), 8.30–8.38 (m, 2H). ^13^C NMR (101 MHz, CDCl_3_) δ = 122.67, 124.68, 124.99, 126.36, 127.01, 127.80, 128.52, 128.75, 130.03, 131.18, 132.45, 133.44, 140.40, 142.78, 149.95, 156.17, 158.95.

#### 6-(4-Methylpiperazin-1-yl)-3-(naphthalen-2-yl)-4-(pyridin-4-yl)­pyridazine 6 (MW150)^16^

Synthesized following *GP1* from **5a** (0.20 g, 0.63 mmol) and *N*-methylpiperazine (0.35 mL, 3.15 mmol) and isolated following *GP2*. Yield: 106 mg (0.28 mmol, 44%) of off-yellow semisolid. ESI-HRMS: *m*/*z* = 382.20187 (MH^+^); C_24_H_24_N_5_ requires: *m*/*z* = 382.20262 (MH^+^). ν_max_ 3045, 2937, 1844, 2796, 1583, 1550, 1498, 1441, 1430, 1375, 1314, 1287, 1240, 1226, 1165, 1143, 1051, 1033, 1004, 952, 862, 819, 780, 745, 720, 624, 573 cm^–1^. Purity: UHPLC (254 nm): *t*_r_ = 4.187 min, 98.4% total area. ^1^H NMR (400 MHz, CDCl_3_) δ = 2.35 (s, 3H), 2.56 (t, *J* = 5.1 Hz, 4H), 3.75–3.81 (m, 4H), 6.85 (s, 1H), 7.09–7.12 (m, 2H), 7.32 (dd, *J* = 1.8, 8.5 Hz, 1H), 7.38–7.46 (m, 2H), 7.64–7.71 (m, 2H), 7.75 (dd, *J* = 1.9, 7.4 Hz, 1H), 7.91 (d, *J* = 1.8 Hz, 1H), 8.47–8.52 (m, 2H). ^13^C NMR (101 MHz, CDCl_3_) δ = 44.96, 46.27, 54.69, 112.55, 123.59, 126.25, 126.56, 127.04, 127.61, 127.64, 128.40, 129.17, 132.89, 133.09, 133.85, 137.74, 146.09, 150.11, 150.90, 158.99.

#### *N*,*N*-Dimethyl-6-(naphthalen-2-yl)-5-(pyridin-4-yl)­pyridazin-3-amine 7 (MW01–11–108SRM = MW108)^82^

Synthesized following *GP1* from **5a** (0.20 g, 0.63 mmol) and Me_2_NH (2.46 M in THF; 1.28 mL, 3.15 mmol) and isolated following *GP2*. Yield: 46 mg (0.14 mmol, 22%) of orange oil. ESI-HRMS: *m*/*z* = 327.16001 (MH^+^); C_21_H_19_N_4_ requires: *m*/*z* = 327.16042 (MH^+^). ν_max_ 3040, 2926, 2802, 1610, 1589, 1552, 1507, 1435, 1405, 1356, 1275, 1206, 1177, 1146, 1057, 1016, 993, 975, 957, 943, 921, 876, 836, 820, 780, 767, 755, 720, 654, 624, 593, 521 cm^–1^. Purity: UHPLC (254 nm): *t*_r_ = 4.373 min, 99.0% total area. ^1^H NMR (400 MHz, CDCl_3_) δ = 3.26 (s, 6H), 6.72 (s, 1H), 7.12–7.14 (m, 2H), 7.35 (dd, *J* = 1.8, 8.5 Hz, 1H), 7.39–7.46 (m, 2H), 7.65–7.72 (m, 2H), 7.75–7.78 (m, 1H), 7.90–7.92 (m, 1H), 8.49–8.52 (m, 2H). ^13^C NMR (101 MHz, CDCl_3_) δ = 38.19, 111.33, 123.67, 126.20, 126.45, 127.18, 127.59, 127.62, 128.40, 129.08, 132.83, 133.14, 134.12, 137.53, 146.36, 149.64, 150.09, 158.86.

#### *N* ^1^,*N* ^1^-Dimethyl-*N* ^2^-(6-(naphthalen-2-yl)-5-(pyridin-4-yl)­pyridazin-3-yl)­ethane-1,2-diamine **8**

Synthesized following *GP1* from **5a** (0.20 g, 0.63 mmol) and *N*,*N*-dimethylethylenediamine (0.34 mL, 3.15 mmol) and isolated following *GP2*. Yield: 59 mg (0.16 mmol, 25%) of orange oil. ESI-HRMS: *m*/*z* = 370.20189 (MH^+^); C_23_H_24_N_5_ requires: *m*/*z* = 370.20262 (MH^+^). ν_max_ 3262, 3052, 2945, 2861, 2821, 2774, 1608, 1590, 1555, 1499, 1473, 1406, 1352, 1245, 1159, 1130, 1042, 903, 861, 821, 780, 725, 644, 624 cm^–1^. Purity: UHPLC (254 nm): *t*_r_ = 4.107 min, 98.9% total area. ^1^H NMR (400 MHz, CDCl_3_) δ = 2.26 (s, 6H), 2.59 (t, *J* = 5.9 Hz, 2H), 3.57–3.63 (m, 2H), 5.76 (t, *J* = 4.9 Hz, 1H), 6.66 (s, 1H), 7.02–7.05 (m, 2H), 7.29 (dd, *J* = 1.8, 8.5 Hz, 1H), 7.37–7.45 (m, 2H), 7.62–7.69 (m, 2H), 7.75 (dd, *J* = 1.9, 7.5 Hz, 1H), 7.87 (d, *J* = 1.8 Hz, 1H), 8.43–8.47 (m, 2H). ^13^C NMR (101 MHz, CDCl_3_) δ = 39.15, 45.22, 57.75, 114.29, 123.50, 126.22, 126.48, 127.12, 127.58, 127.60, 128.37, 129.07, 132.83, 133.07, 134.11, 137.69, 145.78, 150.01, 151.03, 158.22.

#### *N* ^1^,*N* ^1^-Diethyl-*N* ^2^-(6-(naphthalen-2-yl)-5-(pyridin-4-yl)­pyridazin-3-yl)­ethane-1,2-diamine **9**

Synthesized following *GP1* from **5a** (0.10 g, 0.31 mmol) and *N*,*N*-diethylethylenediamine (0.26 mL, 1.86 mmol) and isolated following *GP2*. Yield: 67 mg (0.17 mmol, 55%) of yellow solid. ESI-HRMS: *m*/*z* = 398.23334 (MH^+^); C_25_H_28_N_5_ requires: *m*/*z* = 398.23392 (MH^+^). ν_max_ 3266, 3052, 2970, 2934, 2820, 1590, 1556, 1499, 1473, 1408, 1384, 1243, 1199, 1169, 1130, 1060, 895, 862, 821, 780, 748, 652, 624 cm^–1^. Purity: UHPLC (254 nm): *t*_r_ = 4.710 min, 96.8% total area. ^1^H NMR (400 MHz, CDCl_3_) δ = 1.04 (t, *J* = 7.1 Hz, 6H), 2.59 (q, *J* = 7.1 Hz, 4H), 2.75 (dd, *J* = 6.6, 5.3 Hz, 2H), 3.57 (dt, *J* = 6.4, 5.1 Hz, 2H), 5.71 (t, *J* = 5.0 Hz, 1H), 6.68 (s, 1H), 7.05–7.13 (m, 2H), 7.32 (dd, *J* = 8.5, 1.8 Hz, 1H), 7.39–7.48 (m, 2H), 7.64–7.73 (m, 2H), 7.74–7.81 (m, 1H), 7.89 (d, *J* = 1.7 Hz, 1H), 8.35–8.54 (m, 2H). ^13^C NMR (101 MHz, MeOH-*d*_4_) δ = 11.61, 39.84, 48.10, 52.50, 117.01, 125.45, 127.54, 127.77, 128.04, 128.68, 128.80, 129.21, 130.15, 134.36, 134.42, 135.17, 139.63, 147.78, 150.29, 152.20, 159.94.

#### *N* ^1^,*N* ^1^-Dimethyl-*N* ^3^-(6-(naphthalen-2-yl)-5-(pyridin-4-yl)­pyridazin-3-yl)­propane-1,3-diamine **10**

Synthesized following *GP1* from **5a** (0.15 g, 0.47 mmol) and *N*,*N*-dimethyl-1,3-propanediamine (0.59 mL, 4.70 mmol) and isolated following *GP2*. Yield: 101 mg (0.26 mmol, 55%) of yellow solid. ESI-HRMS: *m*/*z* = 384.21762 (MH^+^); C_24_H_26_N_5_ requires: *m*/*z* = 384.21827 (MH^+^). ν_max_ 3238, 3093, 2960, 2940, 2852, 2811, 2779, 2762, 1611, 1592, 1508, 1487, 1464, 1429, 1408, 1374, 1350, 1243, 1171, 1057, 903, 864, 823, 781, 755 cm^–1^. Purity: UHPLC (254 nm): *t*_r_ = 4.343 min, 97.1% total area. ^1^H NMR (400 MHz, CDCl_3_) δ = 1.87 (p, *J* = 6.4 Hz, 2H), 2.27 (s, 6H), 2.47 (t, *J* = 6.4 Hz, 2H), 3.60 (q, *J* = 6.2 Hz, 2H), 6.28 (t, *J* = 5.3 Hz, 1H), 6.64 (s, 1H), 7.07–7.14 (m, 2H), 7.33 (dd, *J* = 8.5, 1.8 Hz, 1H), 7.40–7.50 (m, 2H), 7.68 (d, *J* = 8.6 Hz, 1H), 7.69–7.73 (m, 1H), 7.75–7.83 (m, 1H), 7.90 (d, *J* = 1.7 Hz, 1H), 8.47–8.57 (m, 2H). ^13^C NMR (101 MHz, CDCl_3_) δ = 26.39, 41.83, 45.66, 58.48, 113.38, 123.66, 126.29, 126.55, 127.25, 127.68, 127.71, 128.50, 129.18, 132.94, 133.21, 134.25, 137.92, 146.08, 150.14, 150.96, 158.68.

#### *N* ^1^,*N* ^2^-Dimethyl-*N* ^1^-(6-(naphthalen-2-yl)-5-(pyridin-4-yl)­pyridazin-3-yl)­ethane-1,2-diamine **11**

*tert*-Butyl methyl­(2-(methyl­(6-(naphthalen-2-yl)-5-(pyridin-4-yl)­pyridazin-3-yl)­amino)­ethyl)­carbamate **11a** was synthesized following *GP1* from **5a** (0.10 g, 0.31 mmol) and *tert*-butyl methyl­(2-(methylamino)­ethyl)­carbamate (0.32 g, 1.86 mmol) and isolated following *GP2*. Yield: 79 mg (0.17 mmol, 55%) of yellow oil. ESI-HRMS: *m*/*z* = 470.25459 (MH^+^); C_28_H_32_N_5_O_2_ requires: *m*/*z* = 470.25505 (MH^+^). ^1^H NMR (400 MHz, CDCl_3_) δ = 1.34 and 1.35 (2 × s, 9H), 2.92 (d, *J* = 4.9 Hz, 3H), 3.21 (s, 3H), 3.47–3.62 (m, 2H), 3.81–4.00 (m, 2H), 6.76 (d, *J* = 48.6 Hz, 1H), 7.14 (d, *J* = 4.5 Hz, 2H), 7.31 (dd, *J* = 8.5, 1.8 Hz, 1H), 7.38–7.46 (m, 2H), 7.63–7.79 (m, 3H), 7.91 (d, *J* = 7.7 Hz, 1H), 8.51 (d, *J* = 5.3 Hz, 2H)–mixture of conformers. ^13^C NMR (101 MHz, CDCl_3_) δ = 28.39, 28.52, 34.91, 35.65, 36.71, 37.41, 46.33, 46.60, 48.62, 79.49, 79.68, 110.95, 111.24, 123.71, 126.23, 126.48, 127.13, 127.62, 128.40, 129.05, 132.85, 133.17, 134.16, 137.65, 137.84, 146.23, 150.10, 155.59, 155.92, 158.04, 158.55–mixture of conformers.

*tert*-Butyl methyl­(2-(methyl­(6-(naphthalen-2-yl)-5-(pyridin-4-yl)­pyridazin-3-yl)­amino)­ethyl)­carbamate **11a** (0.050 g, 0.11 mmol) in DCM (10 mL) and TFA (1 mL) was stirred at rt for 24 h, then volatiles were evaporated *in vacuo*, and **11** was isolated following *GP2*. Yield: 33 mg (0.088 mmol, 80%) of pale-yellow crystals. ESI-HRMS: *m*/*z* = 370.20206 (MH^+^); C_23_H_24_N_5_ requires: *m*/*z* = 370.20262 (MH^+^). ν_max_ 3271, 3068, 2928, 2844, 2790, 1606, 1588, 1548, 1503, 1458, 1408, 1356, 1189, 894, 865, 837, 820, 803, 779, 766, 749, 719, 648, 625 cm^–1^. Purity: UHPLC (254 nm): *t*_r_ = 4.400 min, 95.9% total area. ^1^H NMR (400 MHz, CDCl_3_) δ = 2.49 (s, 3H), 2.94 (t, *J* = 6.5 Hz, 2H), 3.25 (s, 3H), 3.90 (t, *J* = 6.5 Hz, 2H), 6.78 (s, 1H), 7.12–7.18 (m, 2H), 7.34 (dd, *J* = 8.5, 1.8 Hz, 1H), 7.39–7.49 (m, 2H), 7.66–7.74 (m, 2H), 7.74–7.81 (m, 1H), 7.93 (d, *J* = 1.7 Hz, 1H), 8.48–8.57 (m, 2H). ^13^C NMR (101 MHz, CDCl_3_) δ = 36.82, 37.02, 49.66, 50.33, 111.43, 123.73, 126.29, 126.54, 127.21, 127.67, 127.69, 128.48, 129.14, 132.93, 133.23, 134.12, 137.73, 146.39, 149.92, 150.20, 158.65.

#### *N*-Isopentyl-6-(naphthalen-2-yl)-5-(pyridin-4-yl)­pyridazin-3-amine **12**

Synthesized following *GP1* from **5a** (0.10 g, 0.31 mmol) and isopentylamine (0.22 mL, 1.86 mmol) and isolated following *GP2*. Yield: 66 mg (0.18 mmol, 25%) of yellow solid. ESI-HRMS: *m*/*z* = 369.20693 (MH^+^); C_24_H_25_N_4_ requires: *m*/*z* = 369.20737 (MH^+^). ν_max_ 3263, 3058, 2968, 2945, 2862, 2833, 1611, 1592, 1546, 1499, 1487, 1448, 1426, 1405, 1381, 1352, 1329, 1252, 1170, 1130, 1058, 903, 865, 820, 779, 749, 651, 623 cm^–1^. Purity: UHPLC (254 nm): *t*_r_ = 5.433 min, 97.7% total area. ^1^H NMR (400 MHz, MeOH-*d*_4_) δ = 0.99 (d, *J* = 6.6 Hz, 6H), 1.56–1.65 (m, 2H), 1.77 (dp, *J* = 13.3, 6.7 Hz, 1H), 3.48–3.59 (m, 2H), 6.93 (s, 1H), 7.19–7.24 (m, 2H), 7.27 (dd, *J* = 8.5, 1.8 Hz, 1H), 7.40–7.49 (m, 2H), 7.67–7.75 (m, 2H), 7.75–7.83 (m, 2H), 8.34–8.44 (m, 2H). ^13^C NMR (101 MHz, MeOH-*d*_4_) δ = 22.97, 27.06, 39.19, 40.86, 116.95, 125.42, 127.51, 127.72, 128.05, 128.66, 128.75, 129.19, 130.10, 134.32, 134.40, 135.23, 139.50, 147.85, 150.26, 151.82, 159.93.

#### *N*-Isobutyl-6-(naphthalen-2-yl)-5-(pyridin-4-yl)­pyridazin-3-amine **13**

Synthesized following *GP1* from **5a** (0.10 g, 0.31 mmol) and isobutylamine (0.18 mL, 1.86 mmol) and isolated following *GP2*. Yield: 35 mg (0.10 mmol, 33%) of orange semisolid. ESI-HRMS: *m*/*z* = 355.19111 (MH^+^); C_23_H_23_N_4_ requires: *m*/*z* = 355.19172 (MH^+^). ν_max_ 3288, 3052, 2959, 2869, 2447, 1612, 1591, 1551, 1500, 1479, 1426, 1405, 1386, 1361, 1250, 1173, 1058, 823, 749 cm^–1^. Purity: UHPLC (254 nm): *t*_r_ = 5.113 min, 95.3% total area. ^1^H NMR (400 MHz, CDCl_3_) δ = 1.02 (d, *J* = 6.7 Hz, 6H), 2.01 (dp, *J* = 13.4, 6.7 Hz, 1H), 3.30 (dd, *J* = 6.9, 5.9 Hz, 2H), 5.30 (t, *J* = 5.9 Hz, 1H), 6.64 (s, 1H), 7.04–7.13 (m, 2H), 7.31 (dd, *J* = 8.5, 1.8 Hz, 1H), 7.38–7.49 (m, 2H), 7.62–7.74 (m, 2H), 7.74–7.80 (m, 1H), 7.89 (d, *J* = 1.9 Hz, 1H), 8.47–8.52 (m, 2H). ^13^C NMR (101 MHz, MeOH-*d*_4_) δ = 20.70, 29.23, 50.21, 116.86, 125.43, 127.50, 127.72, 128.05, 128.66, 128.75, 129.18, 130.10, 134.32, 134.40, 135.24, 139.55, 147.87, 150.26, 151.80, 160.13.

#### *N*-Benzyl-6-(naphthalen-2-yl)-5-(pyridin-4-yl)­pyridazin-3-amine **14**

Synthesized following *GP1* from **5a** (0.15 g, 0.47 mmol) and benzylamine (0.26 mL, 2.37 mmol) and isolated by column chromatography on silica (DCM/MeOH = 20:1). Yield: 50 mg (0.13 mmol, 28%) of off-yellow solid. ESI-HRMS: *m*/*z* = 389.17507 (MH^+^); C_26_H_21_N_4_ requires: *m*/*z* = 389.17607 (MH^+^). ν_max_ 2766, 1777, 1672, 1638, 1581, 1499, 1403, 1359, 1322, 1267, 1132, 1047, 1028, 990, 899, 865, 824, 796, 750, 721, 701, 622, 569, 516 cm^–1^. Purity: UHPLC (254 nm): *t*_r_ = 4.920 min, 95.4% total area. ^1^H NMR (400 MHz, DMSO-*d*_6_) δ = 4.71 (d, *J* = 5.9 Hz, 2H), 6.94 (s, 1H), 7.18–7.24 (m, 2H), 7.24–7.31 (m, 2H), 7.36 (dd, *J* = 8.4, 6.8 Hz, 2H), 7.40–7.45 (m, 2H), 7.45–7.53 (m, 2H), 7.73 (t, *J* = 6.0 Hz, 1H), 7.78 (dd, *J* = 8.6, 5.5 Hz, 2H), 7.82–7.90 (m, 2H), 8.47–8.53 (m, 2H). ^13^C NMR (101 MHz, DMSO-*d*_6_/TFA-*d* = 1:1) δ = 46.00, 126.57, 127.47, 127.52, 128.18, 128.27, 128.46, 128.50, 128.78, 128.98, 129.24, 129.25, 130.37, 132.82, 133.70, 143.55, 150.40, 151.59.

#### *O*-Benzyl-*N*-(6-(naphthalen-2-yl)-5-(pyridin-4-yl)­pyridazin-3-yl)­hydroxylamine **15**

Synthesized following *GP1* from **5a** (0.25 g, 0.79 mmol) and *O*-benzylhydroxylamine hydrochloride (0.76 g, 4.74 mmol) and DIPEA (0.83 mL, 4.74 mmol), and isolated following *GP2*. Yield: 45 mg (0.11 mmol, 14%) of orange solid. ESI-HRMS: *m*/*z* = 405.17067 (MH^+^); C_26_H_21_N_4_O requires: *m*/*z* = 405.17099 (MH^+^). ν_max_ 3058, 2921, 2876, 1618, 1585, 1502, 1455, 1416, 1398, 1361, 1333, 1170, 1041, 1011, 1000, 927, 896, 862, 816, 758, 740, 727, 697, 679, 655, 635, 621, 562 cm^–1^. Purity: UHPLC (254 nm): *t*_r_ = 6.067 min, 95.4% total area. ^1^H NMR (400 MHz, CDCl_3_) δ = 5.11 (s, 2H), 6.77 (d, *J* = 2.4 Hz, 1H), 6.94–7.01 (m, 2H), 7.09–7.12 (m, 1H), 7.30–7.50 (m, 7H), 7.63–7.72 (m, 3H), 7.73–7.82 (m, 1H), 8.40–8.50 (m, 2H), 9.64–9.76 (m, 1H). ^13^C NMR (101 MHz, CDCl_3_) δ = 76.32, 122.93, 125.98, 126.64, 126.96, 127.13, 127.78, 127.92, 128.19, 128.34, 128.41, 128.62, 132.46, 132.91, 133.17, 135.19, 137.82, 142.84, 144.79, 144.99, 145.24, 150.09.

#### *N*,*O*-Dimethyl-*N*-(6-(naphthalen-2-yl)-5-(pyridin-4-yl)­pyridazin-3-yl)­hydroxylamine **16**

Synthesized following *GP1* from **5a** (0.10 g, 0.31 mmol) and *N*,*O*-dimethylhydroxylamine hydrochloride (0.18 g, 1.86 mmol) and DIPEA (0.32 mL, 1.86 mmol), and isolated following *GP2*. Yield: 16 mg (0.047 mmol, 15%) of white solid. ESI-HRMS: *m*/*z* = 343.15489 (MH^+^); C_21_H_19_N_4_O requires: *m*/*z* = 343.15534 (MH^+^). ν_max_ 3053, 2927, 2852, 1583, 1551, 1499, 1460, 1438, 1410, 1385, 1269, 1160, 1131, 1110, 1061, 1022, 1014, 896, 861, 820, 783, 748, 732, 718, 678, 656, 624, 559 cm^–1^. Purity: UHPLC (254 nm): *t*_r_ = 5.203 min, 98.6% total area. ^1^H NMR (400 MHz, CDCl_3_) δ = 3.54 (s, 3H), 3.86 (s, 3H), 7.14–7.21 (m, 2H), 7.30 (s, 1H), 7.35 (dd, *J* = 8.5, 1.8 Hz, 1H), 7.42–7.54 (m, 2H), 7.69–7.76 (m, 2H), 7.78–7.83 (m, 1H), 7.95–7.99 (m, 1H), 8.52–8.58 (m, 2H). ^13^C NMR (101 MHz, MeOH-*d*_4_) δ = 38.47, 61.76, 116.82, 125.64, 127.69, 127.96, 128.05, 128.73, 128.98, 129.30, 130.62, 134.41, 134.58, 134.71, 140.46, 147.57, 150.41, 155.20, 164.52.

#### 6-Hydrazineyl-3-(naphthalen-2-yl)-4-(pyridin-4-yl)­pyridazine **17**

Synthesized following *GP1* from **5a** (0.10 g, 0.31 mmol) and hydrazine hydrate (0.09 mL, 1.86 mmol), and isolated following *GP2*. Yield: 35 mg (0.11 mmol, 36%) of orange solid. ESI-HRMS: *m*/*z* = 314.13959 (MH^+^); C_19_H_16_N_5_ requires: *m*/*z* = 314.14002 (MH^+^). ν_max_ 3745, 3587, 3244, 3026, 1589, 1545, 1475, 1442, 1405, 1247, 1156, 1129, 1068, 897, 866, 825, 781, 743, 651, 624, 611, 551 cm^–1^. Purity: UHPLC (254 nm): *t*_r_ = 3.977 min, 96.3% total area. ^1^H NMR (400 MHz, DMSO-*d*_*6*_) δ = 4.46 (s, 2H), 7.10 (s, 1H), 7.20–7.26 (m, 2H), 7.30 (dd, *J* = 8.4, 1.8 Hz, 1H), 7.46–7.54 (m, 2H), 7.80 (t, *J* = 8.7 Hz, 2H), 7.85–7.90 (m, 2H), 8.34 (s, 1H), 8.49–8.54 (m, 2H). ^13^C NMR (101 MHz, DMSO-*d*_*6*_) δ = 111.94, 123.54, 126.35, 126.43, 127.19, 127.24, 127.50, 128.05, 128.39, 132.18, 132.56, 134.66, 137.25, 145.52, 149.78, 150.40, 162.01.

#### 6-(2-Methylhydrazineyl)-3-(naphthalen-2-yl)-4-(pyridin-4-yl)­pyridazine **18**

*Tert*-butyl 1-methyl-2-(6-(naphthalen-2-yl)-5-(pyridin-4-yl)­pyridazin-3-yl)­hydrazine-1-carboxylate **18a** was synthesized following *GP1* from **5a** (0.25 g, 0.79 mmol) and *tert*-butyl 1-methylhydrazine-1-carboxylate (0.56 mL, 3.80 mmol), and isolated by column chromatography on silica (1. EtOAc/*n*-hex = 4:1; 2. EtOAc). Yield: 138 mg (0.17 mmol, 41%) of orange solid. ESI-HRMS: *m*/*z* = 428.20765 (MH^+^); C_25_H_26_N_5_O_2_ requires: *m*/*z* = 428.20810 (MH^+^). ^1^H NMR (400 MHz, CDCl_3_) δ = 1.45 (s, 9H), 3.32 (s, 3H), 6.88 (s, 1H), 7.05–7.11 (m, 2H), 7.30 (dd, *J* = 8.6, 1.8 Hz, 1H), 7.40–7.54 (m, 2H), 7.60–7.75 (m, 3H), 7.75–7.81 (m, 1H), 7.93 (d, *J* = 1.7 Hz, 1H), 8.48–8.54 (m, 2H). ^13^C NMR (101 MHz, CDCl_3_) δ = 28.37, 38.39, 82.04, 112.58, 123.60, 126.45, 126.85, 127.09, 127.73, 127.86, 128.54, 129.57, 133.11, 133.14, 133.64, 138.80, 145.47, 150.24, 154.03, 156.19, 160.00.

*tert*-Butyl 1-methyl-2-(6-(naphthalen-2-yl)-5-(pyridin-4-yl)­pyridazin-3-yl)­hydrazine-1-carboxylate **18a** (0.10 g, 0.24 mmol) in DCM (10 mL) and TFA (1 mL) was stirred at rt for 24 h, then volatiles were evaporated *in vacuo*, and **18** was isolated following *GP2*. Yield: 65 mg (0.20 mmol, 85%) of orange crystals. ESI-HRMS: *m*/*z* = 328.15520 (MH^+^); C_20_H_18_N_5_ requires: *m*/*z* = 328.15567 (MH^+^). ν_max_ 3237, 3058, 2960, 1589, 1550, 1475, 1437, 1406, 1359, 1158, 1131, 1063, 903, 865, 822, 781, 726, 644, 623, 609, 552 cm^–1^. Purity: UHPLC (254 nm): *t*_r_ = 4.210 min, 96.2% total area. ^1^H NMR (400 MHz, CDCl_3_) δ = 2.77 (s, 3H), 7.10–7.16 (m, 2H), 7.26 (d, *J* = 1.4 Hz, 1H), 7.32 (dd, *J* = 8.5, 1.8 Hz, 1H), 7.42–7.50 (m, 2H), 7.65–7.75 (m, 2H), 7.75–7.82 (m, 1H), 7.92 (d, *J* = 1.8 Hz, 1H), 8.47–8.53 (m, 2H). ^13^C NMR (101 MHz, CDCl_3_) δ = 39.56, 113.30, 123.69, 126.38, 126.68, 127.18, 127.71, 127.79, 128.47, 129.28, 132.99, 133.17, 134.10, 138.66, 145.87, 150.11, 152.72, 161.24.

#### 3-(Naphthalen-2-yl)-4-(pyridin-4-yl)-6-vinylpyridazine **19**

6-Chloro-3-(naphthalen-2-yl)-4-(pyridin-4-yl)­pyridazine **5a** (0.60 g, 1.89 mmol), potassium vinyltrifluoroborate (0.30 g, 2.27 mmol), triphenylphosphine (0.05 g, 0.19 mmol) and Cs_2_CO_3_ (1.85 g, 5.68 mmol) in water (5 mL) and THF (20 mL) were bubbled under a stream of argon for 15 min, then palladium­(II) chloride (0.017 g, 0.095 mmol) was added and the mixture stirred at 80 °C for 24 h in a glass pressure tube. The volatiles were evaporated *in vacuo*, the residue partitioned between EtOAc (3 × 50 mL) and water (50 mL), the organic phase separated, dried over anhydrous sodium sulfate, filtered, volatiles evaporated *in vacuo*, and **19** isolated by column chromatography on silica (1. EtOAc/*n*-hex = 1:1; 2. EtOAc/*n*-hex = 1:4). Yield: 465 mg (1.50 mmol, 79%) of yellow solid. ESI-HRMS: *m*/*z* = 310.13362 (MH^+^); C_21_H_16_N_3_ requires: *m*/*z* = 310.13387 (MH^+^). ν_max_ 3025, 2919, 1707, 1632, 1598, 1567, 1547, 1500, 1466, 1411, 1399, 1354, 1321, 1267, 1214, 1197, 1147, 1130, 1066, 1037, 1015, 993, 972, 951, 926, 893, 860, 847, 820, 798, 767, 753, 747, 731, 719, 655, 628, 616, 607, 550, 507 cm^–1^. Purity: UHPLC (254 nm): *t*_r_ = 5.050 min, 98.1% total area. ^1^H NMR (400 MHz, CDCl_3_) δ = 5.78 (d, *J* = 11.0 Hz, 1H), 6.41 (d, *J* = 17.7 Hz, 1H), 7.10–7.22 (m, 3H), 7.39 (dd, *J* = 8.5, 1.8 Hz, 1H), 7.43–7.55 (m, 2H), 7.58 (s, 1H), 7.70–7.79 (m, 2H), 7.82 (dd, *J* = 7.8, 1.6 Hz, 1H), 8.06 (d, *J* = 1.7 Hz, 1H), 8.54–8.60 (m, 2H). ^13^C NMR (101 MHz, CDCl_3_) δ = 121.41, 123.64, 124.18, 126.64, 126.98, 127.27, 127.79, 128.10, 128.75, 130.25, 133.13, 133.33, 133.46, 134.03, 136.69, 145.27, 150.43, 156.83, 157.87.

#### *N*,*N*-Dimethyl-2-(6-(naphthalen-2-yl)-5-(pyridin-4-yl)­pyridazin-3-yl)­ethan-1-amine **20**

3-(Naphthalen-2-yl)-4-(pyridin-4-yl)-6-vinylpyridazine **19** (0.10 g, 0.32 mmol) and Me_2_NH (2.46 M in THF; 1.65 mL, 1.60 mmol) in THF (8 mL) were stirred at 75 °C for 24 h in a glass pressure tube. The volatiles were evaporated *in vacuo*, the residue partitioned between DCM (50 mL) and water (50 mL), the organic phase separated, dried over anhydrous sodium sulfate, filtered, volatiles evaporated *in vacuo*, and **20** isolated following *GP2*. Yield: 17 mg (0.048 mmol, 15%) of beige solid. ESI-HRMS: *m*/*z* = 355.19126 (MH^+^); C_23_H_23_N_4_ requires: *m*/*z* = 355.19172 (MH^+^). ν_max_ 3036, 2939, 2855, 2810, 2766, 1580, 1545, 1507, 1467, 1402, 1362, 1321, 1266, 1218, 1199, 1133, 1108, 1066, 1049, 1021, 990, 971, 958, 934, 899, 866, 846, 826, 795, 778, 754, 725, 691 665, 658, 626, 567, 556, 513 cm^–1^. Purity: UHPLC (254 nm): *t*_r_ = 4.227 min, 98.8% total area. ^1^H NMR (400 MHz, CDCl_3_) δ = 2.34 (s, 6H), 2.87 (t, *J* = 7.4 Hz, 2H), 3.28 (t, *J* = 7.4 Hz, 2H), 7.13–7.17 (m, 2H), 7.37 (dd, *J* = 1.8, 8.5 Hz, 1H), 7.42 (s, 1H), 7.45–7.54 (m, 2H), 7.73 (d, *J* = 8.5 Hz, 1H), 7.74–7.79 (m, 1H), 7.79–7.84 (m, 1H), 8.02–8.05 (m, 1H), 8.50–8.57 (m, 2H). ^13^C NMR (101 MHz, CDCl_3_) δ = 34.14, 45.56, 58.93, 123.72, 126.57, 127.07, 127.14, 127.27, 127.77, 128.03, 128.69, 130.14, 133.14, 133.37, 133.52, 136.48, 145.31, 150.32, 157.45, 161.09.

#### *N* ^1^,*N* ^1^-Dimethyl-*N* ^2^-(6-(naphthalen-1-yl)-5-(pyridin-4-yl)­pyridazin-3-yl)­ethane-1,2-diamine **21**

Synthesized following *GP1* from **5b** (0.09 g, 0.28 mmol) and *N*,*N*-dimethylethylenediamine (0.30 mL, 2.80 mmol) and isolated following *GP2*. Yield: 63 mg (0.17 mmol, 62%) of orange oil. ESI-HRMS: *m*/*z* = 370.20211 (MH^+^); C_23_H_24_N_5_ requires: *m*/*z* = 370.20262 (MH^+^). ν_max_ 3240, 3046, 1590, 1556, 1501, 1476, 1408, 1395, 1240, 1161, 1046, 868, 826, 806, 782, 623, 589, 549 cm^–1^. Purity: UHPLC (254 nm): *t*_r_ = 3.717 min, 96.4% total area. ^1^H NMR (400 MHz, CDCl_3_) δ = 2.32 (s, 6H), 2.61–2.68 (m, 2H), 3.64 (q, *J* = 5.4 Hz, 2H), 5.60 (t, *J* = 5.0 Hz, 1H), 6.76 (s, 1H), 6.91–6.96 (m, 2H), 7.28 (d, *J* = 1.3 Hz, 1H), 7.31–7.46 (m, 3H), 7.66 (d, *J* = 8.4 Hz, 1H), 7.82 (d, *J* = 8.2 Hz, 2H), 8.30–8.35 (m, 2H). ^13^C NMR (101 MHz, CDCl_3_) δ = 39.20, 45.26, 57.81, 113.47, 122.98, 125.09, 125.58, 126.00, 126.42, 128.35, 128.68, 128.94, 132.11, 133.67, 134.48, 139.16, 145.31, 149.68, 151.02, 158.67.

#### *N*,*N*-Dimethyl-6-(naphthalen-1-yl)-5-(pyridin-4-yl)­pyridazin-3-amine 22 (MW01–10–181SRM = MW181)^82^

Synthesized following *GP1* from **5b** (0.09 g, 0.28 mmol) and Me_2_NH (33% in EtOH; 0.50 mL, 2.80 mmol) and isolated following *GP2*. Yield: 57 mg (0.17 mmol, 61%) of beige solid. ESI-HRMS: *m*/*z* = 327.16001 (MH^+^); C_21_H_19_N_4_ requires: *m*/*z* = 327.16042 (MH^+^). ν_max_ 3048, 2916, 2853, 1587, 1555, 1508, 1486, 1423, 1398, 1205, 1192, 1178, 1157, 828, 802, 779, 771 cm^–1^. Purity: UHPLC (254 nm): *t*_r_ = 4.380 min, 96.8% total area. ^1^H NMR (400 MHz, Acetone-*d*_*6*_) δ = 3.32 (s, 6H), 7.09–7.14 (m, 2H), 7.15 (s, 1H), 7.37 (td, *J* = 6.8, 1.4 Hz, 2H), 7.44 (ddd, *J* = 8.2, 6.9, 1.3 Hz, 2H), 7.61–7.66 (m, 1H), 7.89 (dt, *J* = 8.3, 1.0 Hz, 2H), 8.19–8.33 (m, 2H). ^13^C NMR (101 MHz, Acetone-*d*_*6*_) δ = 38.17, 111.42, 124.07, 125.89, 126.56, 126.66, 126.91, 129.05, 129.22, 129.46, 133.14, 134.51, 136.46, 139.43, 146.49, 150.14, 150.27, 160.45.

#### 4-Bromo-1,2-dihydropyridazine-3,6-dione **23**

To a heated solution (100 °C) of hydrazine sulfate (2.30 g, 17.66 mmol) in water (10 mL), bromomaleic anhydride (2.50 g, 14.13 mmol) was added dropwise over 15 min, and the mixture was stirred at 100 °C for 24 h. The mixture was cooled to room temperature, the precipitate collected by filtration, washed with acetone, and dried. Yield: 2672 mg (13.99 mmol, 99%) of white solid. ESI-HRMS: *m*/*z* = 190.94501 (MH^+^); C_4_H_4_BrN_2_O_2_ requires: *m*/*z* = 190.94507 (MH^+^). ^1^H NMR (500 MHz, DMSO-*d*_6_) δ = 7.59 (br s, 1H), 11.14 (br s, 1H), 12.35 (br s, 1H).^13^C NMR (126 MHz, MeOH-*d*_4_) δ = 129.85, 132.50, 157.11, 157.35.

#### 3,6-Dichloro-4-(pyridin-4-yl)­pyridazine **24**

4-Bromo-1,2-dihydropyridazine-3,6-dione **23** (2.50 g, 13.09 mmol) and 4-pyridineboronic acid pinacol ester (3.68 g, 17.93 mmol) were dissolved in DME/water (10:1, 63 mL) in a pressure tube, degassed for 30 min, then Pd­(PPh_3_)_4_ (1.51 g, 1.31 mmol) and Na_2_CO_3_ (4.16 g, 39.27 mmol,) were added and the reaction stirred at 110 °C for 24 h. Then, the reaction mixture was cooled, filtered through a pad of Celite, washed with MeOH, and the filtrate evaporated *in vacuo*. Crude 4-(pyridin-4-yl)-1,2-dihydropyridazine-3,6-dione (2.48 g, 13.09 mmol) was dissolved in MeCN (8 mL), cooled to 0 °C, POCl_3_ (6.85 mL, 73.30 mmol) added, and the resulting mixture stirred at 90 °C for 24 h. The volatiles were evaporated *in vacuo*, and the crude mixture was poured onto ice, stirred for 2 h, then neutralized to a pH of ∼7 using saturated aqueous Na_2_CO_3_ solution. The organics were extracted with DCM (3 × 40 mL). The combined organic phase was dried over anhydrous sodium sulfate, filtered, volatiles evaporated *in vacuo*, and **24** isolated by column chromatography on silica (1. EtOAc/*n*-hex = 1:10; 2. EtOAc/*n*-hex = 1:1; 3. EtOAc/*n*-hex = 3:1). Yield: 384 mg (1.70 mmol, 13%) of yellow solid. ESI-HRMS: *m*/*z* = 225.99333 (MH^+^); C_9_H_6_Cl_2_N_3_ requires: *m*/*z* = 225.99333 (MH^+^). ^1^H NMR (500 MHz, CDCl_3_) δ = 7.42 (dd, *J* = 4.5, 1.5 Hz, 2H), 7.51 (s, 1H), 8.82 (dd, *J* = 4.5, 1.6 Hz, 2H). ^13^C NMR (126 MHz, CDCl_3_) δ = 123.18, 129.52, 140.18, 141.01, 150.67, 154.11, 156.39.

#### *N* ^1^-(6-Chloro-5-(pyridin-4-yl)­pyridazin-3-yl)-*N* ^2^,*N* ^2^-dimethylethane-1,2-diamine **25**

3,6-Dichloro-4-(pyridin-4-yl)­pyridazine **24** (1.75 g, 7.74 mmol) and *N*,*N*-dimethylethylenediamine (3.38 mL, 30.97 mmol) were dissolved in EtOH (60 mL) in a pressure tube and the reaction was stirred at 90 °C for 24 h. Then, volatiles were removed *in vacuo*, and the residue was partitioned between water (50 mL) and DCM (3 × 80 mL). The combined organic phase was dried over anhydrous sodium sulfate, filtered, and volatiles evaporated *in vacuo*. Yield: 2128 mg (7.66 mmol, 99%) of yellow solid. ESI-HRMS: *m*/*z* = 278.11652 (MH^+^); C_13_H_17_ClN_5_ requires: *m*/*z* = 278.11670 (MH^+^). ^1^H NMR (500 MHz, CDCl_3_) δ = 2.42 (s, 6H), 2.80–2.83 (m, 2H), 3.66–3.69 (m, 2H), 6.51–6.56 (m, 1H), 6.68 (s, 1H), 7.36 (dd, *J* = 4.5, 1.6 Hz, 2H), 8.72 (dd, *J* = 4.4, 1.6 Hz, 2H). ^13^C NMR (126 MHz, CDCl_3_) δ = 38.18, 44.35, 57.13, 116.71, 123.33, 138.27, 143.02, 144.37, 149.99, 158.35.

#### *N* ^1^,*N* ^1^-Dimethyl-*N* ^2^-(6-(naphthalen-1-yl)-5-(pyridin-4-yl)­pyridazin-3-yl)­ethane-1,2-diamine 21 (from pyridazine derivative **25**)

Pyridazine **25** (1.00 g, 3.60 mmol) and 1-naphthaleneboronic acid (0.85 g, 4.93 mmol) were dissolved in DME/water (10:1, 37 mL) in a pressure tube, degassed for 30 min, then Pd­(PPh_3_)_4_ (0.42 g, 0.36 mmol) and Na_2_CO_3_ (1.14 g, 10.80 mmol,) were added and the reaction stirred at 110 °C for 24 h. Then, the reaction mixture was cooled, filtered through a pad of Celite, washed with MeOH and the filtrate evaporated *in vacuo*. Product **21** was isolated by reversed-phase column chromatography on a C18 column (gradient 0.05% AcOH_(aq)_/MeCN). Yield: 680 mg (1.84 mmol, 51%) of yellow solid. The analytical data correspond to the data of the final product **21**, synthesized according to the general synthetic approach (Scheme 1). Purity: UHPLC (280 nm, Method B): >99% total area. The tested compound was dissolved in 50% methanol at a concentration of 0.5 mg/mL. Noncalibrated purity was determined by UHPLC analysis using an Agilent 1290 Infinity II system equipped with a DAD detector (Agilent Technologies, USA). Chromatographic separation was achieved using an Astra C18 column (50 × 2.1 mm^2^, 2.0 μm) (Chromservis, Czech Republic). The mobile phase consisted of (A) 5 mM ammonium formate (pH 3.2), and (B) a 1:1 mixture of MeCN and MeOH containing 0.1% formic acid. An injection volume of 0.5 μL was used.

### Molecular Modeling

Computational studies were performed on workstations at the Faculty of Pharmacy, University of Ljubljana, using Schrödinger Small Molecule Discovery Suite Release 2021–1 (Schrödinger, LLC, New York, USA, 2021). hBChE (PDB code 6QAA) crystal structure was preprocessed using the Protein Preparation Wizard: missing hydrogens were added, bond orders were assigned using the CCD database, the missing side chains and loops were modeled with Prime, whereas the het protonation states (pH 7.0 ± 2.0) were modeled with Epik.^83^ All hets, cosolvent molecules, and waters were removed. Hydrogen bonds were automatically assigned and optimized using PROPKA (pH 7.0). Receptor grids were generated (van der Waals radii scaling: 1, partial charge cutoff: 0.25). The active site was defined as the centroid of the cocrystallized ligand (inner box: 10 Å^3^).

### Inhibition of Cholinesterases

The inhibitory potencies of the compounds against the ChEs (*i*.*e*., hAChE or hBChE) were determined using the method of Ellman following the procedure described previously.^84^ For screening, 10 mM compound stock solutions in DMSO were used, and then incubated at 100 μM with Ellman’s reagent (5,5-dithio-bis-(2-nitrobenzoic acid–DTNB), final concentration: 370 μM) and the ChEs (final concentration: approximately 50–100 pM or 1 nM for hAChE or hBChE, respectively) in 0.1 M sodium phosphate (pH = 8.0) for 5 min at 20–25 °C. The reactions were started by the addition of the substrate (final concentration, 500 μM butyrylthiocholine iodide (BTCI) or acetylthiocholine iodide (ATCI) for hBChE and hAChE, respectively). The final DMSO concentration was always 1% (v/v). The increase in the absorbance at 412 nm was monitored for 2 min using a Synergy HT microplate reader (BioTek Instruments, Charlotte, VT, USA). The initial velocities in the presence (*v*_*i*_) and absence (*v*_*o*_) of the test compounds were calculated, and the inhibitory potencies expressed as the residual activities—RA [%] = (*v*_*i*_ – *b*)/ (*v*_*o*_ – *b*) × 100%, where *b* represents the blank (without the enzyme). To determine IC_50_ values, at least seven different concentrations for each compound were used. The IC_50_ values were obtained by plotting the residual ChE activity against the applied inhibitor log_10_ concentrations, and the experimental data were fitted to a four-parameter logistic function using GraphPad Prism 10.4 (GraphPad Software, CA, USA). Donepezil and tacrine were used as positive controls.

### Inhibition of p38α MAPK

The inhibitory potency against human p38α MAPK was assessed using the commercially available ADP-Glo Kinase Assay (Promega, Inc., Madison, WI, USA), following the manufacturer’s instructions. The kit components included ADP-Glo reagent, Kinase Detection Buffer, Kinase Detection Substrate, Ultra-Pure ATP (10 mM), and ADP (10 mM). Assays were conducted in white 384-well microtiter plates (ref: 784075, Greiner Bio-One GmbH, Austria) using Synergy HT (BioTek Instruments, Charlotte, VT, USA) or Tecan Spark (Tecan Group Ltd. Männedorf, Zürich, CH) plate readers. For a reference control, 1% (v/v) DMSO was used in place of the test compound. The enzyme solution was prepared by diluting the stock solution of human recombinant p38α MAPK (expressed in Sf9, an insect cell line; stock concentration: 0.1 μg/μL; SignalChem Biotech Inc., Richmond, BC, Canada) in 1× Kinase Buffer (pH = 7.5). This buffer was prepared using Milli-Q water and contained 40 mM Tris–HCl, 20 mM MgCl_2_, 0.1% BSA and 50 μM DTT (pH = 7.5). The substrate/ATP mix was prepared by diluting the stock solution of peptide substrate IPTTPITTTYFFFKKK (1 mg/mL) and ATP (1 mM) in 1× Kinase Buffer to reach final concentrations of 0.2 μg/μL and 100 μM, respectively, in each well. The enzymatic reaction was initiated by adding the enzyme solution. The final DMSO concentration in all wells was 1% (v/v). Plates were incubated for 60 min at room temperature (20–25 °C), followed by the addition of 5 μL of ADP-Glo reagent and a further 40-min incubation. Subsequently, 10 μL of Kinase Detection Substrate was added, and the plates were incubated for an additional 40 min. Luminescence was then measured at 570 nm using a Synergy HT microtiter plate reader (BioTek Instruments, Charlotte, VT, USA) with the following measurement parameters: measurement from top, gain of 150, and an integration time of 1 s. The RA and IC_50_ values were calculated as described in the Inhibition of Cholinesterases section.

### hBChE Crystallography

Crystals of hBChE in complex with **8** and **21** were obtained by soaking with ligand solutions in the crystallization mother liquor. The recombinant protein was produced in Chinese hamster ovary cells as reported previously,^85^ and purified using BChE-specific affinity chromatography (Hupresin, CHEMFORASE, Rouen, France)^86,87^ and size exclusion chromatography (Superdex 200, Cytiva). Crystals were obtained by vapor diffusion using the hanging drop method at 293 K using a 10 mg/mL hBChE and 0.1 M MES pH 6.5, 2.15 M (NH_4_)_2_SO_4_ as crystallization buffer. Stock solutions of **8** and **21** were prepared at 0.1 M in 100% MeOH, and soaks were performed at 1 mM final ligand concentration (1% MeOH final concentration). Crystals were cryoprotected using a solution of 0.1 M MES pH 6.5, 2.15 M (NH_4_)_2_SO_4_, 20% glycerol, and 1 mM ligand, 1% MeOH before flash cooling into liquid nitrogen.

X-ray diffraction data were collected at the Proxima-2A beamline of the SOLEIL synchrotron (Saint Aubin, France) and the ID30B beamline of the European Synchrotron Research Facility (Grenoble, France) for **8** and **21**-soaked crystals, respectively. Data sets of **8** and **21** were processed using the XDSME^88^ and autoPROC^89^ pipelines, respectively, based on the XDS^90^ and AIMLESS/POINTLESS software for indexing and scaling. Data analysis was done with the Phenix software suite.^91^ Initial models were obtained after molecular replacement (Phaser-MR) using the hBChE structure (PDB: 1P0I), and final structures were obtained through multiple cycles of model construction in the *Coot* software^92^ and refinement (Phenix.refine). Ligand geometry restraints were processed with Phenix eLBOW using the semiempirical quantum mechanical method (AM1).^93^ Structures of hBChE in complex with **8** and **21** were deposited into the Protein Data Bank under accession numbers 9GCQ and 9GCR, respectively (Table S2). Figures were prepared in PyMOL.

### p38α MAPK Crystallography

The human p38α MAPK used in these studies was produced in *Escherichia coli* and purified. Briefly, bacterial cultures were grown in Terrific Broth medium (BD Difco) at 30 °C. Protein expression was induced at OD_600_ = 1.4 by the addition of 1 mM isopropyl-β-d-1-thiogalactopyranoside (Research Products International), and incubation continued at 22 °C for 16 h. Cells were harvested by centrifugation and resuspended at 4 °C in a lysis buffer composed of 10 mM Tris–HCl (pH 8.3), 1 M NaCl, 1 mM MgCl_2_, 10% glycerol, 0.01% IGEPAL CA-630 (Sigma), 1 mM *tris*(2-carboxyethyl)­phosphine (TCEP), and the Pierce EDTA-free protease inhibitor tablet (Thermo Scientific). Resuspended pellets were placed at −30 °C until purification. Frozen cell suspensions were thawed under cold running water, sonicated using a Branson SFX250 sonicator with 1/2″ disruptor horn for 15 min (5 s “on”, 10 s “off”), 42% amplitude, 4 °C, and the resultant lysate clarified by centrifugation at 39,000*g* for 45 min, 4 °C. The supernatant was collected and subjected to Ni-NTA-affinity column chromatography on a HisTrap FF (5 mL) column equilibrated with loading buffer (20 mM Tris-HCl (pH 8.3), 1 M NaCl, 10 mM imidazole (pH 8.3), 2 mM TCEP), washed with 5 CV of loading buffer and 10 CV of loading buffer containing 20 mM imidazole. The protein was collected *via* step-elution with a loading buffer containing 500 mM Imidazole. Collected protein was dialyzed *vs* low salt buffer (20 mM Tris–HCl (pH 8.3), 50 mM NaCl, 2 mM TCEP) using D-Tube Dialyzer Mega, MWCO 6–8 kDa (Millipore) and was subjected to ion-exchange chromatography on HiTrap Q HP (5 mL) column (GE Healthcare) using the 30 CV gradient 0.050–1 M NaCl in buffer containing 20 mM Tris-HCl (pH 8.3) and 2 mM TCEP. Multiple fractions showing absorption at A_280_ were SDS-PAGE verified, and the ones containing the p38α MAPK were combined and dialyzed using D-Tube Dialyzer Mega, MWCO 6–8 kDa (Millipore) *vs* crystallization buffer composed of 10 mM Tris–HCl (pH 8.3), 150 mM NaCl, 1 mM TCEP. The equilibrated protein sample was concentrated to 20 mg/mL *via* multiple 20 min centrifugations at 5000*g*, 4 °C in Vivaspin 15R, 10 kDa MWCO (Sartorius) concentrator.

Protein samples for crystallization were prepared immediately before use by the addition of 4 mM 4-[3-(4-fluorophenyl)-1*H*-pyrazol-4-yl] pyridine (4-FPP) (ZW-4–252A) in 8% (v/v) DMSO (Sigma) and kept on ice bath for 45 min. Crystals of human p38α MAPK were obtained using the sitting drop vapor diffusion method in CombiClover crystallization plates (Rigaku). Drop solutions of protein (5 μL) were composed of 3 μL protein solution/ 2 μL reservoir solution. The reservoir solution was composed of 15–20% (w/V) PEG10,000, containing 100 mM ammonium acetate and 100 mM Bis-Tris (pH 5.5) at 20 °C. Protein/ligand complexes were generated using the crystal soak approach. Solutions of ligands **8** and **21**, 50 mM in neat HPLC-grade MeOH, were added to a final concentration of 2.5 mM to the protein crystals in the reservoir solution with 20% (w/v) PEG10,000 at 20 °C. Selected crystals were flash-cooled directly in liquid nitrogen from the soaking solution and then taken for data collection. Diffraction data were collected on beamline ID30B at the European Synchrotron Radiation Facility at 100 K from orthorhombic crystals using an Eiger2 9 M detector.^94^ All data were processed in the XDS software package^90^ *via* autoPROC.^89^ Initial data fitting utilized the DIMPLE pipeline within the CCP4 program suite (CCP4, 1994) and a ligand- and water-free human p38α MAPK model (PDB: 4EWQ).^15^ Ligands were identified and placed into sigma-A weighted Fo–Fc maps with Coot^92^ using real-space refinement. Ligand restraint dictionaries were generated with the grade2 server. Iterative rounds of refinement and model building were performed with phenix.refine^95^ and Coot. Polder maps^96^ were created for every ligand to ensure the correct placement of its atoms. The model quality was analyzed by MolProbity^97^ within Phenix. The coordinates and structure factors have been deposited in the Protein Data Bank with accession codes 9D8G and 9D6P for **8** and **21**, respectively (Table S3).

### Kinase Selectivity Profiling

The kinase inhibition profile of compounds **8** and **21** was assessed against 21 protein kinases (ABL1, ABL2, AKT1, CDK1/CycA2, CK1δ, ERK1, ERK2, FAK, FGR, GSK3β, JNK3, LCK, MAPKAPK5, NLK, p38α MAPK, p38β MAPK, p38γ MAPK, p38δ MAPK, PDGFRβ, PIM2, and RET) by Reaction Biology Europe GmbH (RBE) using the ^33^PanQuinase Activity Assay. In the assay, radioactive ^33^P is transferred from ATP to the kinase substrate, with the degree of enzyme inhibition depending on the occupancy of the ATP-binding site.^98^ The phosphorylated substrate is then detected using a microplate scintillation counter, where the level of scintillation is inversely correlated with kinase inhibition. Measurements were performed using 96-well ScintiPlate microtiter plates (Perkin-Elmer, USA). For the reference sample, 1% DMSO replaced the inhibitor’s solution. The inhibitor potency was expressed as the residual activity (RA) of the enzyme at two inhibitor concentrations, namely 10 μM and 1 μM, in a single replicate. Staurosporine, a competitive nonselective protein kinase inhibitor, was used as a positive control. All protein kinases provided by RBE were expressed in Sf9 insect cells or in *E. coli* as recombinant GST-fusion or His-tagged proteins, either as full-length or enzymatically active fragments. The kinases were produced from human cDNAs and purified *via* affinity chromatography using either GSH-agarose or immobilized metal. Affinity tags were removed from selected kinases during the purification process. Kinases from external vendors, including CAR = Carna Biosciences Inc. INV = Life Technologies (Invitrogen Corporation), and MIL = Merck-Millipore (Millipore Corporation) were expressed, purified, and quality-controlled according to vendor specifications. The assay mixture for all protein kinases contained 70 mM HEPES pH 7.5, 3 mM MgCl_2_, 3 mM MnCl_2_, 3 μM Na-orthovanadate, 1.2 mM DTT, 50 μg/mL PEG20000, ATP (variable concentrations, corresponding to the apparent ATP-*K*_*m*_) of the respective kinase, [γ-^33^P]-ATP (approximately 8 × 10^5^ counts per minute (*cpm*) per well), protein kinase, and substrate (variable amounts; see Table S5). The final reaction volume in each well was 50 μL with the reaction cocktail pipetted in 4 steps in the following order:

- 10 μL of nonradioactive ATP solution (in H_2_O)
- 25 μL of assay buffer/ [γ-^33^P]-ATP mixture
- 5 μL of test compound in 10% DMSO
- 10 μL of enzyme/substrate mixture

The reaction cocktails were incubated at 30 °C for 60 min. The reaction was stopped with 50 μL of 2% [v/v] H_3_PO_4_, plates were aspirated and washed twice with 200 μL of 0.9% [w/v] NaCl. Incorporation of ^33^P (counting of “*cpm*”) was determined with a microplate scintillation counter (Microbeta, Wallac).

For each kinase, the median value of *cpm* from three wells containing complete reaction cocktails but without kinase was defined as “low control” (*n* = 3). This value represents the nonspecific binding of radioactivity to the plate in the absence of protein kinase but in the presence of the substrate. Similarly, for each kinase, the median value of *cpm* from three additional wells containing the complete reaction cocktail but without any test compound was designated as the “high control” (*n* = 3), representing full enzymatic activity in the absence of an inhibitor. The difference between high and low control was considered 100% activity for each kinase. The RA, expressed as a percentage, for each compound-containing well was calculated by using the following formula

RA=100%×[(cpmofcompound−lowcontrol)/(highcontrol−lowcontrol)]

### Log *D*_7.4_ Determination

Calibration curves for phosphate-buffered saline (PBS) (pH 7.4) and *n*-octanol were prepared (0.05–5 μM). The phase ratios of *n*-octanol to buffer for LogD_7.4_ determination were 1:1 (0.8 mL *n*-octanol: 0.8 mL PBS) and 1:3 (0.4 mL *n*-octanol: 1.2 mL PBS). *n*-Octanol was pipetted into an HPLC vial, followed by the addition of 1.6 μL of a 10 mM stock DMSO solution and the PBS buffer. The vial was mixed on a vortex shaker and shaken on an orbital shaker for 3 h in the dark at 23 °C. Subsequently, the aqueous and organic phases were sampled with a syringe into a clean HPLC vial, and the samples were analyzed using UHPLC/DAD.

### BBB-PAMPA Assay

The parallel artificial membrane permeability assay (PAMPA)^54,99^ was performed using a coated 96-well membrane filter. The filter membrane of the donor plate was coated with PBL (Polar Brain Lipid, Avanti, USA) in dodecane (4 μL of 20 mg/mL PBL in dodecane) and the acceptor well was filled with 300 μL of PBS (pH 7.4; *V*_*A*_). The tested compounds were first dissolved in DMSO and then diluted with PBS (pH 7.4) to a final concentration of 100 μM in the donor wells. The final concentration of DMSO did not exceed 0.5% [v/v] in the donor solution. 300 μL of the donor solution was added to the donor wells (*V*_D_) and the donor filter plate was carefully placed on the acceptor plate so that the coated membrane was in contact with both the donor solution and the acceptor buffer. In principle, the test compound diffused from the donor well through the lipid membrane (Area = 0.28 cm^2^) to the acceptor well. The concentration of the drug in both donor and acceptor wells was assessed after 3, 4, 5, and 6 h of incubation in quadruplicate using the UV plate reader Spark (Tecan Group Ltd., Switzerland) at the maximum absorption wavelength of each compound. Besides that, the solution of theoretical compound concentration, simulating the equilibrium state established if the membrane were ideally permeable was prepared and assessed as well. Concentration of the compounds in the donor and acceptor wells and equilibrium concentration were calculated from the standard curve and expressed as the permeability (*P*_e_) according to the equation

$$P_{e}=C\times -\ln\left(1-\frac{{\left[drug\right]}_{acceptor}}{{\left[drug\right]}_{equilibrium}}\right)$$

where $C=\left(\frac{V_{D}\times V_{A}}{\left(V_{D}+V_{A}\right)\times \mathrm{Area}\times \mathrm{Time}}\right)$

### Microsomal Stability Assay

The 100 μL assay volume contained 5 μL human liver microsomes (Sekisui Xenotech Xtreme 200, PN H2620), 10 μL of NADPH generating system, 10 μL of 100 μM solution of compounds **8** (or **21**) in 10 mM sodium phosphate buffer (pH 7.4). Blank samples were devoid of either microsomes or test compounds. After 5 h of incubation at 37 °C, the reaction was stopped by adding MeOH (100 μL) and MeCN (100 μL), vortexing for 15 min, and then filtering through a 0.22 μm PTFE syringe filter into HPLC vial. Metabolites were detected using Agilent Infinity II 1290 UHPLC system coupled to Agilent 6470 QqQ mass spectrometer. Chromatographic conditions were maintained at gradient elution of 0.4 mL/min by 0.1% formic acid in water (A) and MeCN (B) (gradient: 0–0.5 min, 95:5; 0.5–3.0 min, 95:5 → 5:95, 3.0–4.0 min, 5:95; 4.0–5.0 min 95:5), thermostatted autosampler set to 15 °C and column thermostat equipped with Zorbax Eclipse plus RRHD C18 column (2.1 × 50 mm^2^, 1.8 μm, PN 959757–902) thermostatted at 30 °C. MS analysis was performed in MS2 scan mode in the range 100 to 800 (*m*/*z*), 500 ms scan time, 100 V at collision cell, 5 V cell acceleration voltage, positive mode. MS source parameters were set as follows: drying gas temperature 300 °C, drying gas flow 8 L/min, nebulizer pressure 35 psi, sheath gas temperature 400 °C, sheath gas flow 11 L/min, capillary voltage 2500 V.

### Cell-Based Assay

#### Cell Cultures

The human hepatocellular carcinoma HepG2 and human neuroblastoma SH-SY5Y cell lines were purchased from American Type Culture Collection (Manassas, VA, USA). The murine microglia BV2 cell line was a generous gift from Dr. Biljana Božić Nedeljković (University of Belgrade, Belgrade, Serbia). Cells were cultured in Advanced Dulbecco’s modified Eagle’s medium (Gibco, Thermo Fisher Scientific, Waltham, MA, USA) supplemented with 10% fetal bovine serum (FBS, Gibco), 2 mM L-glutamine, 50 μg/mL streptomycin and 50 U/mL penicillin (Sigma, MO, USA) in a humidified atmosphere of 95% air and 5% CO_2_ at 37 °C, and grown to 80% confluence. Before cell treatment, the complete medium was replaced with serum-free medium. Compounds **MW150**, **8**, **21** and hBChE inhibitor **A** were prepared as 30 mM DMSO stock solution and were used at concentrations of 1–100 μM.

#### Metabolic Activity

BV2 (5 × 10^3^/well), HepG2 and SH-SY5Y cells (1 × 10^4^/well for both) were seeded in 96-well plates and assessed by MTS (3-(4,5-dimethylthiazol-2-yl)-5-(3-carboxymethoxyphenyl)-2-(4-sulfophenyl)-2*H* tetrazolium, inner salt) assay for their response to compounds **MW150**, **8**, **21** and hBChE inhibitor **A**. Cells were treated with compounds (1–100 μM) in serum-free medium. Metabolic activity was determined after 24 h using the CellTiter 96 Aqueous One Solution Cell Proliferation Assay (Promega, WI, USA), in accordance with the manufacturer’s instructions. Absorbance was measured with an automatic microplate reader (Tecan Safire^2^, Switzerland) at a wavelength of 492 nm. Results are presented as a percentage of the DMSO control.

#### Cytotoxicity and Gliaprotection Assay

The cytotoxicity on SH-SY5Y and BV2 cells, as well as the gliaprotective effect of **MW150**, **8**, **21**, and hBChE inhibitor **A** in the LPS cell model of neuroinflammation, were determined using the fluorescent intercalator 7-AAD, assessed by flow cytometry. For cytotoxicity, SH-SY5Y and BV2 cells were seeded in 24-well culture plates (2 × 10^4^/well) and the next day treated with test compounds (1, 5, and 10 μM). For the gliaprotection, BV2 cells were seeded in 24-well culture plates (2 × 10^4^/well) and the next day pretreated with test compounds **6** (**MW150**), **8** and **21** (1, 2.5, 5, and 10 μM) for 1 h, followed by LPS (1 μg/mL; L6529; *E. coli* O55:B5; Sigma-Aldrich, MO, USA) stimulation. After 24 h treatment, cells were harvested and washed with cold PBS, and labeled with 7-AAD (2 μg/mL; Sigma-Aldrich) for 10 min at room temperature. Cells were then analyzed for cytotoxicity by flow cytometry on an Attune NxT flow cytometer (Thermo Fisher Scientific). The percentage of 7-AAD positive (7-AAD^pos^) cells was evaluated using FlowJo software (FlowJo, LLC, Ashland, OR, USA) and recorded relative to control cells treated with DMSO.

#### Quantification of Nitrite

The effect of tested compounds on the pro-inflammatory effect of LPS was determined by measuring nitrite in the culture supernatants of BV2 cells using the Griess assay. BV2 cells were seeded in 12-well culture plates (5 × 10^4^ /well) and stimulated after 24 h with 1 μg/mL LPS (L6529; *Escherichia coli* O55:B5; Sigma-Aldrich, MO, USA) in serum-free medium in the absence or presence of compounds at concentrations of 1, 2.5, 5, and 10 μM. After 24 h of stimulation, aliquots of the cell supernatants were collected and obtained by centrifugation at 2000 rpm for 5 min and were then either stored at –80 °C or used immediately to determine the nitrite content, an indicator of nitric oxide (NO) production. The secretion of NO into culture supernatants of activated microglia was determined using Griess reagent kits (Promega, WI, USA), according to the manufacturer’s instructions. Absorbance was measured at 550 nm using a microplate reader (Tecan Safire^2^ Switzerland). NaNO_2_ was used as the standard to calculate nitrite concentrations (in μM range).

#### Quantification of IL-6 and TNF-α

IL-6 and TNF-α levels in BV2 cell culture supernatants were quantified using commercial sandwich ELISA kits (Mouse TNFα ELISA kit #ab208348 and Mouse IL-6 ELISA Kit, Abcam) according to the manufacturer’s instructions. BV2 cells were seeded in 6-well plates (2 × 10^5^ cells/well), pretreated with test compounds (1–10 μM) or DMSO control for 1 h, and then stimulated with LPS (1 μg/mL) for 24 h. Culture supernatants were collected, centrifuged, and stored at −80 °C until analysis. Briefly, 50 μL of standards or samples and 50 μL of antibody cocktail were added to 96-well plates and incubated for 1 h at room temperature. Wells were washed, 100 μL 3,3′,5,5′-tetramethylbenzidine substrate was added, and plates were incubated for 10 min in the dark. The reaction was stopped, and absorbance was read at 450 nm. Cytokine concentrations were determined from standard curves and expressed in pg/mL.

#### Statistical Analysis

All the analyses were carried out at least in triplicate, and data were expressed as mean ± SEM. To compare differences among groups, one-way ANOVA followed by Tukey’s test (Prism 7; GraphPad Software, San Diego, CA) was used. Differences at the level of *p* < 0.05 were considered statistically significant.

### *In Vivo* Assays

#### Behavioral Testing Protocol

In the scopolamine and LPS mouse models PA, NOR, and MWM tasks were applied to evaluate the effects of compounds **MW150**, **8**, **21**, and the selective hBChE inhibitor **A** on distinct types of learning and memory. To examine potential effects of the tested compounds on motor coordination and general activity, the locomotor activity and the rotarod tests were conducted. In the scopolamine model, the PA and NOR tasks were first used for dose selection for subsequent experiments, which involved assessment of LPS-induced spatial memory impairment using the MWM and/or PA task.

Briefly, two series of experiments were conducted (Figure S5). In the first series, compounds **6** (**MW150**), **8**, and the selective hBChE inhibitor **A** were initially evaluated for their effects on learning and memory using the PA task (‘pre-LPS PA task’). Subsequently, these three compounds were administered once daily for 10 consecutive days. During the last six days of compound treatment, LPS was also administered i.p., 5 h before each compound dose. Over the course of these 6 days, the acquisition phase of the MWM task was conducted. One day after completion of the acquisition phase, the retention (drug-off) trial of the MWM task was performed. Finally, 24 h after the MWM retention trial, the compounds were administered once more, followed by the post-LPS PA task. In the second series of experiments (done for **21**), the pre-LPS PA and post-LPS PA tasks were omitted, and the effects of 10-day treatment with **21** on spatial learning and memory in the LPS model were measured in the MWM task.

#### Animals and Housing Conditions

In this *in vivo* study, adult male Albino Swiss (CD-1) mice (18–22 g) obtained from the Animal Breeding Farm of the Faculty of Pharmacy, Jagiellonian University Medical College, were used in the scopolamine model. For the LPS model, adult male C57BL/6J mice of comparable weight and age from the same breeding facility were employed. The animals were housed in groups of ten per cage under controlled environmental conditions (temperature 22 ± 2 °C; relative humidity 55 ± 10%; 12 h light/dark cycle) with unrestricted access to food and water. Standardized housing conditions were maintained throughout the experiment, including tree bedding (Transwior, Poland) and cage enrichment (tunnels, nesting material, wooden igloos, *etc.*). For behavioral testing, mice were randomly assigned to experimental groups (7–11 animals per group). All behavioral testing was conducted between 9 a.m. and 5 p.m. Following *in vivo* testing, animals were immediately euthanized, and their tissues were collected and stored for subsequent *ex vivo* analyses. All procedures involving maintenance and treatment of laboratory animals were approved by the first Local Ethics Committee in Krakow and were carried out in full accordance with Polish and EU regulations on animal research (Directive 2010/63/EU).

#### Drugs and Dose Selection for the *In Vivo* Assays

Scopolamine hydrobromide (Sigma Aldrich, Poland) at a dose of 1 mg/kg was used to induce learning and memory deficits in mice.^68,100^ For the *in vivo* experiments, scopolamine hydrobromide was dissolved in distilled water (Polfa Kutno, Poland) and administered subcutaneously (s.c.) 30 min before behavioral tests during the acquisition (training) trial of the PA and NOR tasks. LPS from *E. coli* (O111:B4, Sigma-Aldrich, Poland) was used to induce neuroinflammation in mice.^66,67^ For this purpose, LPS was freshly prepared in distilled water (Polfa Kutno, Poland) and administered intraperitoneally (i.p.) to mice in a cumulative dose of 12 mg/kg per mouse in two protocols: for **MW150**, **8** and **A** at doses 2.5 mg/kg (3 days) and 1.5 mg/kg (3 days) and for **21** at doses of 2.5 mg/kg (2 days), 2 mg/kg (2 days), and 1.5 mg/kg (2 days). Compounds **MW150**, **8**, **21**, and the selective hBChE inhibitor **A** were suspended in 1% Tween 80 (Sigma Aldrich, Poland) and administered intraperitoneally (i.p.) 60 min before the acquisition trial of the PA, NOR and MWM tasks. In the rotarod and locomotor activity tests, compounds **MW150**, **8**, **21**, and the selective hBChE inhibitor **A** were likewise administered i.p. 60 min before testing.

As noted above, in the scopolamine model, the PA and NOR tasks were employed as screening assays to evaluate the antiamnesic properties of **MW150**, **8**, **21** and the selective hBChE inhibitor **A**, and to facilitate dose selection for subsequent experiments. Accordingly, the pharmacological activity of three doses of each compound (5, 10 and 30 mg/kg) was assessed in these assays. In the rotarod and locomotor activity tests of **MW150**, **8** and the selective hBChE inhibitor **A**, the highest dose that proved effective in NOR task (10 mg/kg), and three doses of **21** (5, 10, and 30 mg/kg) were evaluated to exclude any potential influence of compounds on the animals’ motor performance.

#### Behavioral Tests

##### Passive Avoidance Task

The PA apparatus (Panlab Harvard Apparatus, Spain) consisted of a large illuminated white compartment (26 cm × 26 cm × 34 cm) and a small dark compartment (13 cm × 7.5 cm × 7.5 cm), separated by a guillotine door (5 cm × 5 cm). The PA task consisted of two trials: the acquisition (conditioning) trial and the retention (drug-off) trial, conducted 24 h apart.^68^ On the first day (acquisition trial), the test compounds (**MW150**, **8**, **21**, the selective hBChE inhibitor **A**, and vehicle) were administered. Before the acquisition trial, scopolamine or LPS was also administered at designated time points. Each mouse was then placed in the illuminated compartment for a 30-s habituation period (with the guillotine door closed). After habituation, the door was opened, initiating a 180-s testing period. Upon entry into the dark compartment, the guillotine door automatically closed, and a mild foot shock was delivered (intensity: 0.2 mA, duration: 2 s) through the grid floor. For each animal, the latency to enter the dark compartment was recorded. On the following day (retention trial), the mice were again placed into the illuminated compartment for 30 s of habituation, after which the latency to enter the dark compartment was measured. An increased latency to enter the dark compartment in the retention trial in scopolamine- or LPS-treated mice receiving **MW150**, **8**, **21**, or **A–** compared to the scopolamine/LPS control group was interpreted as an indicator of antiamnesic (procognitive) activity of the tested compounds.

##### Novel Object Recognition Task

The experiment was conducted in opaque black boxes with dimensions of 50 × 50 × 50 cm^3^.^101^ The three-day procedure consisted of habituation to the test arena (without objects) for 10 min on the first day (T0), followed by a training trial (T1) and a testing trial (T2) separated by a 24 h intertrial interval.^102^ During T1 (familiarization phase), two identical objects (A and B) were placed in the opposite corners of the arena, approximately 5 cm from the walls. During T2 (recognition phase), one of the familiar objects (B) was replaced with a novel object (C), so that the animals were exposed to A (familiar) and C (novel) objects. Both T1 and T2 trials lasted 10 min, after which the animals were returned to their home cages. White plastic round containers (height 5 cm, diameter 5 cm) fixed to the floor with double-sided tape served as objects A and B, while green cuboids of similar dimensions served as object C. The objects were stable enough to prevent displacement by the mice. The sequence and position of the objects were randomly assigned for each animal. Exploration was defined as looking, licking, sniffing, or touching the object while sniffing, but not leaning against, standing, or sitting on the object. Mice that explored both objects for less than 5 s during either T1 or T2 were excluded from analysis. An experimenter blind to the treatment group recorded the exploration time (E) for each object during T2. The discrimination index (DI) was calculated using the following formula: DI = (EC – EA)/(EA + EC) × 100%. In this task, DI values near zero indicate no preference, while positive values indicate a preference for the novel object, and negative values indicate a preference for the familiar object.

##### Morris Water Maze Task

The MWM consisted of a circular, gray-painted plastic pool (120 cm in diameter, 60 cm in height), filled with water (up to about 48 cm below the edge to prevent animals from jumping out). Water temperature was maintained at 23 ± 1 °C. The pool was divided into four equal quadrants (compass locations: NE, NW, SE, SW) and tracked using a computerized video tracking system (SMART, ver. 3.0; Panlab, Spain). An escape platform made of transparent Plexiglas (11 cm in diameter, 47 cm in height) was positioned at a fixed location (the center of the NW quadrant, *i*.*e*., the target quadrant), submerged 1 cm below the water surface to remain invisible to the swimming mice. The maze was illuminated at an intensity of 45 lx.^68^ During the spatial acquisition trial (6 consecutive days), mice underwent four training sessions per day, with sessions spaced 4 h apart. Mice were trained to escape from water by reaching a hidden platform, whose location could be identified using distal extra-maze cues (A4-size sheets of black laminated paper with colored geometric symbols) attached to the room walls and serving as navigation points. The cues differed in color and shape and were kept constant throughout the experiment. The experimenter remained stationary in a fixed position during all sessions, providing an additional distal reference cue. For each trial, mice were placed in the water starting from a different randomly chosen quadrant that did not contain the platform, while the platform itself remained in a constant location. Each trial lasted 60 s; if a mouse failed to locate the platform within this time, it was gently guided to it and allowed to remain there for 15 s. The escape latency (time to reach the hidden platform) was recorded for each trial across days 1–6. If a mouse did not find the platform’s place within 60 s, it was given a latency score of 60 s. On the seventh day (24 h after the last training session; drug-off trial), the platform was removed, and a probe trial was performed (without drug treatment–assessment of memory retention). During this probe trial, the following parameters were recorded and analyzed: (1) latency to enter the NW quadrant, (2) time spent in the NW quadrant, (3) time spent at the target zone (the previous platform location), and (4) the number of NW quadrant entries.

##### Rotarod Test

Three days before the rotarod test, mice were pretrained on the rotarod apparatus (Rotarod apparatus, May Commat RR0711, Turkey; rod diameter: 2 cm) rotating at a constant speed of 18 rpm. During each training session, mice were placed on the rotating rod for 3 min, with an unlimited number of trials. The test session was conducted 24 h after the final training trial. Sixty minutes after the administration of **MW150**, **8**, **21**, **A**, or vehicle, mice were tested on the rod rotating at 6, 18, and 24 rpm. Motor impairment was defined as the inability to remain on the rotating rod for at least 1 min. The results are expressed as the mean time spent on the rotarod.^103^

##### Locomotor Activity Test

The locomotor activity test was conducted using activity cages (40 cm × 40 cm × 30 cm) equipped with IR beam sensors (Activity Cage 7441, Ugo Basile, Italy), which were connected to an automated counter that recorded beam interruptions. Before the experiment, mice were habituated to the activity cages for 15 min. Subsequently, animals were pretreated intraperitoneally with compounds **MW150**, **8**, **21**, **A**, or vehicle, and then individually placed in activity cages located in a sound-attenuated room. Spontaneous locomotor activity, expressed as the number of beam crossings, was recorded for 60 min (97).

##### Data Analysis

Data from behavioral experiments were analyzed using GraphPad Prism software (version 9.0, CA, USA). All numerical results are presented as mean ± standard error of the mean (SEM). Statistical analyses were performed using repeated measures analysis of variance (ANOVA), one-way ANOVA followed by Dunnett’s, Tukey’s, and Sidak’s *post hoc* test. In all analyses, *p* < 0.05 was considered statistically significant.

#### *Ex Vivo* Western Blot Analysis

##### Tissue Collection and Sample Preparation

Twenty-four hours after the last behavioral test, male C57BL/6J mice were sacrificed by rapid decapitation under nonstressed conditions. The brains were rapidly removed, and the brain structures, *i*.*e*., hippocampus and frontal cortex, were dissected on ice-cold glass plates. Brain structures were rapidly frozen in liquid nitrogen and stored at −80 °C until use. Frozen brain tissues were homogenized in 2% sodium dodecyl sulfate (SDS) solution (TOKU-E, Bellingham, WA, USA) and centrifuged at 18,000*g* for 20 min at room temperature. After measuring the total protein concentration in supernatants with bicinchoninic acid (BCA) method (Thermo Fisher Scientific, Waltham, MA, USA), samples containing equal amounts of protein were mixed at a 4:1 (v/v) ratio with NuPAGE LDS Sample Buffer (#NP0007, Invitrogen, Carlsbad, CA, USA) supplemented with 10% Bond Breaker TCEP solution (#77720, Thermo Scientific, USA) and incubated for 10 min at 70 °C.

##### Immunodetection of Inflammatory Markers

Samples were loaded onto 10% or 12% polyacrylamide gels (depending on the molecular weight of the assayed protein) along with molecular weight marker (PRL0202, HighQu Gmbh, Kraichtal, Germany), separated under constant voltage of 150 V and transferred onto PVDF membranes (Roche Diagnostics, Mannheim, Germany) under 100 V for 90 min. After protein transfer, membranes were cut, allowing for simultaneous incubation with different antibodies. Membranes were blocked with 3% nonfat dry milk (for unphosphorylated proteins) solution or 1% bovine serum albumin (BSA) (for phosphorylated proteins) solutions in TTBS (TRIS-buffered saline with 0.05% Tween 20) for 1 h and incubated overnight with primary antibodies (ABclonal, Woburn, MA, USA) dissolved in SignalBoost Immunoreaction Enhancer Kit (Merck-Millipore, Darmstadt, Germany), raised against amyloid precursor protein (APP) (rabbit monoclonal IgG, A17911, 1: 1000), interleukin-1β (IL-1 β) (rabbit monoclonal IgG, A22257, 1: 1000), myeloid differentiation primary response 88 (MyD88) (rabbit monoclonal IgG, A21905, 1: 2000), nuclear factor kappa-light-chain-enhancer of activated B cells (NF-κB) p65 (rabbit monoclonal IgG, A19653, 1: 10.000), phospho-p38 MAPK-T180 (rabbit monoclonal IgG, AP1508, 1: 1000), p38 MAPK (rabbit monoclonal IgG, A4771, 1: 1500) and Toll-like receptor type 4 (TLR4) (rabbit polyclonal IgG, A5258, 1: 1000) at 4 °C. Next day, membranes were washed 3 times in TTBS for 15 min, incubated for 1 h with HRP-conjugated goat antirabbit secondary antibody (#1–348, Millipore, 1: 4500) and subsequently washed in TTBS, 3 times for 15 min. Chemiluminescence signal was developed with Pierce ECL Western Blotting Substrate (#32106, Thermo Fisher Scientific, USA) and visualized with ChemiDoc MP Imaging System (Bio-Rad, Hercules, CA, USA). Adjusted volume intensities of individual protein bands were calculated using Image Lab 6.1 software (Bio-Rad, USA). β-actin, which served as internal loading control, was detected with HRP-conjugated mouse monoclonal IgG1 (sc-47778, Santa Cruz Biotechnology, Dallas, TX, USA, 1: 1000). Results were calculated and presented as fold change *vs* vehicle group. One-way analysis of variance (ANOVA) and Dunnett’s *post hoc* tests were used to statistically evaluate the results. ROUT test was applied for identification of possible outliers (*Q* = 1%). A *p* < 0.05 was considered statistically significant.

## Supplementary Material





## Notes

X-ray crystallography data collection and refinement statistics for hBChE and p38α MAPK; kinase selectivity profile; *in vitro* pharmacokinetics data (results of BBB-PAMPA assay; major metabolites of **8** and **21** after a 5 h incubation with human liver microsomes); *in vitro* cytotoxicity data (metabolic activity of SH-SY5Y, BV2, and HepG2 cells treated with **MW150**, **8**, **21**, and a selective hBChE inhibitor **A**; *in vitro* toxicity of **MW150**, **8**, **21**, and a selective hBChE inhibitor **A** using 7-AAD); *in vivo* data (detailed experimental protocol, effects of **MW150**, **8**, and **A** and **21** on motor coordination and locomotor activity, additional analysis of the PA task results from the LPS model of neuroinflammation tests; results of Western blot analysis of neuroinflammatory markers in the hippocampus and frontal cortex of male C57BL/6J mice treated with **21**), ^1^H and ^13^C NMR spectra for intermediates and tested compounds; HPLC chromatograms for tested compounds (PDF)
Molecular formula strings (CSV)

## Abbreviations Used

7-AAD: 7-aminoactinomycin D
Aβ: amyloid β
ACh: acetylcholine
(h)­AChE: (human) acetylcholinesterase
AD: Alzheimer’s disease
(h)­BChE: (human) butyrylcholinesterase
ChE: cholinesterase
DCM: dichloromethane
DMF: *N*,*N*-dimethylformamide
DMSO: dimethyl sulfoxide
IC_50_: half maximal inhibitory concentration
LPS: lipopolysaccharide
MAPK: mitogen-activated protein kinase
MTDLs: multitarget directed ligands
PDB: Protein Data Bank
RA: residual activity of the enzyme
TFA: trifluoroacetic acid
THF: tetrahydrofuran
