# Supplementary material for: Dual Inhibitors of p38α Mitogen-Activated Protein Kinase and Butyrylcholinesterase for the Modulation of Neuroinflammation and Cognitive Dysfunction
Source: J Med Chem. 2026 Aug 10;69(16):19891–917. doi: 10.1021/acs.jmedchem.6c01461 (PMC13528200; doi:10.1021/acs.jmedchem.6c01461)
Supplement: Supplementary file 1 [file jm6c01461_si_001.pdf]

SUPPORTING INFORMATION

Dual Inhibitors of p38α Mitogen-Activated Protein Kinase and Butyrylcholinesterase for the Modulation of Neuroinflammation and Cognitive Dysfunction

Damijan Knez[a#], David Malinak[b,c#], Anže Meden[a], Svit Ferjančič Benetik[a], Anja Pišlar[a], Zuzana Kohoutova[b,c], Iveta Vondrackova[b,c], Miroslav Psotka[b,c], Antonin Lycka[c], Lenka Pulkrabkova[b], Ondrej Soukup[b], Adam Skarka[c], Rudolf Andrys[c], Xavier Brazzolotto[d], Alexandre Igert[d], José Dias[d], Florian Nachon[d], Jan Detka[e], Filip Targosiński[e], Dziyana Hliabovich[e], Joseph S. Brunzelle[f], Ludmilla Shuvalova[f], Kinga Sałat[e], Saktimayee Mitra Roy[f], Daniel Martin Watterson[f], Kamil Musílek[b,c*], Stanislav Gobec[a*]

[a] *University of Ljubljana, Faculty of Pharmacy, Department of Pharmaceutical Chemistry, 1000 Ljubljana, Slovenia*

[b] *University Hospital in Hradec Kralove, Biomedical Research Centre, 500 05 Hradec Kralove, Czech Republic*

[c] *University of Hradec Kralove, Faculty of Science, Department of Chemistry, 500 03 Hradec Kralove, Czech Republic*

[d] *Département de Toxicologie et Risques Chimiques, Institut de Recherche Biomédicale des Armées, Brétigny-sur-Orge 91220, France*

[e] *Department of Pharmacodynamics, Chair of Pharmacodynamics, Faculty of Pharmacy, Jagiellonian University Medical College, Krakow 30-688, Poland*

[f] *Department of Pharmacology, Northwestern University, Chicago, IL 60611, USA*

[#] These authors contributed equally; [*]*E-mail: kamil.musilek@uhk.cz; stanislav.gobec@ffa.uni-lj.si*

Table of Contents

TABLE OF CONTENTS **S2**

1. SUPPLEMENTARY RESULTS **S3**

1.1. SUPPLEMENTARY CHEMISTRY S3

1.2. X-RAY CRYSTALLOGRAPHY S6

1.3. KINASE SELECTIVITY PROFILE S10

1.4. *IN VITRO* PHARMACOKINETICS S12

1.5. *IN VITRO* CYTOTOXICITY S14

1.6. *IN VIVO* ASSAYS S16

LPS Model of Neuroinflammation – Additional Analysis of the PA Task Results *S18*

Western blot analysis *S20*

2. NMR SPECTRA **S22**

3. HPLC CHROMATOGRAMS **S55**

1. Supplementary Results

1.1. Supplementary Chemistry

The initial synthetic pathway for preparing 1-naphthyl-based dual inhibitors **21** and **22** proved to be challenging. Although sufficient amounts of both products for *in vitro* studies were obtained by the synthesis outlined in Scheme 1 (*see main text*), approximately 100 milligrams of **21** were needed for *in vivo* studies. One of the issues in the synthesis was dialkylation of ketone **2b**. Sodium hydride cleaved the proton at the α-position, producing an enolate that attacked the α-carbon of ethyl bromoacetate as a good nucleophile to form γ-ketoester **3b**. The α-carbon of **3b** was further alkylated to form **3c** (not isolated, detected on LC-MS, Scheme S1A). To reduce the formation of the dialkylated side product, NaHMDS was used as the base (1). However, the desired γ-ketoester **3b** was obtained with an even lower yield of 27%; this was due to the presence of a major side product **3d** (Scheme S1B). The HRMS spectrum of **3d** was identical to that of the desired product; thus, **3d** can be regarded as a regioisomer. This hypothesis was proven by measuring the ^{1}H-^{15}N HMBC spectrum (Figure S1), where pyridine nitrogen correlated with protons of the acetate, as well as with protons of 1,4-dihydropyridine. Contrary to the desired product **3b**, where protons in pyridine positions C2-C6 and C3-C5 are magnetically equivalent and give two resonances with relative integral intensities 2:2, analogous protons of the side product **3d** are not equivalent, and four signals of 1,4-dihydropyridine were observed. Characteristic ^{1}H, ^{13}C, and ^{15}N chemical shifts of **3d** in DMSO-d_6 are collected in Table S1.

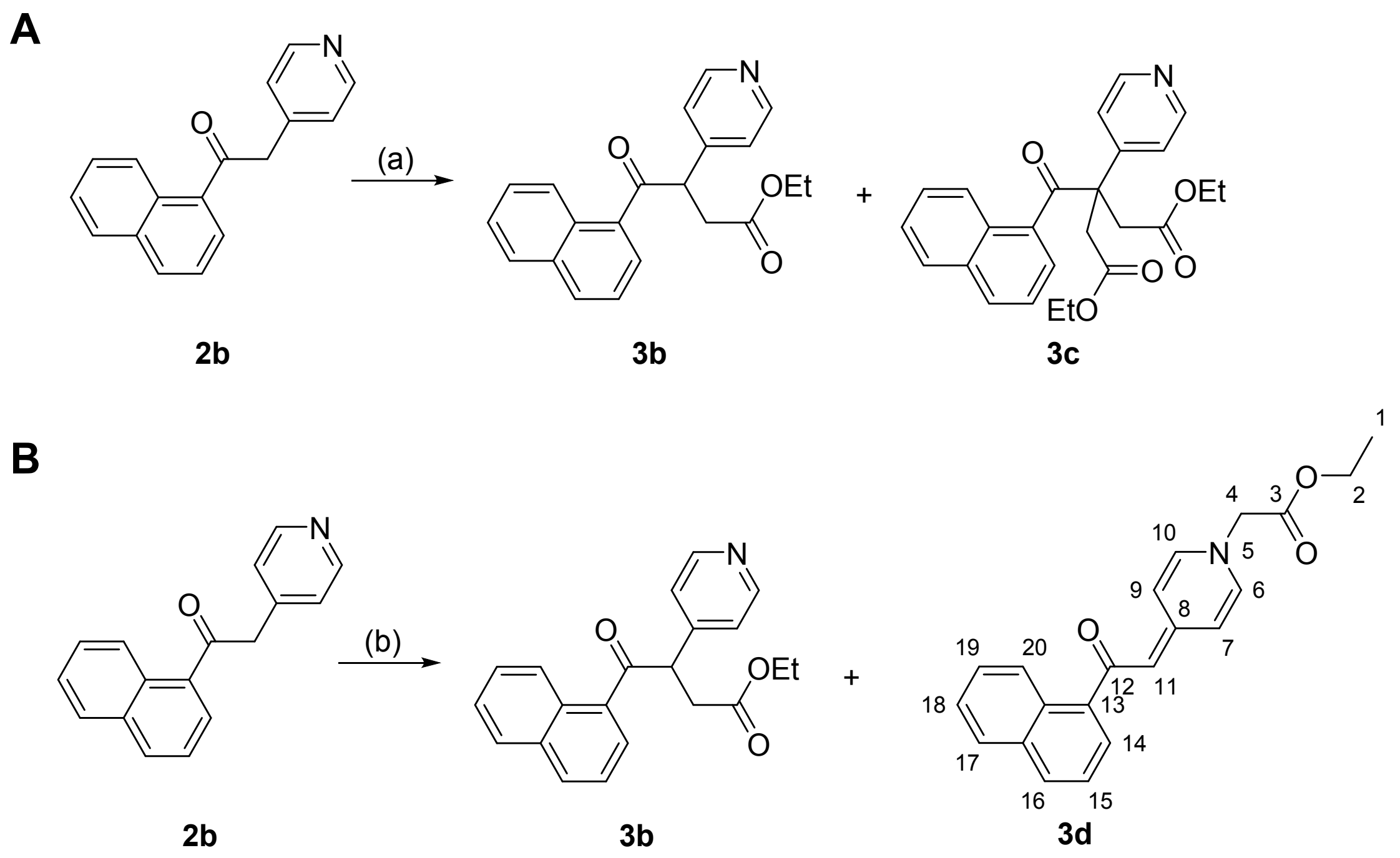

Scheme S1. Side products identified during the synthesis of compound **21**. *Reagents and conditions:* (a) ethyl bromoacetate, DMF, sodium hydride, 0 °C to rt; (b) i. NaHMDS, THF, –78 °C, 45 min; ii) ethyl bromoacetate, THF, –78 °C, 24 h.

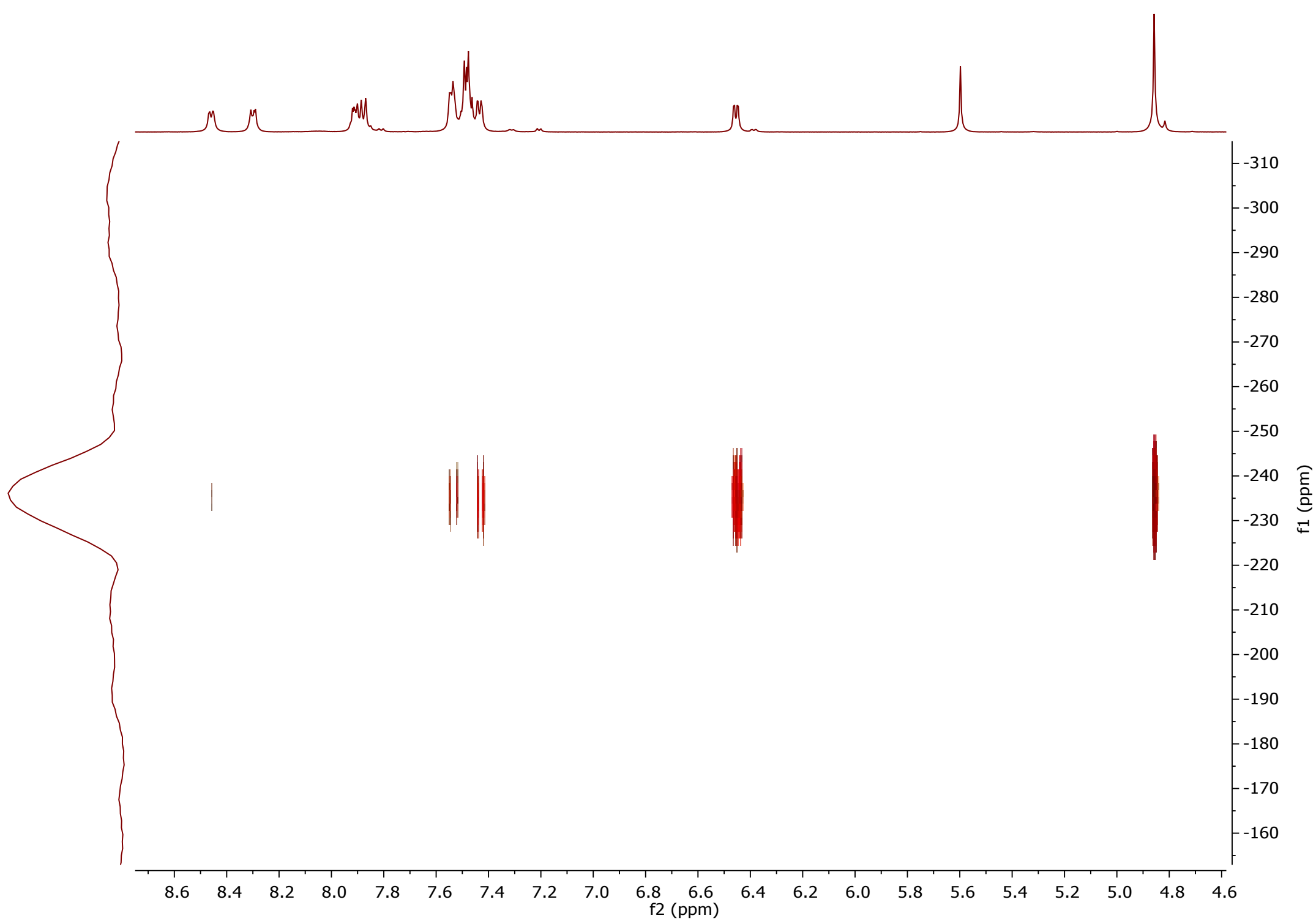

Figure S1. ^{1}H-^{15}N HMBC spectrum of **3d**.

Table S1. Characteristic ^{1}H, ^{13}C and ^{15}N chemical shifts of **3d** in DMSO-d_6.

H/C or N No.	^{1}H	^{13}C/^{15}N [a]	H/C or N No.	^{1}H	^{13}C
1	1.24	14.0	8	-	149.4
2	4.19	61.4	9	7.54	139.6
3	-	168.2	10	8.46	112.2
4	4.86	55.7	11	5.60	99.4
5	-	–234.6 [a]	12	-	188.4
6	7.43	138.3	13	-	143.0
7	6.46	115.6			

[a] δ(^{15}N), referenced to nitromethane

1.2. X-ray crystallography

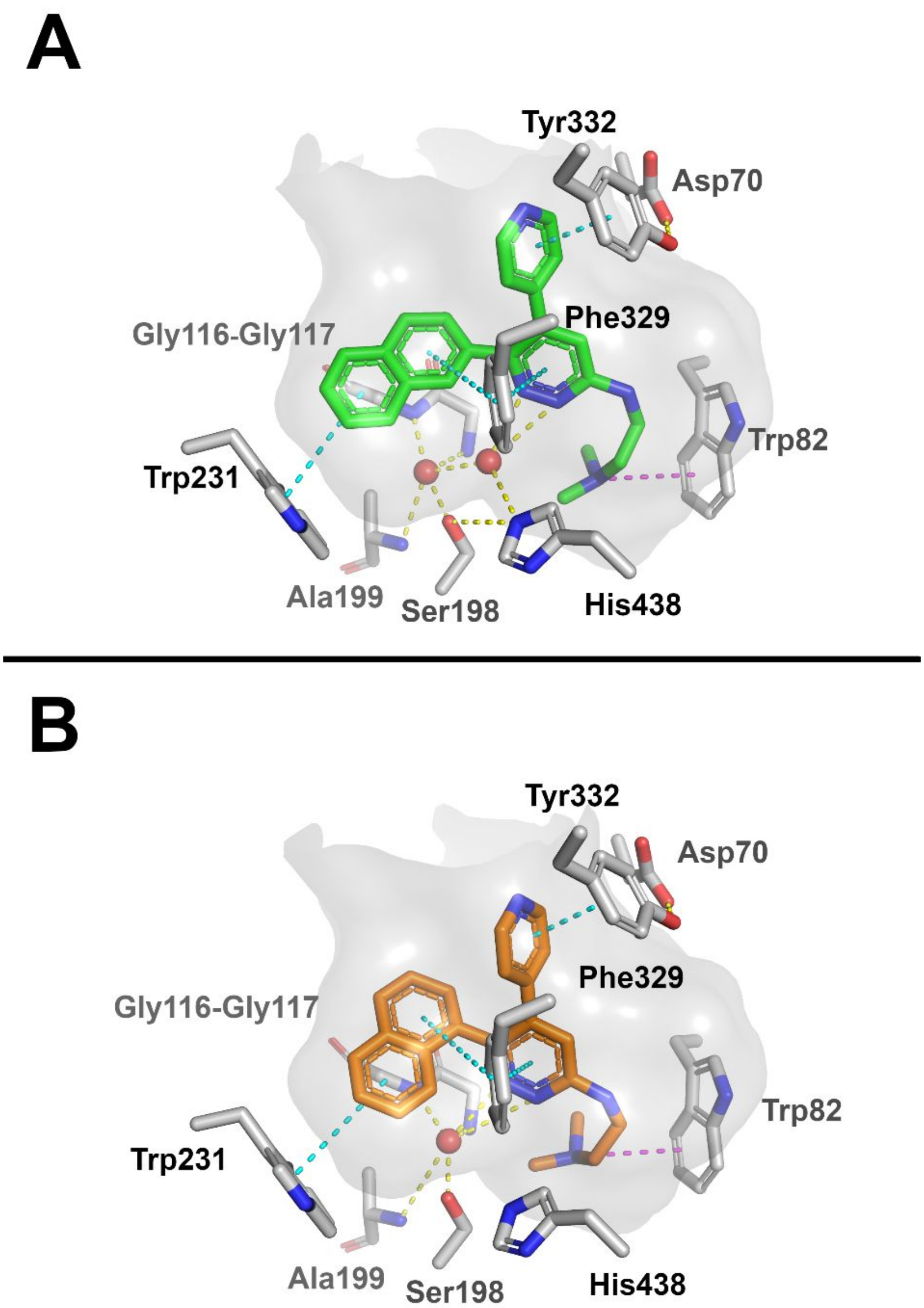

Figure S2. X-ray structures of **8** (**A**) and **21** (**B**) in complex with hBChE. Ligands and notable residues are represented as sticks. Carbon atoms are represented in light grey for hBChE residues, in green for **8**, and in orange for **21**, while nitrogen and oxygen atoms are represented in blue and red, respectively. Water molecules are represented as red spheres. Specific interactions are represented as dashed lines with H-bonds in yellow, π–π interactions in cyan, and a potential cation–π interaction in magenta. The grey semi-transparent surface represents the active site gorge of hBChE.

For **8**, the first water molecule stands in the oxyanion hole, with distances of 2.3, 2.8, 2.8, and 2.2 Å with the nitrogen atoms of Gly117, Gly116, and Ala199, respectively, and 2.2 Å with the Oγ atom of Ser198. Additionally, this water molecule interacts with the second water molecule at a distance of 2.7 Å. Furthermore, this second water molecule interacts with the pyridazine N^1 and N^2 atoms with respective distances of 2.8 and 3.2 Å, and at 3.1 Å from the *Nε*2 nitrogen atom of His438. For **21**, the water molecule in the oxyanion hole lies at 2.6 Å from the nitrogen atom of Gly117, 2.9 Å from the nitrogen atom of Gly116, 3.6 Å from the nitrogen atom of Ala199, and 2.2 Å from the Oγ of Ser198. Furthermore, the water molecule interacts with pyridazine N^1 and N^2 atoms with distances of 3.1 and 3.6 Å, respectively.

Table S2. hBChE X-ray crystallography data collection and refinement statistics calculated using Phenix. R-work=Σ |Fo - |Fc|| / Σ |Fo|, where Fo and Fc are observed and calculated structure factors, respectively. R-free set uses about 5% randomly chosen reflections. Statistics for the highest-resolution shell are shown in parentheses.

	hBChE-21 (PDB 9GCR)	hBChE-8 (PDB 9GCQ)
Data Collection		
**X-ray source**	ESRF ID30B	SOLEIL Proxima-2A
**Wavelength (Å)**	0.9762	0.9801
**Resolution range (Å)**	49.09 - 2.84 (3.02 - 2.84)	48.93 - 2.62 (2.72 - 2.62)
**Space group**	I 4 2 2	I 4 2 2
**Unit cell (Å,°)**	153.926 153.926 127.471 90 90 90	154.74 154.74 127.14 90 90 90
**Total reflections**	224288 (35097)	614062 (33983)
**Unique reflections**	18355 (2986)	23171 (2090)
**Multiplicity**	12.2 (11.8)	26.5 (22.5)
**Completeness (%)**	99.79 (99.07)	99.2 (91.0)
**Mean I/sigma(I)**	9.98 (1.79)	21.1 (1.34)
**Wilson B-factor**	70.98	60.32
**R-merge**	0.1919 (1.436)	0.140 (1.625)
**R-meas**	0.2006 (1.501)	0.145 (1.693)
**R-pim**	0.05734 (0.429)	0.038 (0.466)
**CC1/2**	0.995 (0.674)	0.999 (0.71)
**CC***	0.999 (0.897)	1 (0.915)
Refinement statistics		
**Reflections used in refinement**	18346 (2983)	23171 (2090)
**Reflections used for R-free**	891 (126)	1159 (105)
**R-work**	0.1714 (0.2534)	0.1784 (0.2849)
**R-free**	0.2441 (0.3452)	0.2243 (0.3008)
**Number of non-hydrogen atoms**	4565	4537
**macromolecules**	4232	4248
**ligands**	258	203
**solvent**	75	86
**Protein residues**	526	527
**RMS(bonds)**	0.009	0.008
**RMS(angles)**	1.08	0.94
**Ramachandran favored (%)**	93.32	94.67
**Ramachandran allowed (%)**	6.68	4.95
**Ramachandran outliers (%)**	0.00	0.38
**Rotamer outliers (%)**	2.19	1.97
**Clashscore**	9.19	7.32
**Average B-factor**	77.51	68.64
**macromolecules**	75.34	67.35
**ligands**	115.99	99.00
**solvent**	67.24	60.77

Table S3. p38α MAPK X-ray crystallography data collection and refinement statistics. R-work = Σ |Fo – |Fc|| / Σ |Fo|, where Fo and Fc are observed and calculated structure factors, respectively. R-free set uses about 5% randomly chosen reflections. Statistics for the highest-resolution shell are shown in parentheses.

	**PDB 9D8G** (p38α:**8**)	**PDB 9D6P** (p38α:**21**)
Data collection		
**Space group**	P 21 21 21	P 21 21 21
**Wavelength (Å)**	0.8731	0.8731
**Unit cell parameters *a,b,c* (Å), angles (°)**	67.364, 74.558, 79.853, 90°, 90°, 90°	67.758, 74.556, 80.161, 90°, 90°, 90°
**Resolution (Å)**	42.37 - 2.13	51.75 - 2.132
**Outer Resolution Shell (Å)**	(2.206 - 2.13)	(2.208 - 2.132)
**Completeness (%)**	99.75 (99.11)	99.75 (98.91)
**R-merge**	0.1358 (1.563)	0.1104 (2.165)
**Mean I/σ(I)**	11.70 (1.98)	13.34 (1.36)
**Redundancy**	13.1 (12.1)	13.2 (13.8)
**No. of Unique Reflections**	23066 (2233)	23231 (2267)
Refinement statistics		
**R-work**	0.1978 (0.2817)	0.2119 (0.3334)
**R-free**	0.2475 (0.3697)	0.2449 (0.3854)
**Residues**	339	335
**Atoms**		
**Protein**	2740	2776
**Ligand**	108	108
**Solvent**	108	23
**Mean B-value (Å)**		
**Overall**	53.81	79.49
**Protein**	53.98	78.58
**Ligand**	52.34	79.81
**Solvent**	50.22	64.11
**RMSD bond lengths (Å)**	0.002	0.003
**RMSD bond angles (°)**	0.49	0.59
**Ramachandran plot, %**		
**Most Favored**	97.91	97.89
**Allowed**	2.09	2.11
**Disallowed**	0.00	0.00

1.3. Kinase Selectivity Profile

Table S1. Profiling of compounds **8** and **21** at 1 µM and 10 µM concentrations against 21 protein kinases.

Kinase	RA of protein kinase activity [%]			
	8		21	
	1 µM	10 µM	1 µM	10 µM
ABL1	104	107	103	99
ABL2	99	95	98	97
AKT1	108	96	107	93
CDK1/ CycA2	96	87	98	90
CK1δ	87	23	115	96
ERK1	102	94	101	94
ERK2	108	95	116	93
FAK	104	108	101	95
FGR	97	97	104	90
GSKβ	92	91	98	90
JNK3	83	47	93	68
LCK	99	80	96	81
MAPKAPK5	103	93	101	91
NLK	55	14	77	34
p38α	7	0	22	3
p38β	29	4	47	9
p38δ	99	96	104	91
p38γ	99	77	94	80
PDGFRβ	105	107	102	102
PIM2	109	98	114	99
RET	106	103	98	90

RA – residual activity (one independent experiment performed in singlicate)

Table S5. Assay parameters for the tested protein kinases.

#	Kinase Name	Kinase PQ Lot	Kinase External Vendor Lot	Kinase Concentration [ng/50 µL]	Kinase Concentration [nM][a]	ATP Concentration [µM]	Substrate Name	Substrate Lot	Substrate Concentration [µg/50 µL]
1	ABL1	005,21		5	1.3	0.3	Poly(Ala,Glu,Lys,Tyr)6:2:5:1	SIG_53H5516	0.125
2	ABL2	012,11		8	1.6	0.3	Poly(Ala,Glu,Lys,Tyr)6:2:5:1	SIG_53H5516	0.125
3	AKT1	004,5		50	11.9	1	GSK3(14-27)	45	2.0
4	CDK1/CycA2	005,21		15	2.2	0.3	RBER-CHKtide	120	1.0
5	CK1delta	002,11	INV_843704	1	0.3	0.3	Casein	SIG_SLBW5843	0.5
6	ERK1	002,14		25	7.2	1	RBER-CHKtide	121	2.0
7	ERK2	008,9		10	4.8	0.3	RBER-CHKtide	121	2.0
8	FAK	016,13		50	6.9	1	Poly(Glu,Tyr)4:1	SIG_20K5903	0.125
9	FGR	001,10		25	5.3	0.3	Poly(Glu,Tyr)4:1	SIG_20K5903	0.125
10	GSK3beta	003,27		75	19.7	0.3	RBER-CHKtide	121	1.0
11	JNK3	004,10		5	2.1	0.3	ATF2	14	2.0
12	LCK	003,22		20	4.3	0.3	Poly(Glu,Tyr)4:1	SIG_20K5903	0.125
13	MAPKAPK5	005,8		5	1.8	0.1	RBER-CHKtide	120	2.0
14	NLK	002,7		30	6.5	1	GSK3(14-27)	45	2.0
15	p38alpha	005,28		15	7.2	1	ATF2	14	1.0
16	p38beta	004,10		3	1.4	1	ATF2	14	1.0
17	p38delta	001,8		2	0.9	1	RBER-CHKtide	120	2.0
18	p38gamma	002,8		10	4.7	0.1	RBER-IRStide	46	2.0
19	PDGFRbeta	013,18		10	2.3	0.3	Poly(Ala,Glu,Lys,Tyr)6:2:5:1	SIG_53H5516	0.125
20	PIM2	002,11		50	15.6	0.3	GSK3(14-27)	45	1.0
21	RET	006,12		100	24.9	1	Poly(Glu,Tyr)4:1	SIG_20K5903	0.125

[a] *Maximal molar enzyme assay concentrations, implying enzyme preparations exclusively containing 100% active enzyme*

1.4. *In Vitro* Pharmacokinetics

Table S6. Prediction of blood-brain barrier penetration using BBB-PAMPA for studied compounds expressed as P_e ± SEM (n = 2 for compounds, n = 3 for reference drugs).

Compound	P_e ± SEM ($\times10^{-6}$ cm×s^{-1})	CNS (+/–)
**6 (MW150)**	9.6 ± 0.07	CNS+
**8**	5.5 ± 0.9	CNS+
**21**	4.3 ± 0.03	CNS+
**A**	10.2 ± 0.2	CNS+
donepezil	*9.8 ± 0.4*	CNS+
rivastigmine	*12.6 ± 2.9*	CNS+
obidoxime	*0.34 ± 0.081*	CNS–
cefuroxime	*0.31 ± 0.09*	CNS–

CNS+ (high BBB permeation predicted): P_e ($\times10^{-6}$ cm×s^{-1}) > 4.0
CNS– (low BBB permeation predicted): P_e ($\times10^{-6}$ cm×s^{-1}) < 2.0
CNS+/– (BBB permeation uncertain): P_e ($\times10^{-6}$ cm×s^{-1}) from 4.0 to 2.0

Table S7. Major metabolites of **8** and **21** after a 5 h incubation with human liver microsomes.

Cpd	Structure	$[M+H]^+$	Metabolite 5 h/Parent 0 h [% area]	Cpd	Structure	$[M+H]^+$	Metabolite 5 h/Parent 0 h [% area]
**8**	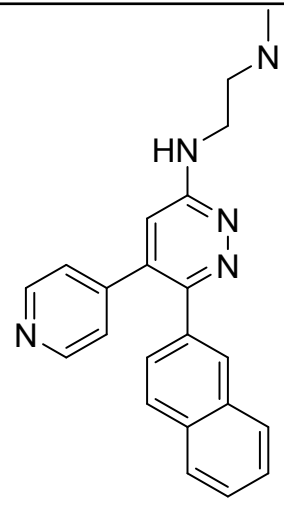	370	/	**21**	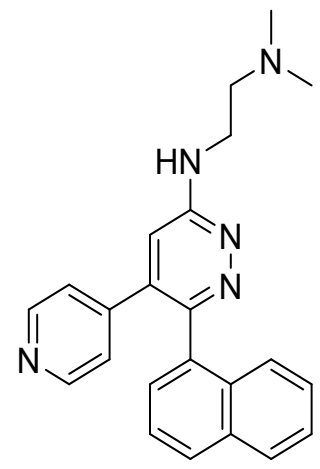	370	/
/	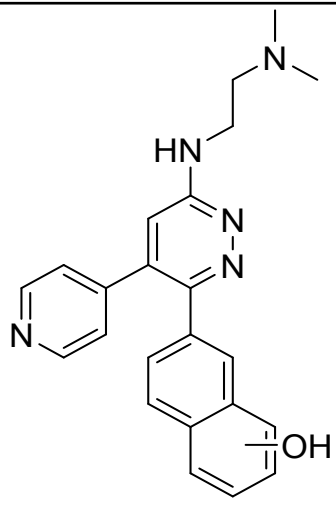 or 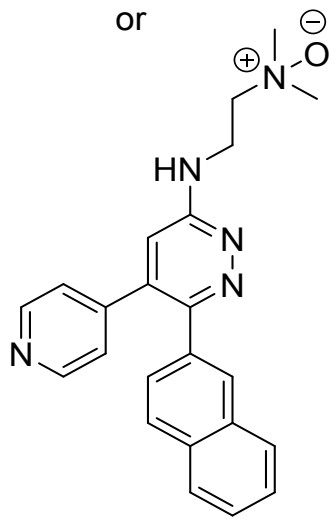	386	26.8%	/	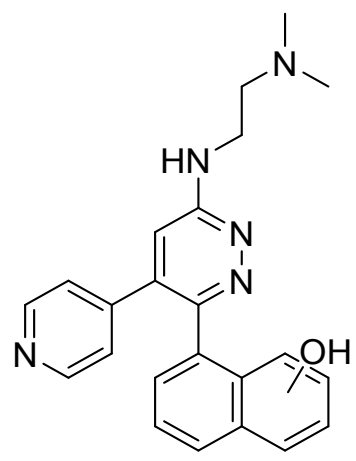 or 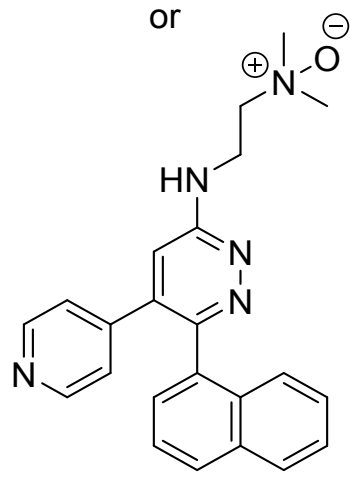	386	8.9%
/	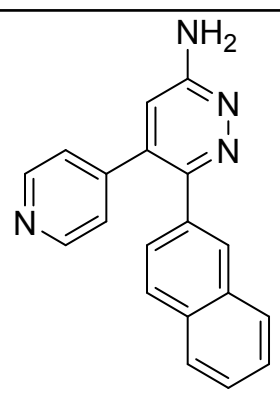	299	17.2%	/	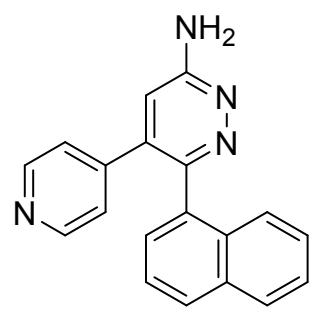	299	7.4%
/	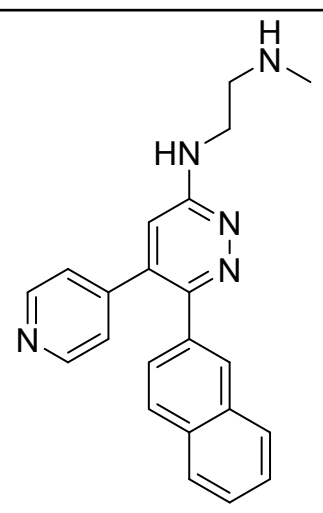	356	9.5%	/	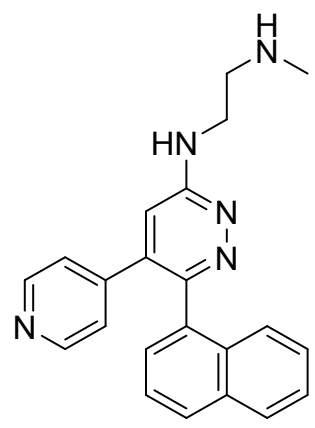	356	19.8%
/	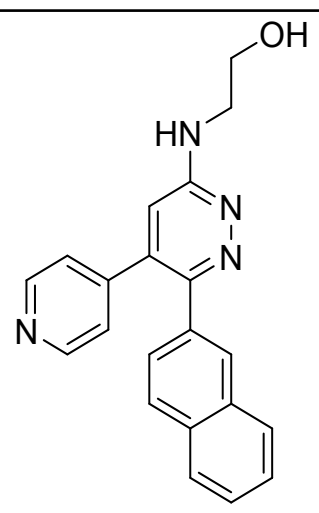	341	1.2%	/	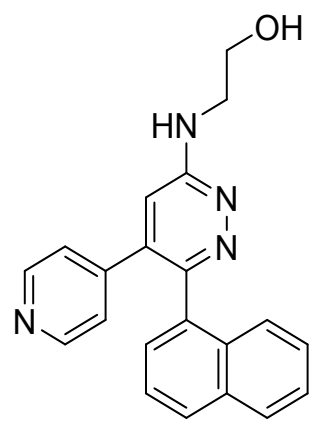	341	1.0%

1.5. *In Vitro* Cytotoxicity

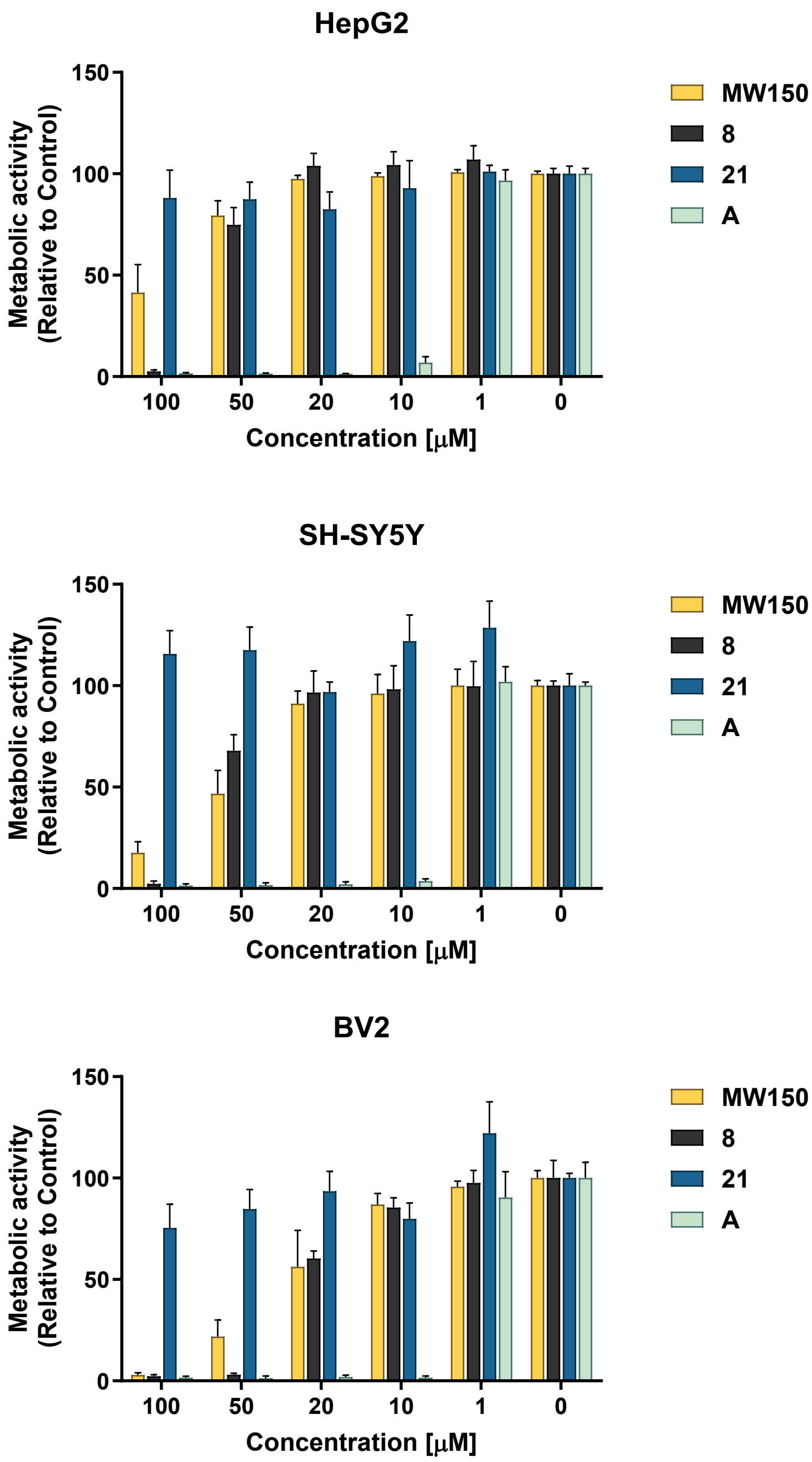

Figure S3. *In vitro* metabolic activity of SH-SY5Y, BV2, and HepG2 cells treated with **MW150**, **8**, **21**, and a selective hBChE inhibitor **A**. SH-SY5Y, BV2, and HepG2 cells were treated with increasing concentrations of the compounds (1–100 μM) in serum-free medium. After 24 h, we evaluated the cell viability using the metabolic MTS assay. The control group (DMSO) was considered to have 100% cell viability. Data are presented as means (± SEM) of at least three independent experiments, each carried out in quadruplicate.

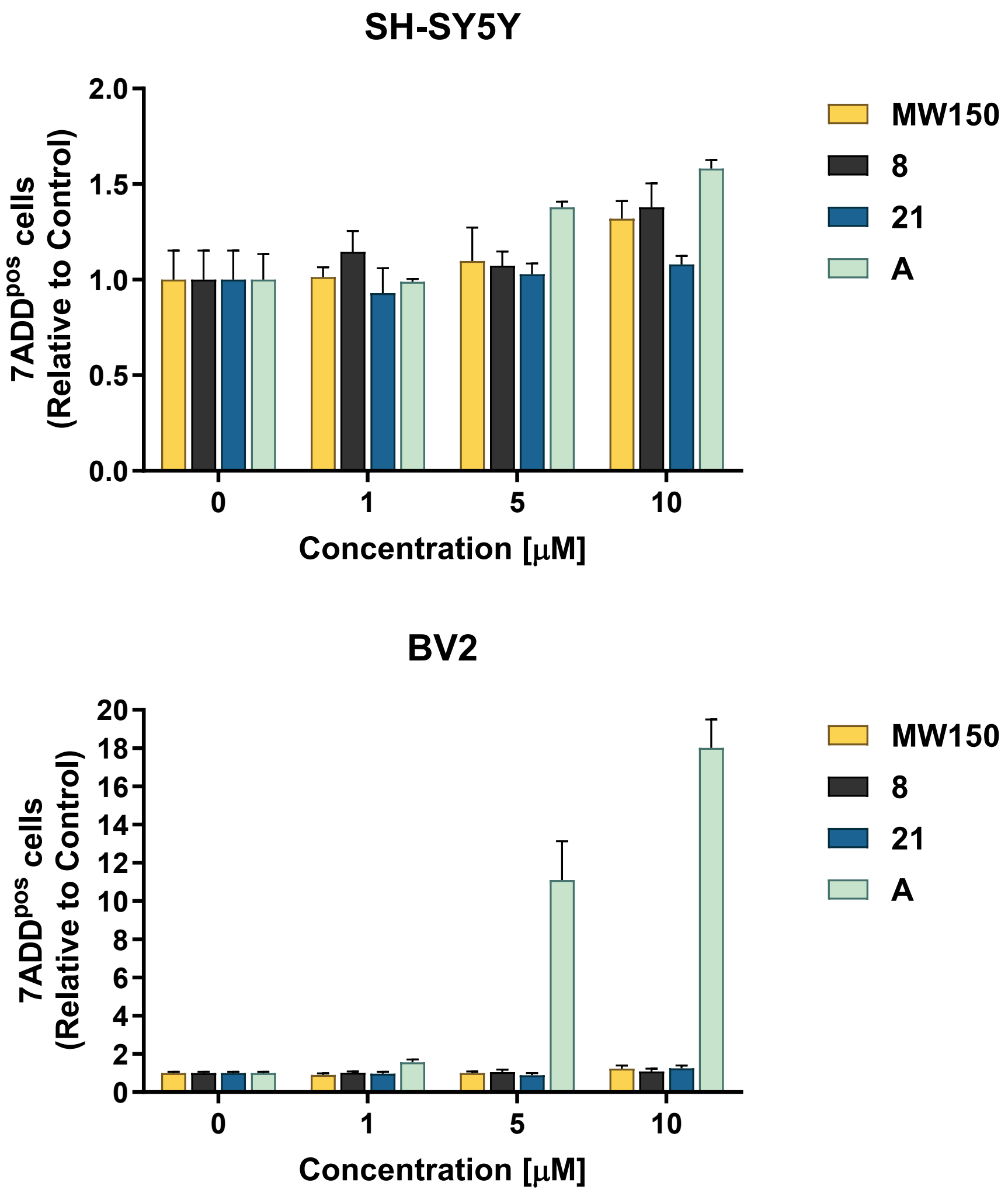

Figure S4. *In vitro* toxicity of **MW150**, **8**, **21**, and a selective hBChE inhibitor **A**. SH-SY5Y and BV2 cells were treated with increasing concentrations of the compounds (1–10 µM) in serum-free medium. After 48 h treatment, toxicity was evaluated by flow cytometry analysis of 7AAD staining. The graph displays the results of the quantitative analysis indicating the percentage of dead cells as a fraction of 7AAD positive cells ($7AAD^{pos}$). DMSO-treated cells were considered as control cells. Data are presented as means (± SEM) of at least three independent experiments, each carried out in quadruplicate.

1.6. *In Vivo* Assays

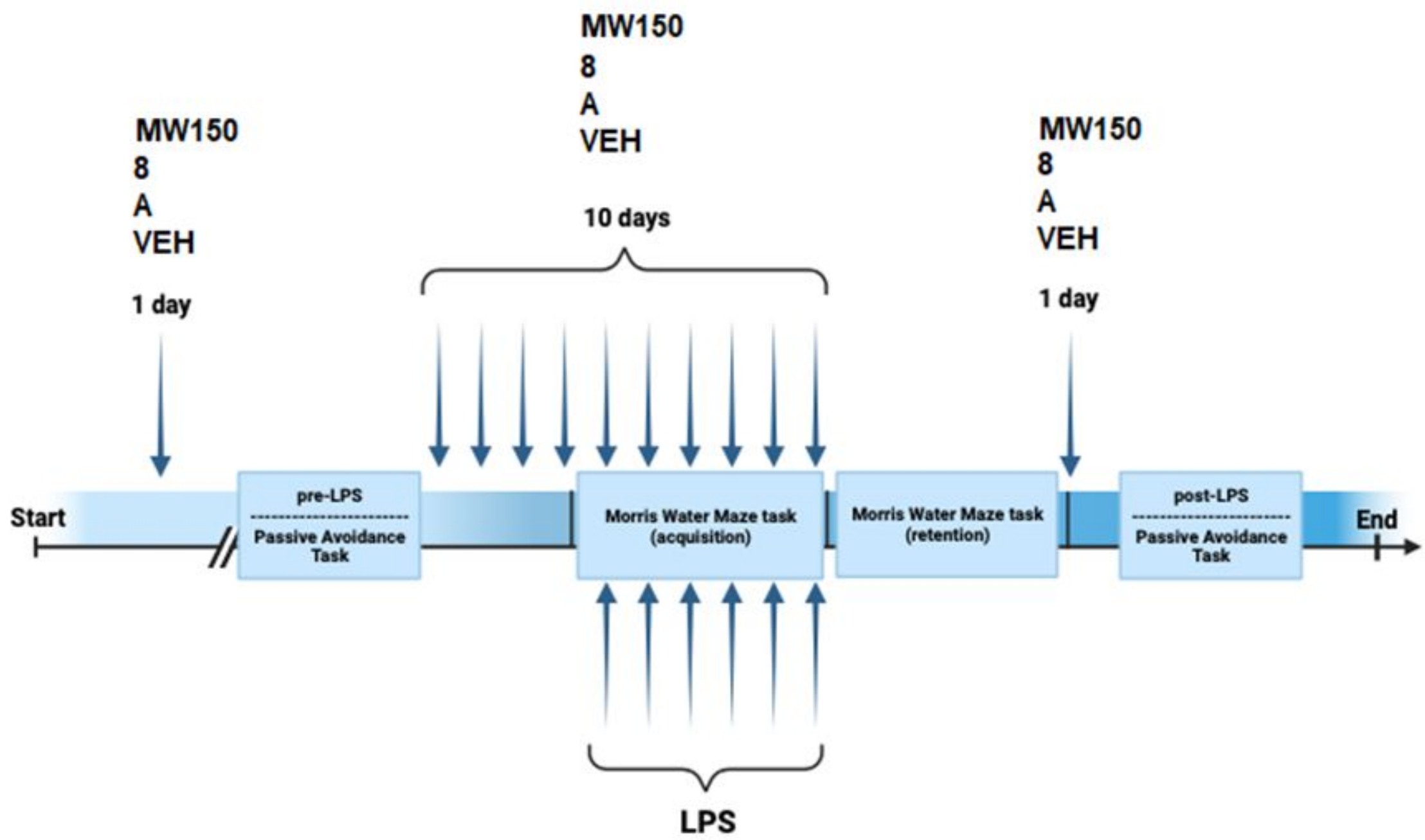

Figure S5. Experimental protocol used to test the activity of **MW150**, **8**, and the selective hBChE inhibitor **A** in the LPS mouse model. Created with BioRender.

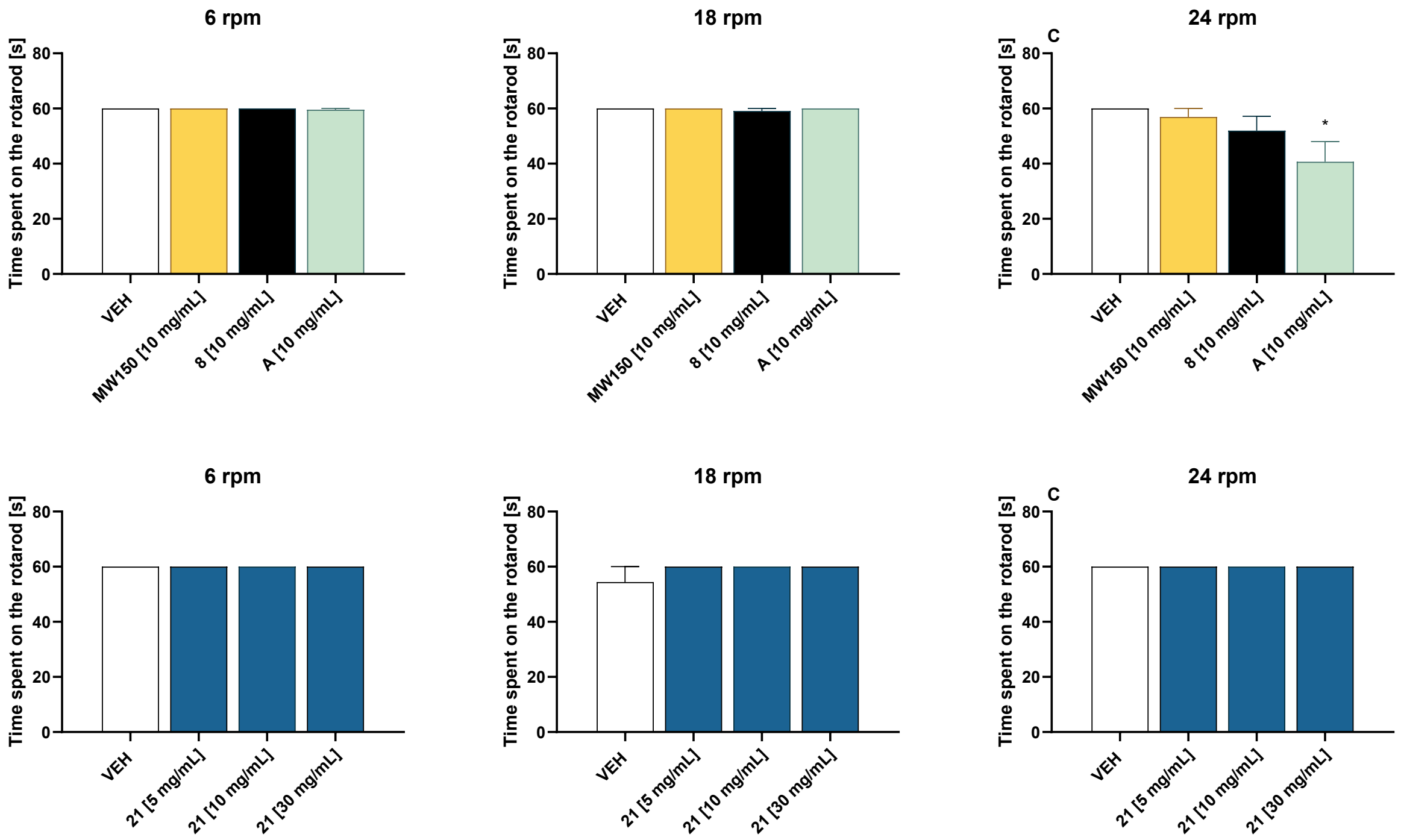

Figure S6. Effect of **MW150**, **8**, and **A** (10 mg/kg, i.p., top), and **21** (5, 10, and 30 mg/kg, i.p., bottom) on motor coordination measured using the rotarod test at 6 rpm (left), 18 rpm (middle), and 24 rpm (right). Results for n = 9–10 are shown as the mean time spent on the rotarod apparatus ± SEM and measured during the 1-minute (for each speed) test. Statistical analysis: one-way ANOVA followed by Dunnett's *post hoc* comparison. Significance *vs.* control (VEH): *$p < 0.05$.

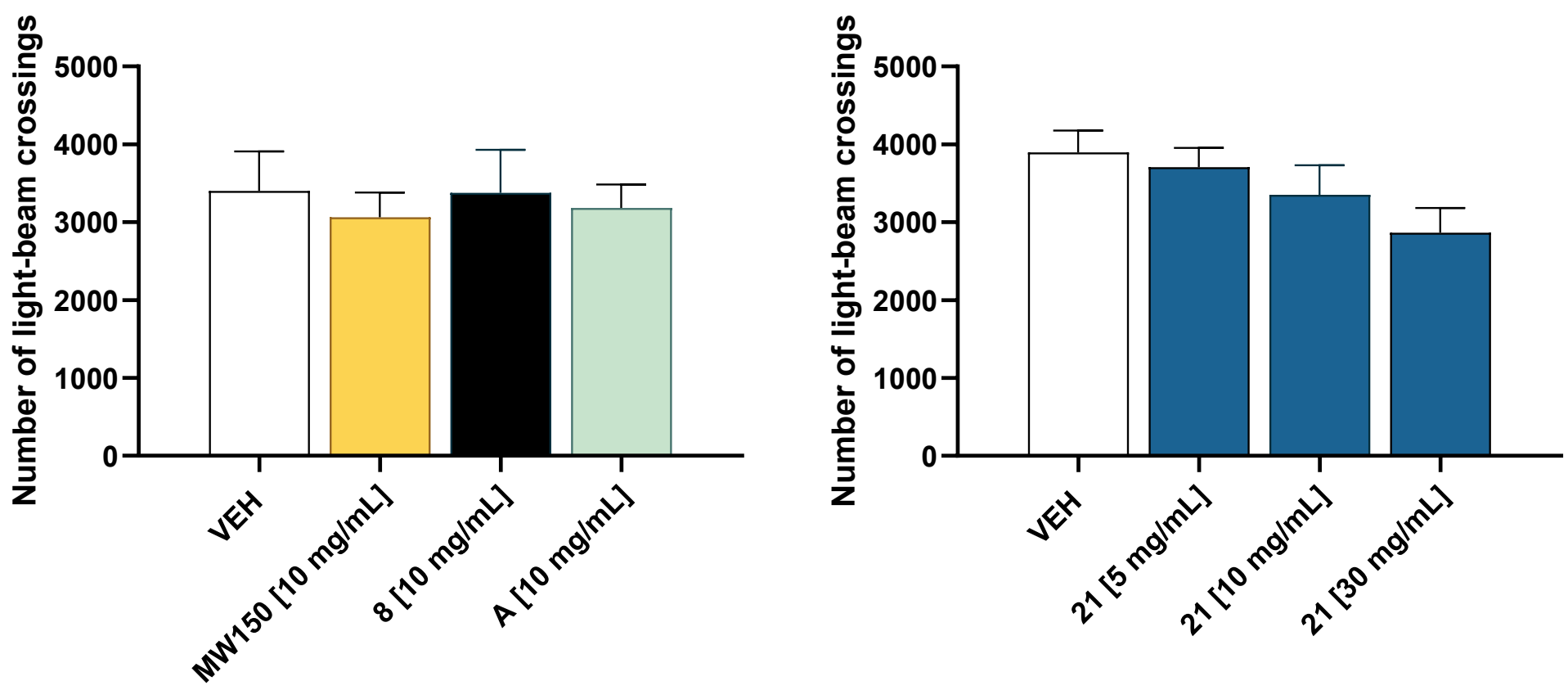

Figure S7. Effect of **MW150**, **8**, and **A** (10 mg/kg, i.p., left), and **21** (5, 10, and 30 mg/kg, i.p., right) on locomotor activity measured in mice. Results are shown as the mean number of light-beam crossings ± SEM measured during the 60-minute test for n = 9–10. Statistical analysis:

one-way ANOVA followed by Dunnett's *post hoc* comparison. Significance *vs.* control (VEH): $p > 0.05$.

LPS Model of Neuroinflammation – Additional Analysis of the PA Task Results

An additional analysis was performed within each experimental group for trials before LPS administration (T1 and T2) and after LPS administration (trials T3, T4) to determine whether a given treatment affected step-through latency within each group. In vehicle-treated, memory-unimpaired mice, there was a significant difference in step-through latency between trials T1 and T2 ($p < 0.01$) and between trials T3 and T4 ($p < 0.001$), which indicated good learning and memory skills in these mice (Figure S8). No significant differences were found between the T1 and T3 trials in control mice not treated with LPS, indicating that treatment with vehicle had no impact on learning skills measured in the PA task. In the LPS-treated control group, step-through latency in T2 was significantly increased compared to the T1 trial ($p < 0.05$). This group also showed a significant difference between post-LPS trials T3 and T4 ($p < 0.001$). However, in the LPS-treated control mice group, no significant difference was detected between the T1 (pre-LPS) and T3 (post-LPS) acquisition trials. This indicates that the treatment with vehicle and LPS had no impact on learning skills measured in the PA task. Of note, T3 and T4 trials of the PA task were performed on days 8 and 9 after the first dose of LPS (Figure S5). As mentioned earlier (please refer to the main text), in the experiment of series 1, no significant behavioral changes were observed in mice at this stage of testing.

In mice treated with a single dose of selective hBChE inhibitor **A** before LPS administration, step-through latency in T2 was significantly increased compared to T1 ($p < 0.01$). A significant difference was also observed between T3 and T4 after LPS administration ($p < 0.05$). In contrast to both vehicle-treated groups, repeated administration of the selective hBChE inhibitor **A** significantly increased step-through latency in the T3 (post-LPS acquisition) trial compared to the T1 (pre-LPS acquisition) trial ($p < 0.0001$) (Figure S8). Similarly, **MW150** and **8** – repeated administrations significantly increased T3 (post-LPS acquisition) trial step-through latency as compared to that of T1 (pre-LPS acquisition) trial ($p < 0.0001$) (Figure S8), which indicated that 10-day treatment with **MW150** and **8** positively influenced and enhanced the formation of long-term memory as this difference between T1 and T3 step-through latency was not observed in any control group.

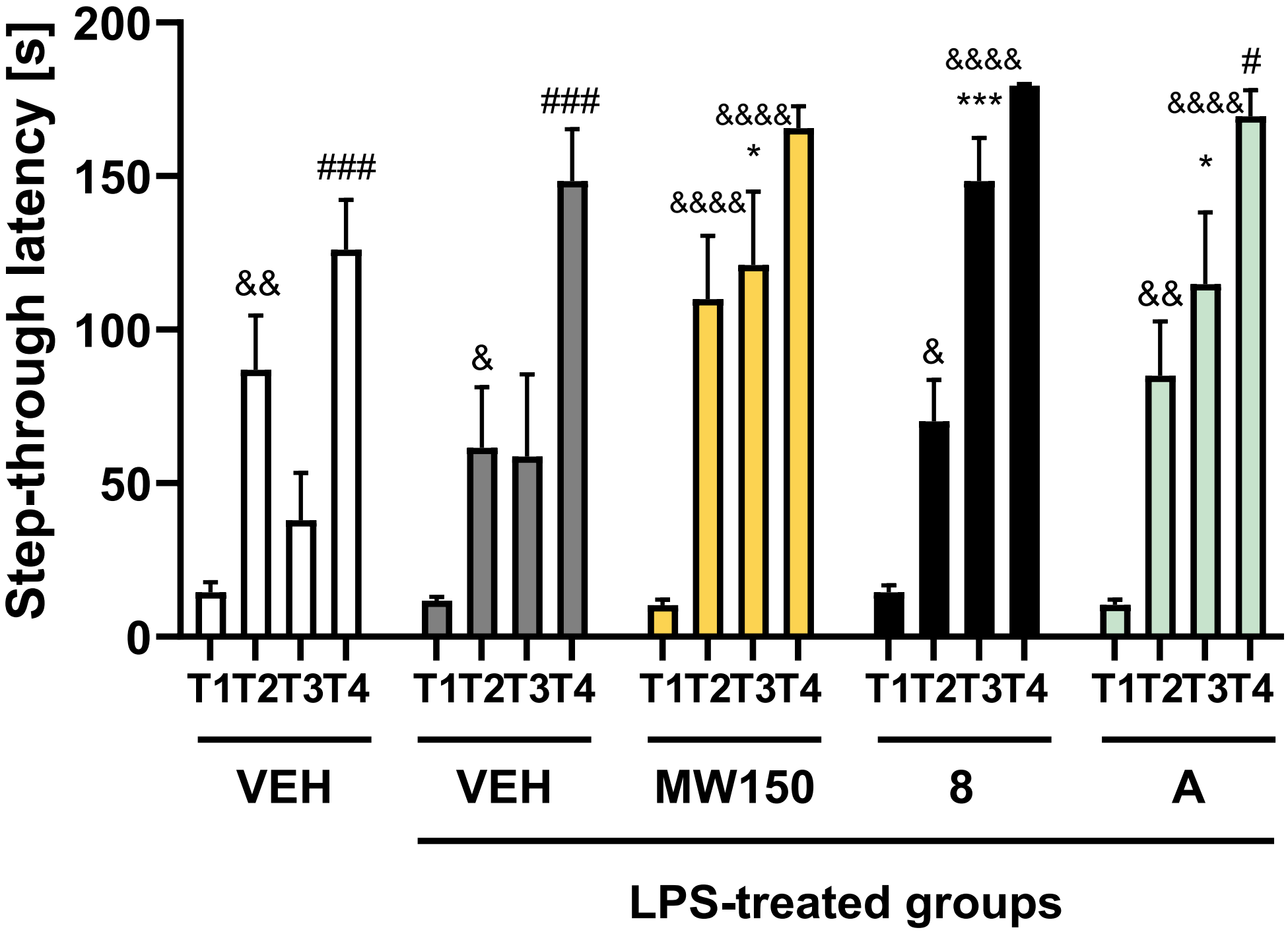

Figure S8. Effects of a selective p38α MAPK ligand **MW150**, dual hBChE/p38α MAPK inhibitor **8**, and a selective hBChE inhibitor **A** (5 mg/kg, i.p.) on fear-motivated contextual learning and memory measured using the passive avoidance (PA) task in mice not exposed to LPS (T1, T2 trials) and in mice exposed to LPS (T3 and T4 trials). A detailed protocol for drug administration is shown in Figure S5. Results are shown as the mean step-through latency (± SEM) assessed in the acquisition trials (T1, T3) and 24 h later in the retention trials (T2, T4) for n = 8–10 mice. Statistical analysis: repeated measures ANOVA followed by Dunnett's and Tukey's *post hoc* comparisons. Significance *vs.* LPS-treated control mice in the respective trial: $^{*}p < 0.05$, $^{***}p < 0.001$; significance in the individual group *vs.* T1 step-through latency: $^{\&}p < 0.05$, $^{\&\&}p < 0.01$, $^{\&\&\&\&}p < 0.0001$ and significance in the individual group *vs.* T3 step-through latency: $^{\#}p < 0.05$, $^{\#\#\#}p < 0.001$.

Western blot analysis

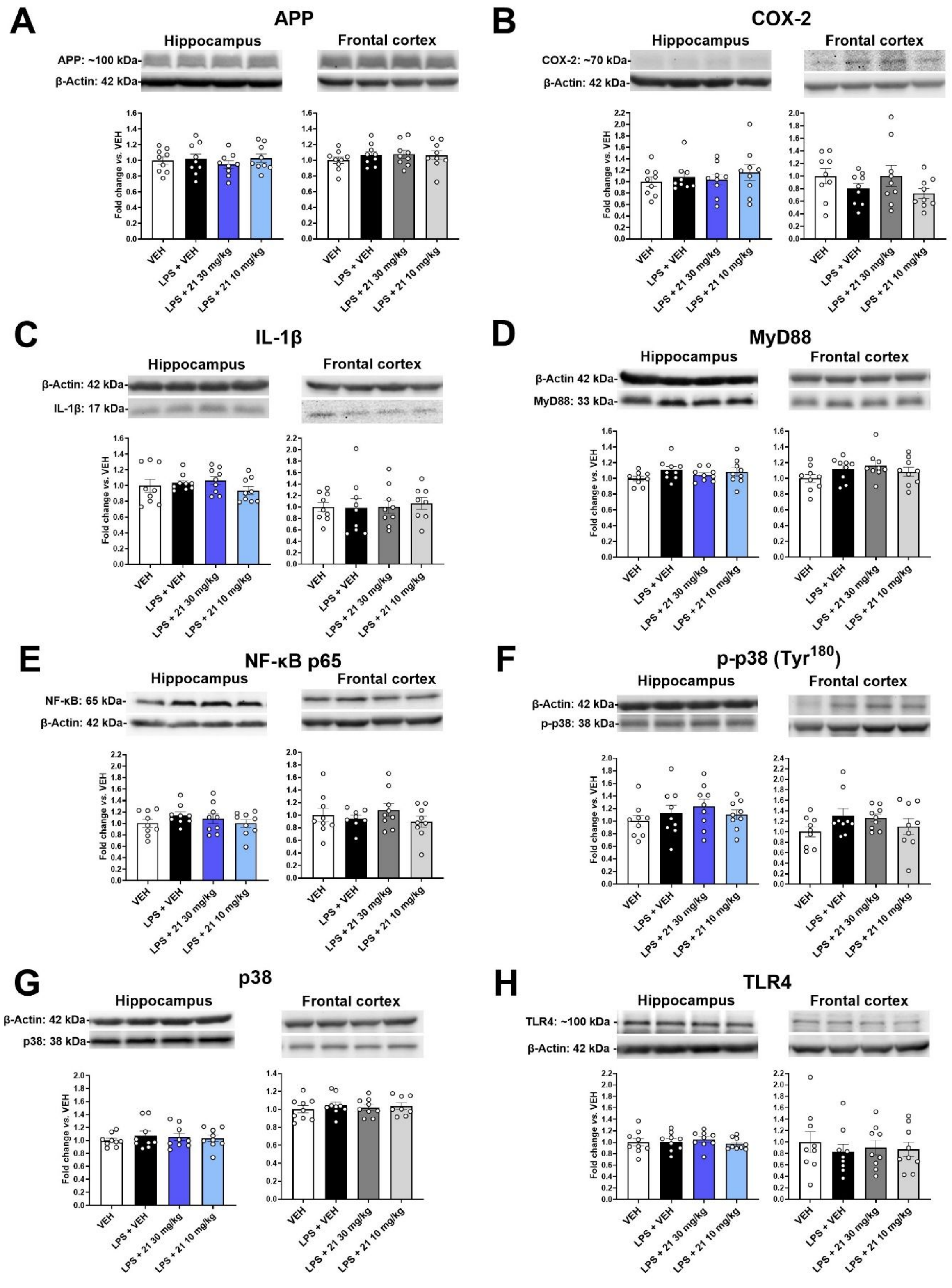

Figure S9. Results of Western blot analysis of neuroinflammatory markers in the hippocampus and frontal cortex of male C57/Bl/6j mice after chronic treatment with dual hBChE/p38α

MAPK inhibitor **21** (at daily doses of 30 mg/kg and 10 mg/kg, i.p.) and LPS (administered at cumulative dose of 12 mg/kg, i.p.). The levels of amyloid precursor protein (APP) (**A**), cyclooxygenase-2 (COX-2) (**B**), interleukin-1β (IL-1β) (**C**), myeloid differentiation primary response 88 adapter protein (MyD88) (**D**), nuclear factor kappa-light-chain-enhancer of activated B cells p65 (NF-κB p65) (**E**), phosphorylated (Tyr^{180}) form and total mitogen activated protein kinase p38 (p-p38 Tyr^{180} and p38) (**F** and **G**) and Toll like receptor 4 (TLR4) (**H**) were assayed 48 hours after last administration of LPS and **21**. Results are expressed as fold change *vs.* VEH group and presented as means ± SEM (n = 8–9 animals per group).

2. NMR spectra

N-Methoxy-*N*-methyl-2-naphthamide **1a**

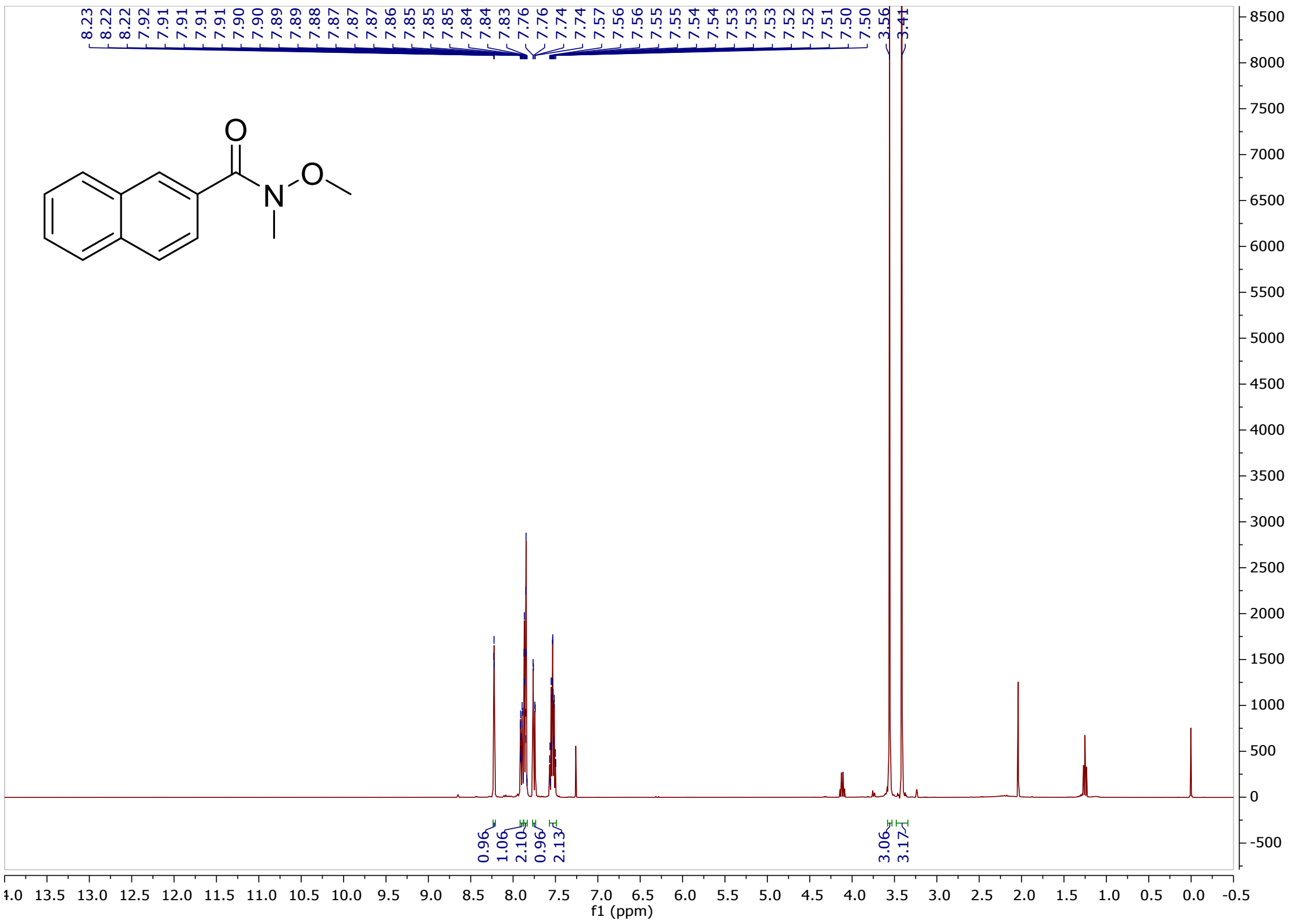

1-(Naphthalen-2-yl)-2-(pyridin-4-yl)ethan-1-one **2a**

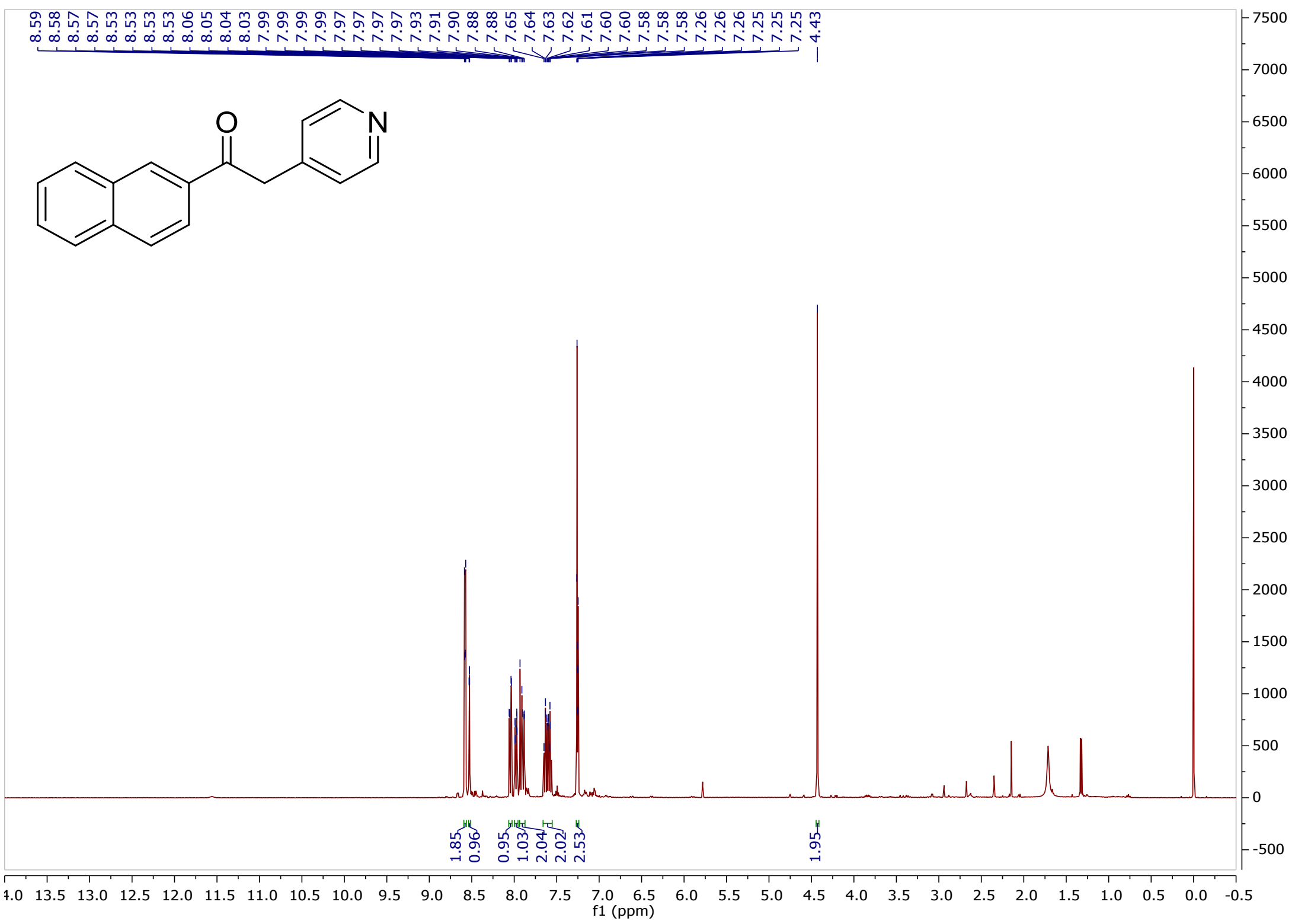

Ethyl 4-(naphthalen-2-yl)-4-oxo-3-(pyridin-4-yl)butanoate **3a**

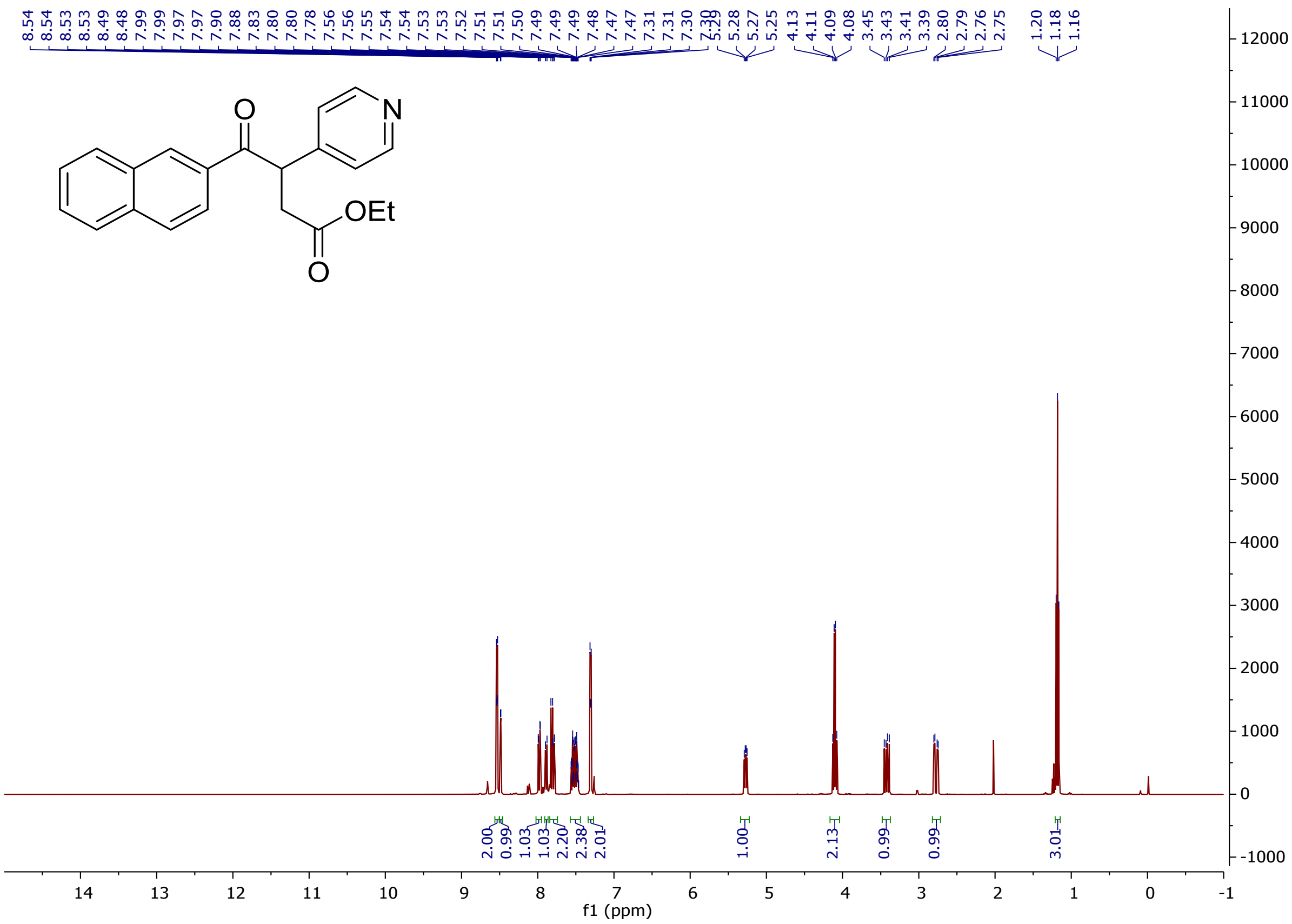

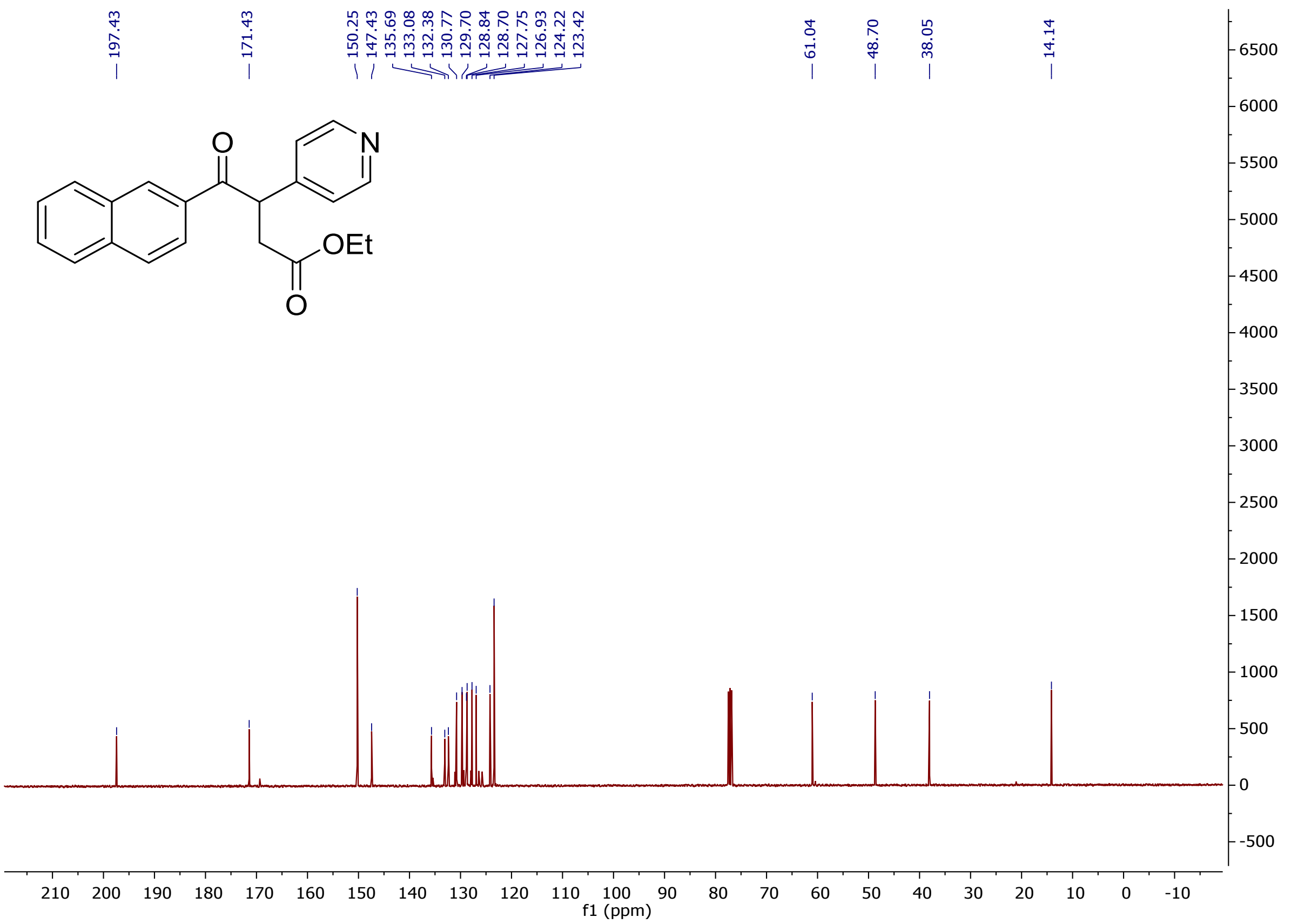

6-(Naphthalen-2-yl)-5-(pyridin-4-yl)-4,5-dihydropyridazin-3(2*H*)-one **4a**

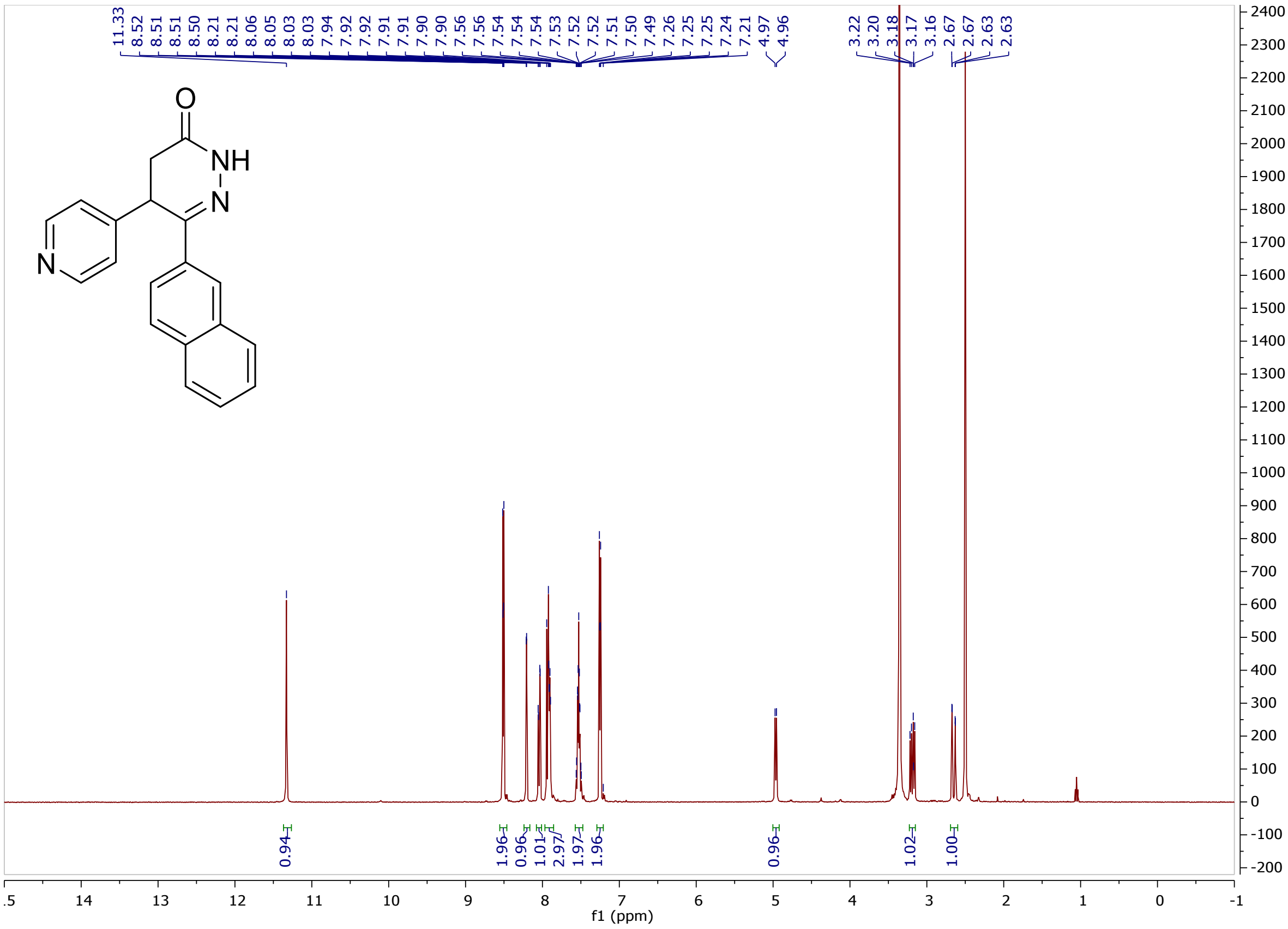

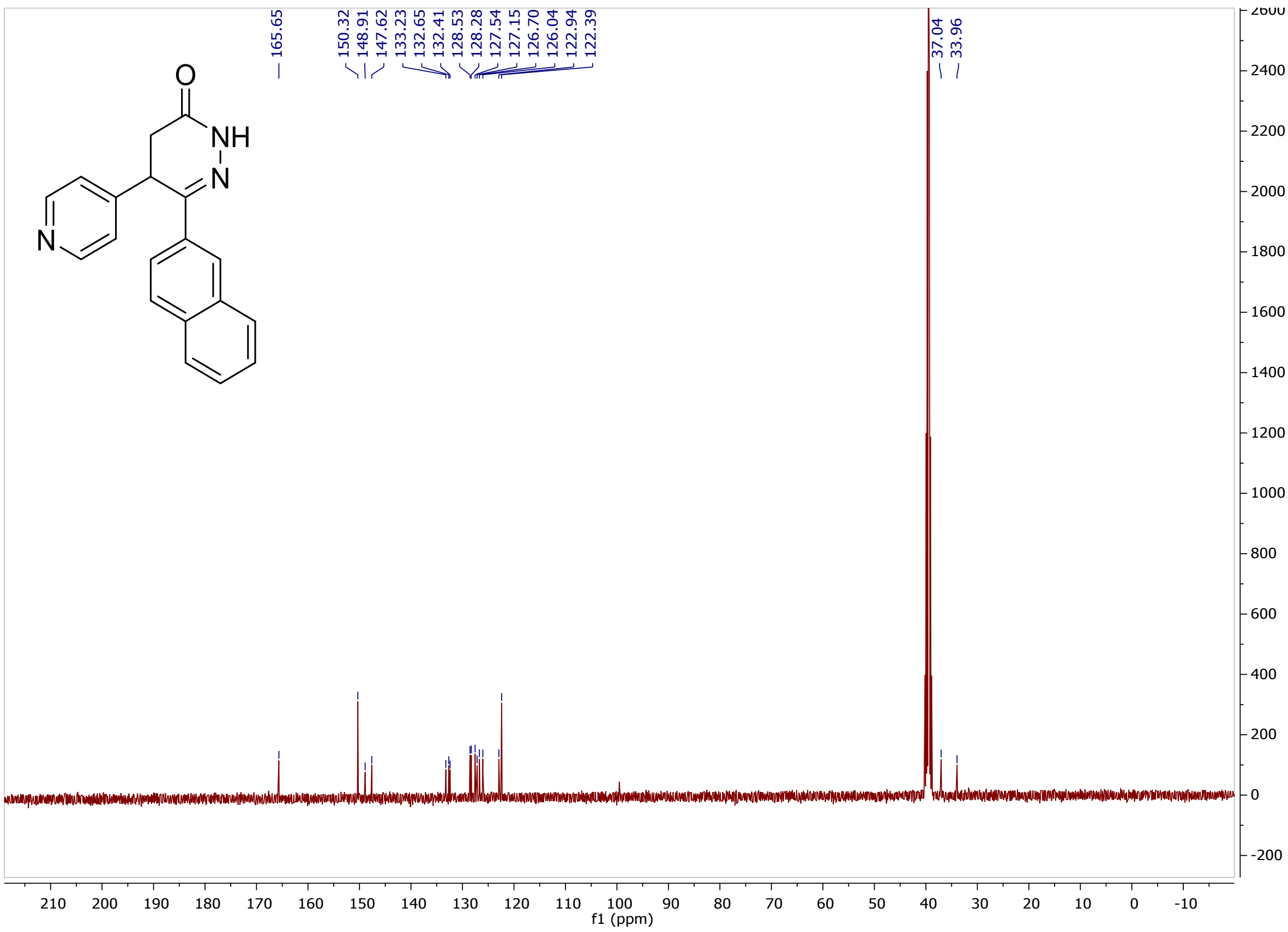

6-(Naphthalen-2-yl)-5-(pyridin-4-yl)pyridazin-3(2*H*)-one **5ai**

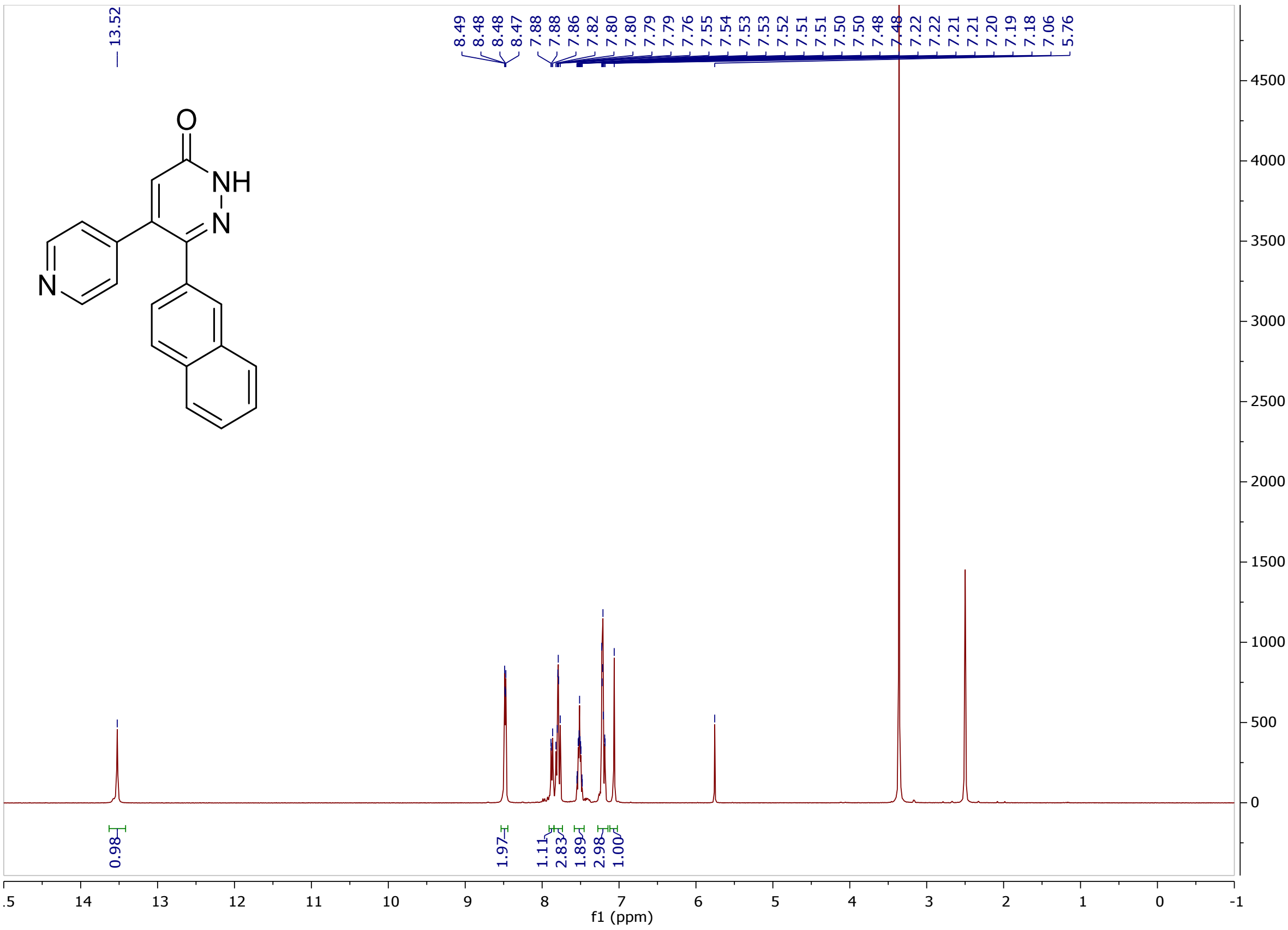

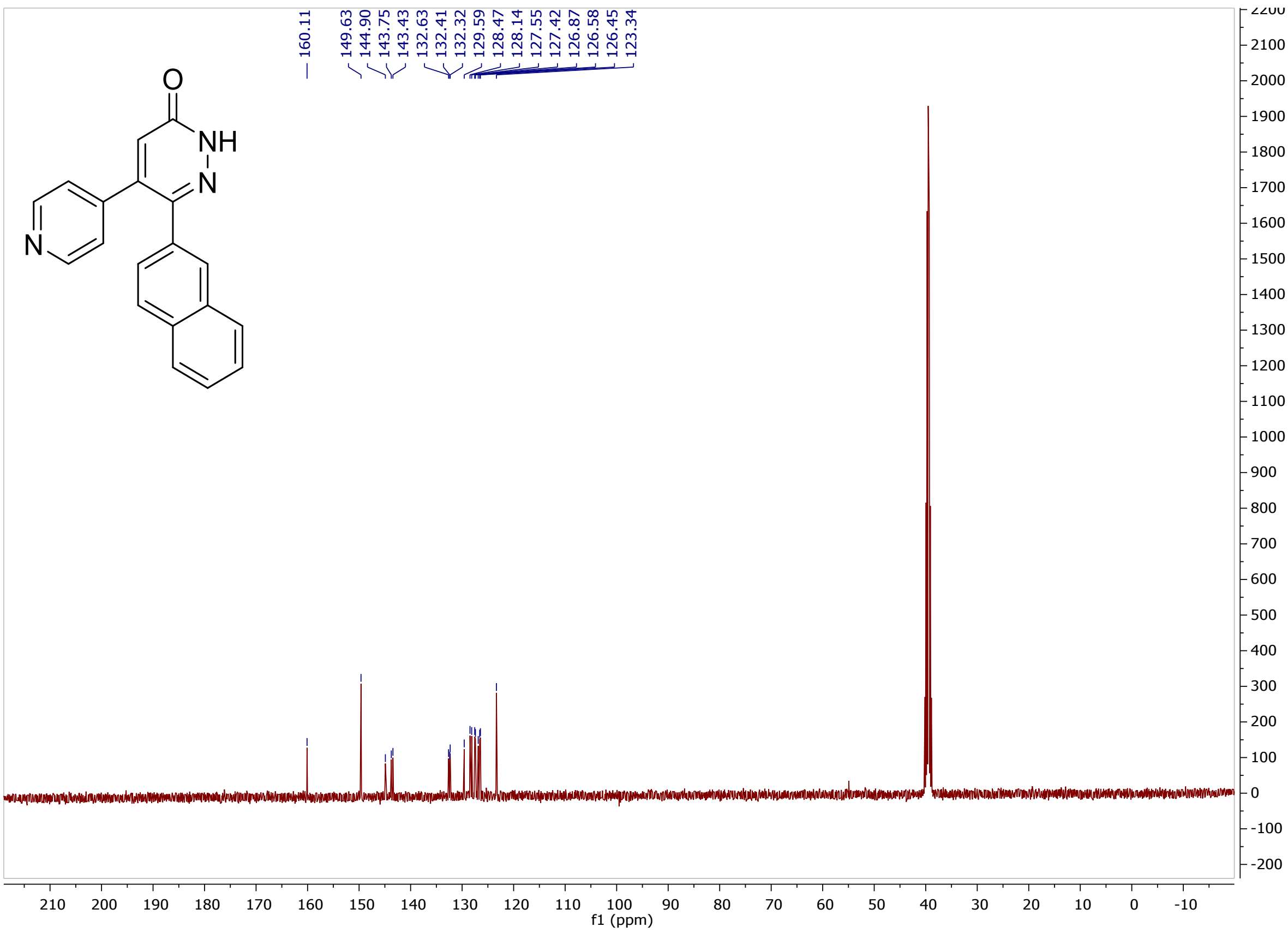

6-Chloro-3-(naphthalen-2-yl)-4-(pyridin-4-yl)pyridazine **5a**

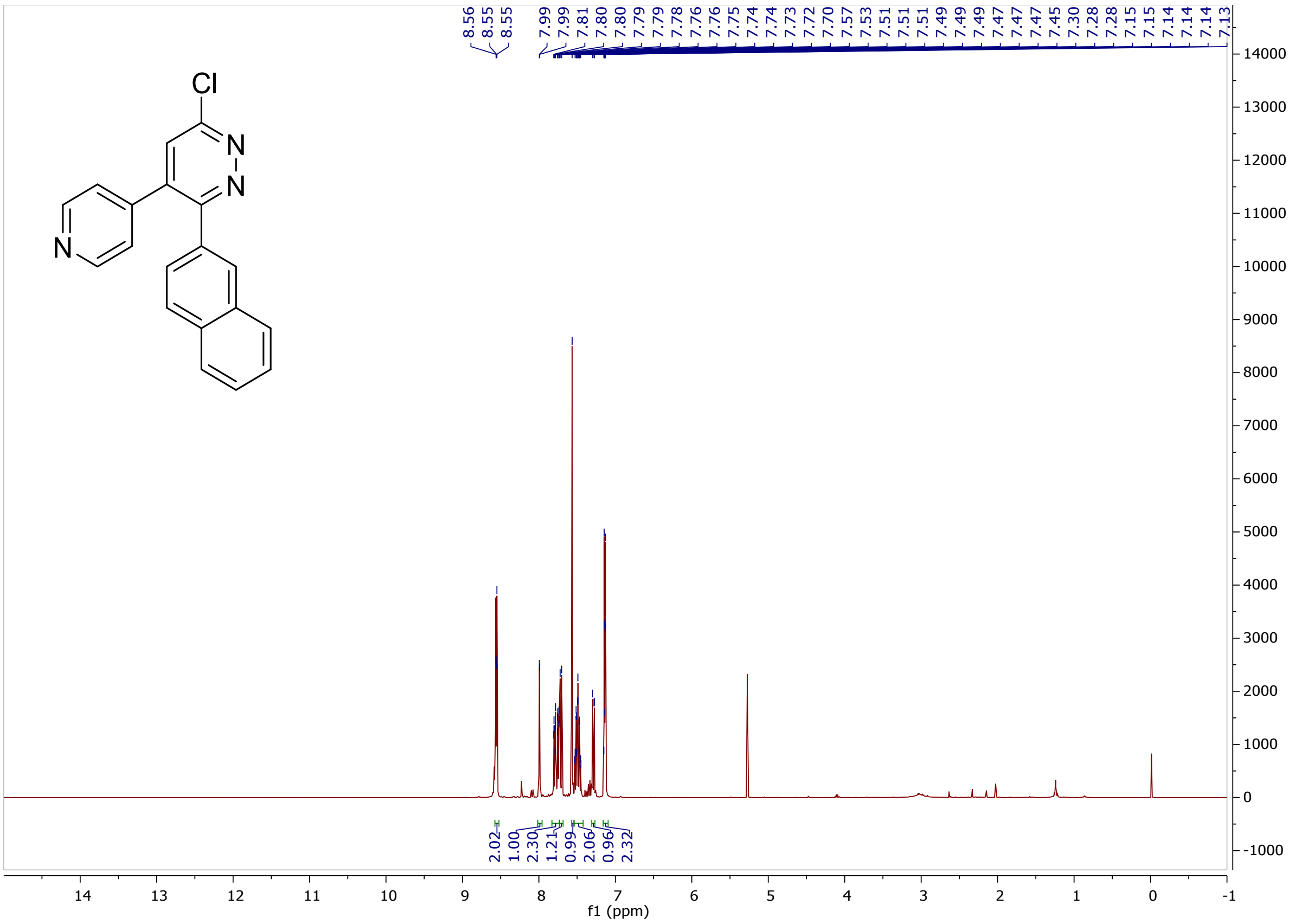

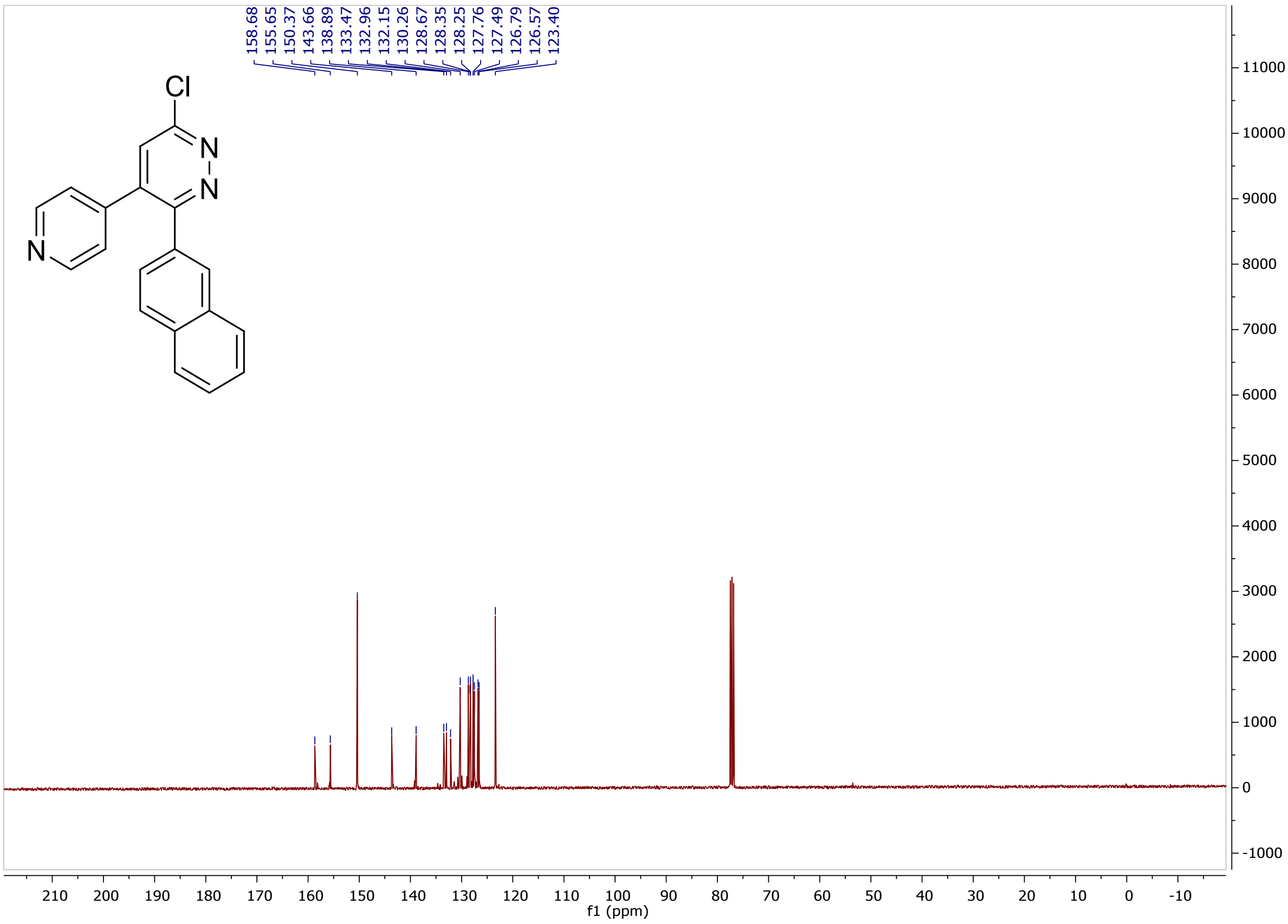

N-Methoxy-*N*-methyl-1-naphthamide **1b**

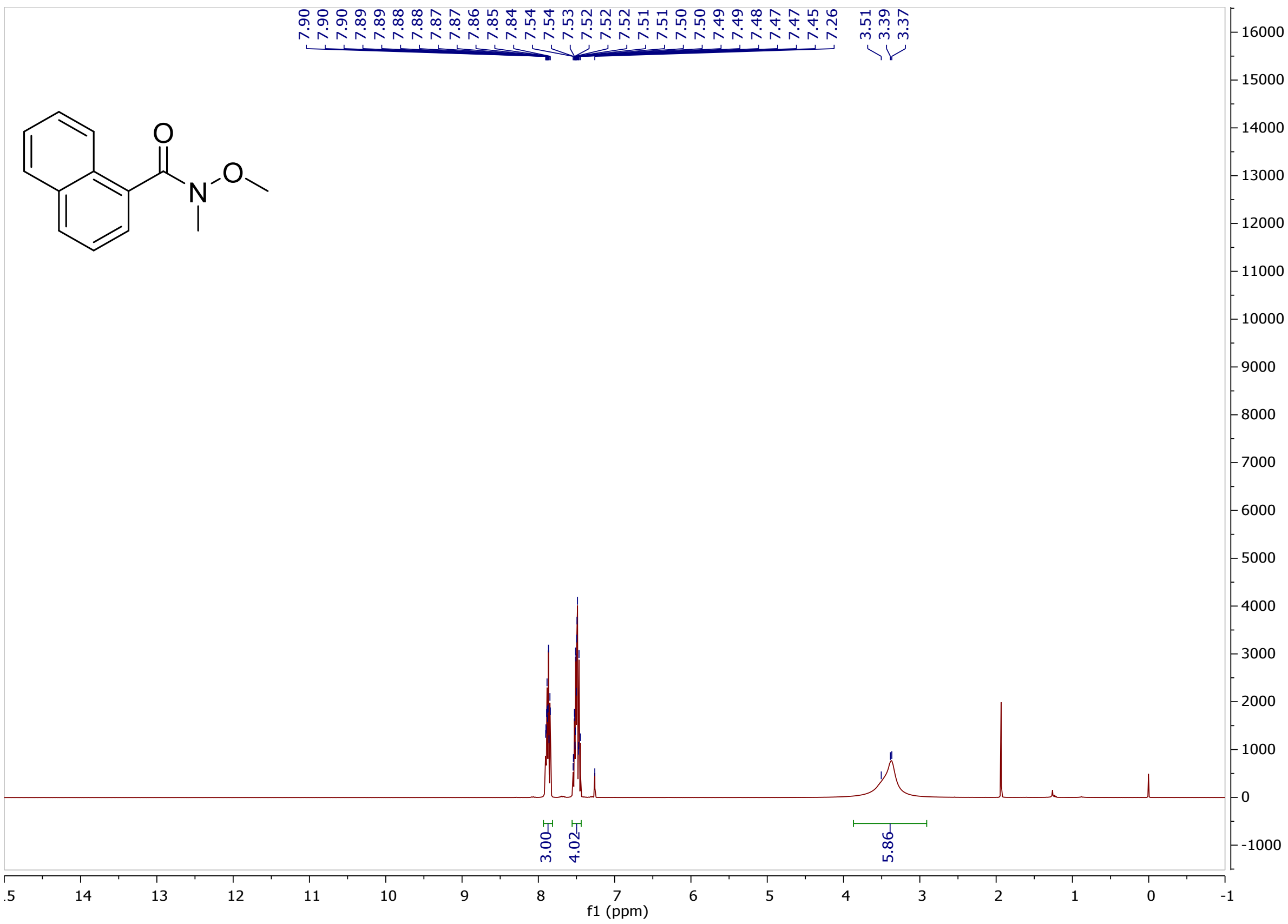

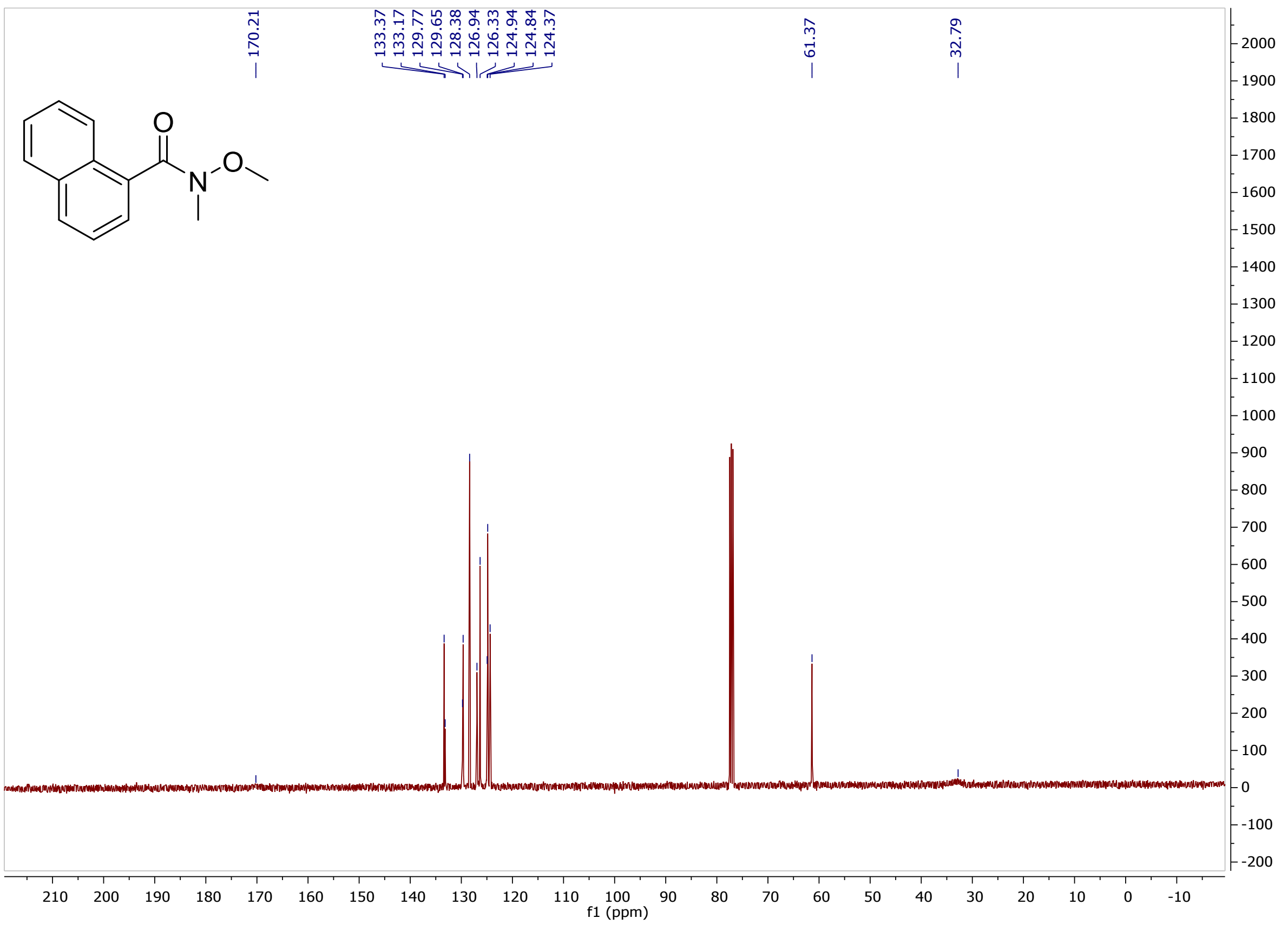

1-(Naphthalen-1-yl)-2-(pyridin-4-yl)ethan-1-one **2b**

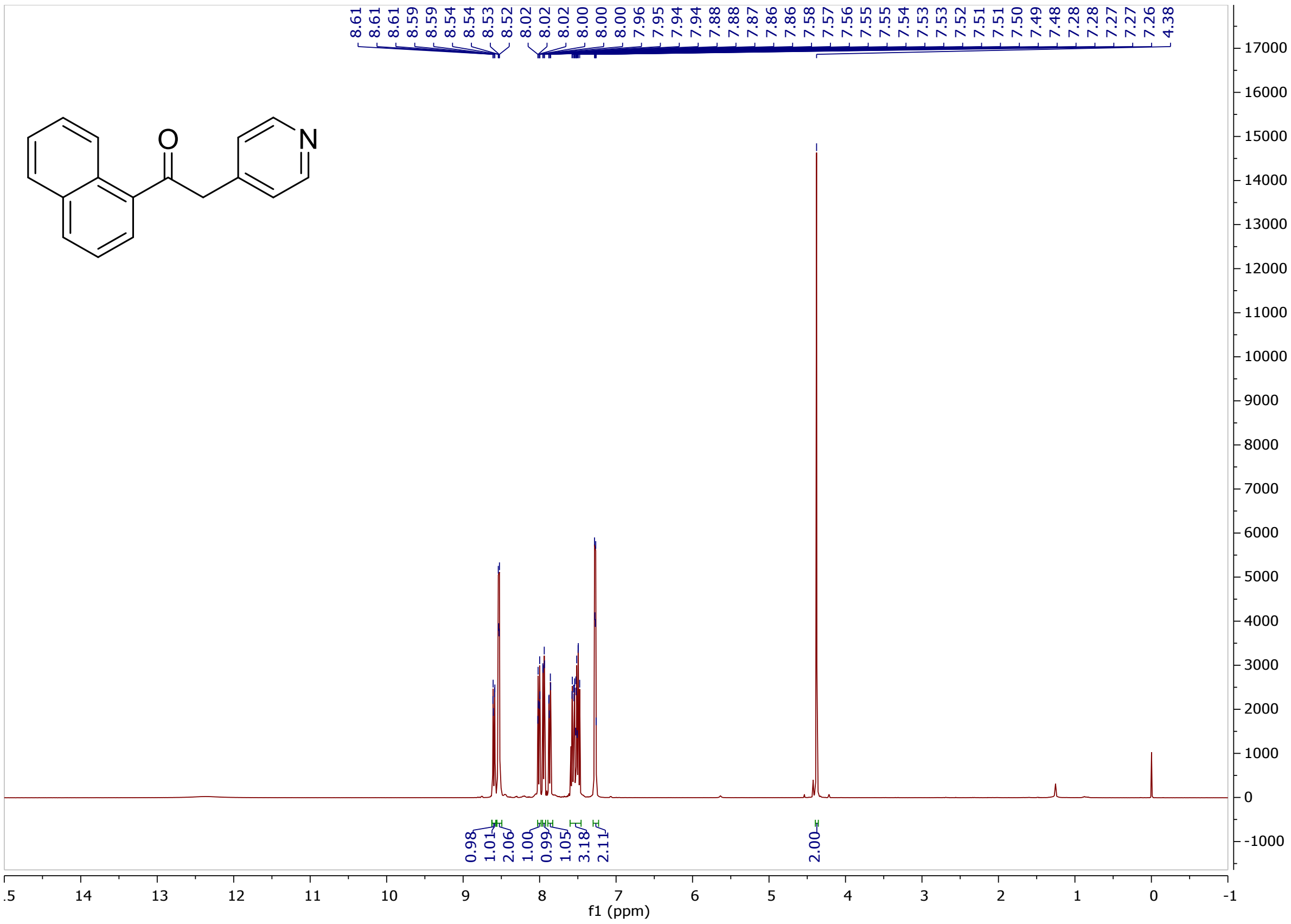

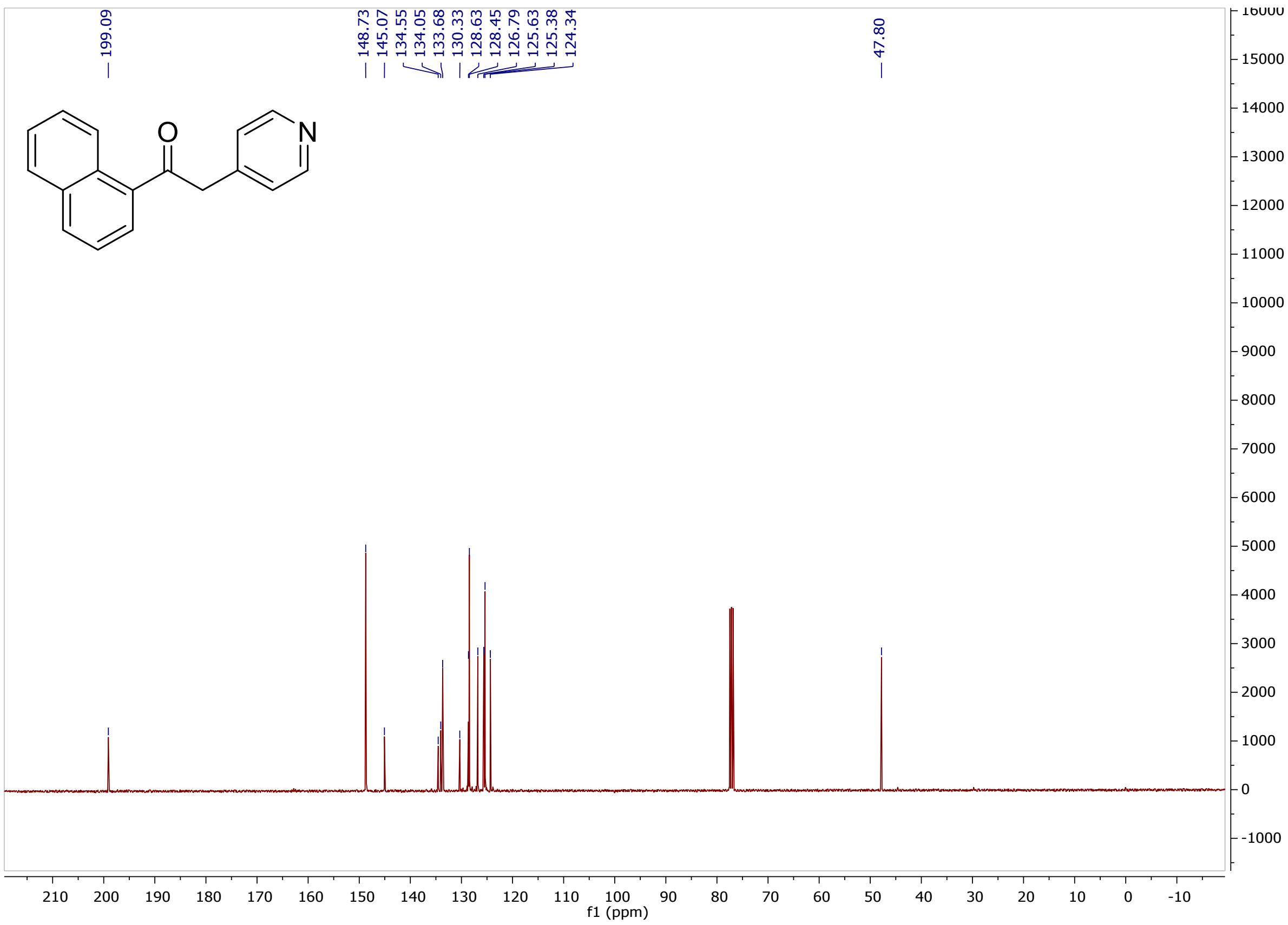

Ethyl 4-(naphthalen-1-yl)-4-oxo-3-(pyridin-4-yl)butanoate **3b**

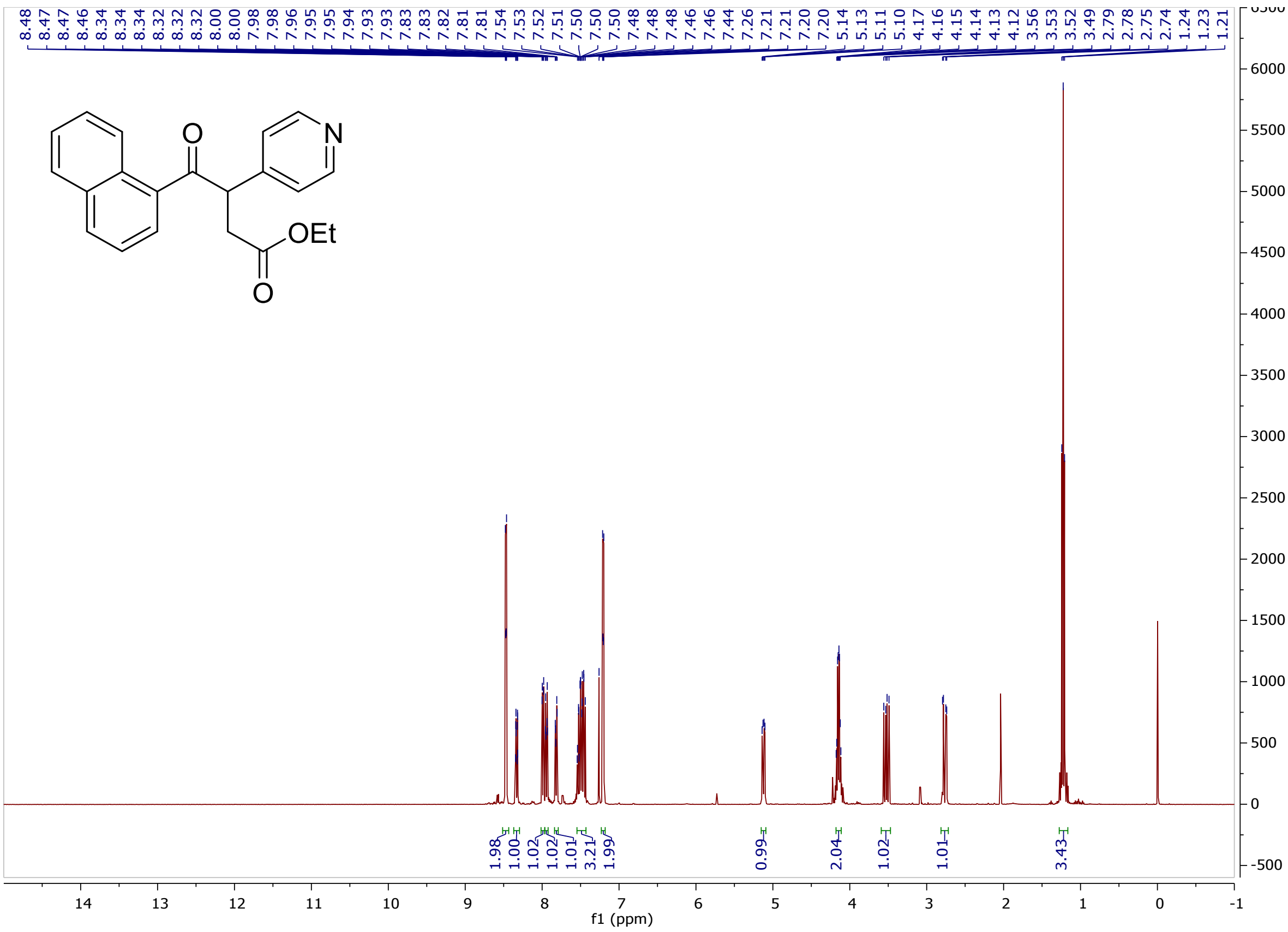

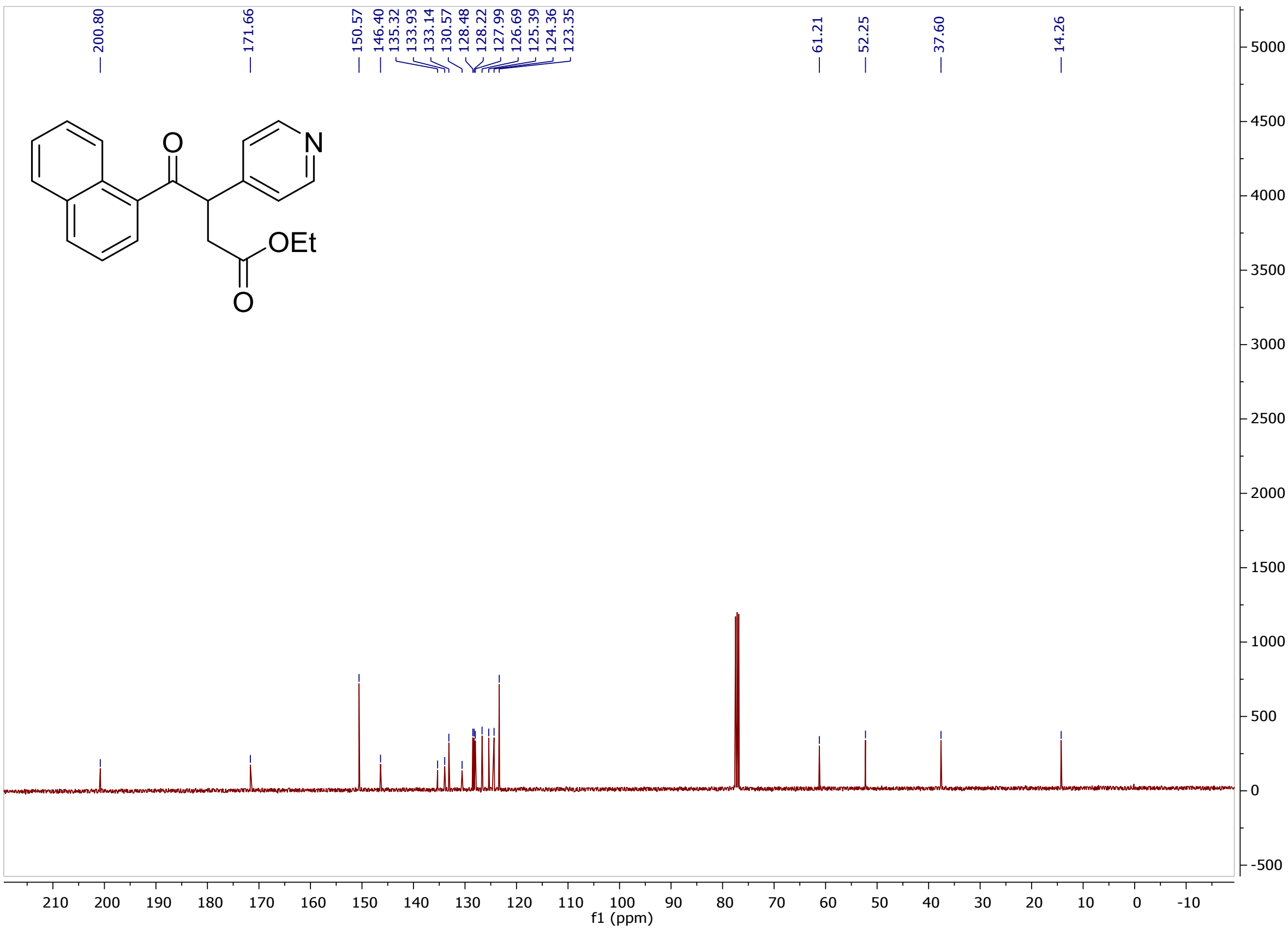

Side product: Ethyl 2-(4-(2-(naphthalen-1-yl)-2-oxoethylidene)pyridin-1(4*H*)-yl)acetate **3d**

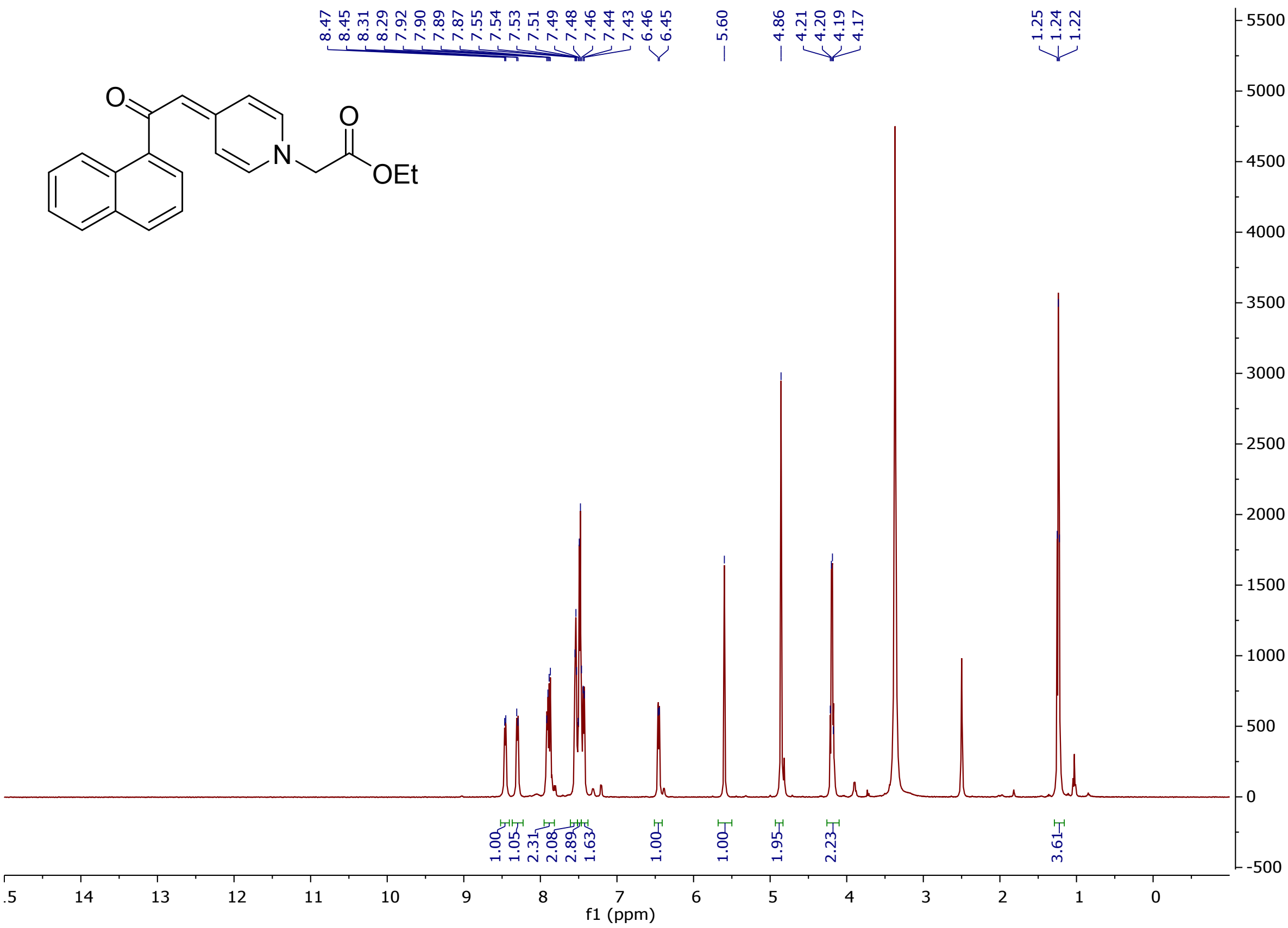

6-(Naphthalen-1-yl)-5-(pyridin-4-yl)pyridazin-3(2*H*)-one **5bi**

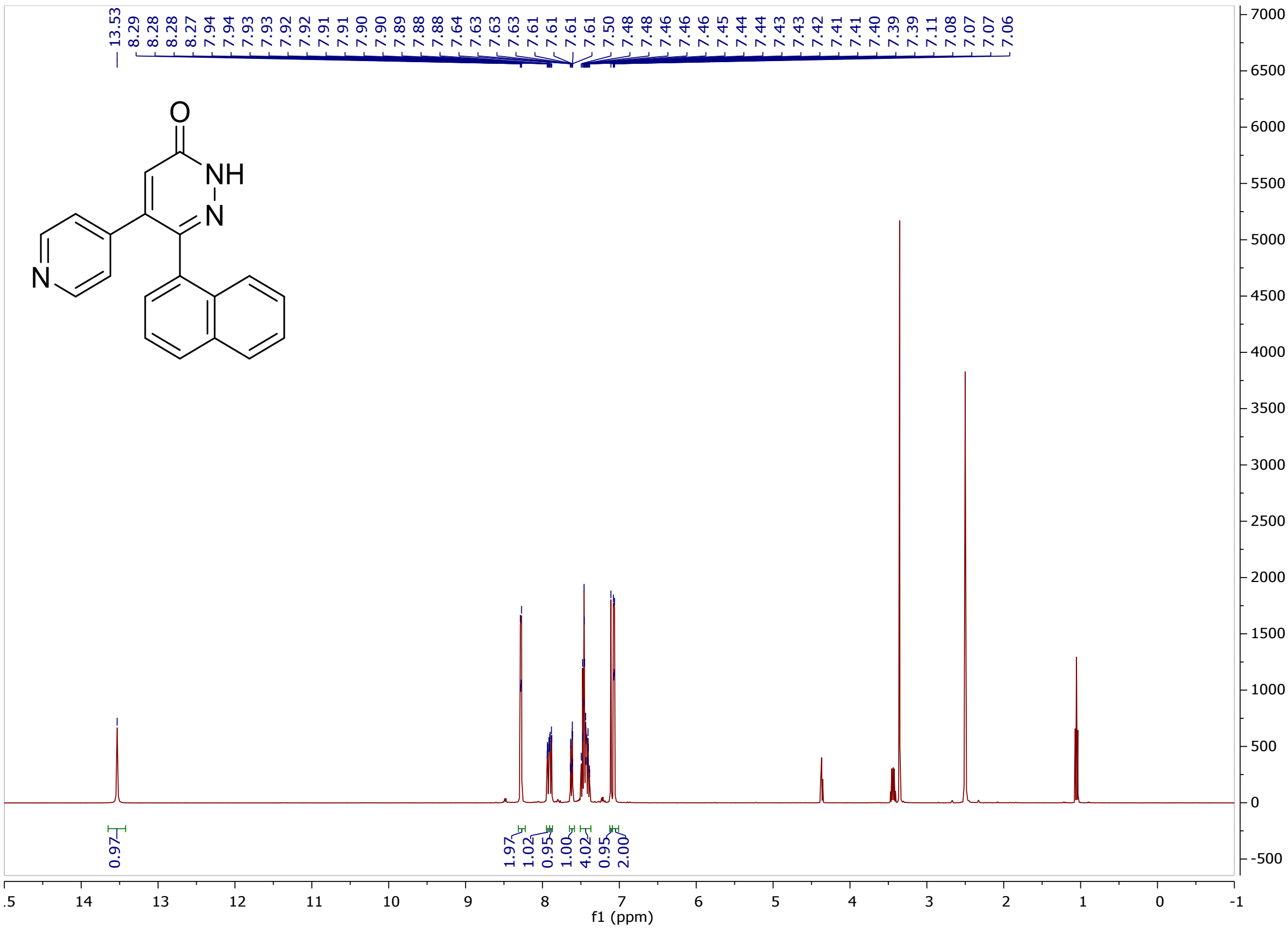

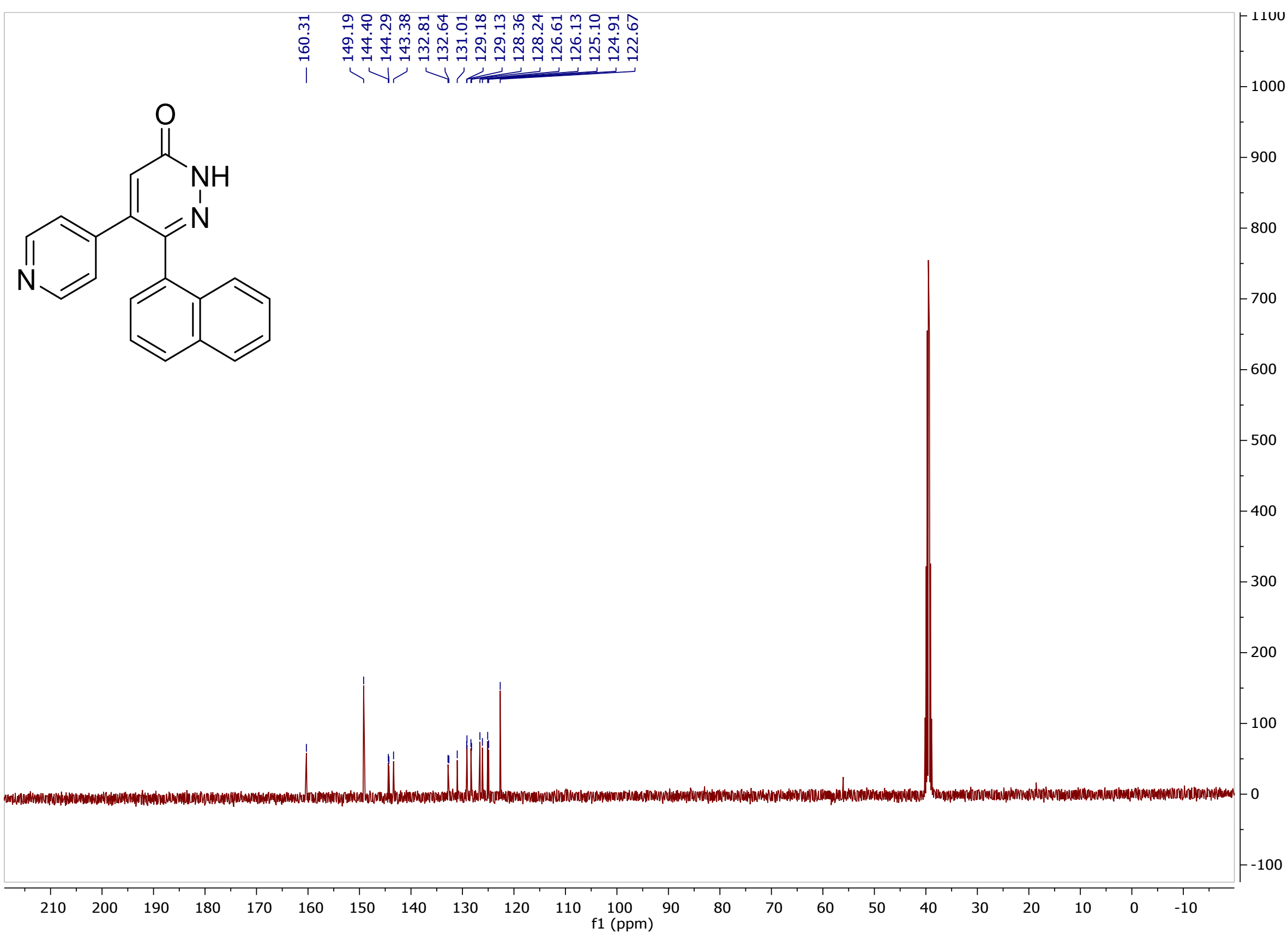

6-Chloro-3-(naphthalen-2-yl)-4-(pyridin-4-yl)pyridazine **5b**

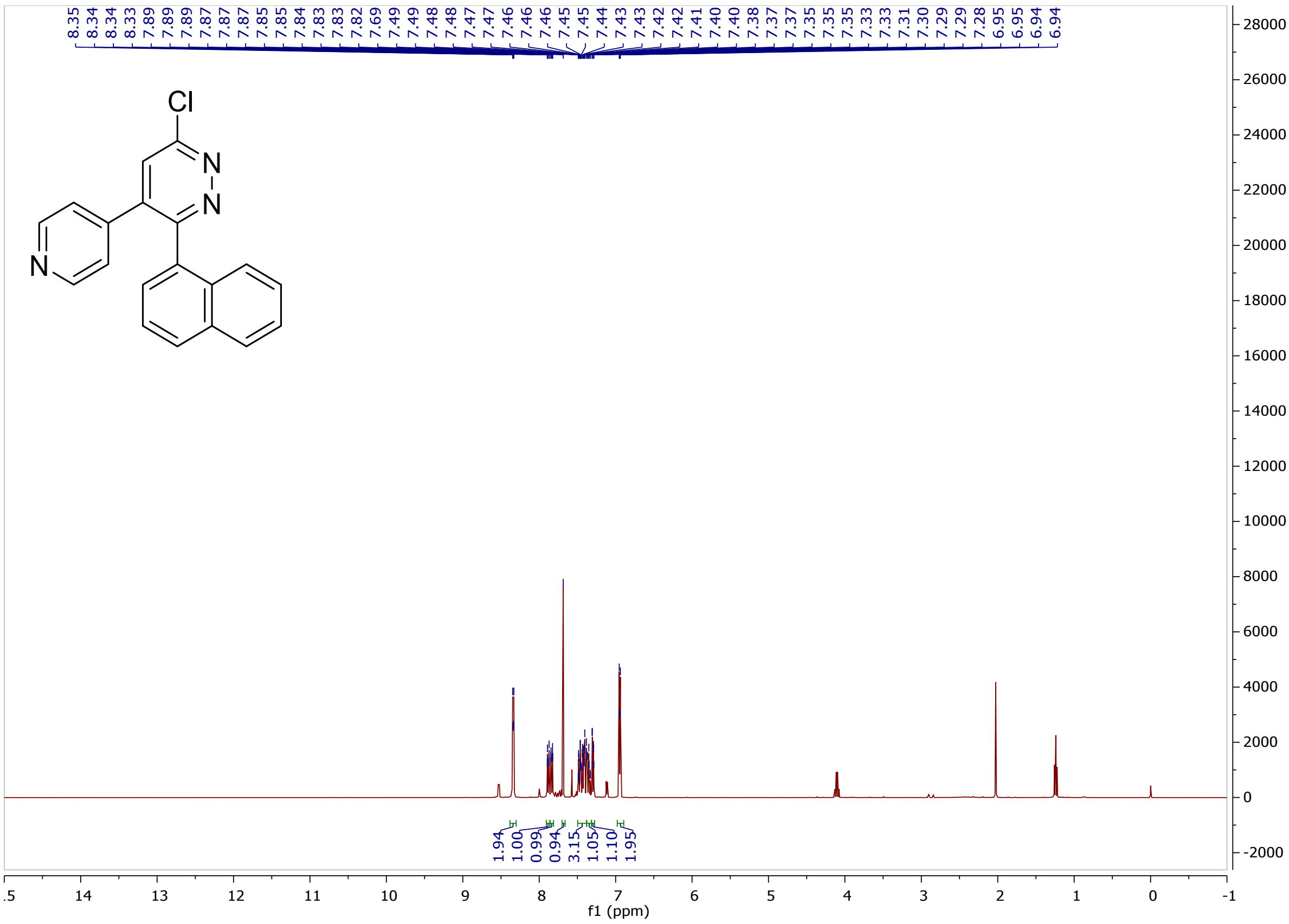

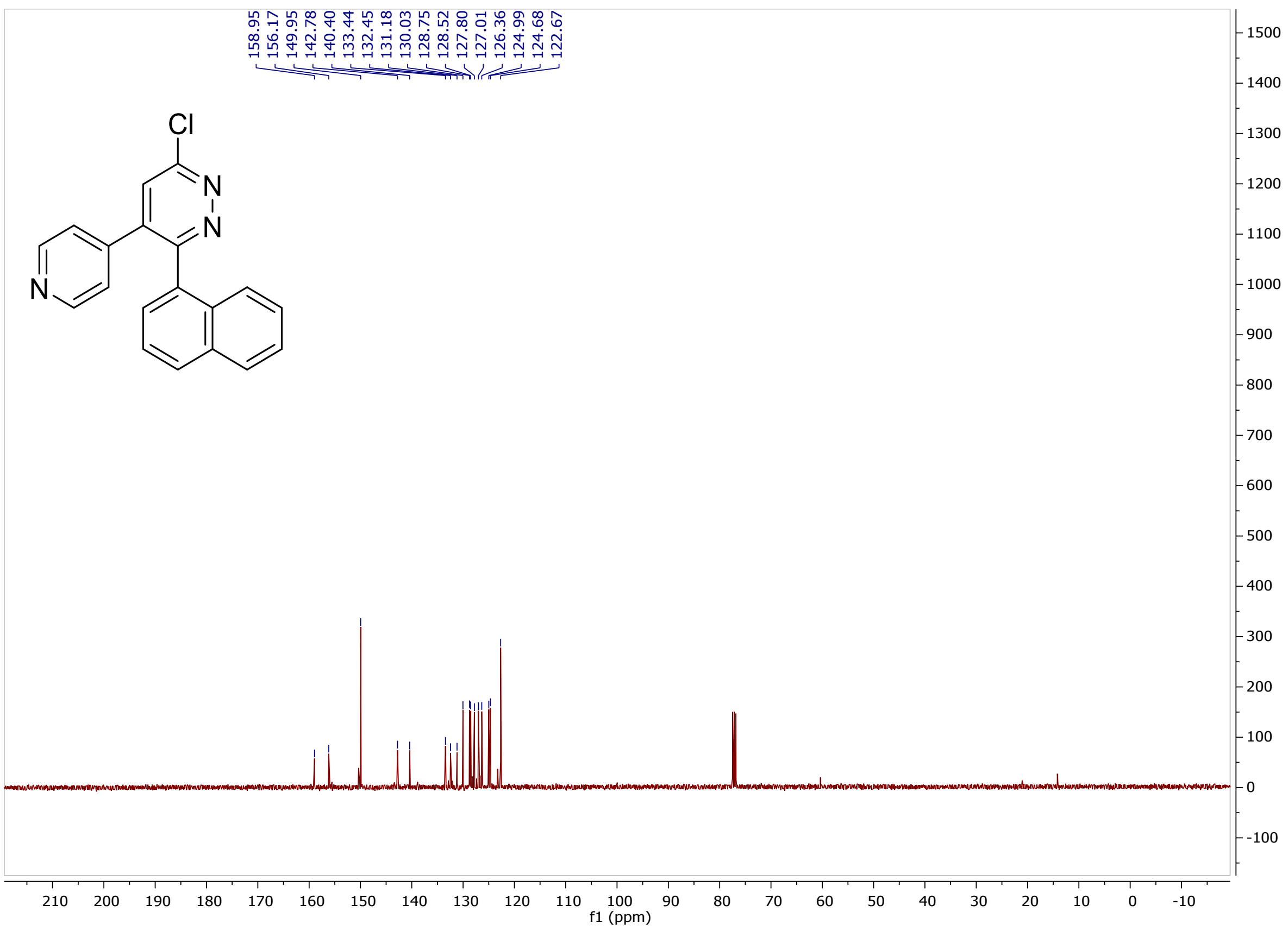

6-(4-Methylpiperazin-1-yl)-3-(naphthalen-2-yl)-4-(pyridin-4-yl)pyridazine (**6**) **MW150**

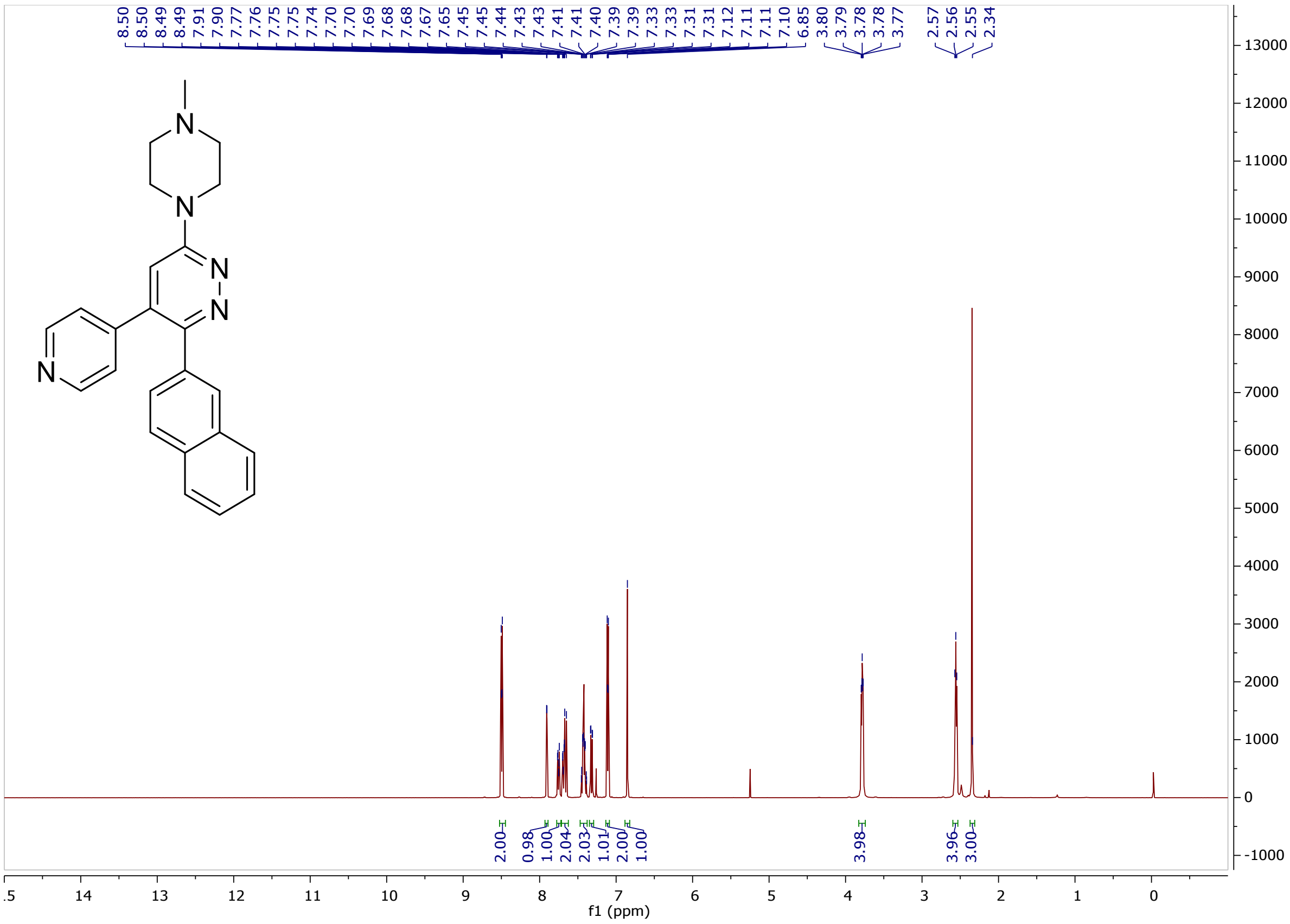

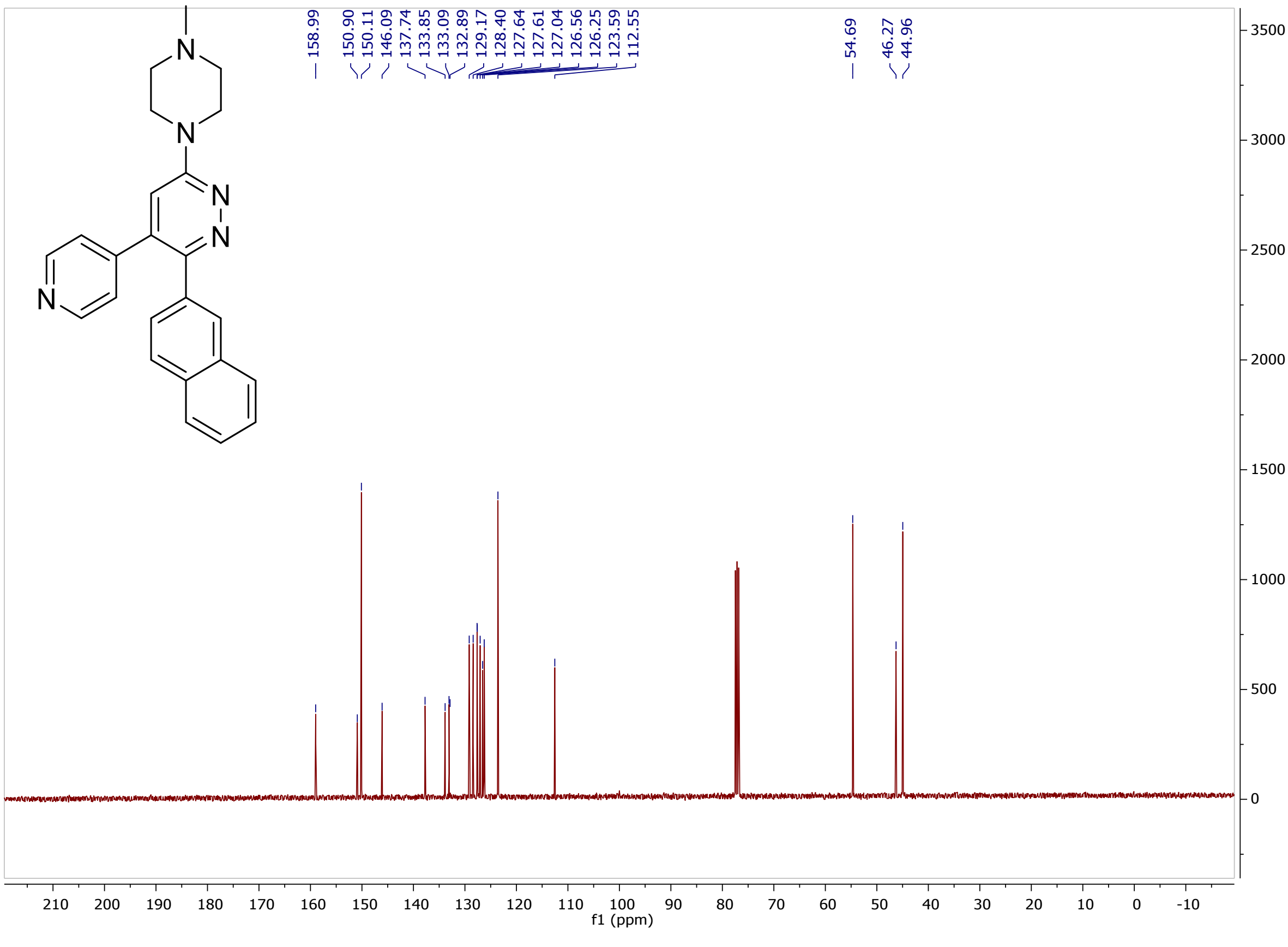

N,*N*-Dimethyl-6-(naphthalen-2-yl)-5-(pyridin-4-yl)pyridazin-3-amine **7** (**MW181**)

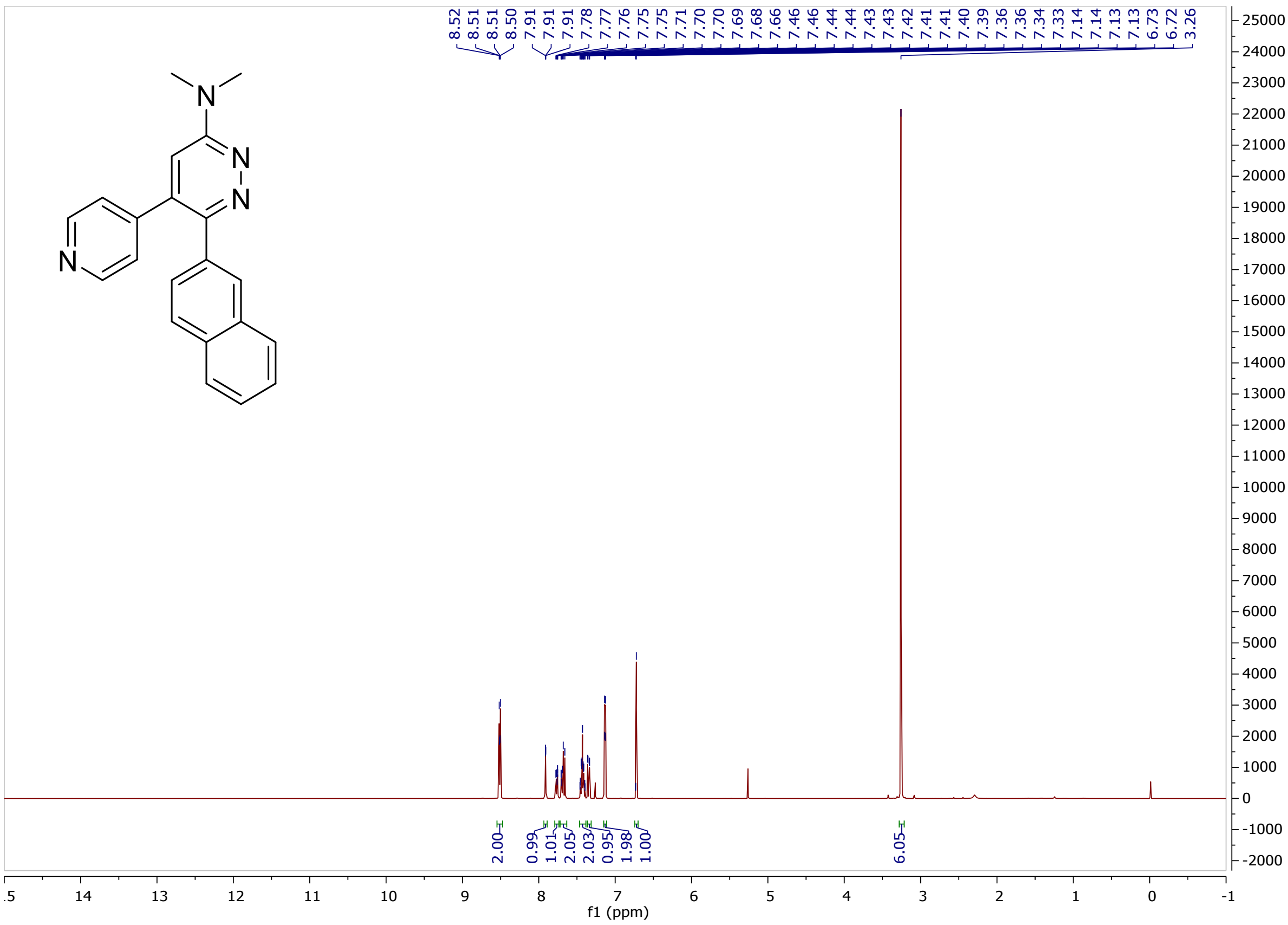

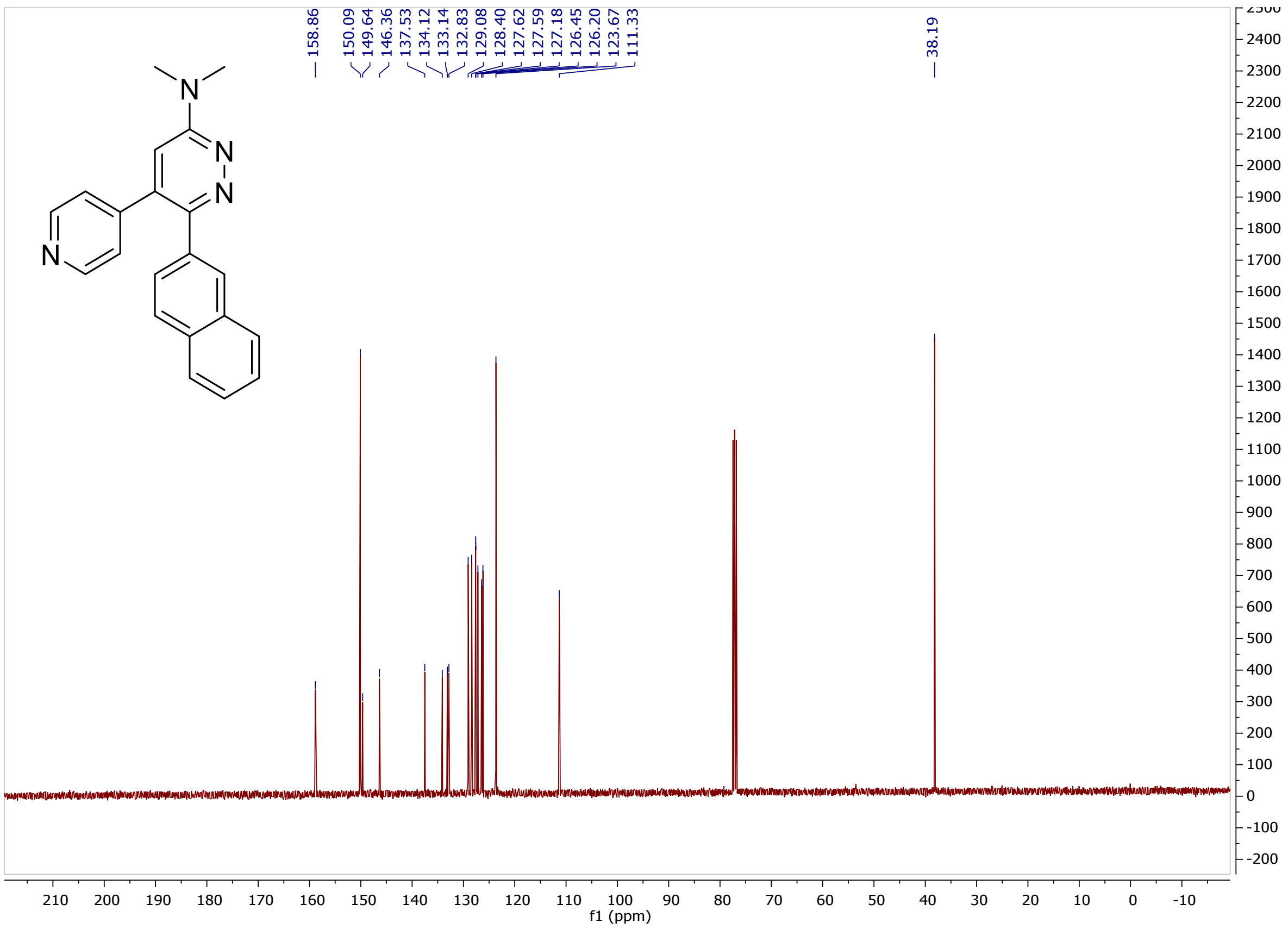

N^1,N^1-Dimethyl-N^2-(6-(naphthalen-2-yl)-5-(pyridin-4-yl)pyridazin-3-yl)ethane-1,2-diamine **8**

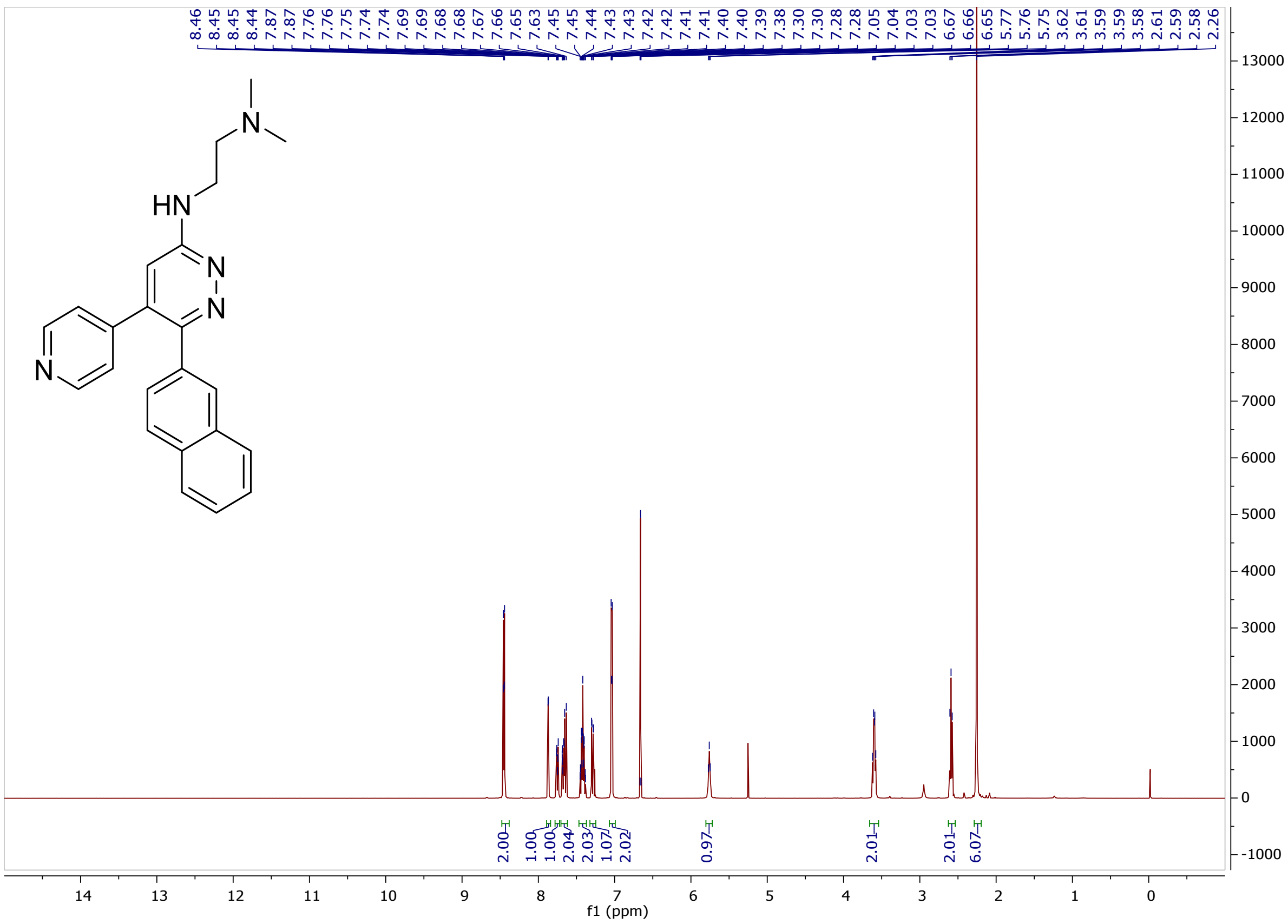

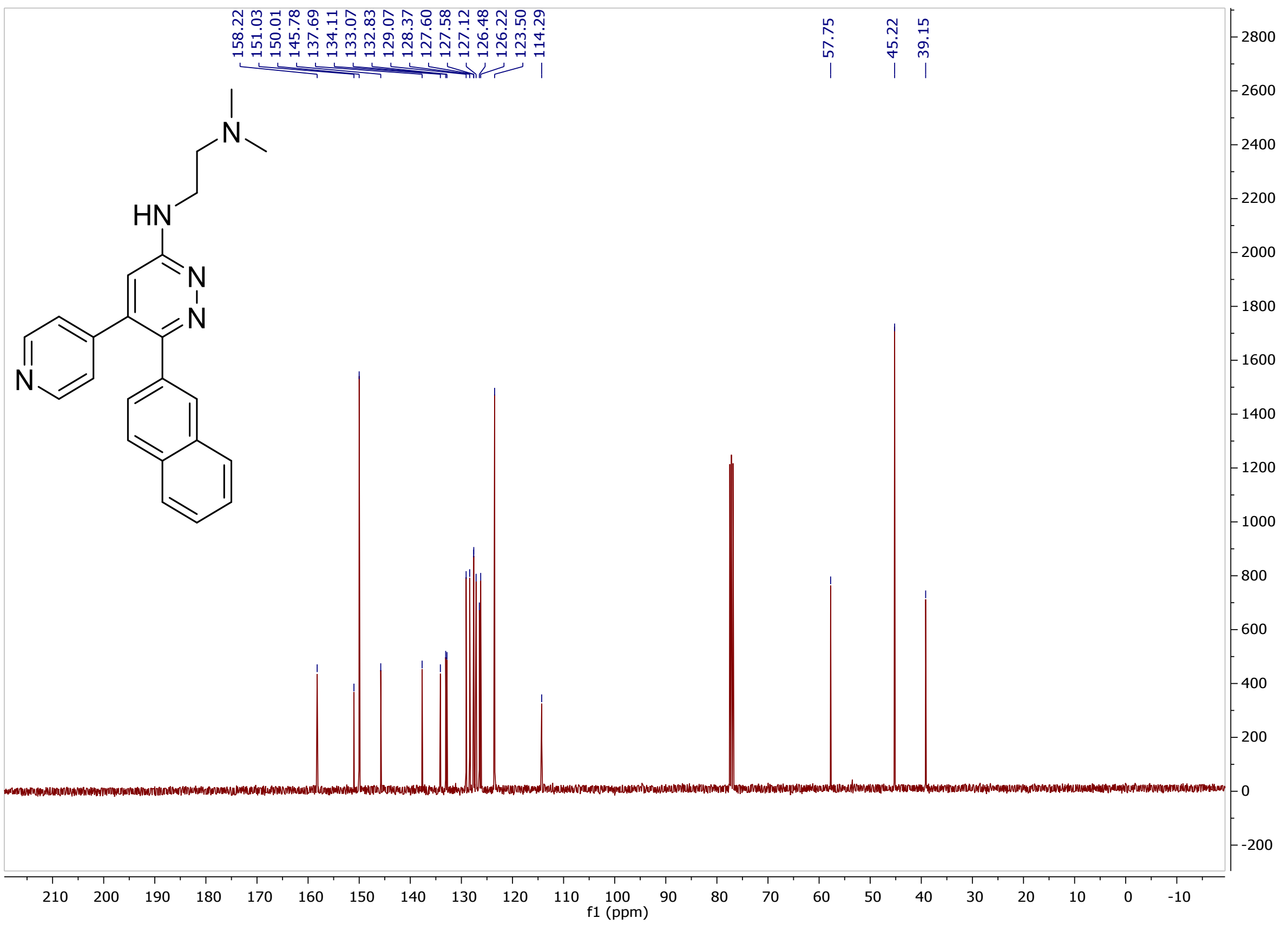

N[1],*N*[1]-Diethyl-*N*[2]-(6-(naphthalen-2-yl)-5-(pyridin-4-yl)pyridazin-3-yl)ethane-1,2-diamine **9**

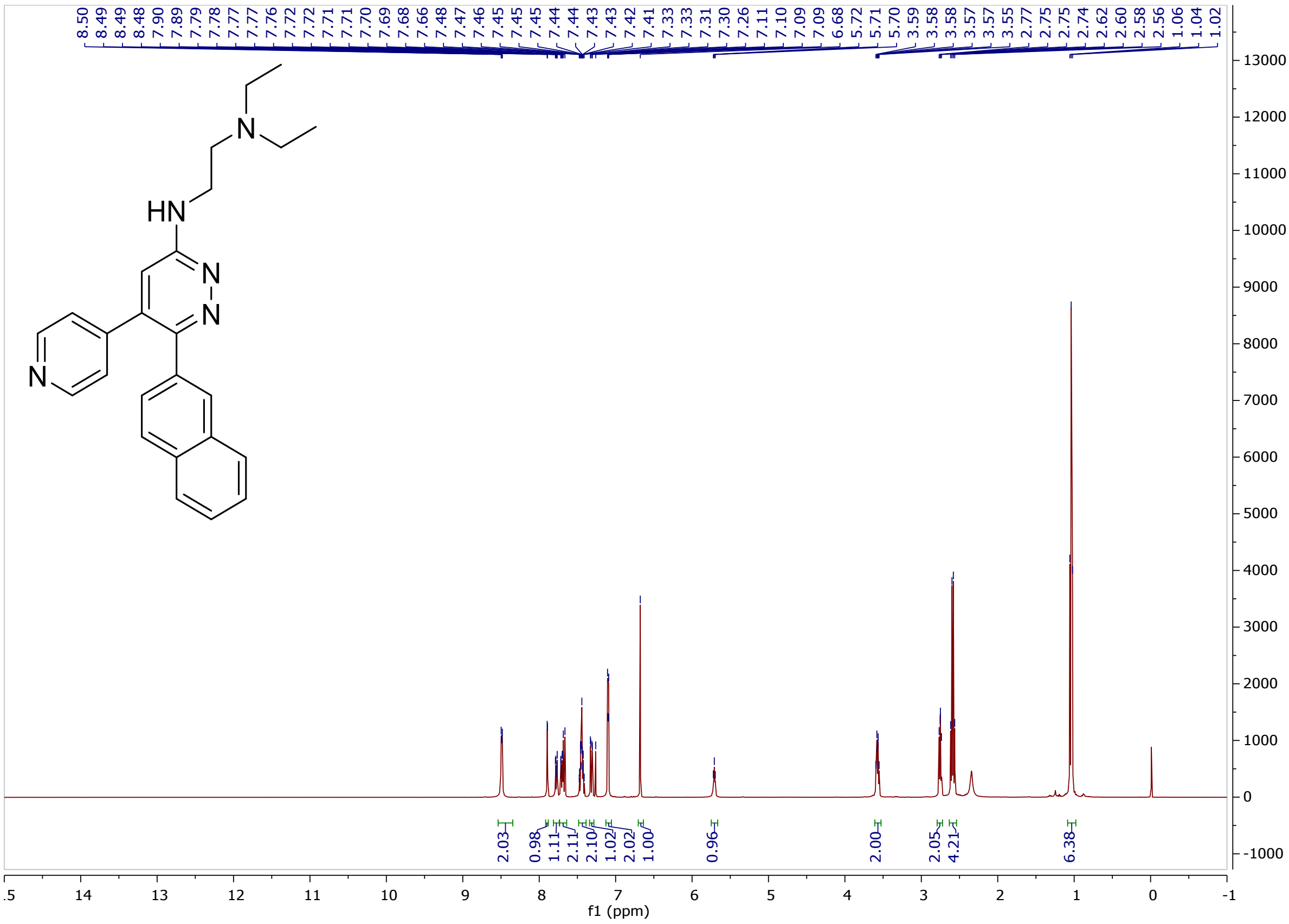

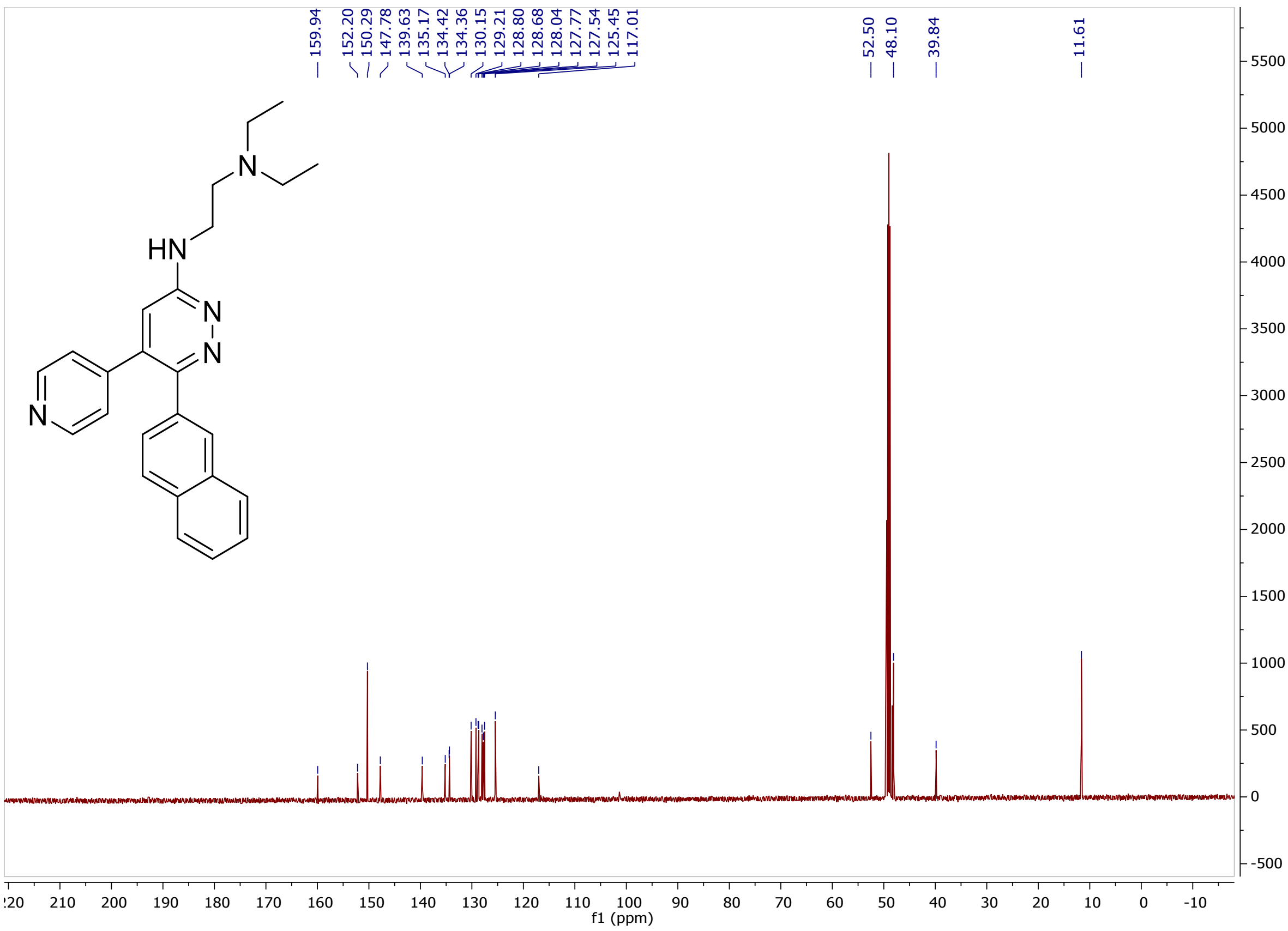

N^1,N^1-Dimethyl-N^3-(6-(naphthalen-2-yl)-5-(pyridin-4-yl)pyridazin-3-yl)propane-1,3-diamine **10**

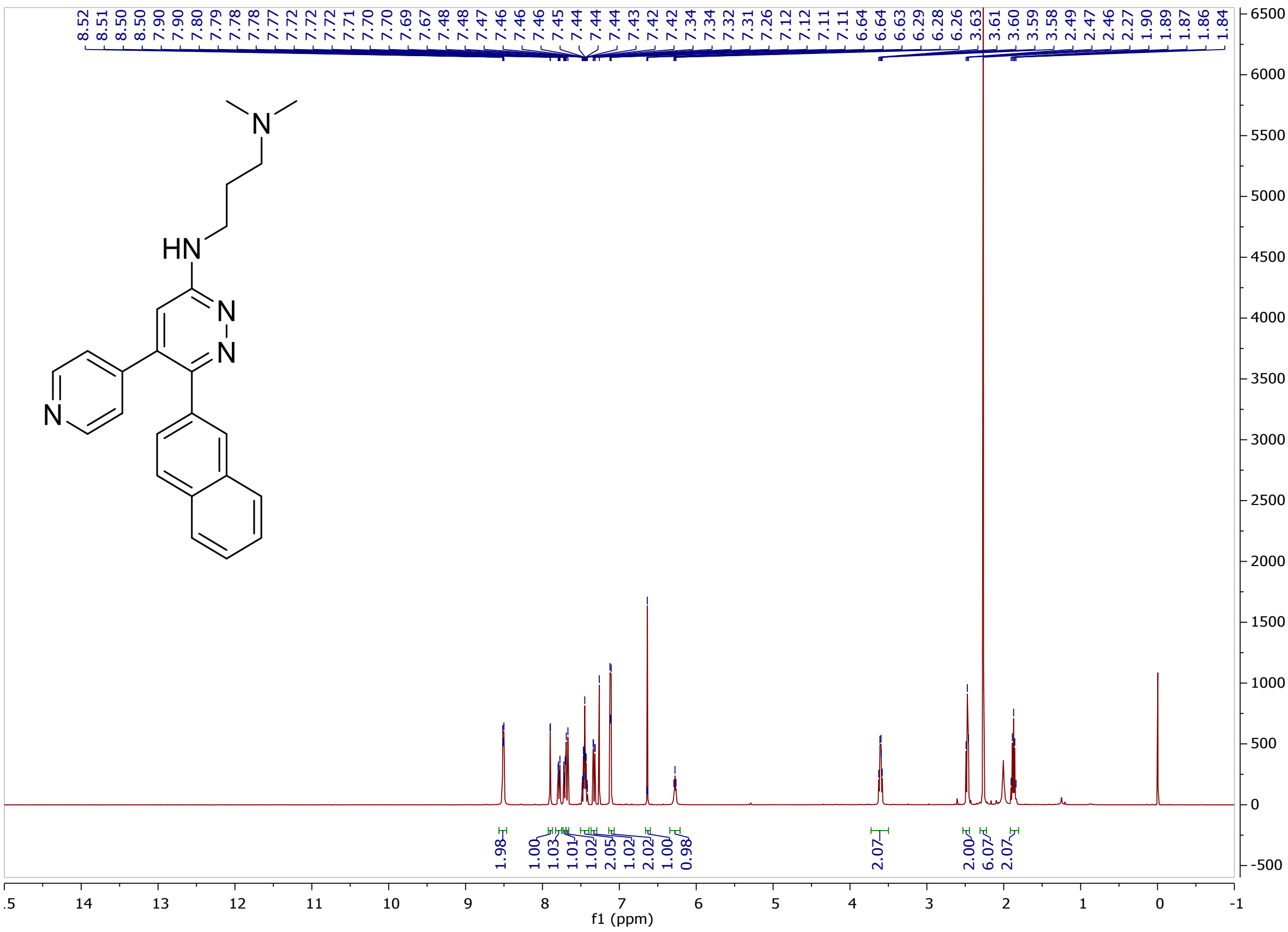

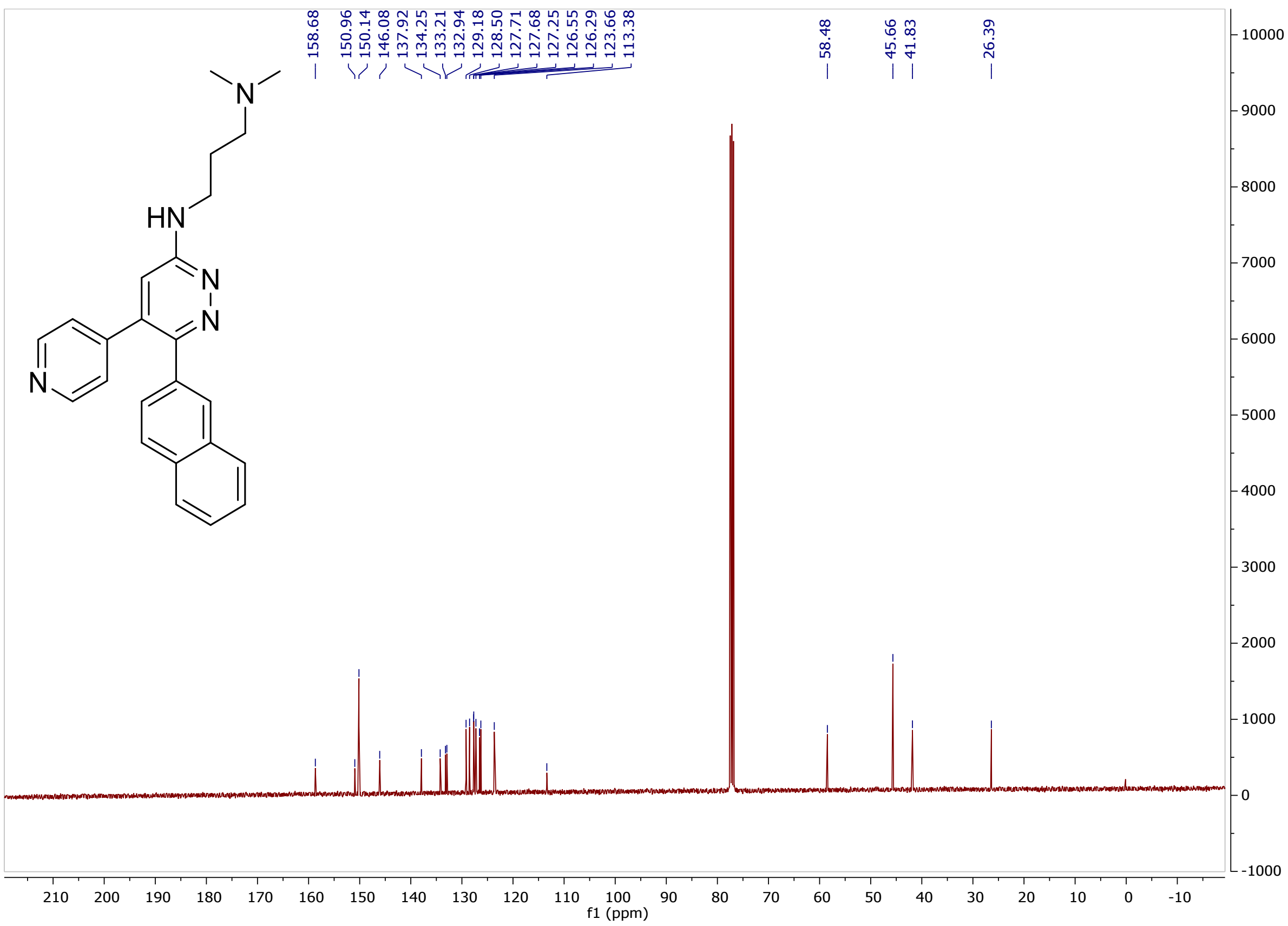

Tert-butyl methyl(2-(methyl(6-(naphthalen-2-yl)-5-(pyridin-4-yl)pyridazin-3-yl)amino)ethyl)carbamate **11a**

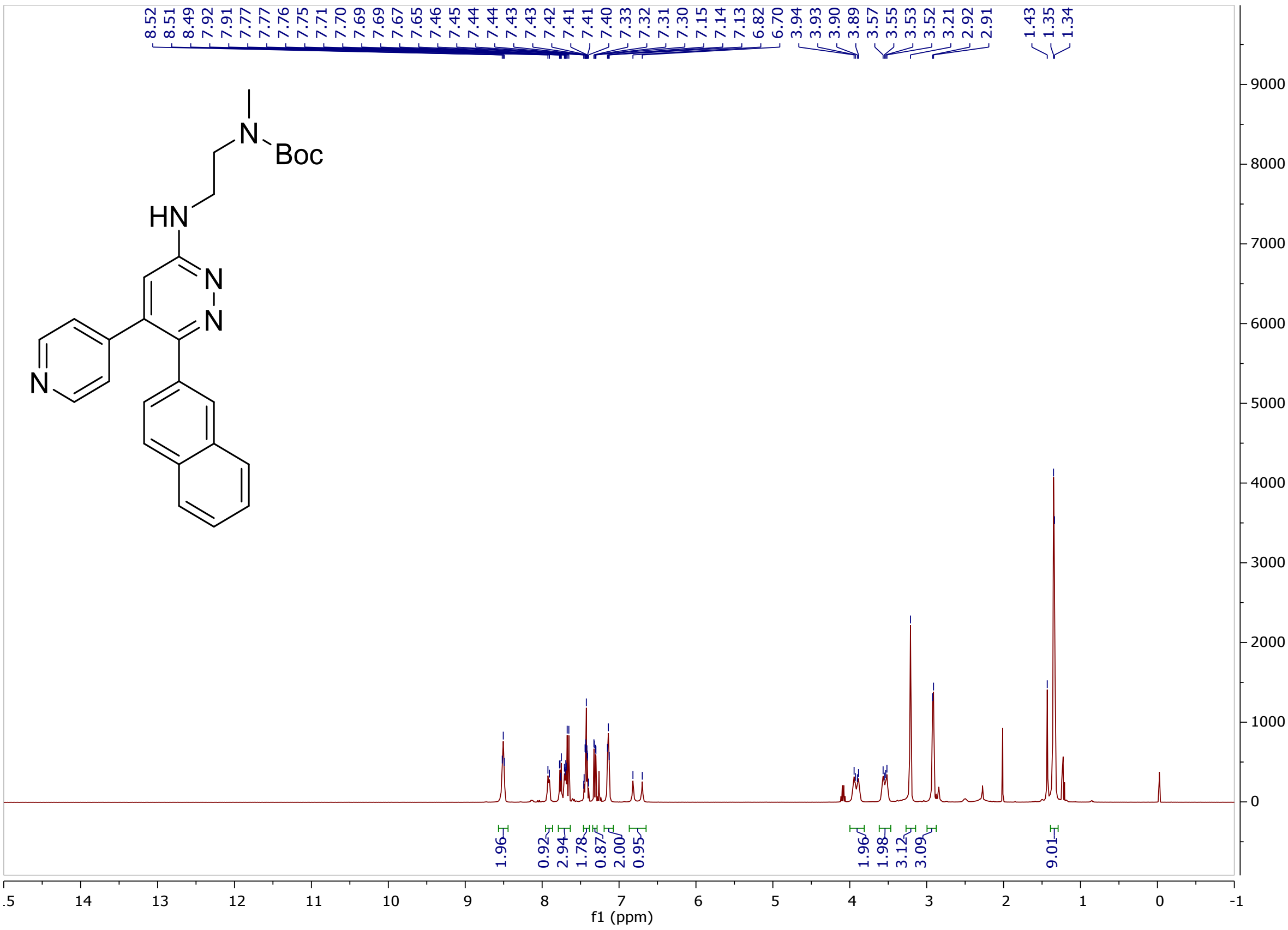

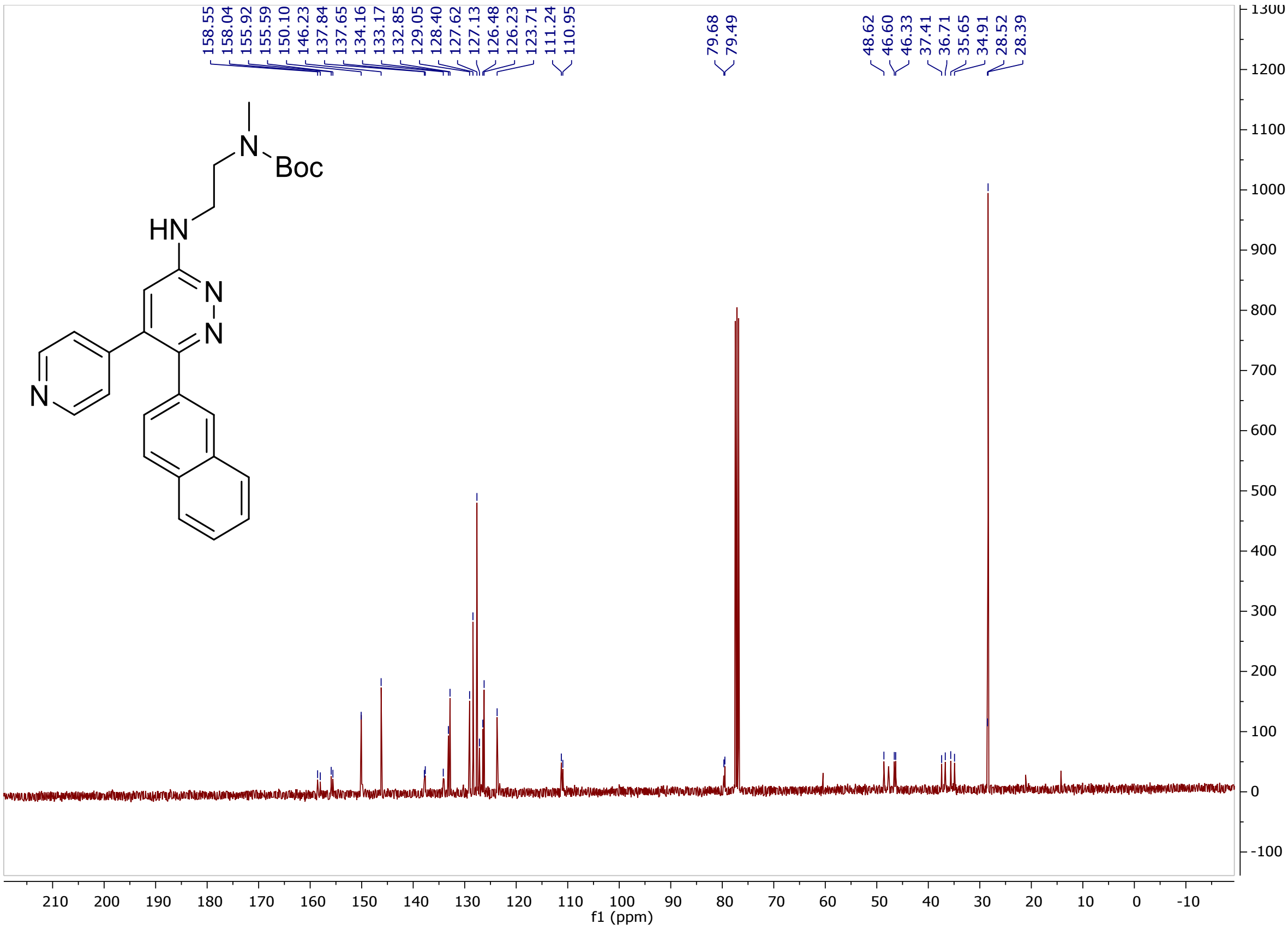

N^1,N^2-Dimethyl-N^1-(6-(naphthalen-2-yl)-5-(pyridin-4-yl)pyridazin-3-yl)ethane-1,2-diamine **11**

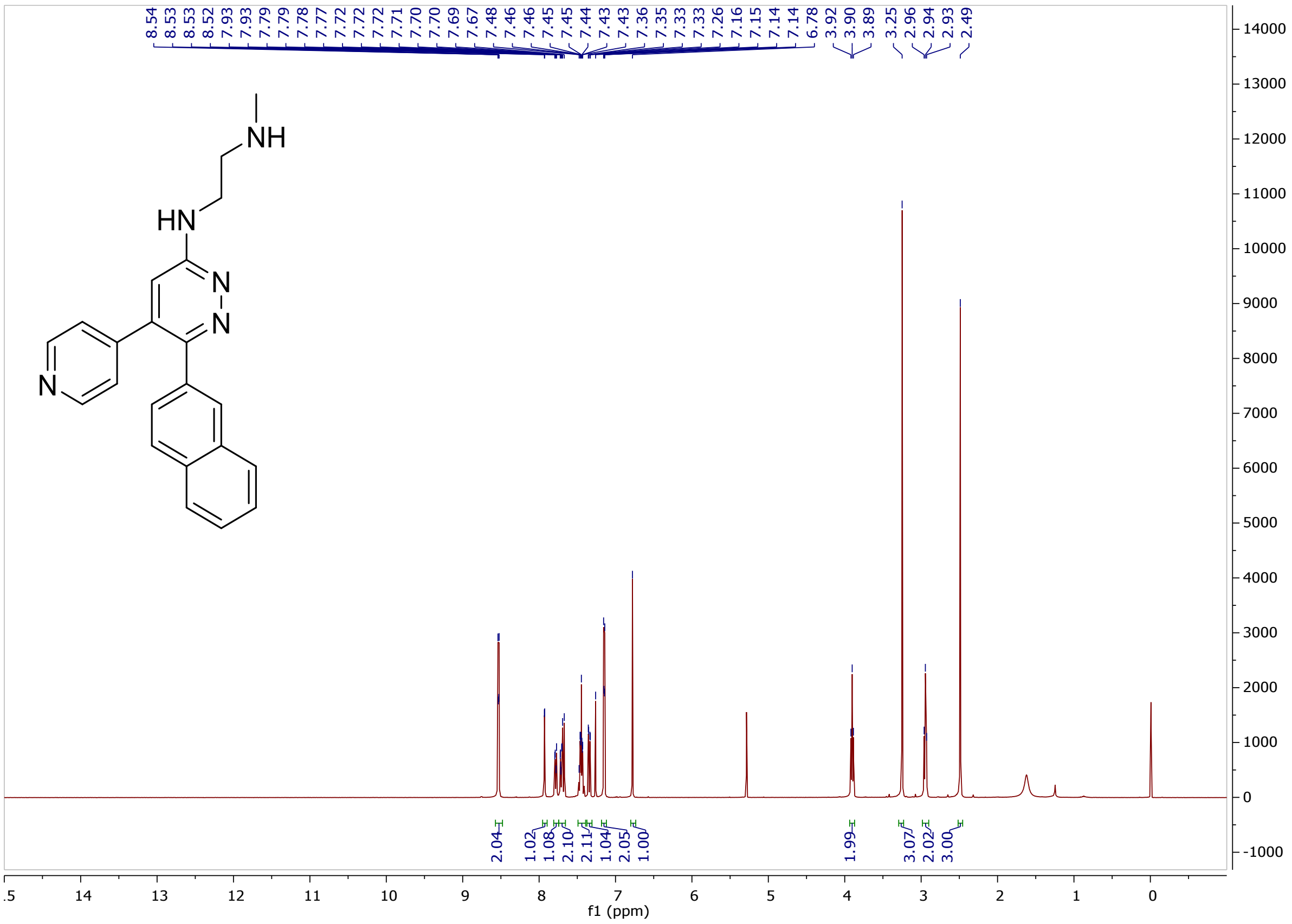

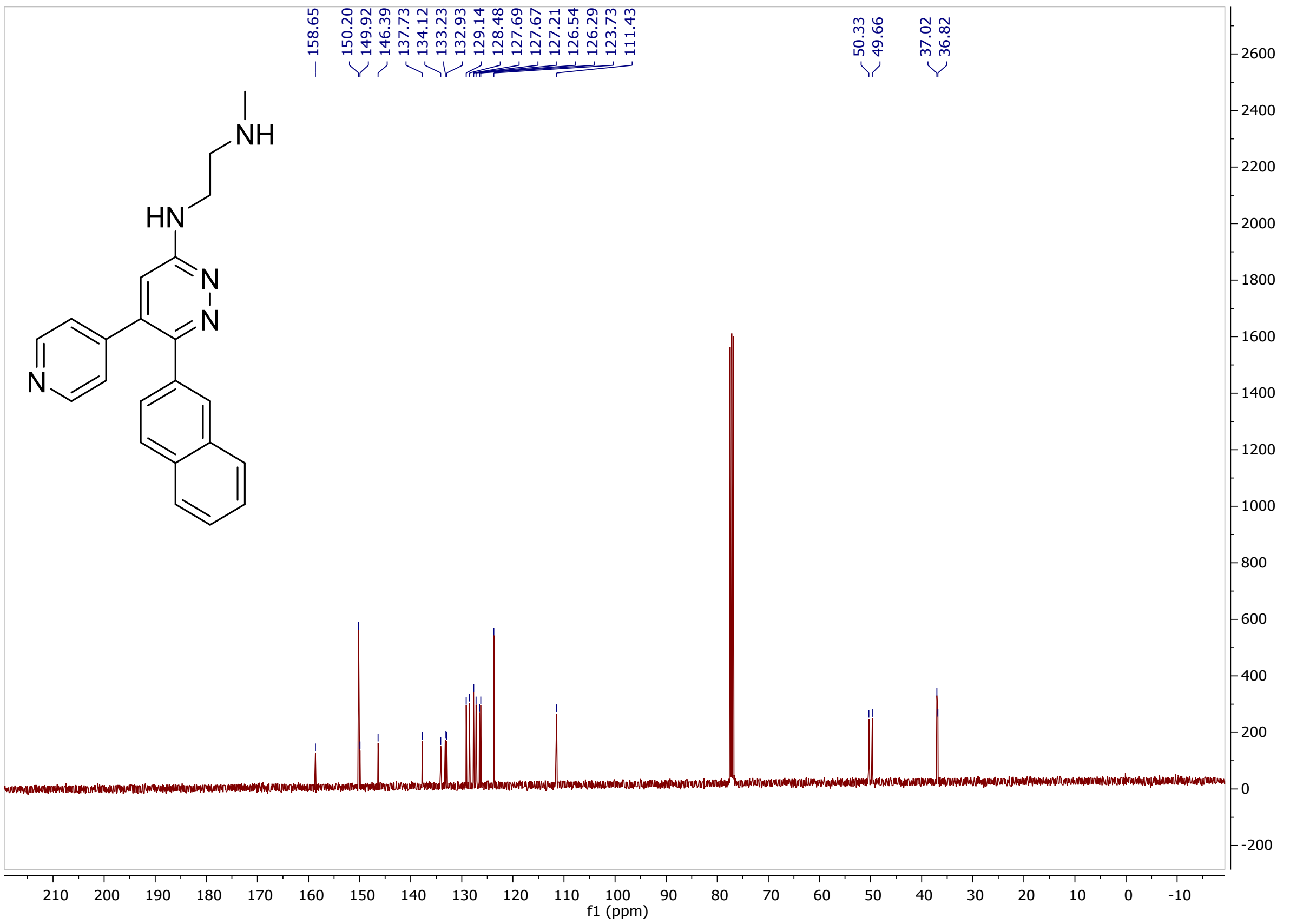

N-Isopentyl-6-(naphthalen-2-yl)-5-(pyridin-4-yl)pyridazin-3-amine **12**

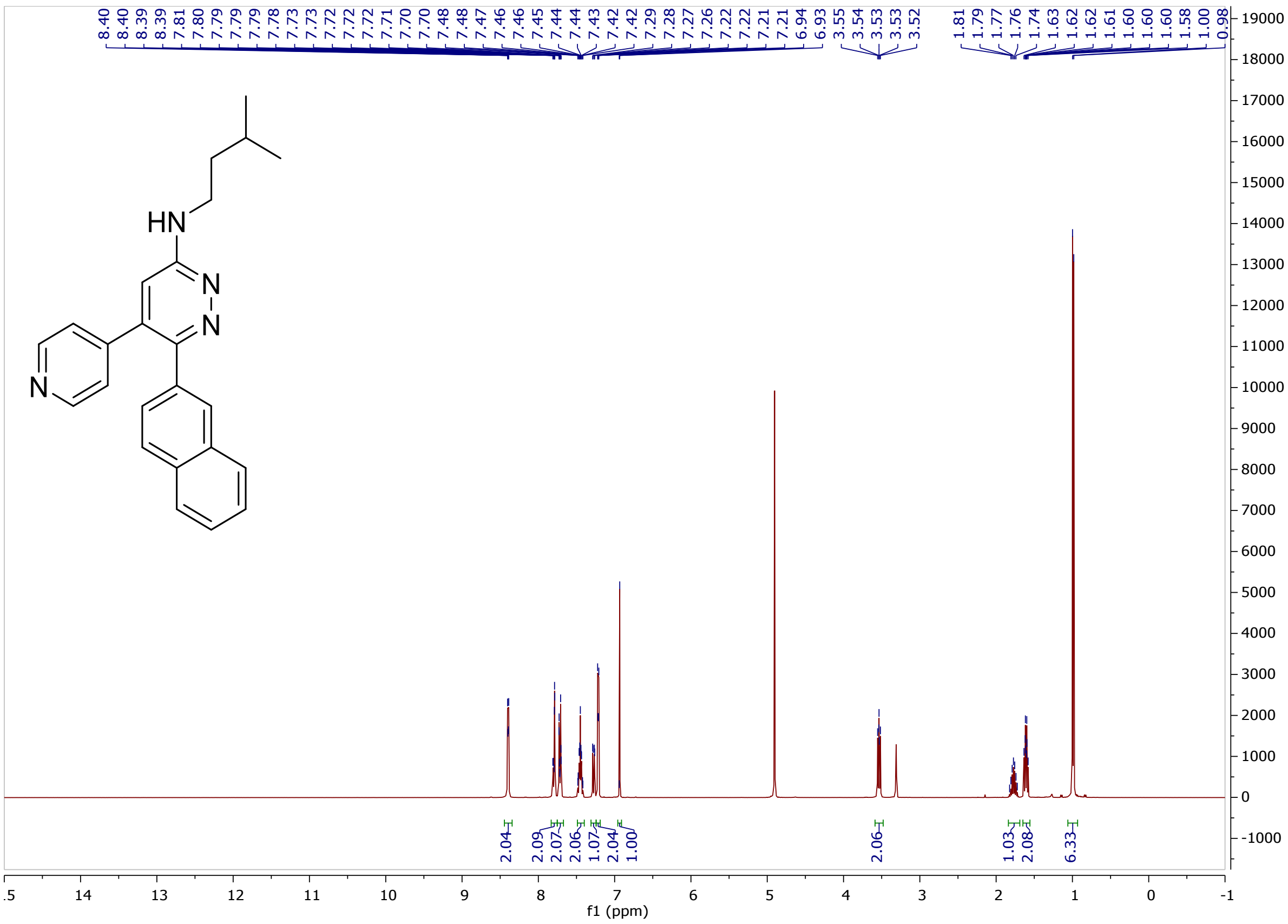

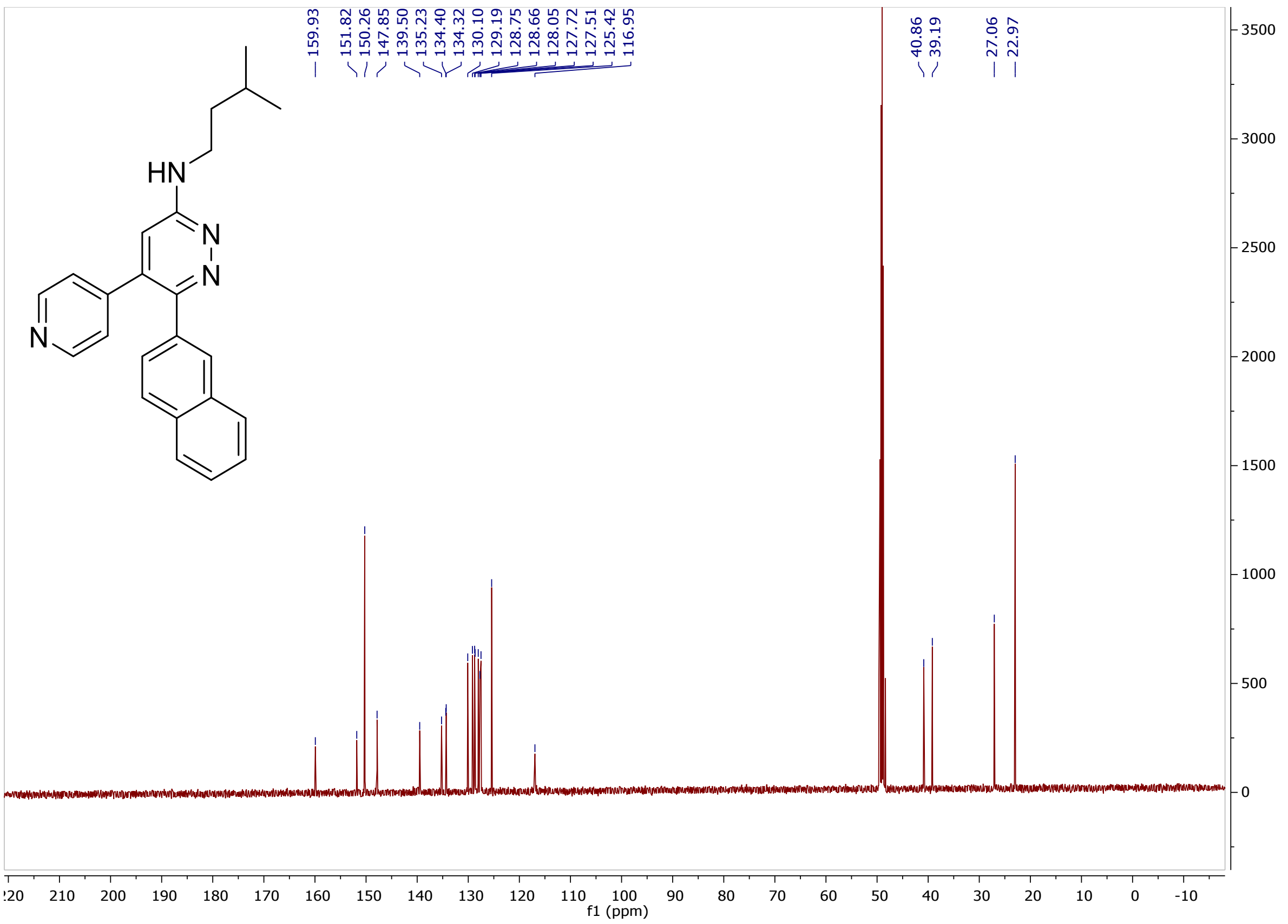

N-Isobutyl-6-(naphthalen-2-yl)-5-(pyridin-4-yl)pyridazin-3-amine **13**

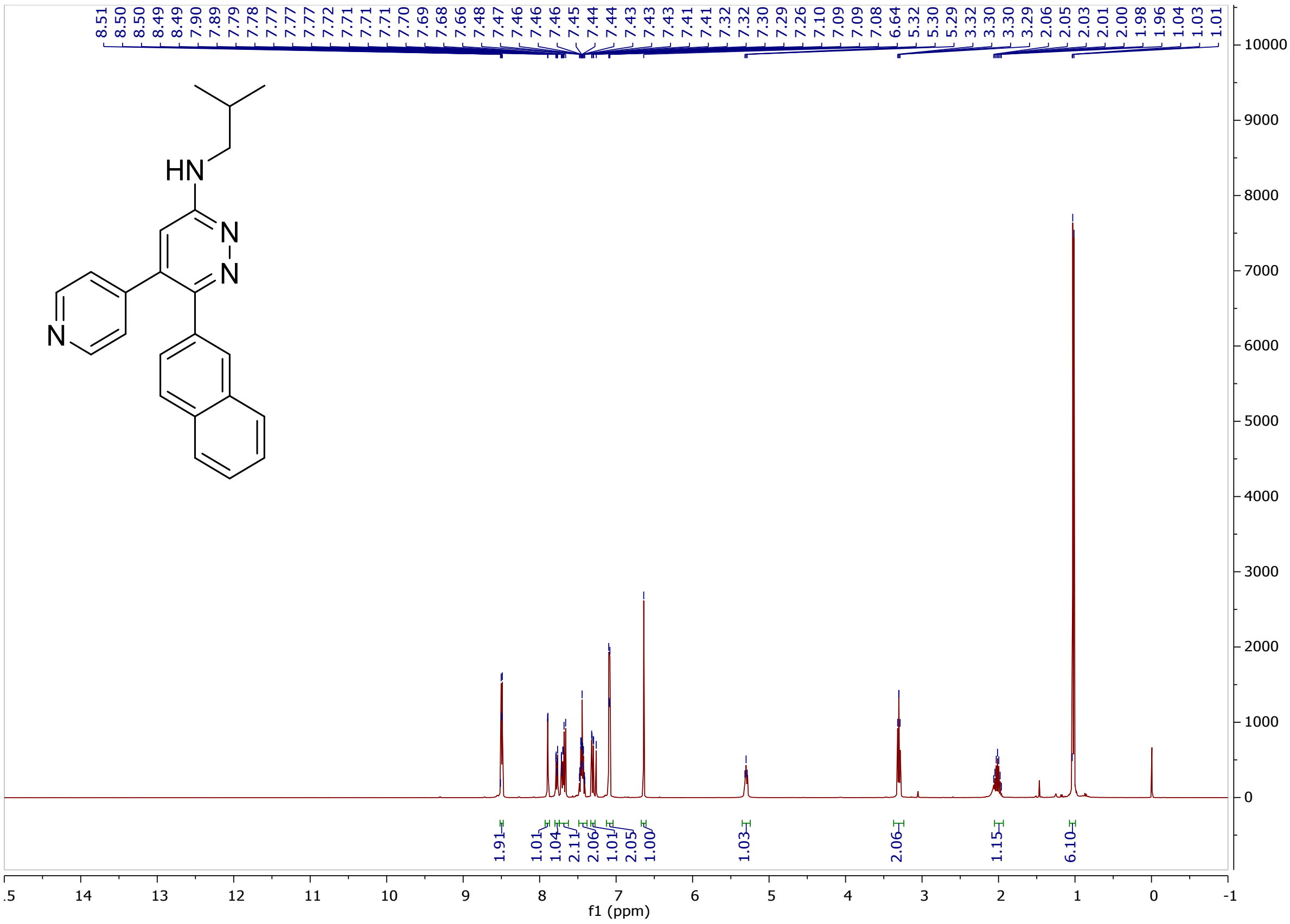

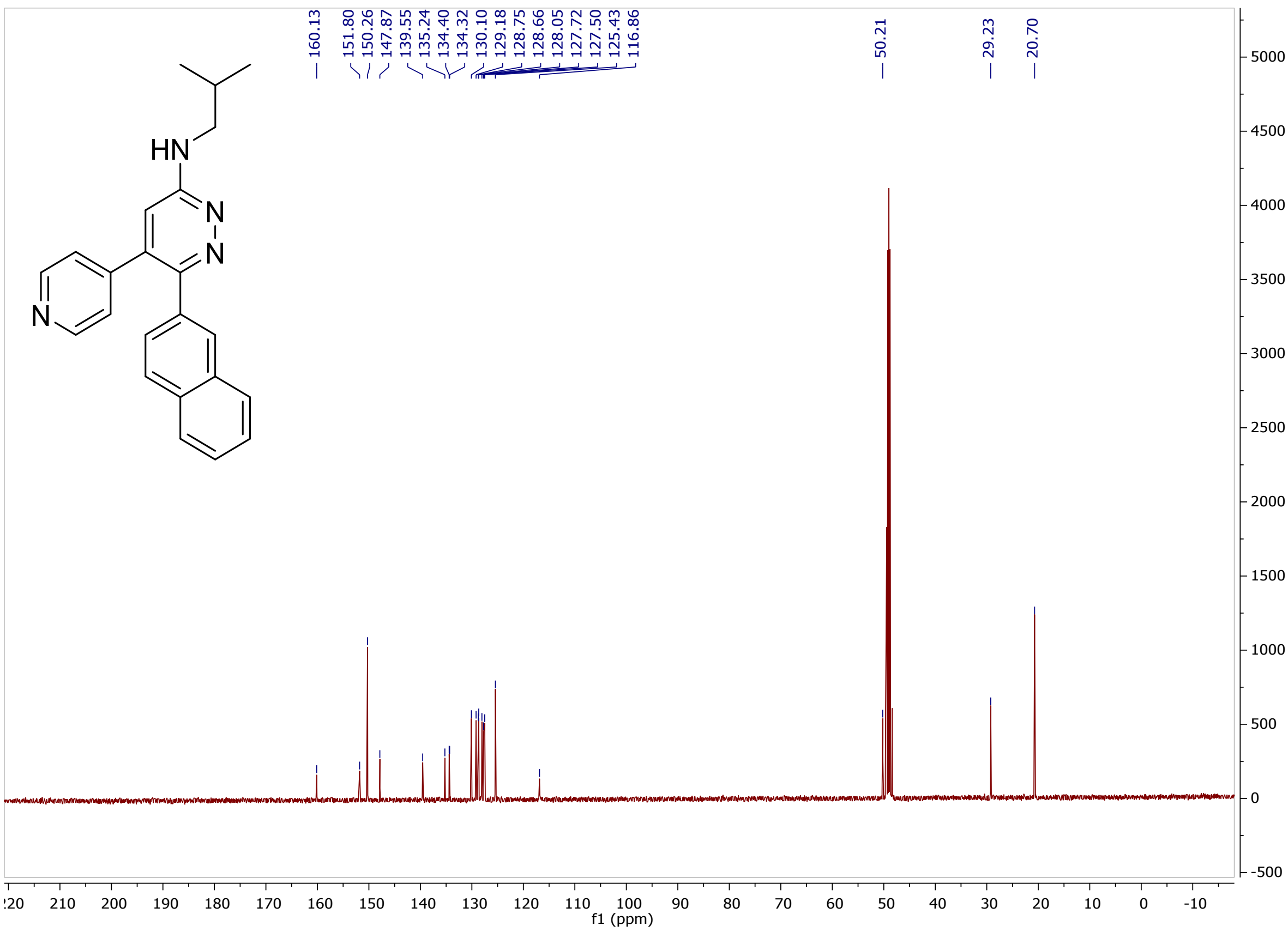

N-Benzyl-6-(naphthalen-2-yl)-5-(pyridin-4-yl)pyridazin-3-amine **14**

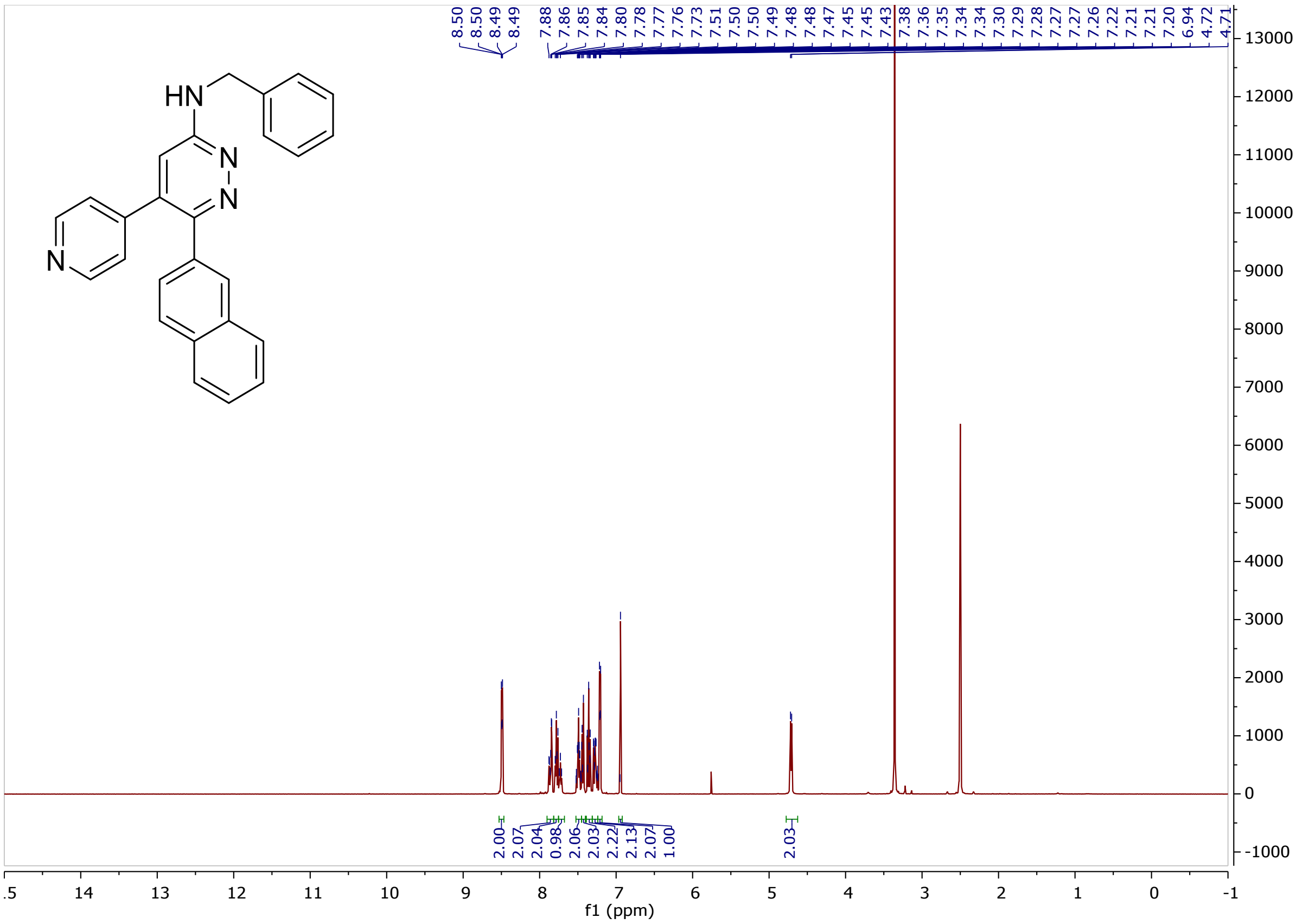

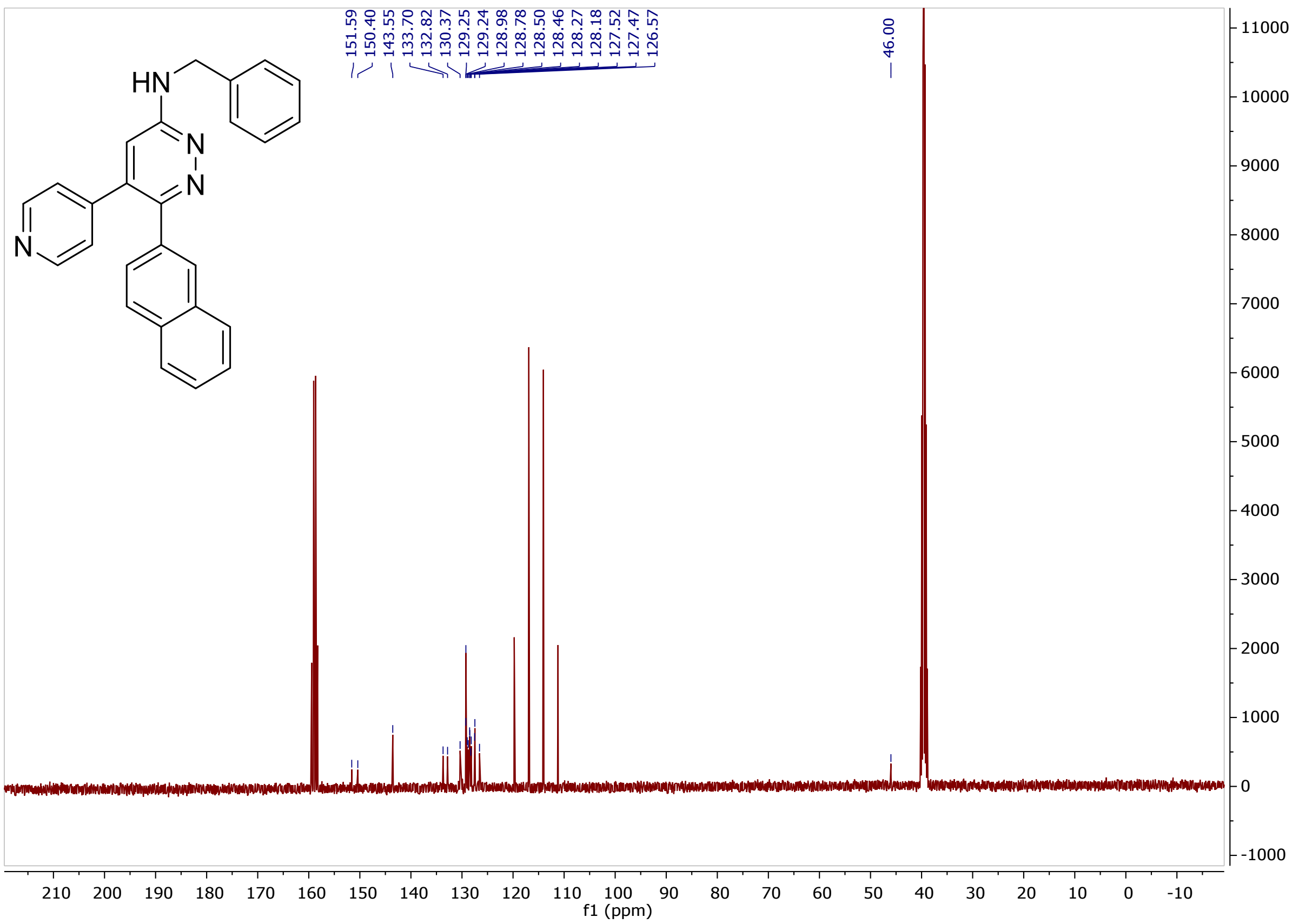

O-Benzyl-*N*-(6-(naphthalen-2-yl)-5-(pyridin-4-yl)pyridazin-3-yl)hydroxylamine **15**

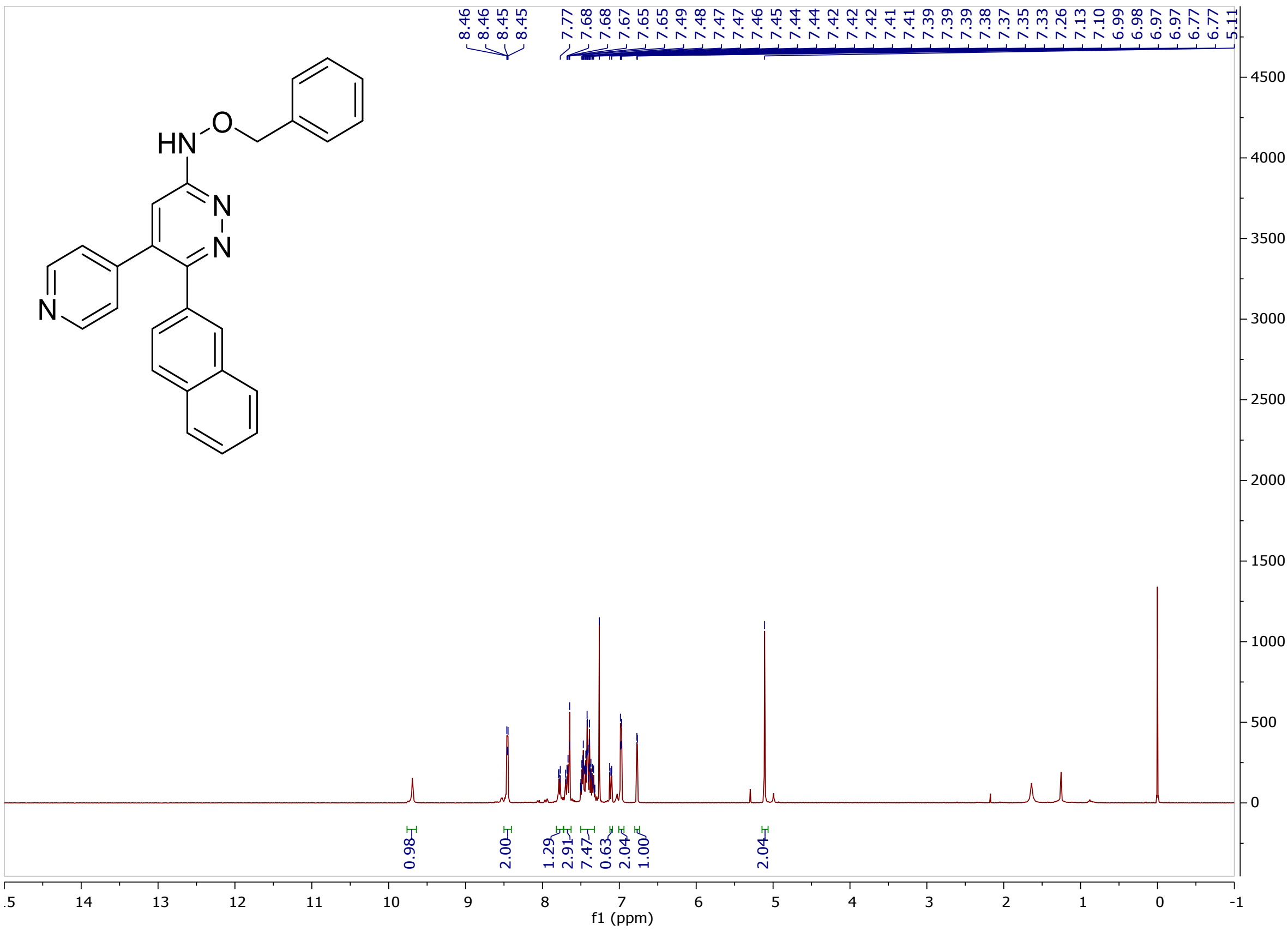

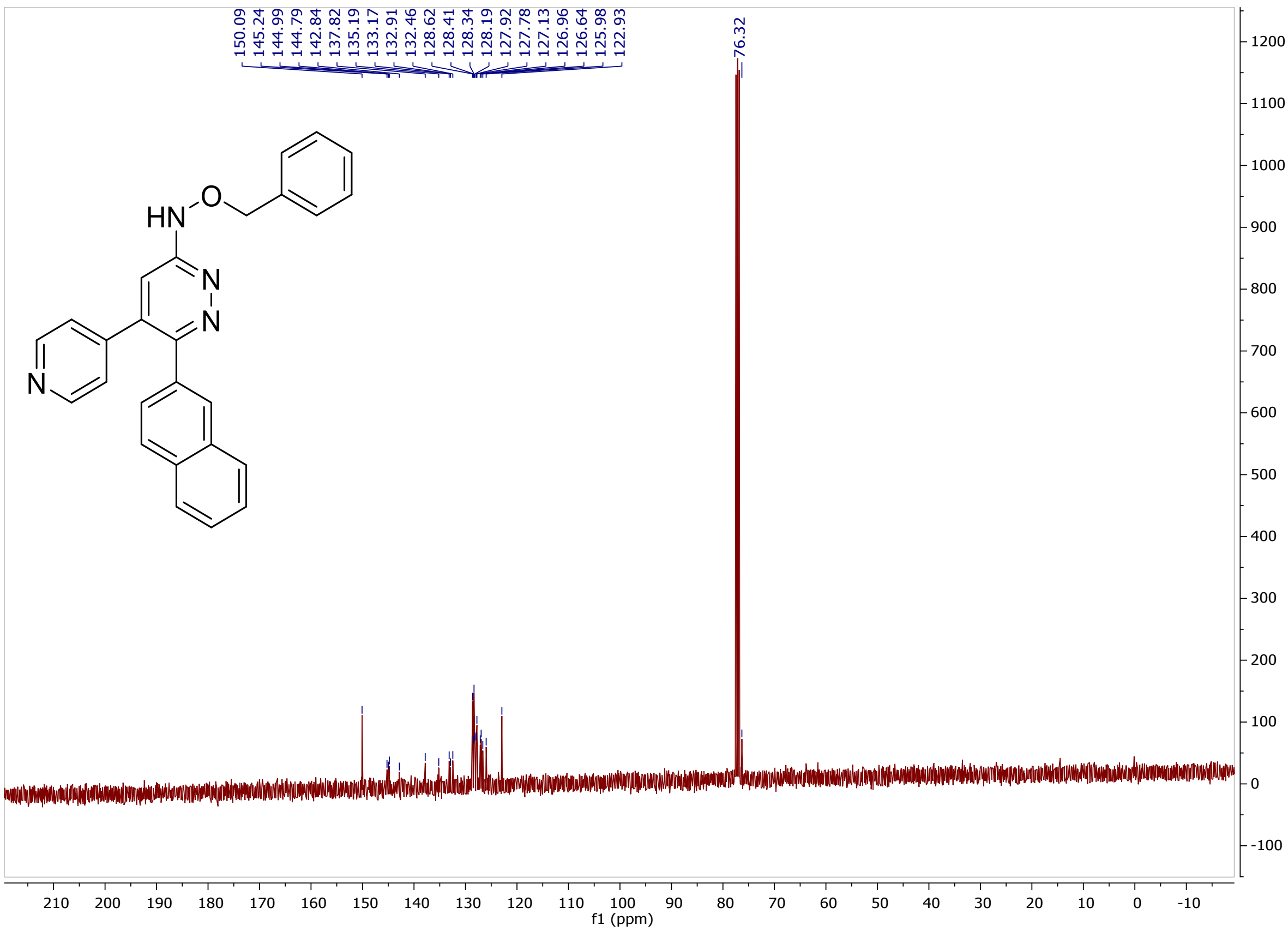

N,*O*-Dimethyl-*N*-(6-(naphthalen-2-yl)-5-(pyridin-4-yl)pyridazin-3-yl)hydroxylamine **16**

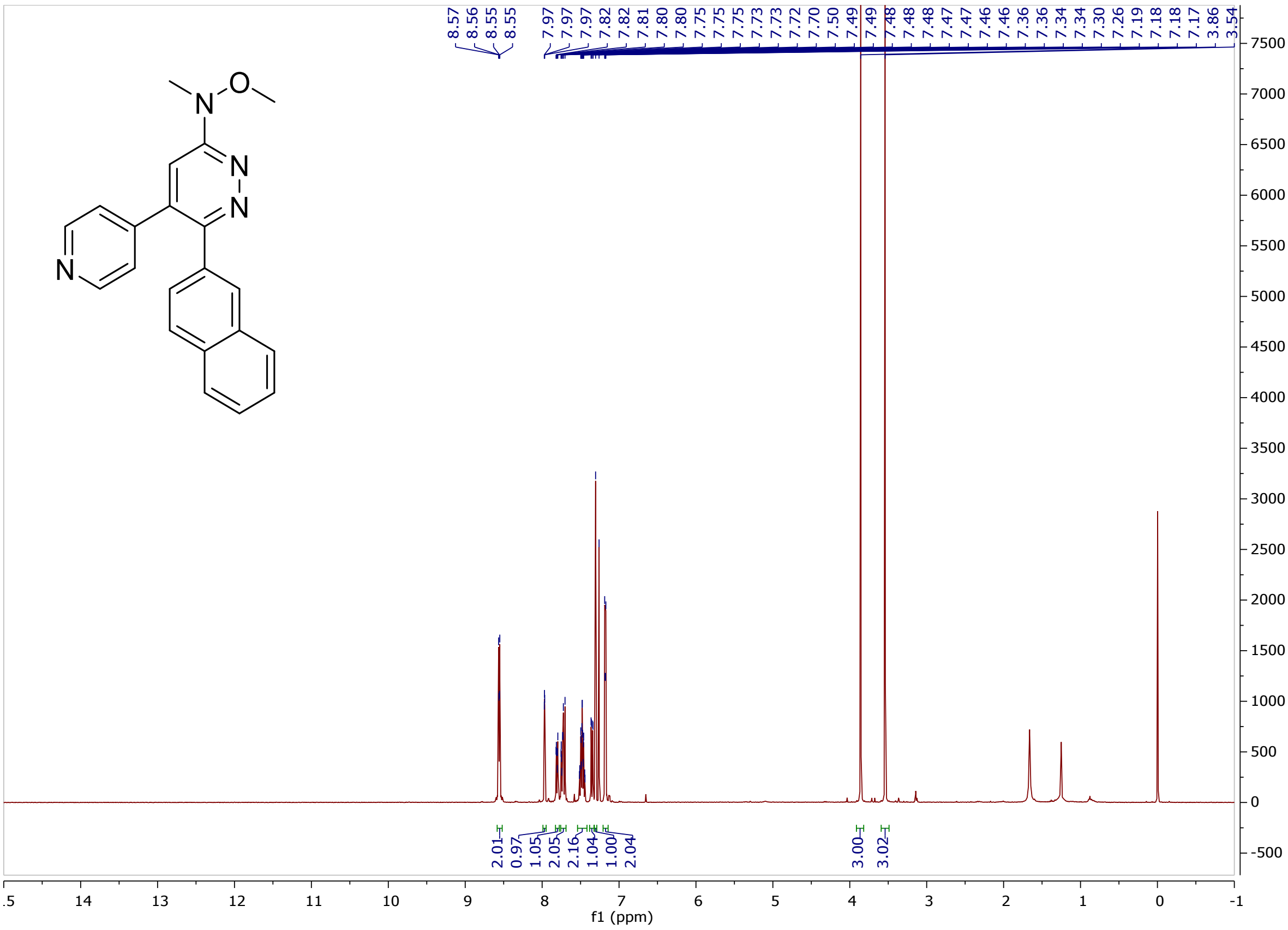

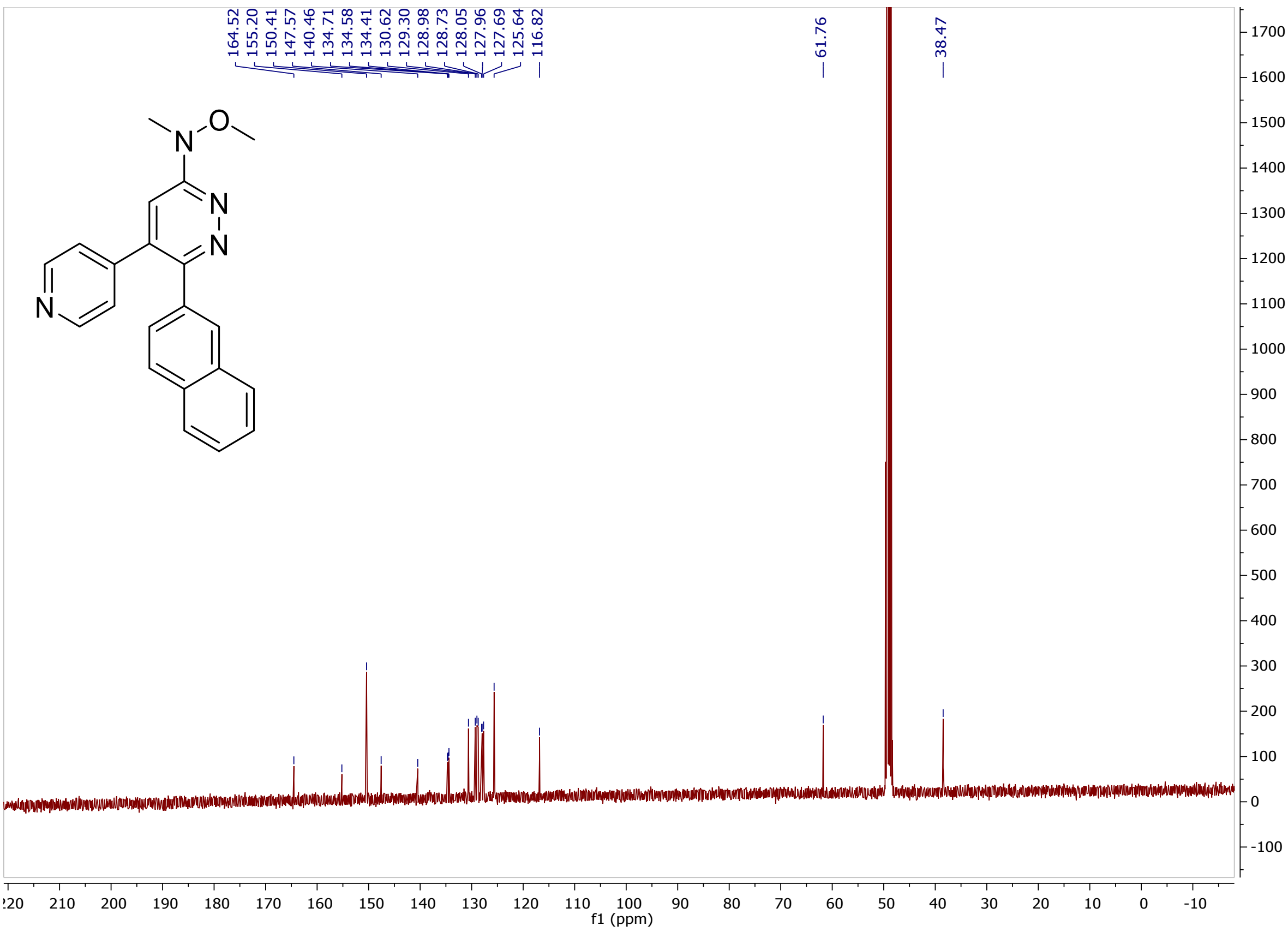

6-Hydrazineyl-3-(naphthalen-2-yl)-4-(pyridin-4-yl)pyridazine **17**

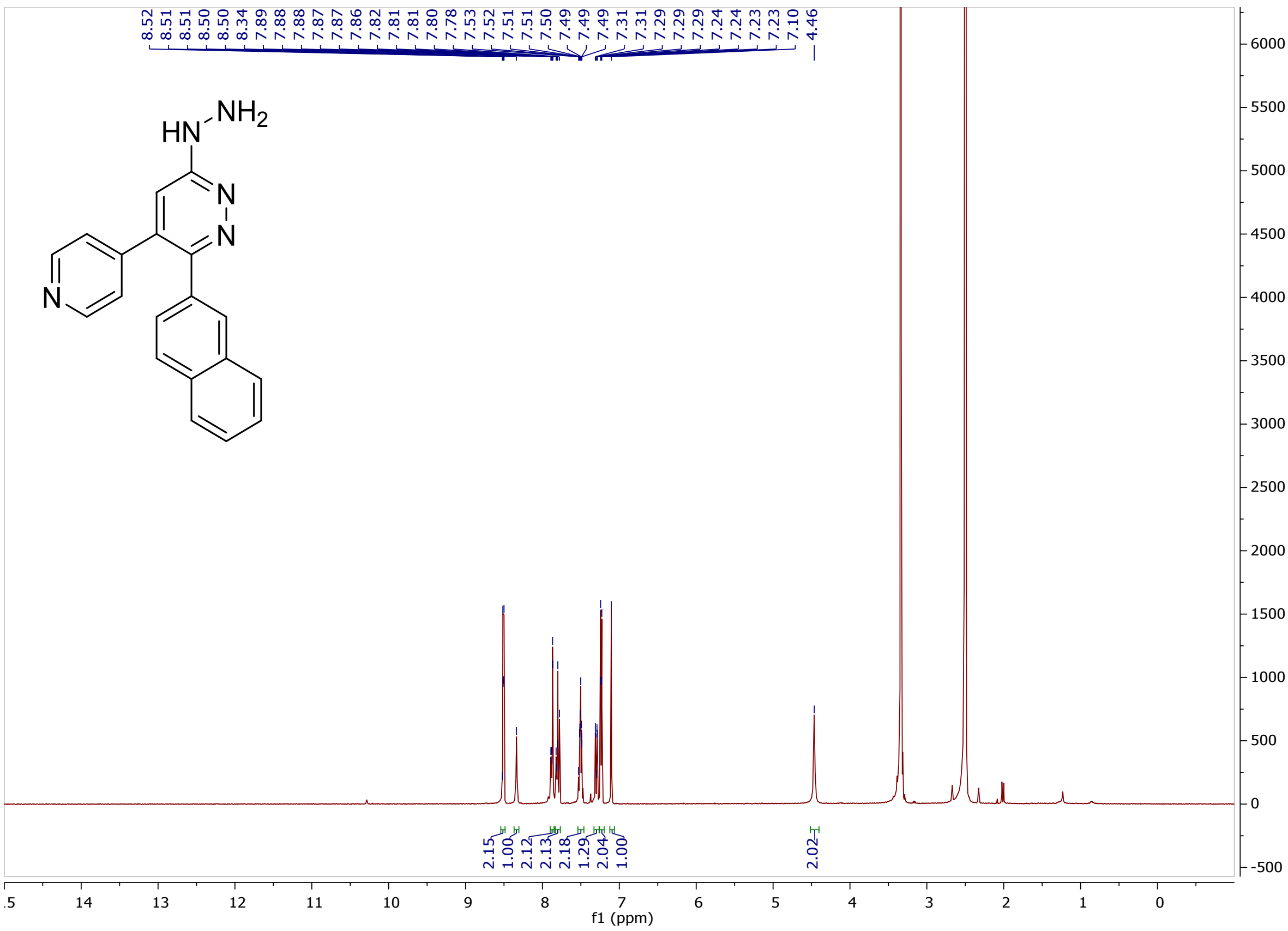

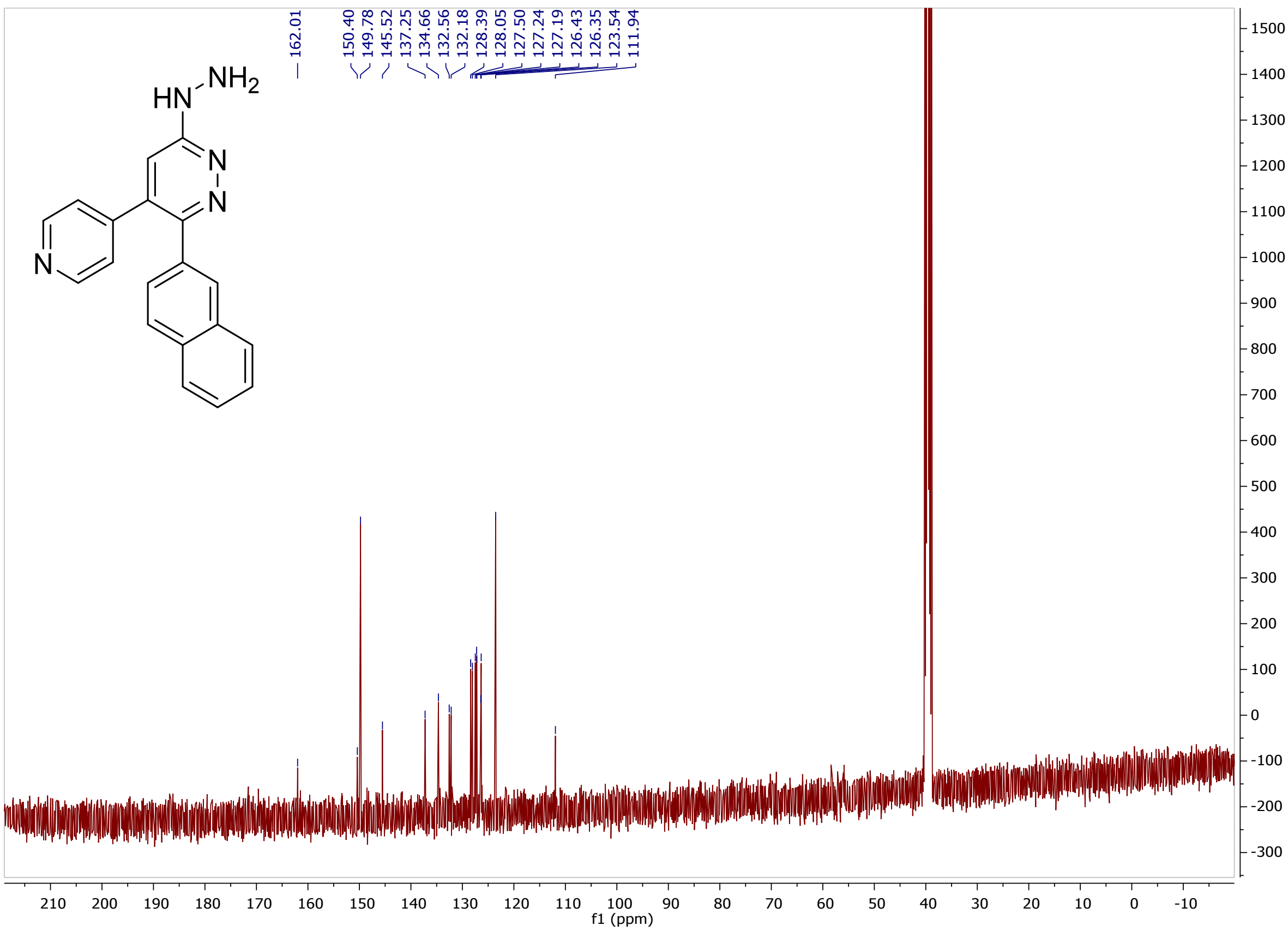

Tert-butyl 1-methyl-2-(6-(naphthalen-2-yl)-5-(pyridin-4-yl)pyridazin-3-yl)hydrazine-1-carboxylate **18a**

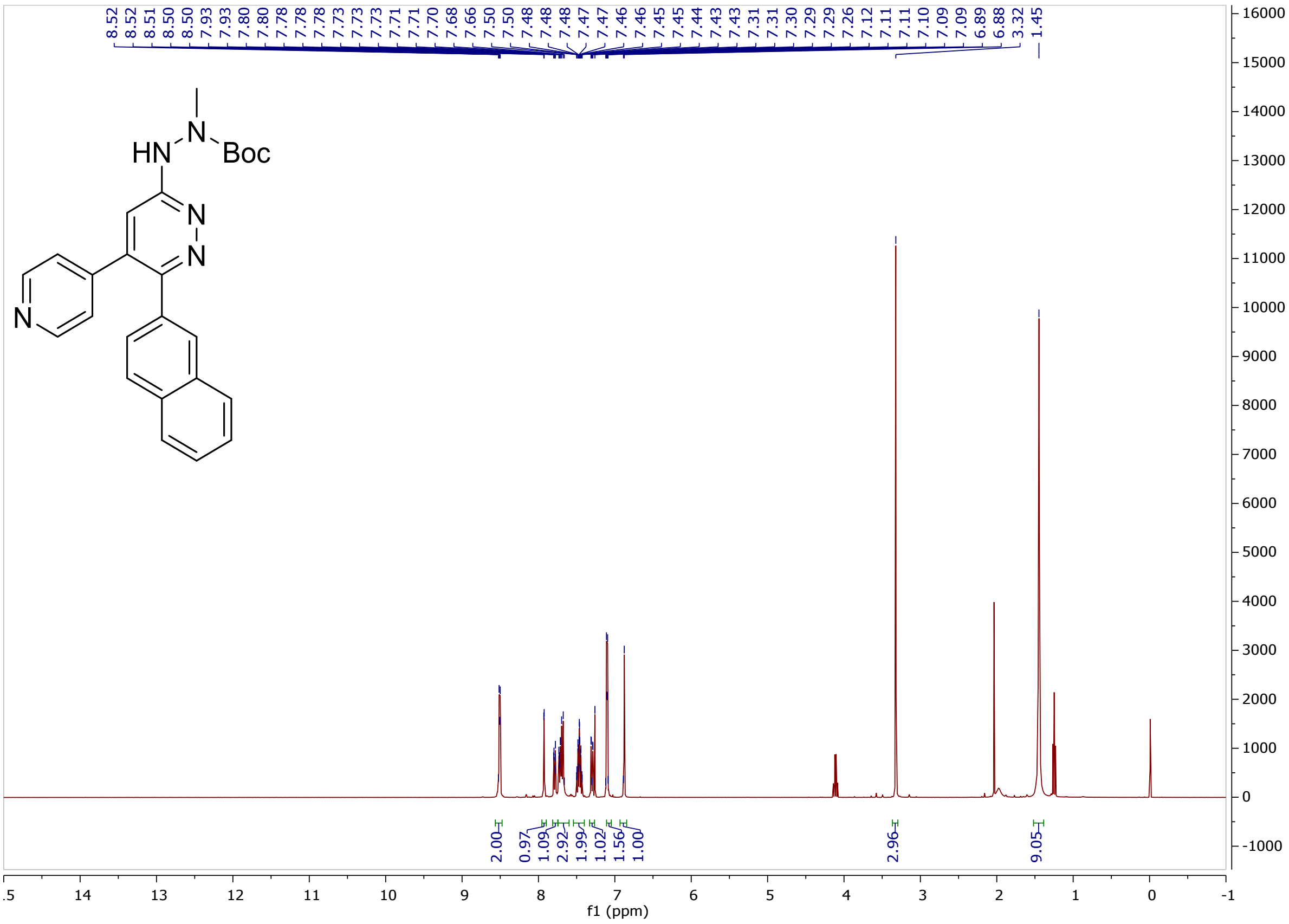

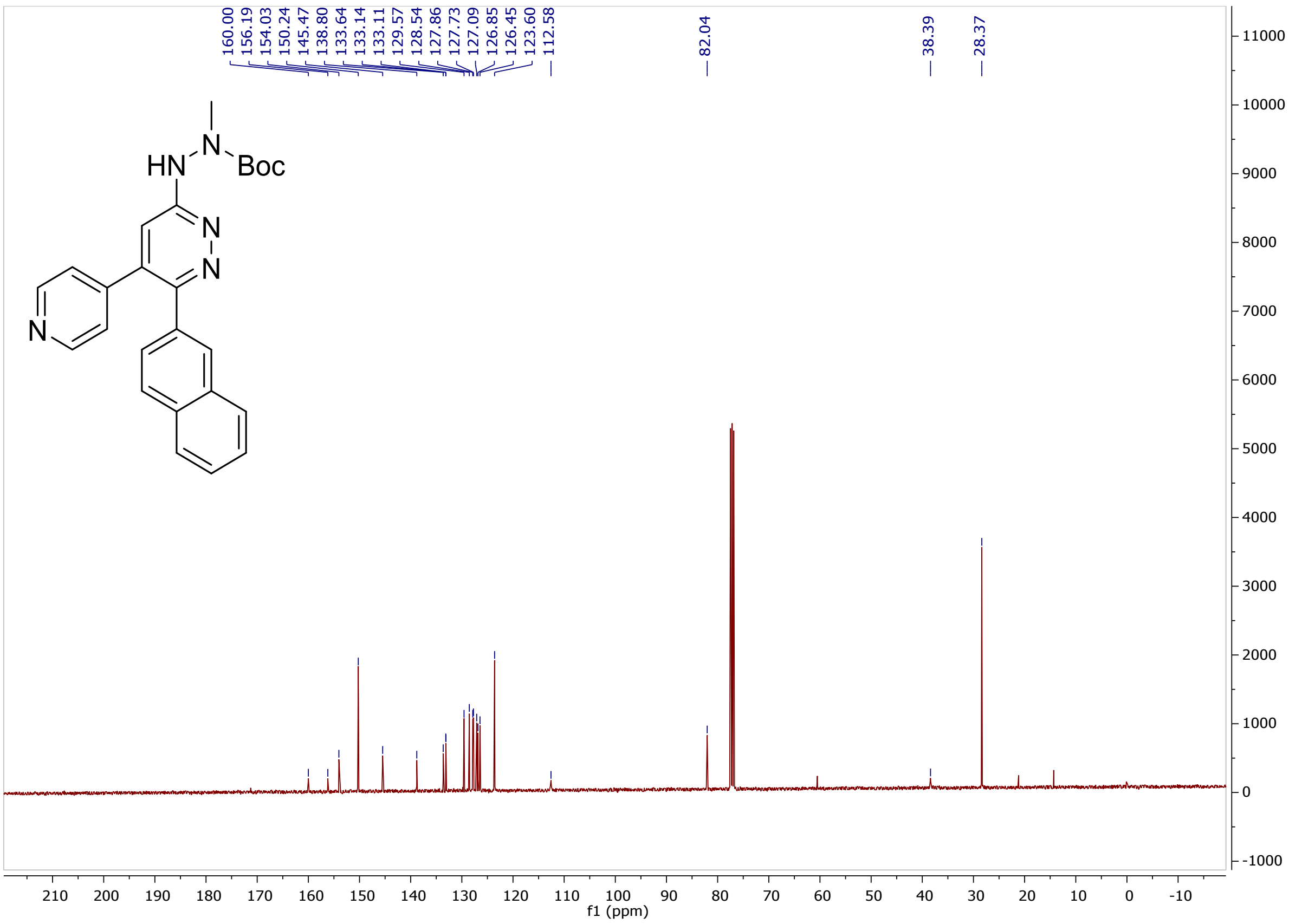

6-(2-Methylhydrazineyl)-3-(naphthalen-2-yl)-4-(pyridin-4-yl)pyridazine **18**

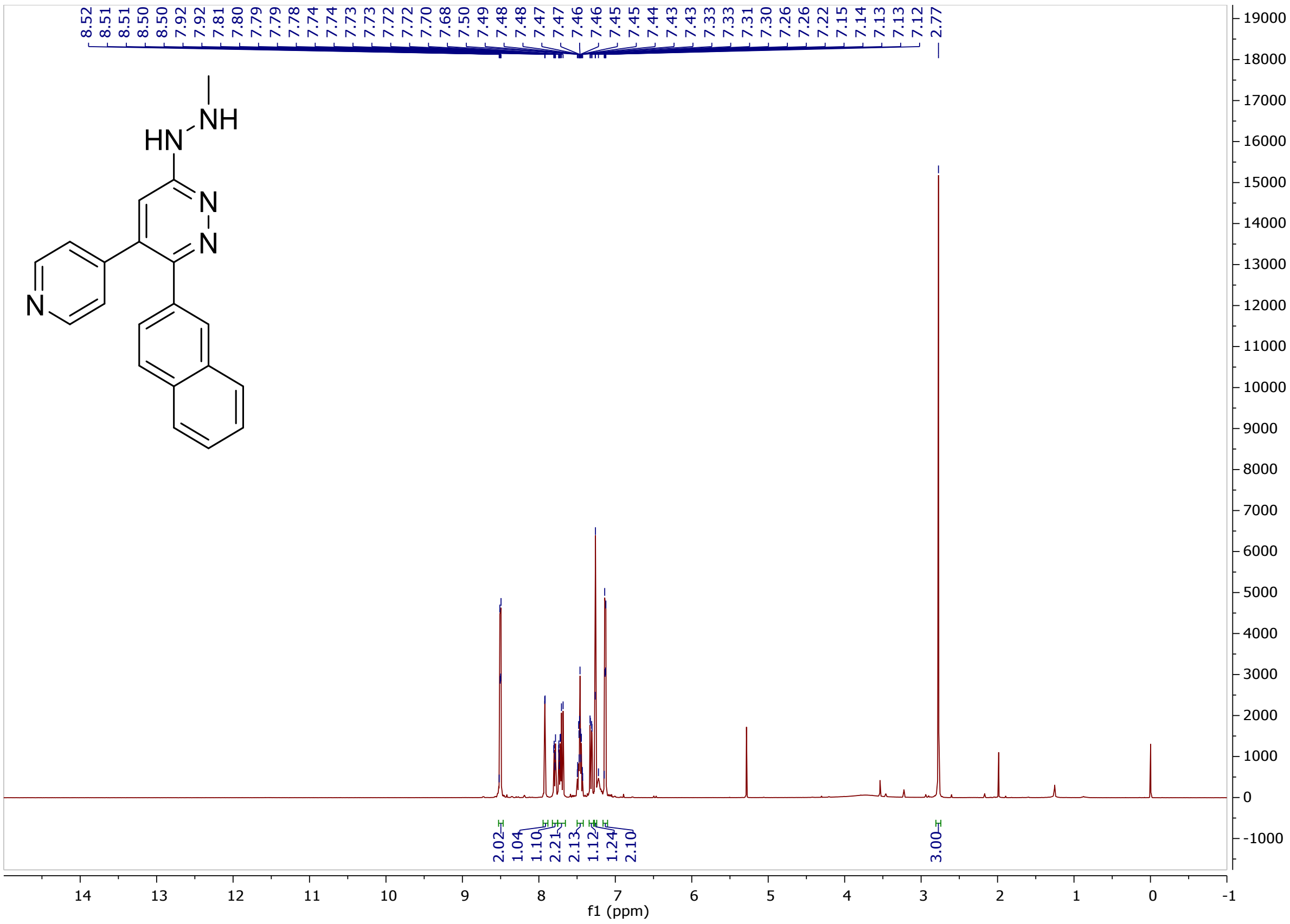

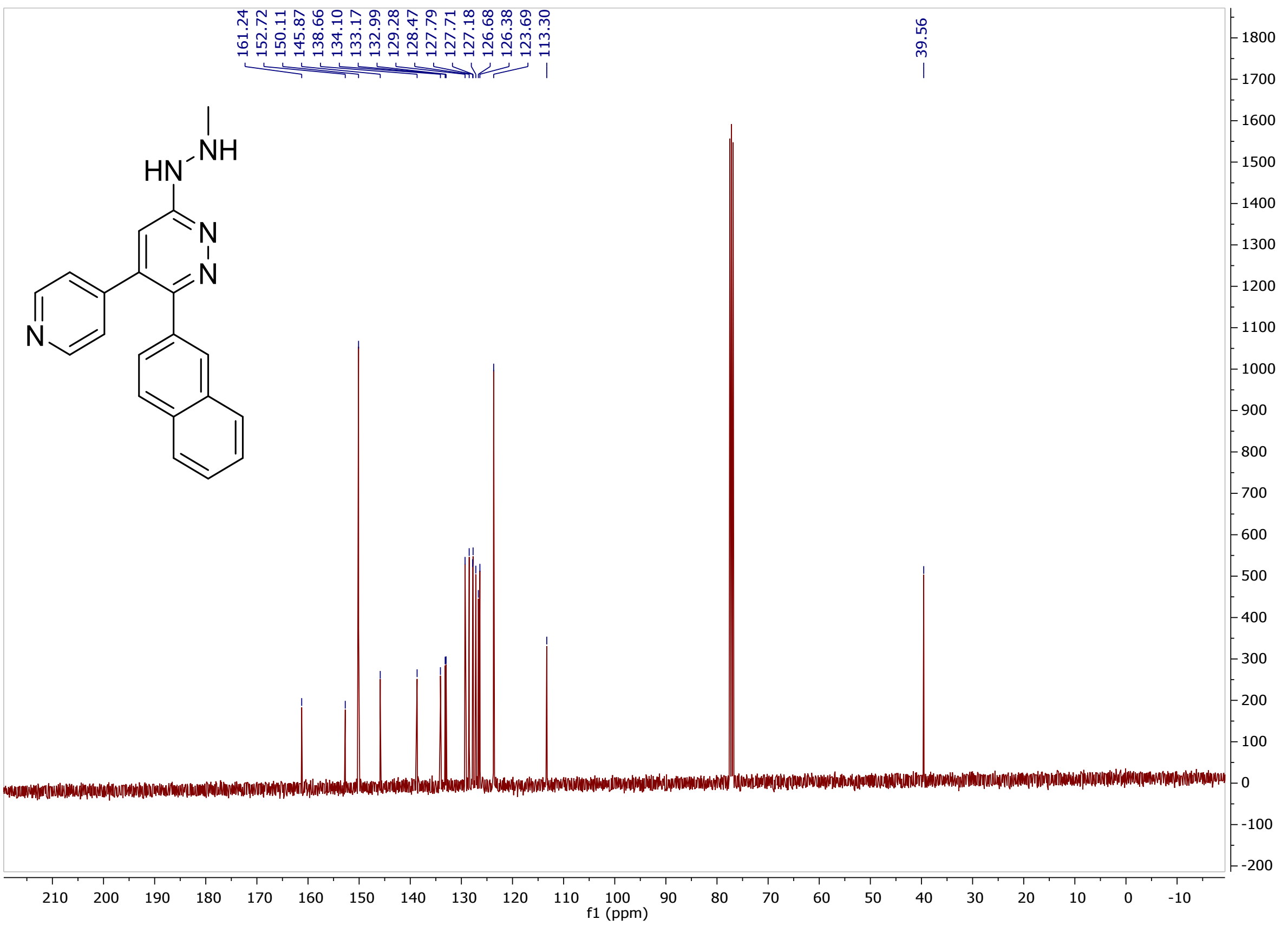

3-(Naphthalen-2-yl)-4-(pyridin-4-yl)-6-vinylpyridazine **19**

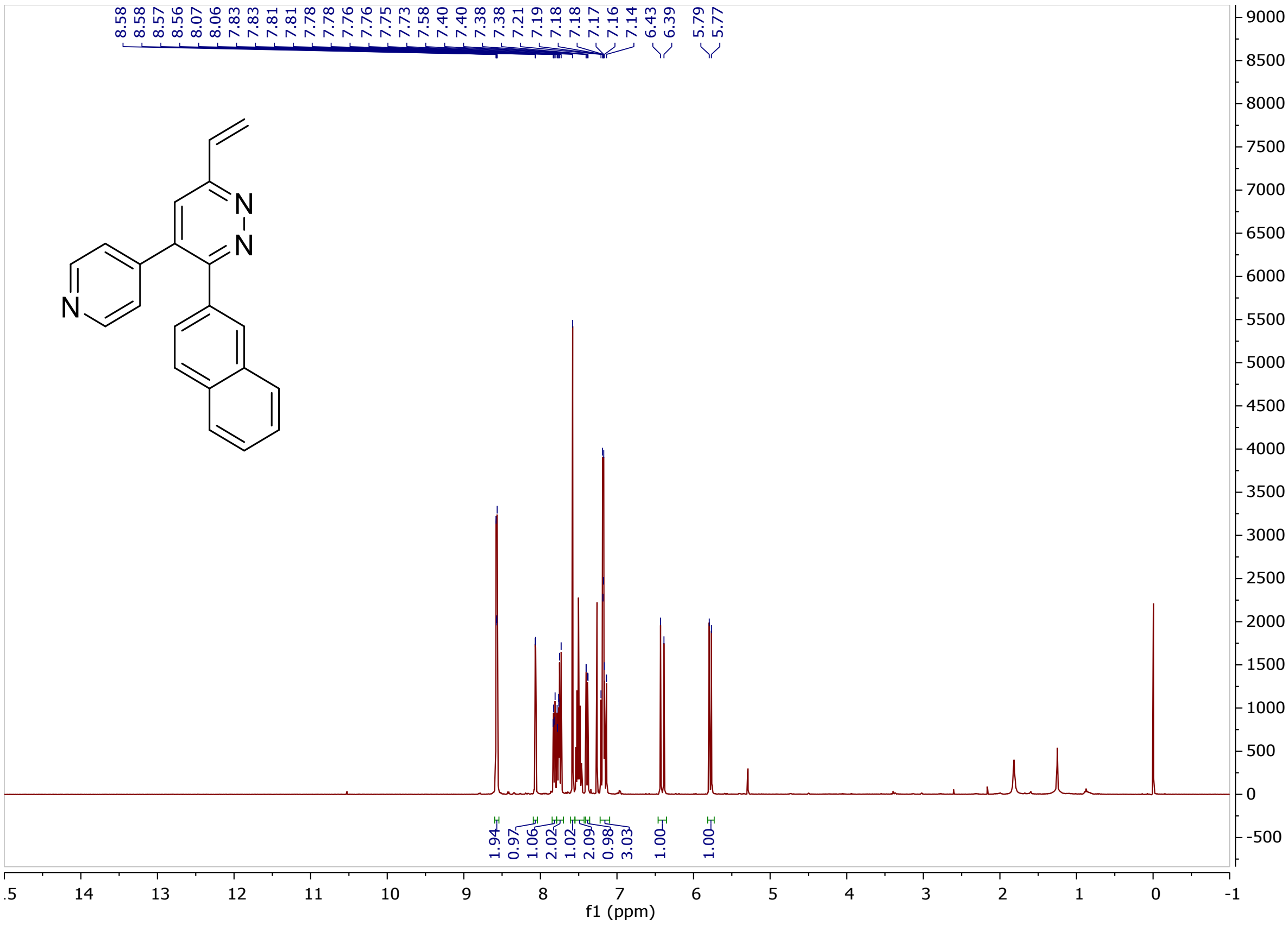

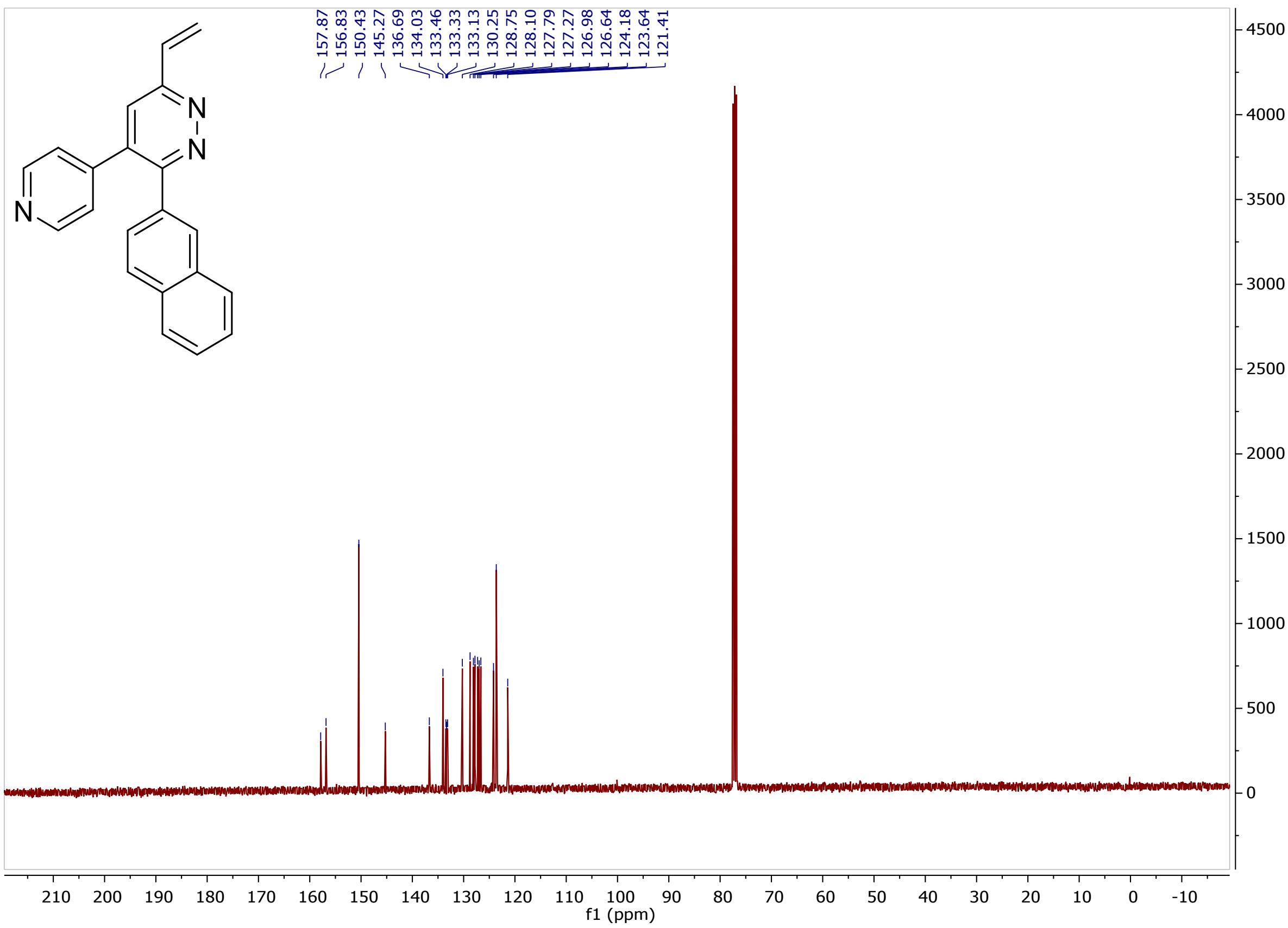

N,*N*-Dimethyl-2-(6-(naphthalen-2-yl)-5-(pyridin-4-yl)pyridazin-3-yl)ethan-1-amine **20**

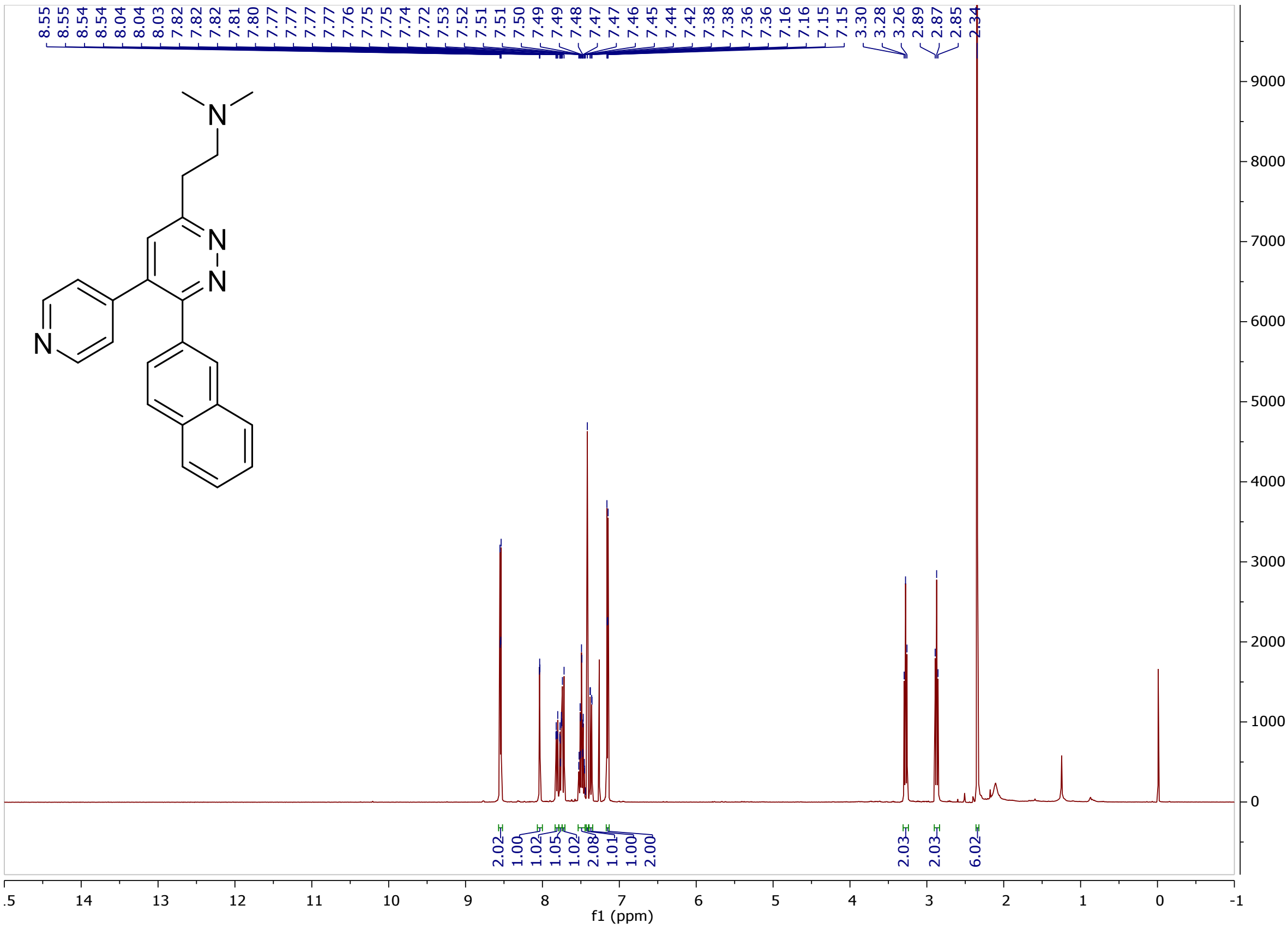

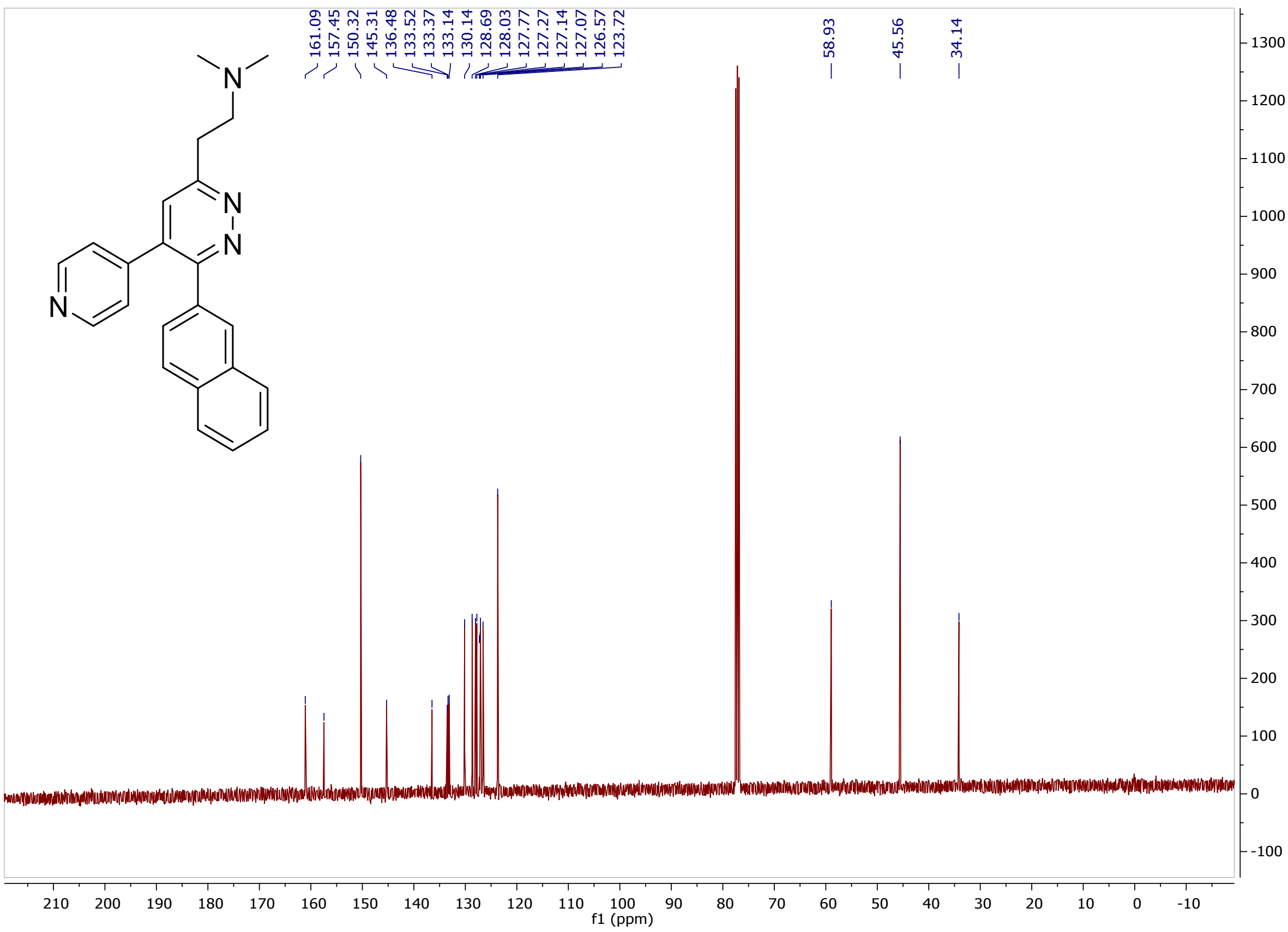

N^1,N^1-Dimethyl-N^2-(6-(naphthalen-1-yl)-5-(pyridin-4-yl)pyridazin-3-yl)ethane-1,2-diamine **21**

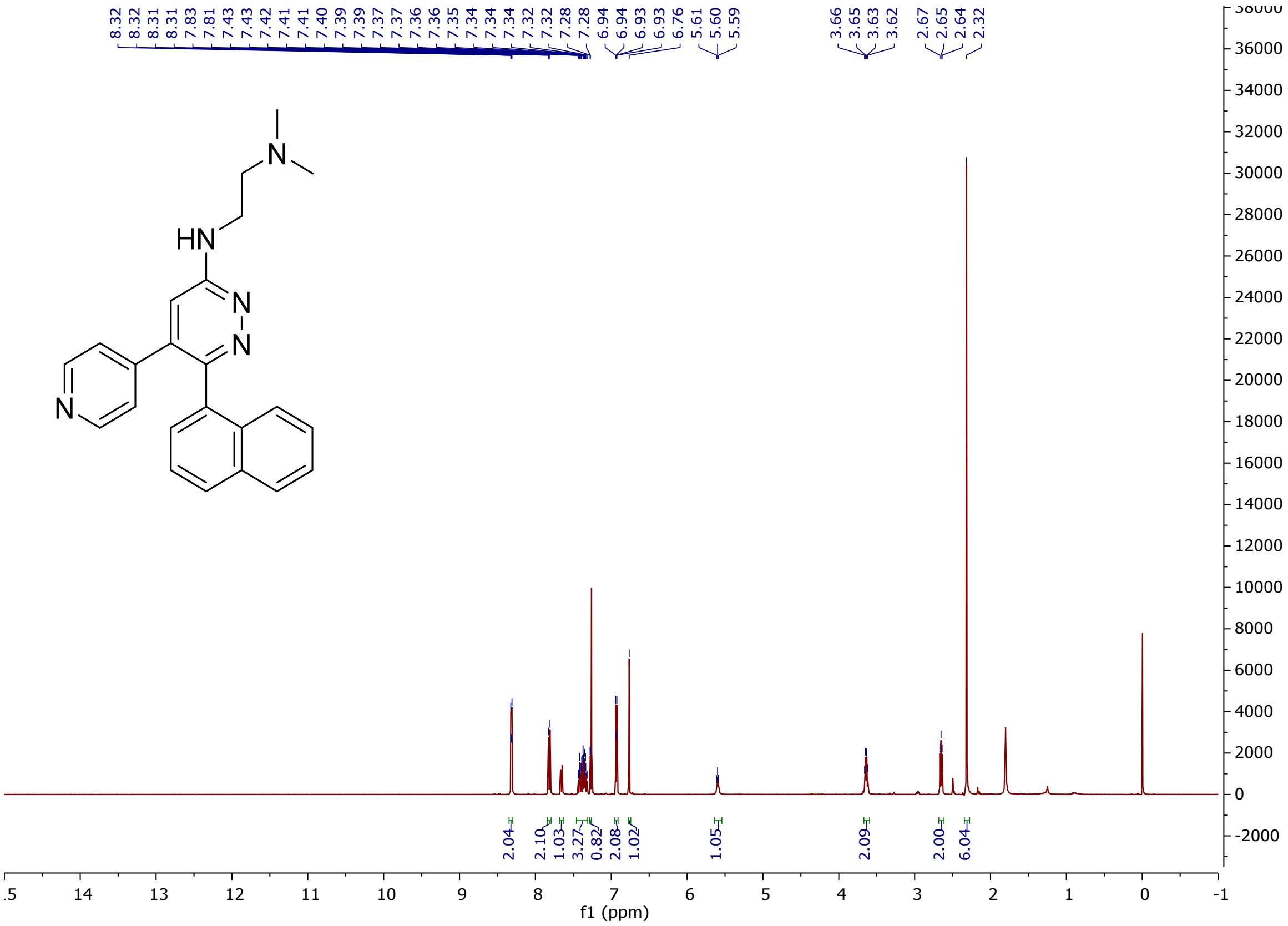

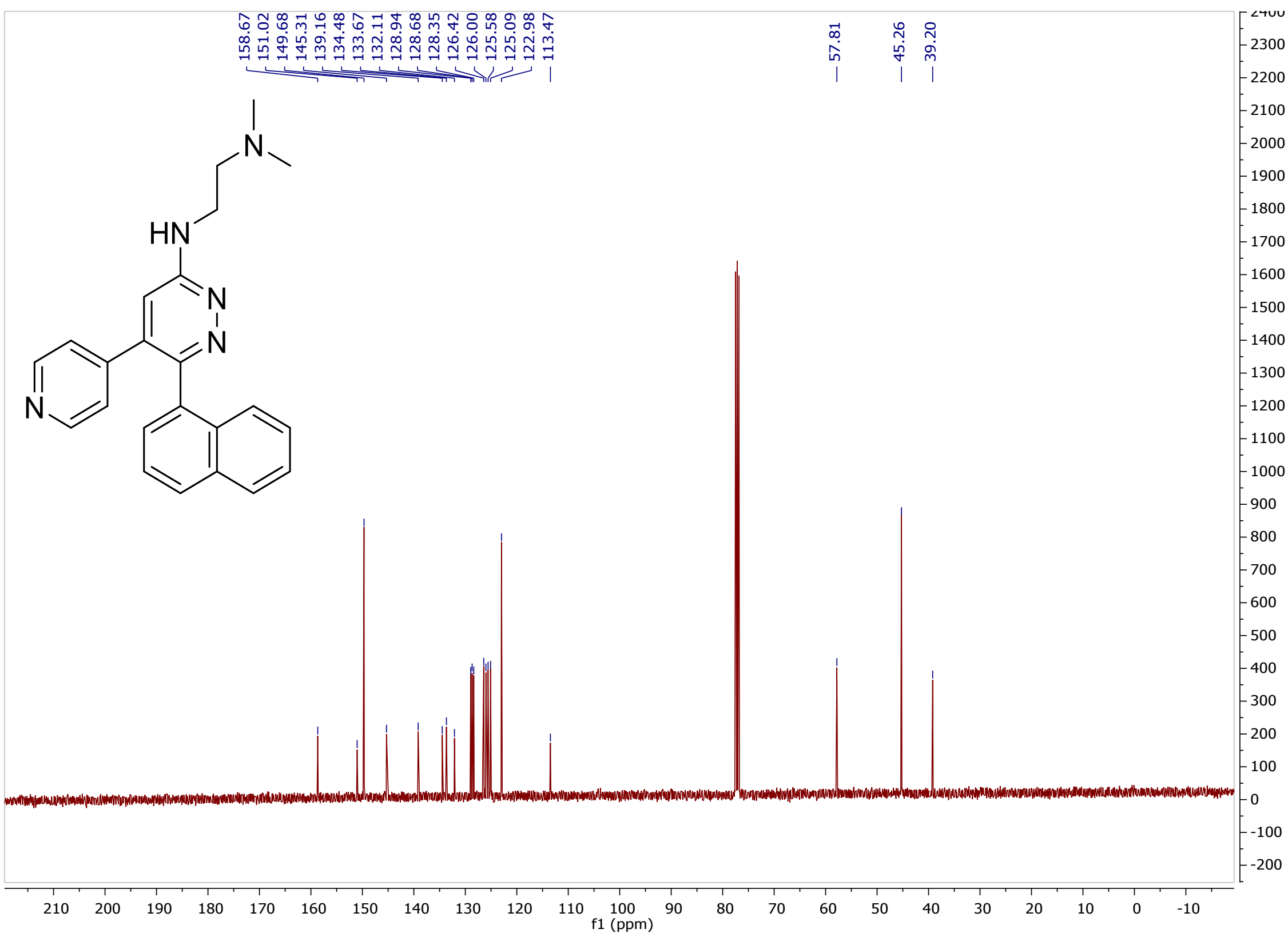

N,*N*-Dimethyl-6-(naphthalen-1-yl)-5-(pyridin-4-yl)pyridazin-3-amine **22**

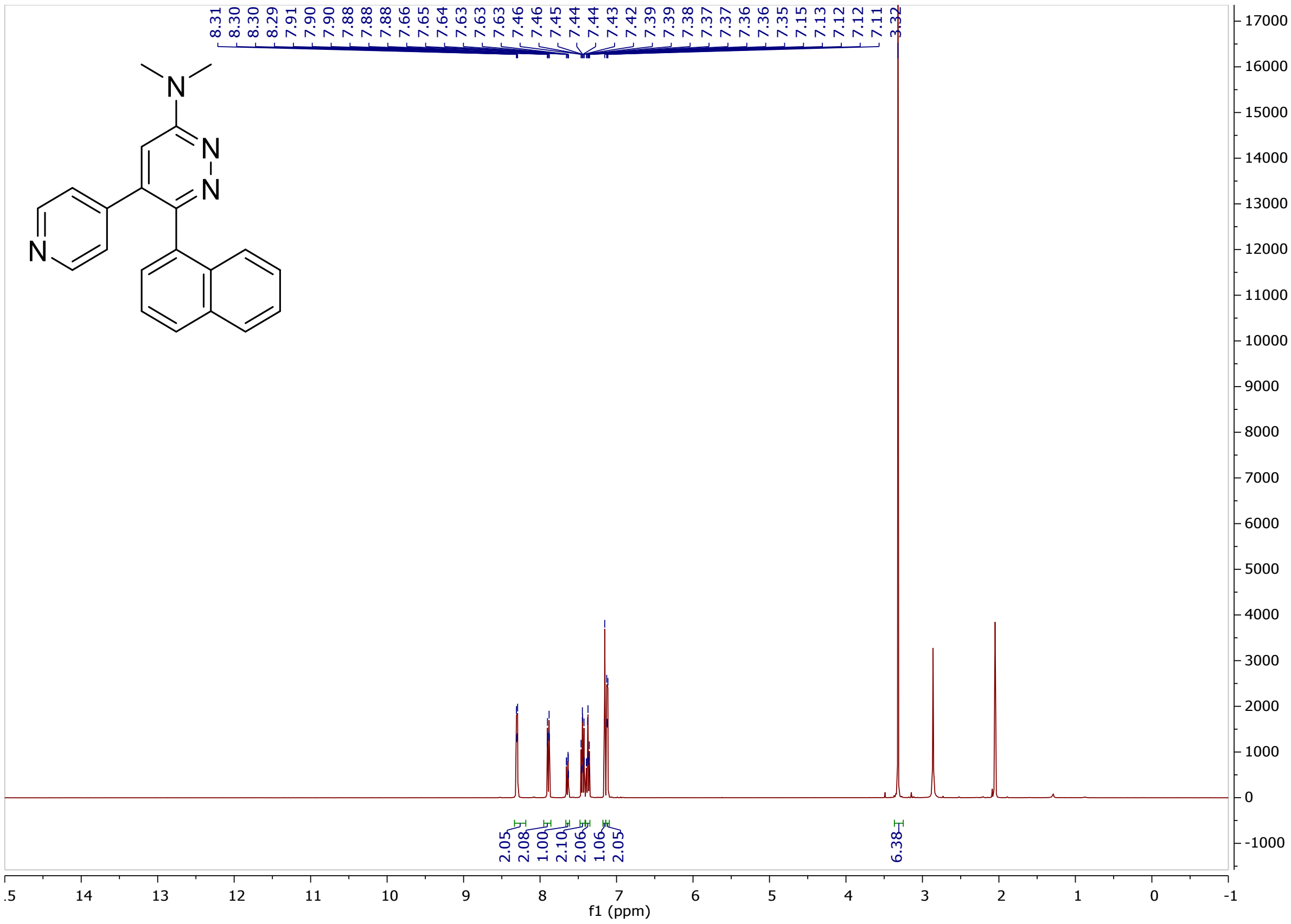

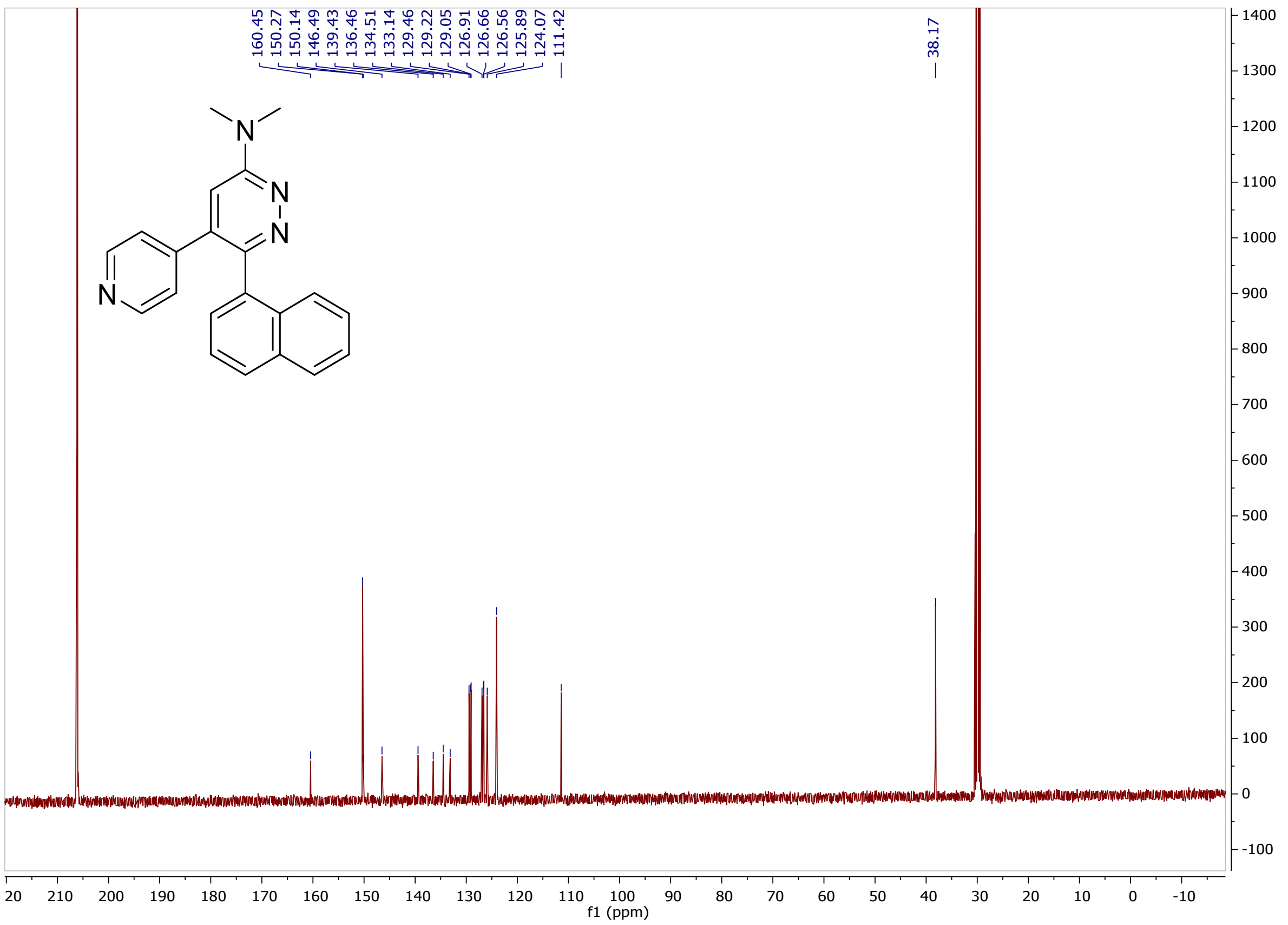

4-Bromo-1,2-dihydropyridazine-3,6-dione **23**

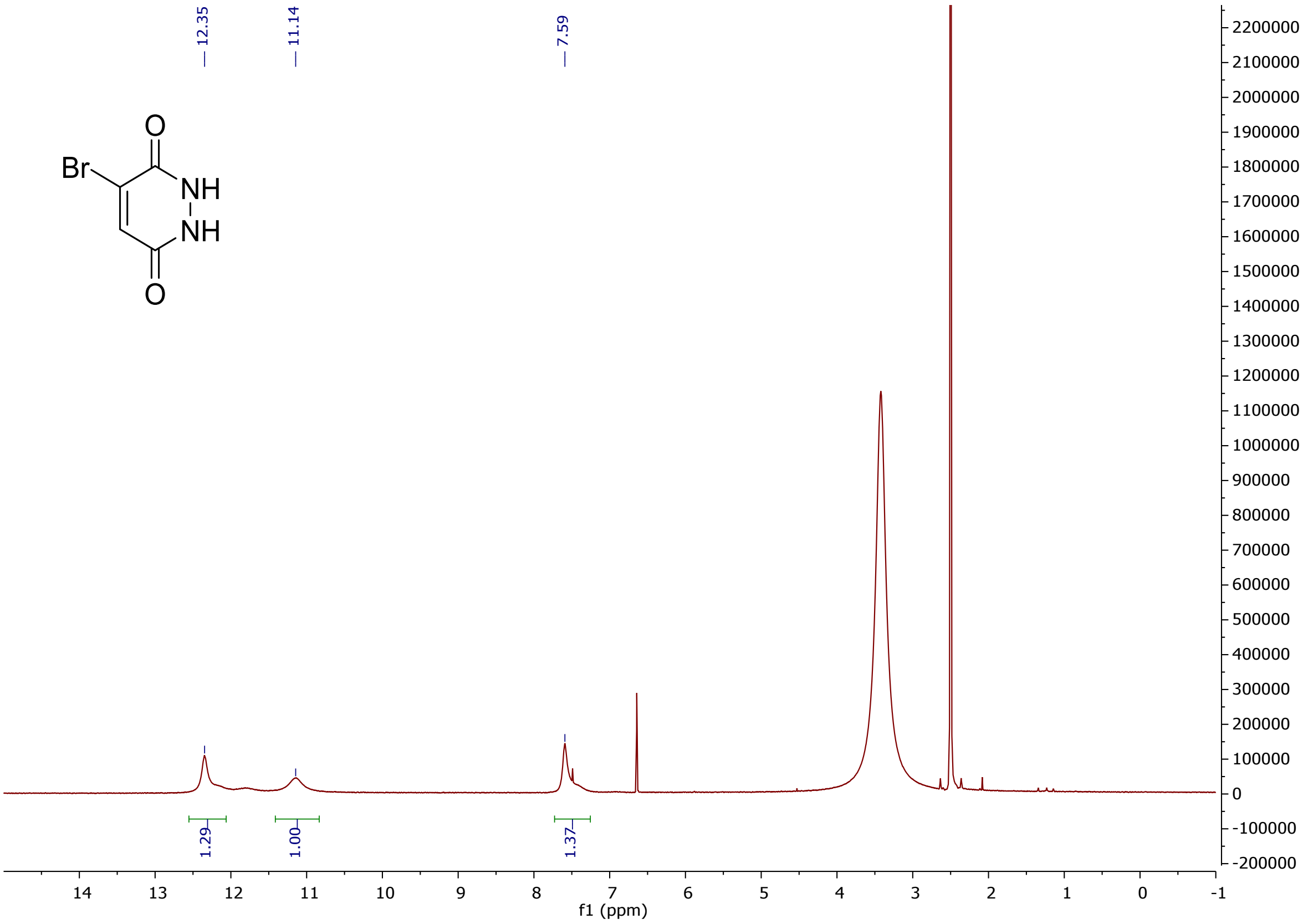

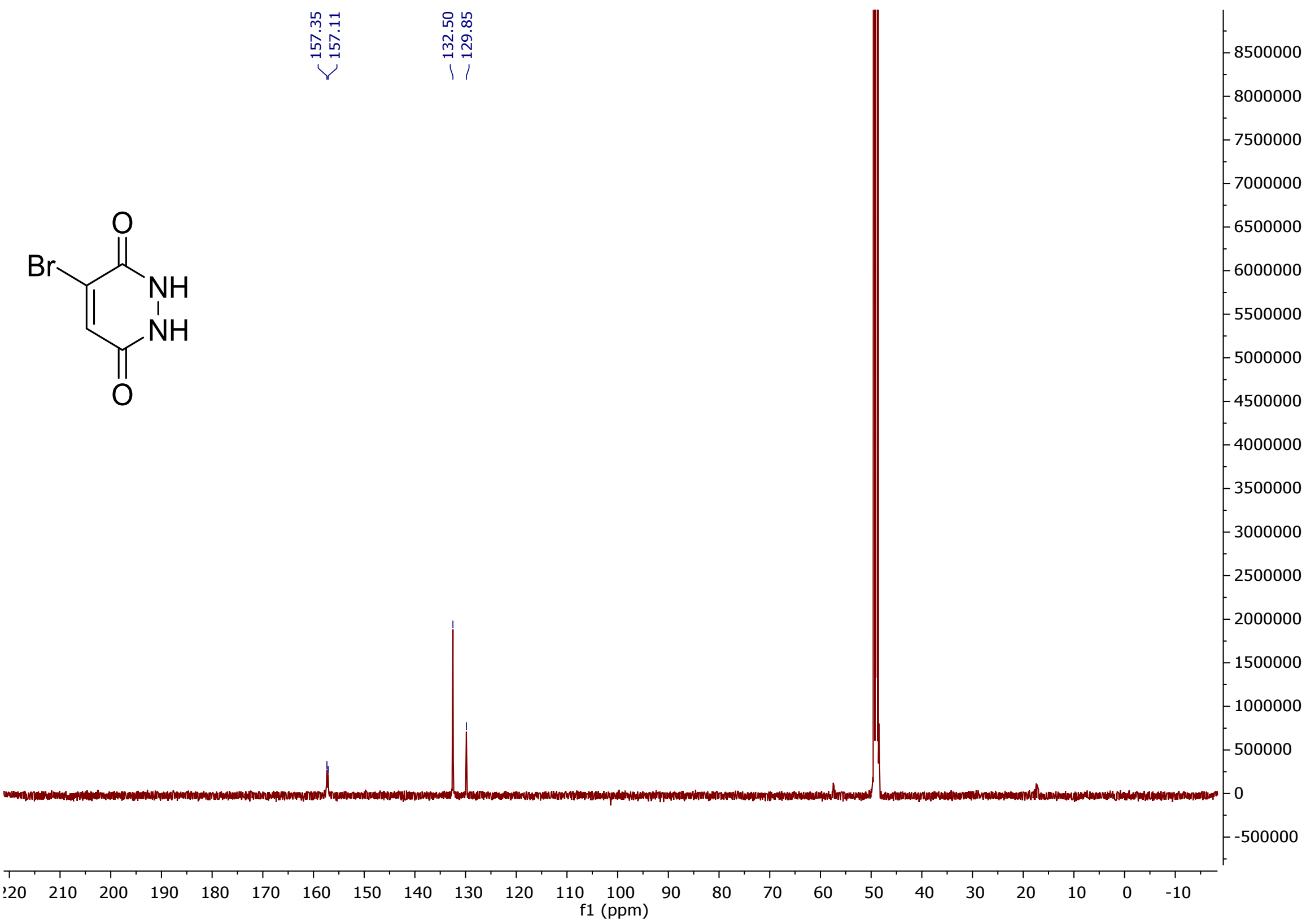

3,6-Dichloro-4-(pyridin-4-yl)pyridazine **24**

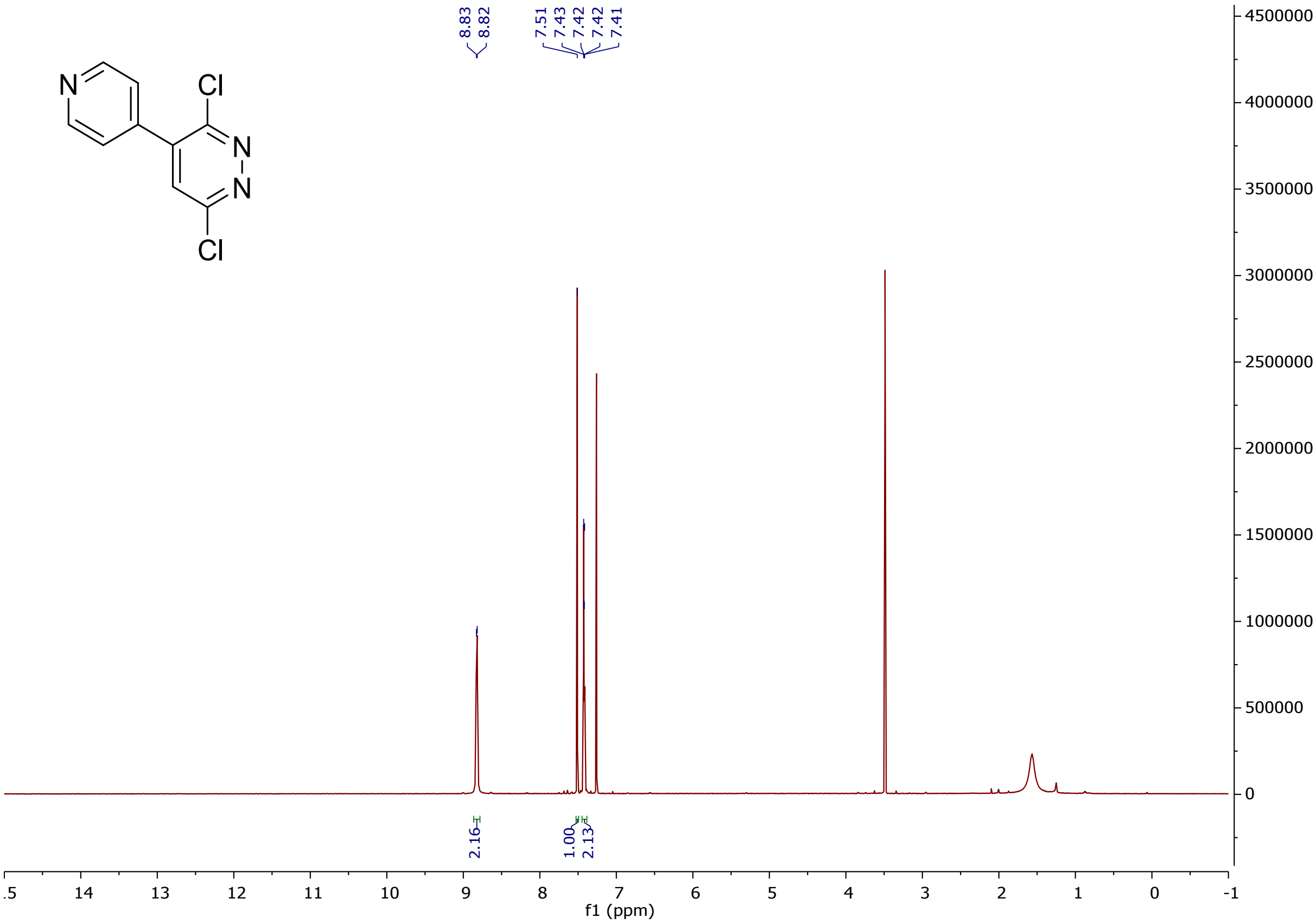

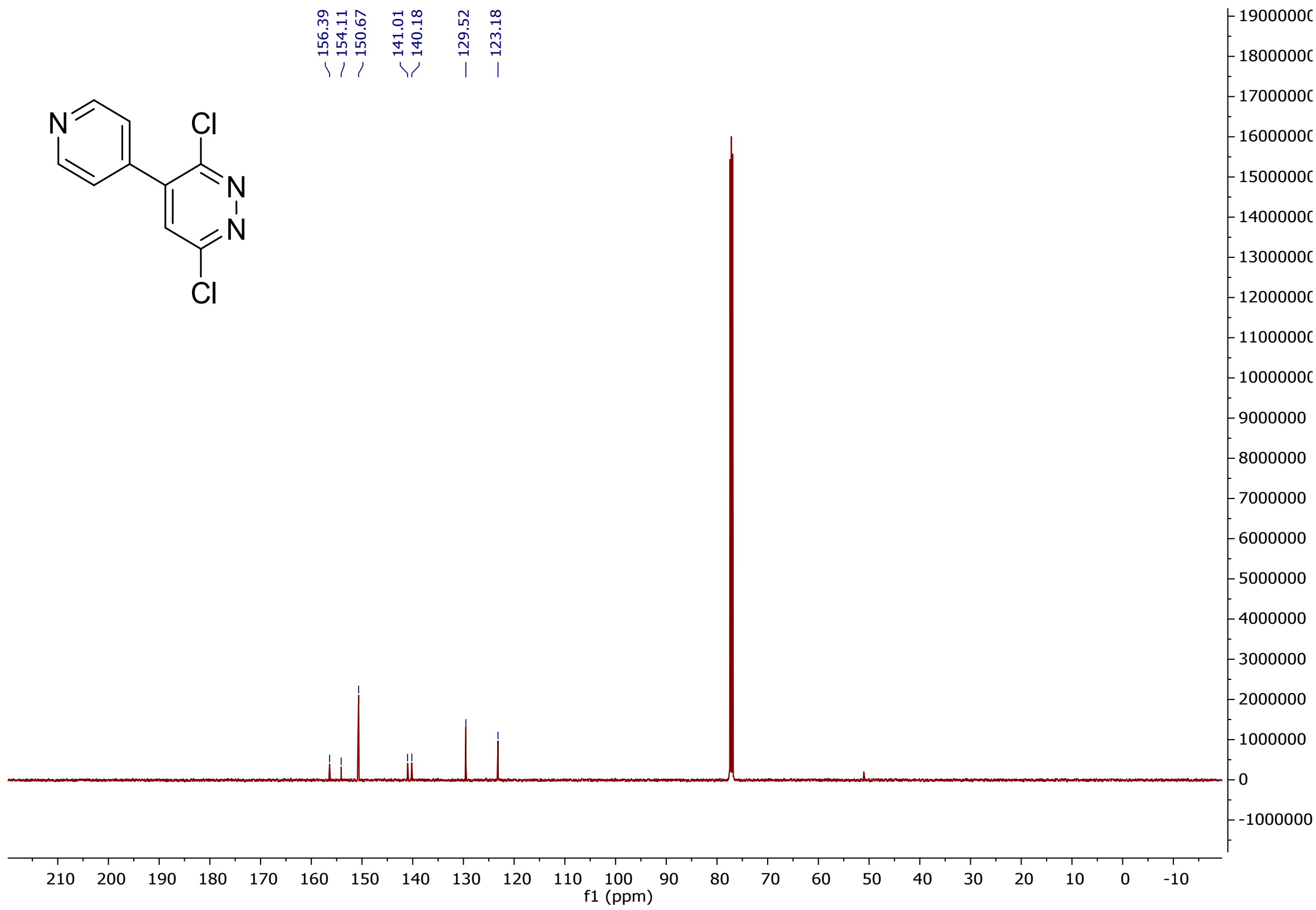

N^1-(6-Chloro-5-(pyridin-4-yl)pyridazin-3-yl)-N^2,N^2-dimethylethane-1,2-diamine **25**

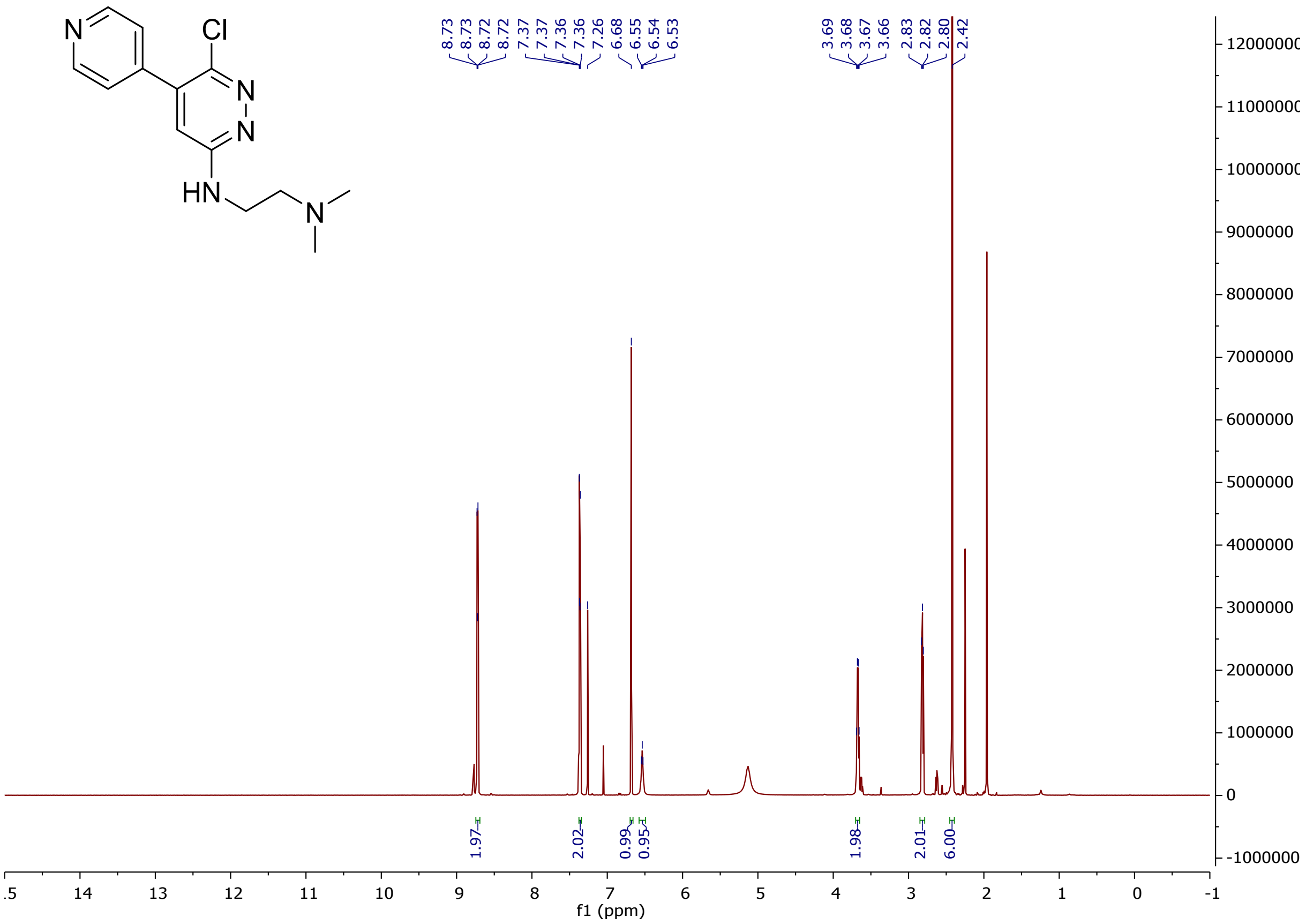

3. HPLC chromatograms

6-(4-Methylpiperazin-1-yl)-3-(naphthalen-2-yl)-4-(pyridin-4-yl)pyridazine 6 (MW150)

Chromatogram

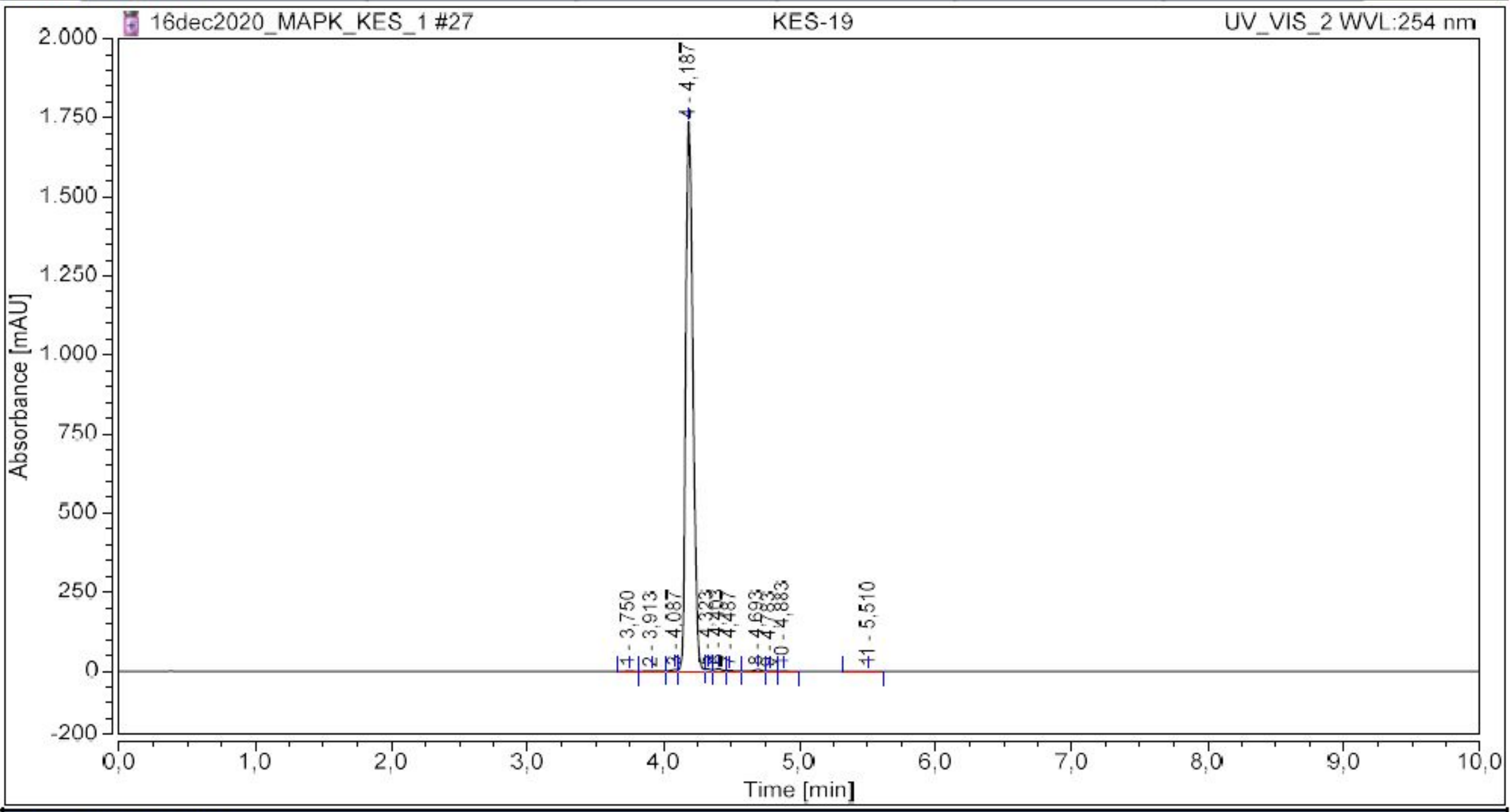

Integration Results

No.	Peak Name	Retention Time min	Area mAU*min	Height mAU	Relative Area %	Relative Height %	Amount n.a.
1		3,750	0,033	0,421	0,03	0,02	n.a.
2		3,913	0,140	0,997	0,13	0,06	n.a.
3		4,087	0,258	5,221	0,24	0,30	n.a.
4		4,187	106,777	1738,022	98,40	98,35	n.a.
5		4,323	0,335	6,495	0,31	0,37	n.a.
6		4,403	0,458	7,726	0,42	0,44	n.a.
7		4,487	0,122	1,640	0,11	0,09	n.a.
8		4,693	0,298	5,189	0,27	0,29	n.a.
9		4,783	0,043	0,726	0,04	0,04	n.a.
10		4,883	0,031	0,512	0,03	0,03	n.a.
11		5,510	0,021	0,253	0,02	0,01	n.a.
**Total:**			**108,517**	**1767,201**	**100,00**	**100,00**	

***N,N*-Dimethyl-6-(naphthalen-2-yl)-5-(pyridin-4-yl)pyridazin-3-amine 7 (MW181)**

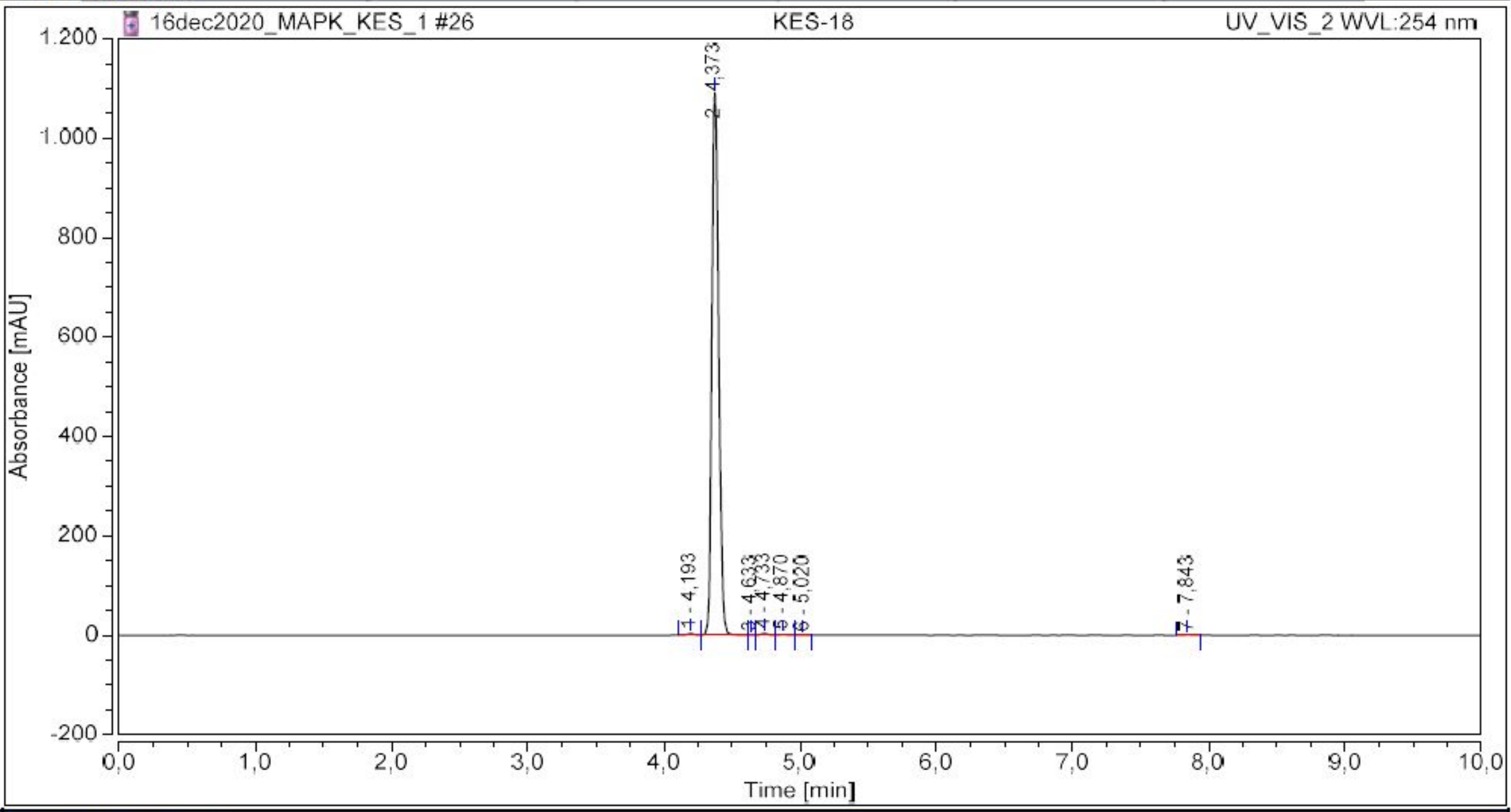

Integration Results

No.	Peak Name	Retention Time min	Area mAU*min	Height mAU	Relative Area %	Relative Height %	Amount n.a.
1		4,193	0,209	3,811	0,32	0,35	n.a.
2		4,373	63,860	1091,523	99,02	98,95	n.a.
3		4,633	0,015	0,286	0,02	0,03	n.a.
4		4,733	0,214	3,901	0,33	0,35	n.a.
5		4,870	0,110	2,055	0,17	0,19	n.a.
6		5,020	0,026	0,540	0,04	0,05	n.a.
7		7,843	0,062	1,005	0,10	0,09	n.a.
**Total:**			**64,495**	**1103,121**	**100,00**	**100,00**	

N^1,N^1-Dimethyl-N^2-(6-(naphthalen-2-yl)-5-(pyridin-4-yl)pyridazin-3-yl)ethane-1,2-diamine 8

Chromatogram

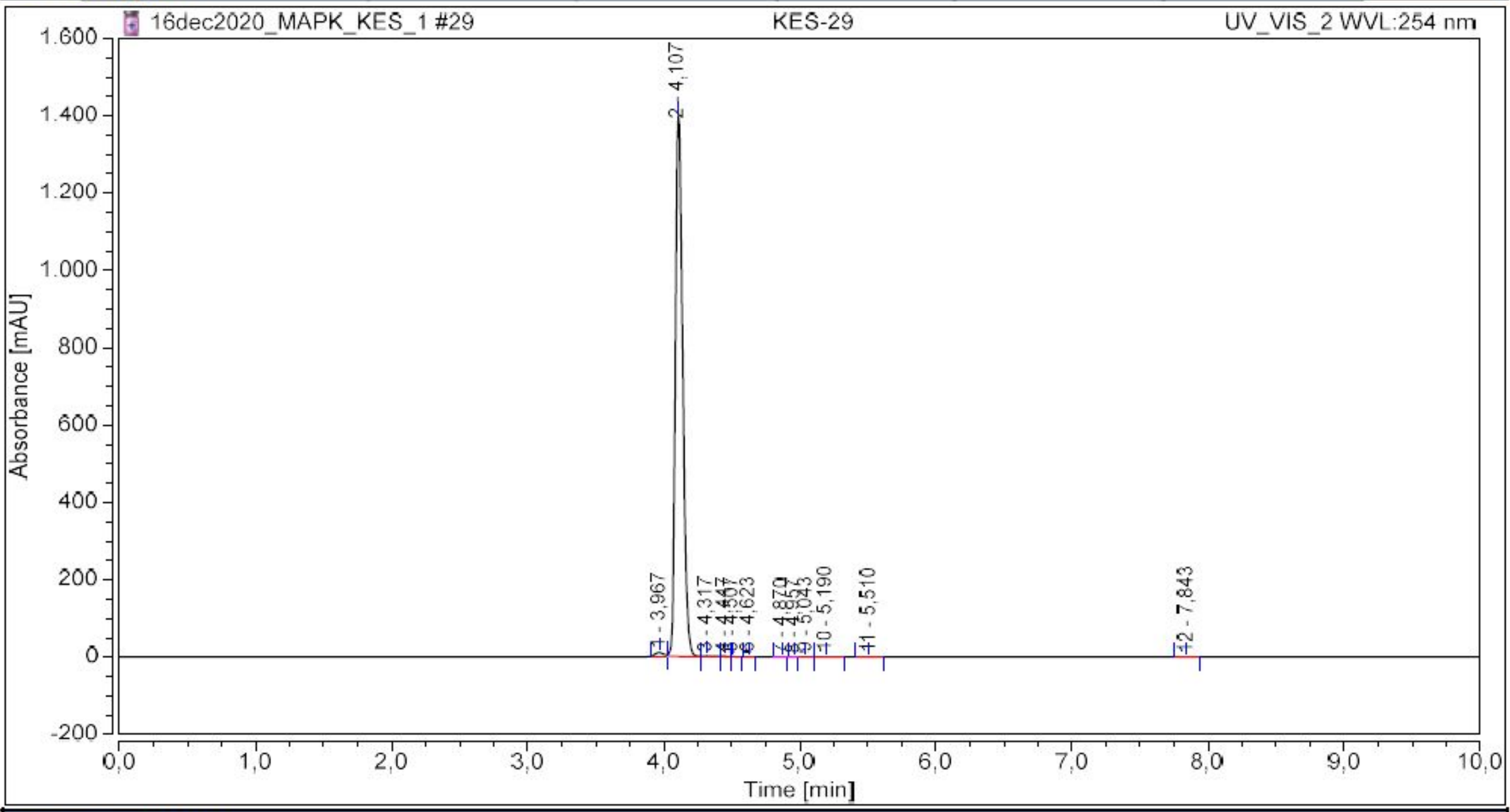

Integration Results

No.	Peak Name	Retention Time min	Area mAU*min	Height mAU	Relative Area %	Relative Height %	Amount n.a.
1		3,967	0,663	9,371	0,76	0,66	n.a.
2		4,107	86,533	1399,062	98,88	99,01	n.a.
3		4,317	0,146	1,609	0,17	0,11	n.a.
4		4,447	0,063	1,152	0,07	0,08	n.a.
5		4,507	0,016	0,402	0,02	0,03	n.a.
6		4,623	0,020	0,495	0,02	0,04	n.a.
7		4,870	0,003	0,050	0,00	0,00	n.a.
8		4,957	0,001	0,033	0,00	0,00	n.a.
9		5,043	0,023	0,377	0,03	0,03	n.a.
10		5,190	0,017	0,184	0,02	0,01	n.a.
11		5,510	0,015	0,205	0,02	0,01	n.a.
12		7,843	0,013	0,179	0,01	0,01	n.a.
**Total:**			**87,513**	**1413,119**	**100,00**	**100,00**	

N^1,N^1-Diethyl-N^2-(6-(naphthalen-2-yl)-5-(pyridin-4-yl)pyridazin-3-yl)ethane-1,2-diamine 9

Chromatogram

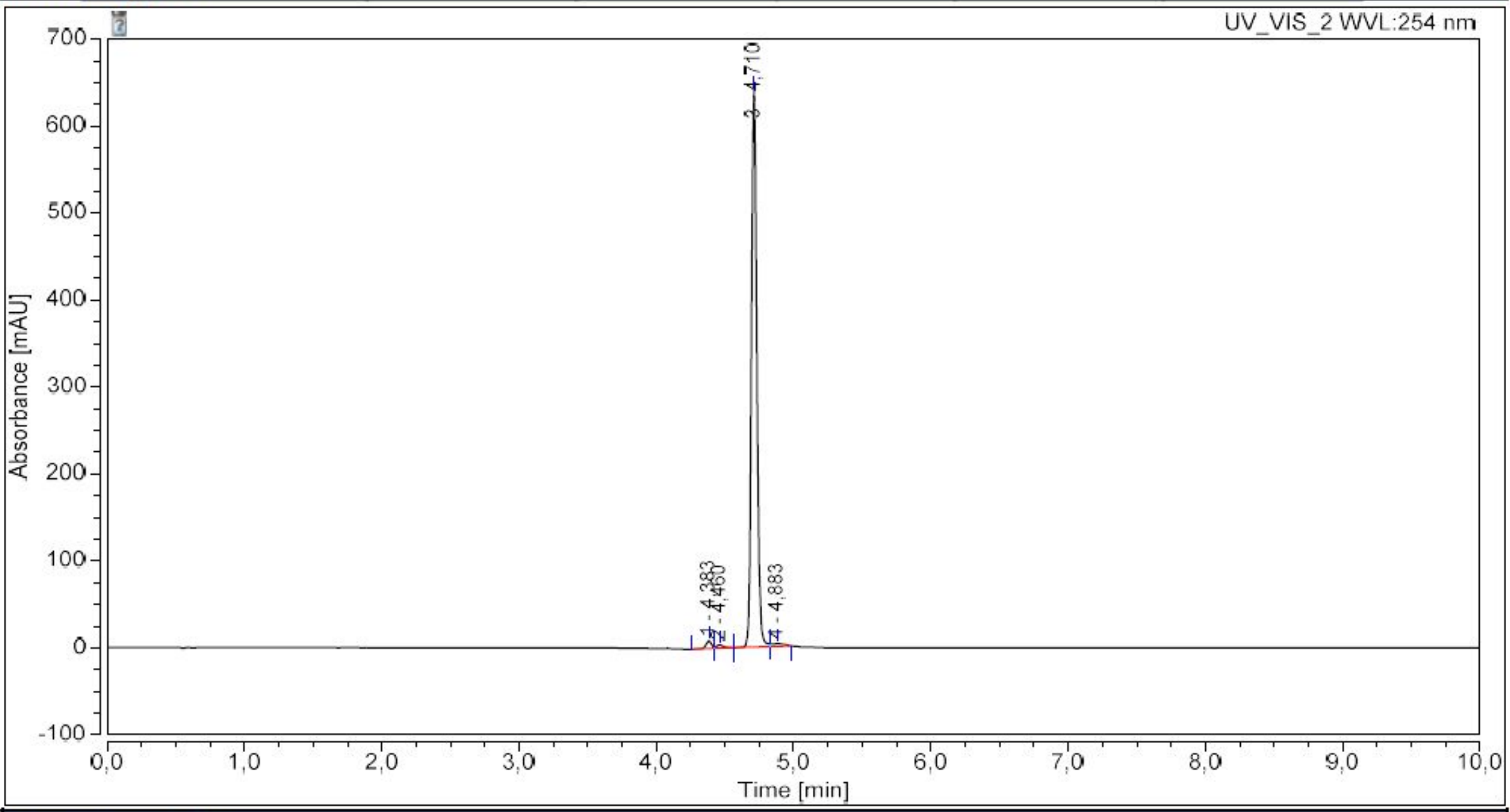

Integration Results

No.	Peak Name	Retention Time min	Area mAU*min	Height mAU	Relative Area %	Relative Height %	Amount n.a.
1		4,383	0,423	9,165	1,42	1,40	n.a.
2		4,460	0,230	4,014	0,77	0,61	n.a.
3		4,710	28,919	639,927	96,82	97,55	n.a.
4		4,883	0,297	2,891	0,99	0,44	n.a.
**Total:**			**29,868**	**655,997**	**100,00**	**100,00**	

N^1,N^1-Dimethyl-N^3-(6-(naphthalen-2-yl)-5-(pyridin-4-yl)pyridazin-3-yl)propane-1,3-diamine 10

Chromatogram

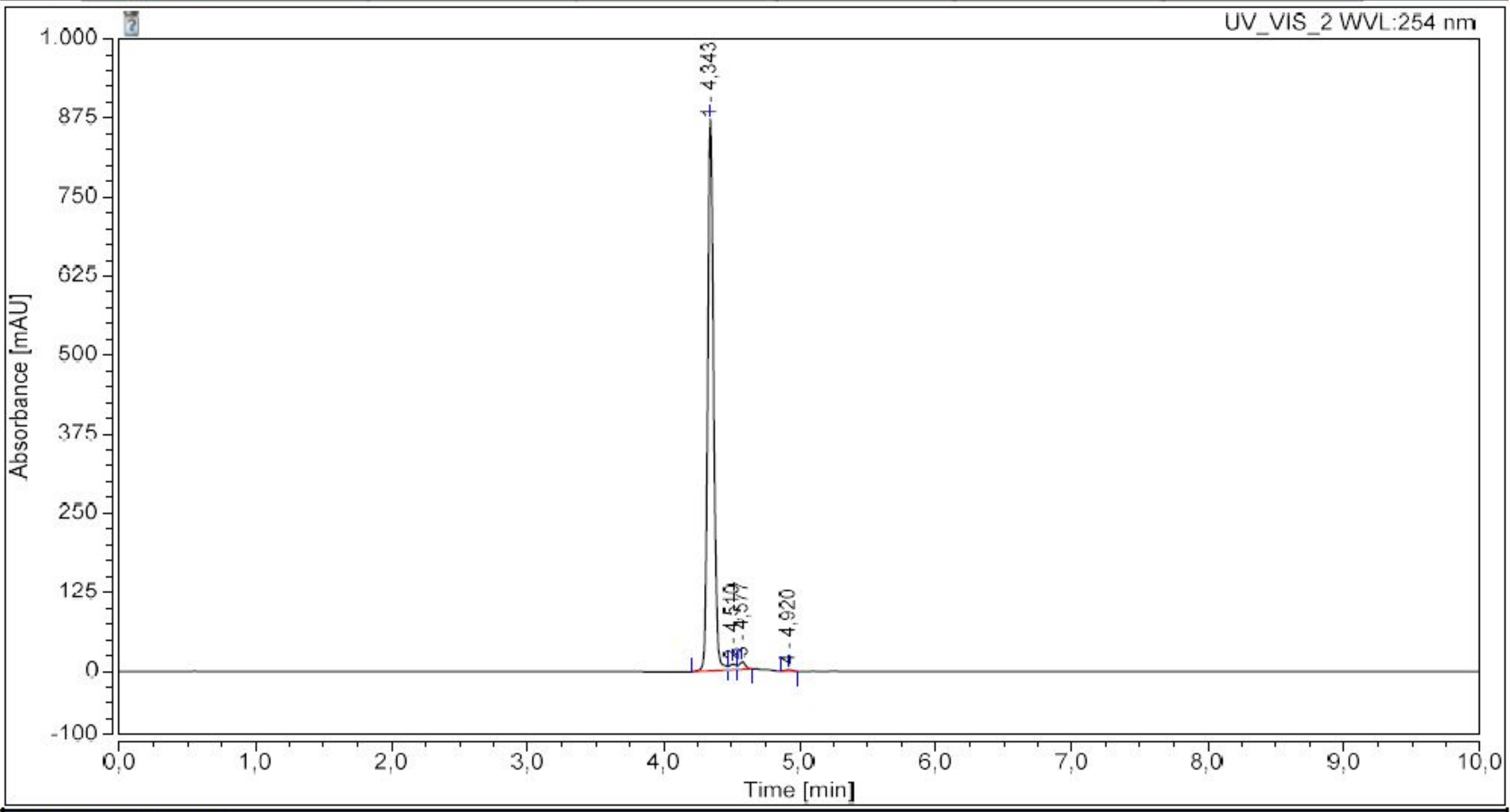

Integration Results

No.	Peak Name	Retention Time min	Area mAU*min	Height mAU	Relative Area %	Relative Height %	Amount n.a.
1		4,343	44,053	872,861	97,06	97,22	n.a.
2		4,510	0,575	9,451	1,27	1,05	n.a.
3		4,577	0,644	12,785	1,42	1,42	n.a.
4		4,920	0,116	2,679	0,26	0,30	n.a.
**Total:**			**45,388**	**897,776**	**100,00**	**100,00**	

N^1,N^2-Dimethyl-N^1-(6-(naphthalen-2-yl)-5-(pyridin-4-yl)pyridazin-3-yl)ethane-1,2-diamine 11

Chromatogram

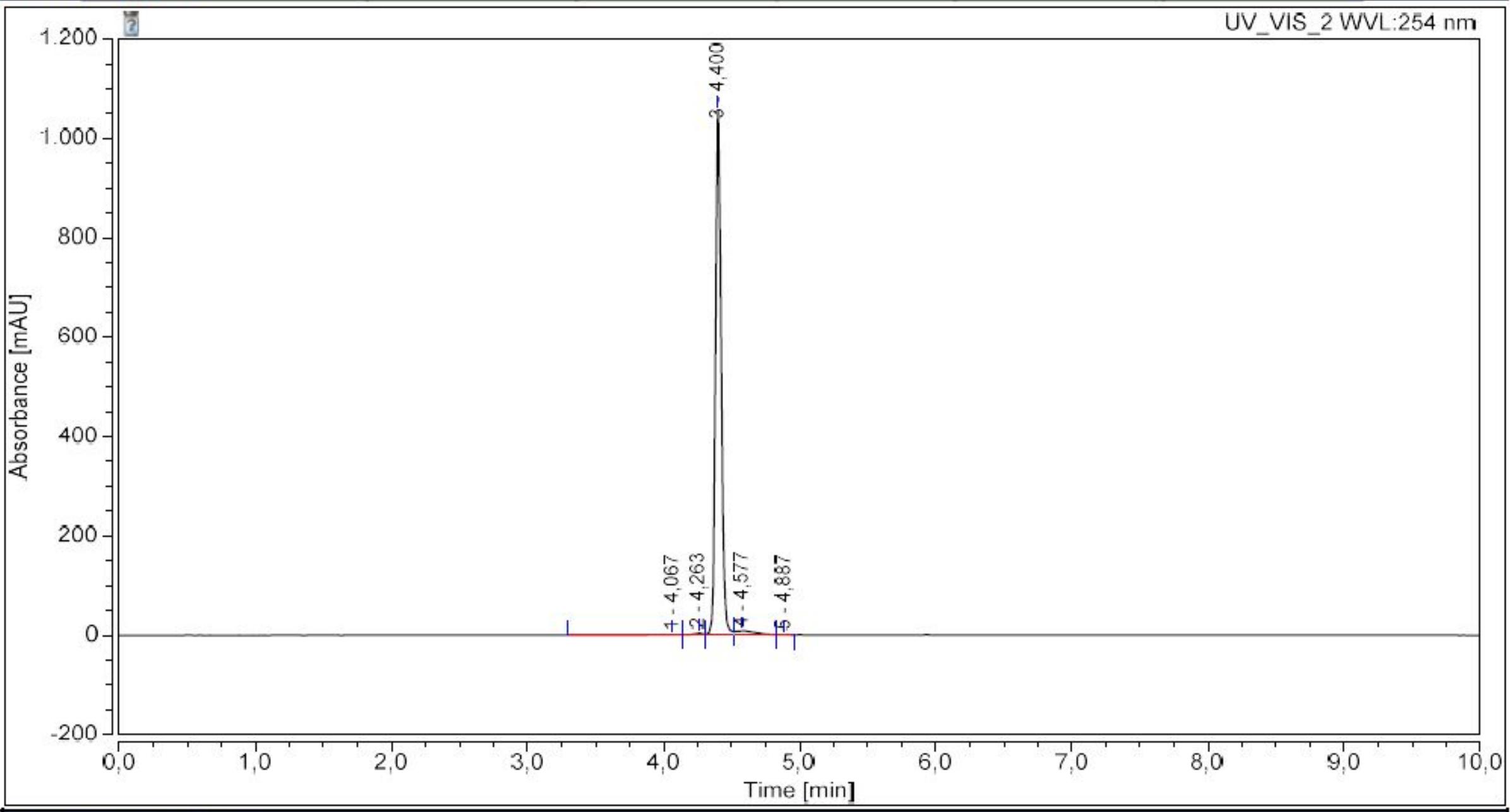

Integration Results

No.	Peak Name	Retention Time min	Area mAU*min	Height mAU	Relative Area %	Relative Height %	Amount n.a.
1		4,067	0,275	0,941	0,51	0,09	n.a.
2		4,263	0,363	3,886	0,67	0,36	n.a.
3		4,400	51,670	1055,537	95,93	98,65	n.a.
4		4,577	1,444	8,228	2,68	0,77	n.a.
5		4,887	0,109	1,358	0,20	0,13	n.a.
**Total:**			**53,860**	**1069,951**	**100,00**	**100,00**	

***N*-Isopentyl-6-(naphthalen-2-yl)-5-(pyridin-4-yl)pyridazin-3-amine 12**

Chromatogram

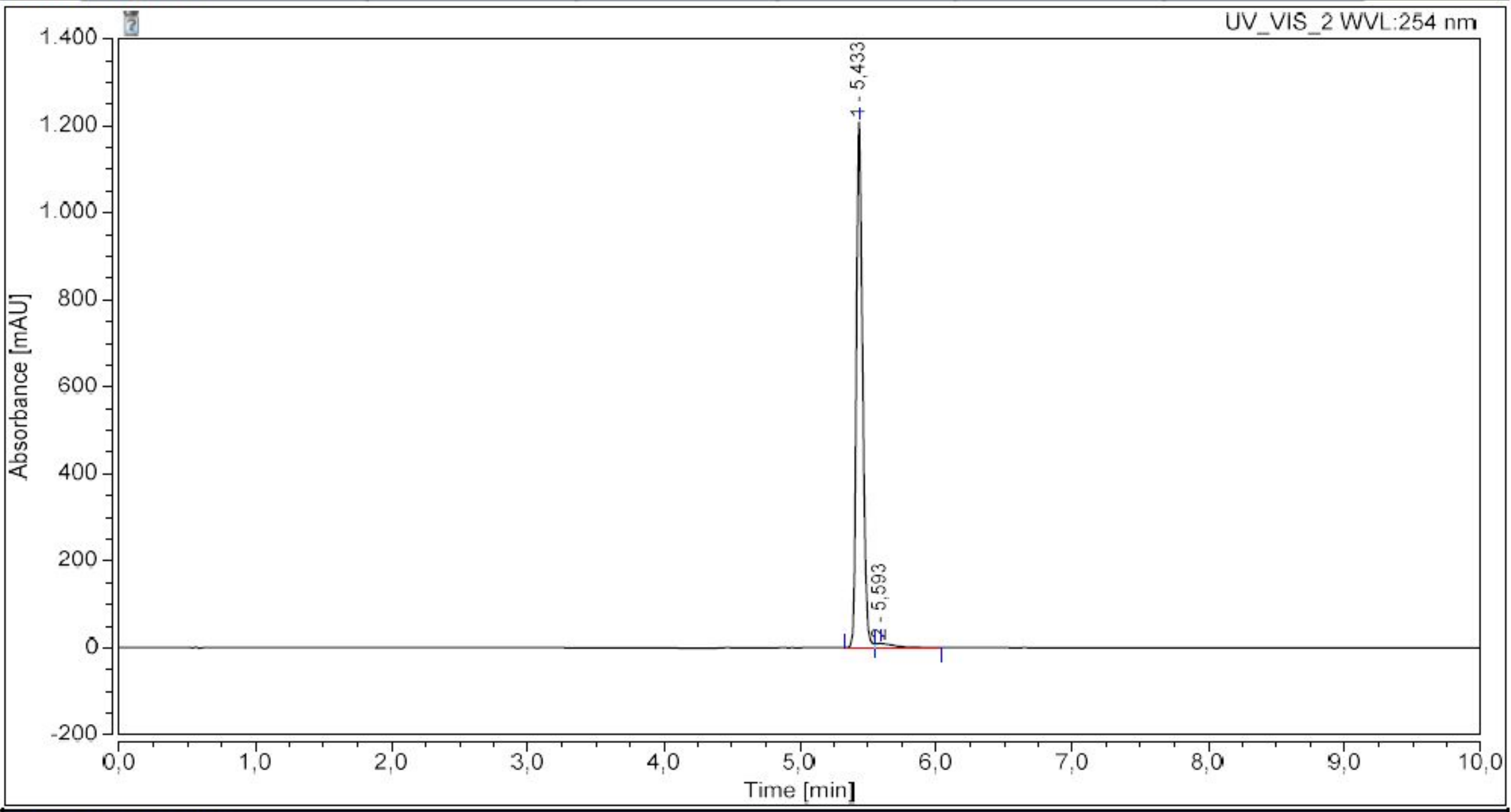

Integration Results

No.	Peak Name	Retention Time min	Area mAU*min	Height mAU	Relative Area %	Relative Height %	Amount n.a.
1		5,433	64,590	1208,986	97,70	99,22	n.a.
2		5,593	1,521	9,560	2,30	0,78	n.a.
**Total:**			**66,111**	**1218,546**	**100,00**	**100,00**	

***N*-Isobutyl-6-(naphthalen-2-yl)-5-(pyridin-4-yl)pyridazin-3-amine 13**

Chromatogram

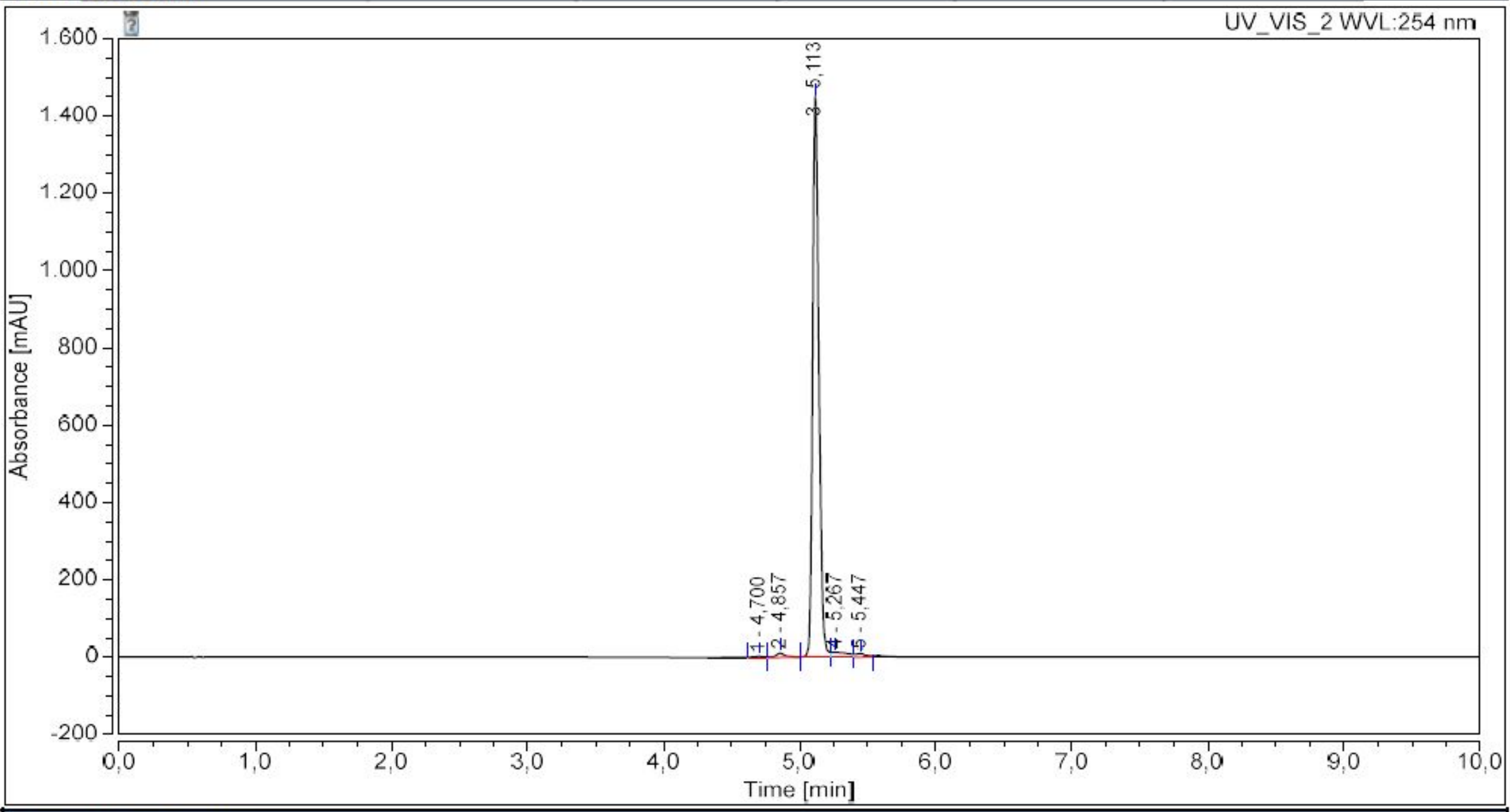

Integration Results

No.	Peak Name	Retention Time min	Area mAU*min	Height mAU	Relative Area %	Relative Height %	Amount n.a.
1		4,700	0,252	3,315	0,31	0,22	n.a.
2		4,857	0,943	11,644	1,16	0,78	n.a.
3		5,113	77,292	1447,422	95,25	97,50	n.a.
4		5,267	1,733	12,574	2,14	0,85	n.a.
5		5,447	0,922	9,625	1,14	0,65	n.a.
**Total:**			**81,143**	**1484,580**	**100,00**	**100,00**	

***N*-Benzyl-6-(naphthalen-2-yl)-5-(pyridin-4-yl)pyridazin-3-amine 14**

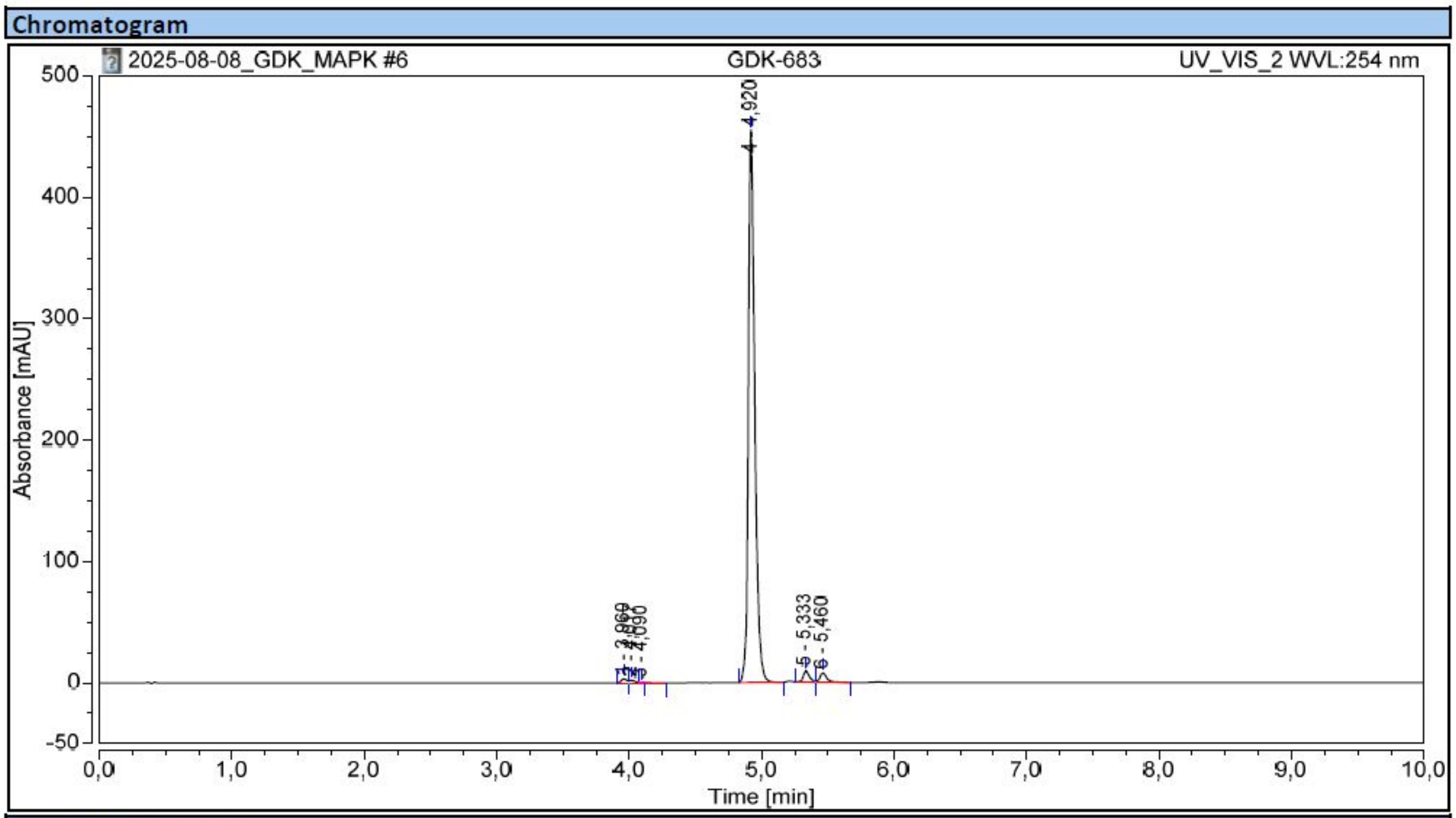

Integration Results

No.	Peak Name	Retention Time min	Area mAU*min	Height mAU	Relative Area %	Relative Height %	Amount n.a.
1		3,960	0,155	3,423	0,58	0,71	n.a.
2		4,017	0,137	2,188	0,51	0,46	n.a.
3		4,090	0,002	0,092	0,01	0,02	n.a.
4		4,920	25,526	455,758	95,39	95,17	n.a.
5		5,333	0,486	9,650	1,82	2,01	n.a.
6		5,460	0,453	7,780	1,69	1,62	n.a.
**Total:**			**26,758**	**478,891**	**100,00**	**100,00**	

***O*-Benzyl-*N*-(6-(naphthalen-2-yl)-5-(pyridin-4-yl)pyridazin-3-yl)hydroxylamine 15**

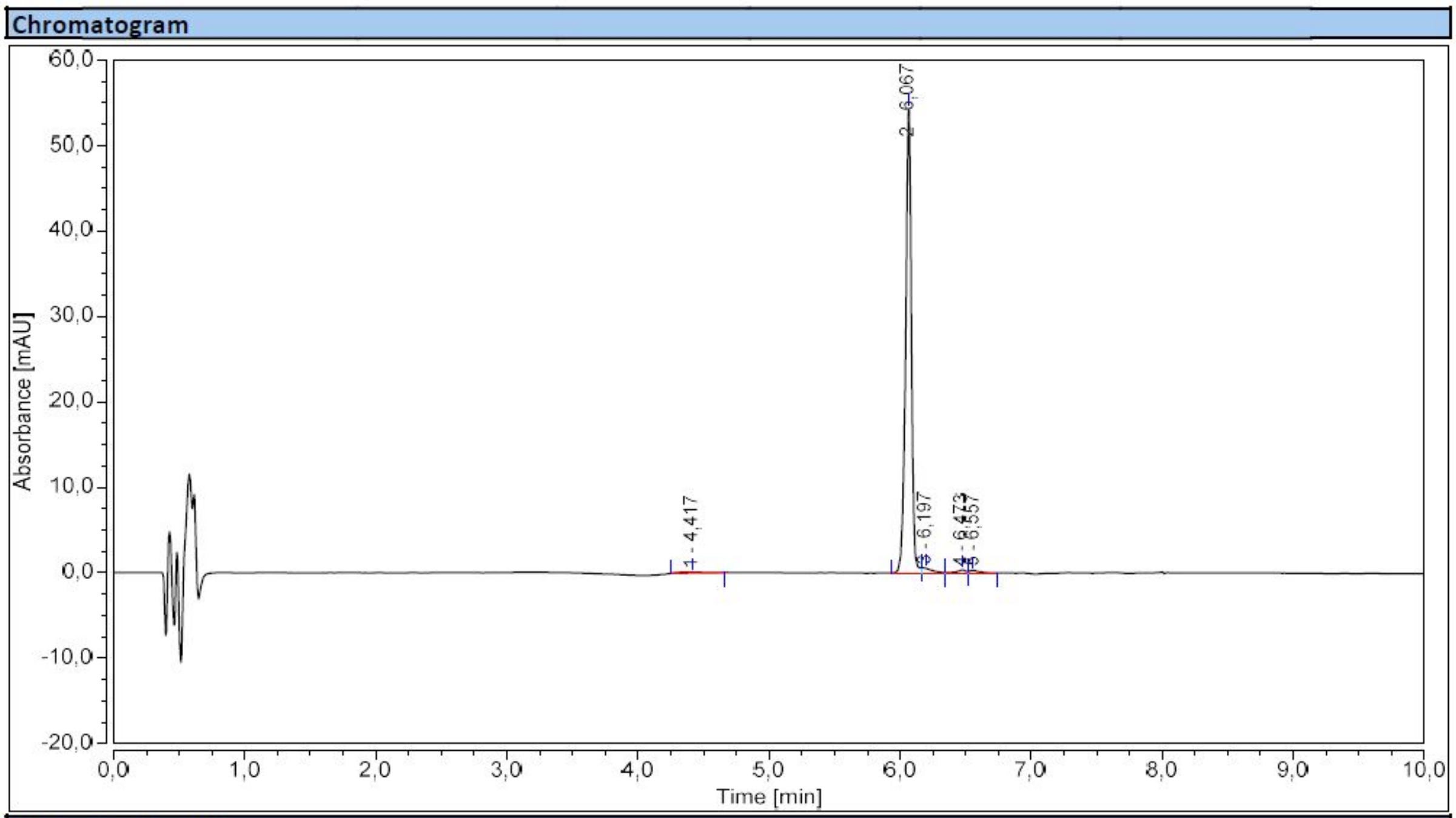

Integration Results

No.	Peak Name	Retention Time min	Area mAU*min	Height mAU	Relative Area %	Relative Height %	Amount n.a.
1		4,417	0,025	0,108	0,87	0,19	n.a.
2		6,067	2,788	54,331	95,42	97,54	n.a.
3		6,197	0,050	0,544	1,72	0,98	n.a.
4		6,473	0,028	0,387	0,97	0,70	n.a.
5		6,557	0,030	0,334	1,03	0,60	n.a.
**Total:**			**2,921**	**55,704**	**100,00**	**100,00**	

***N*,*O*-Dimethyl-*N*-(6-(naphthalen-2-yl)-5-(pyridin-4-yl)pyridazin-3-yl)hydroxylamine 16**

Chromatogram

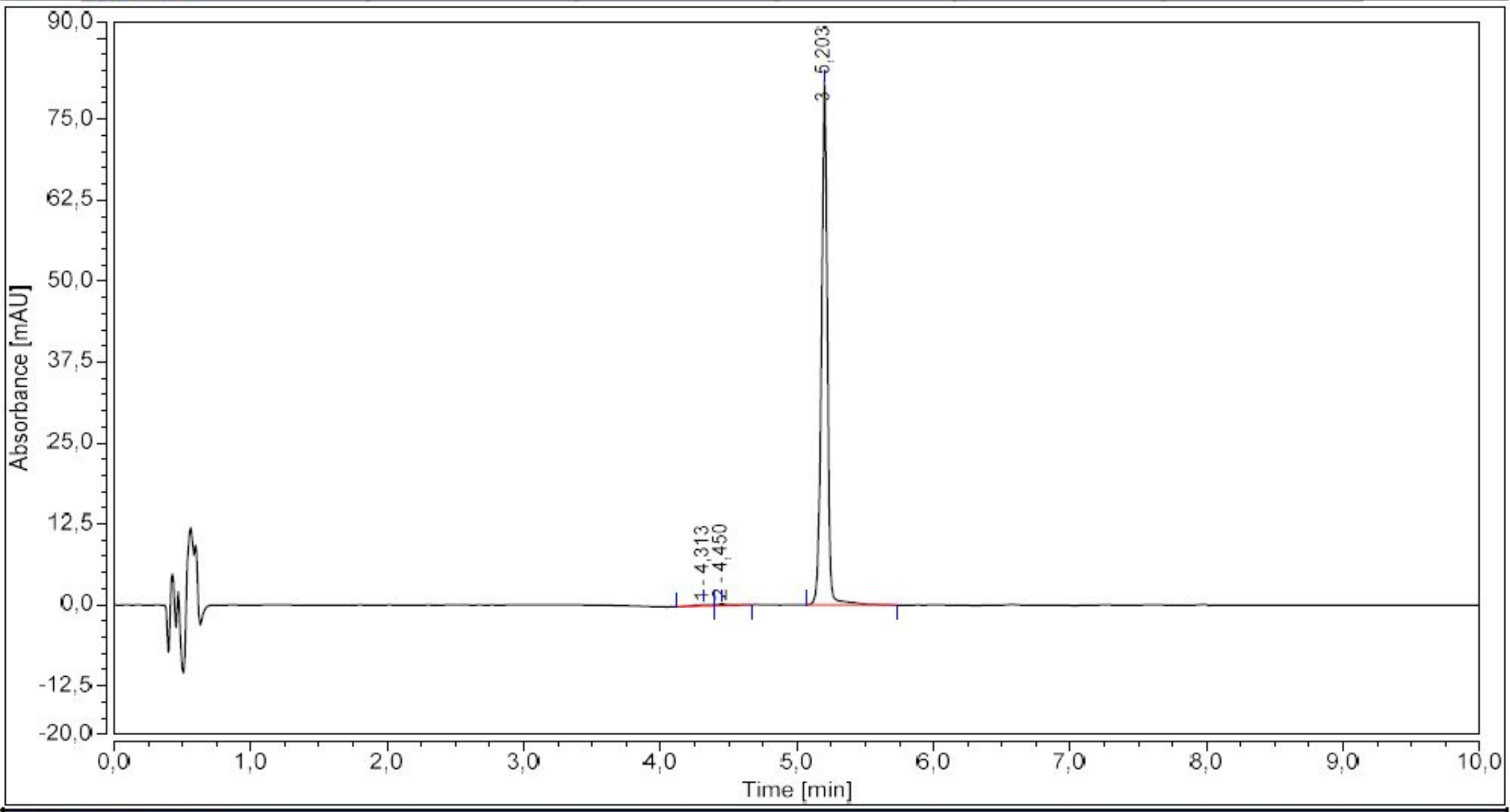

Integration Results

No.	Peak Name	Retention Time min	Area mAU*min	Height mAU	Relative Area %	Relative Height %	Amount n.a.
1		4,313	0,031	0,197	0,76	0,24	n.a.
2		4,450	0,029	0,310	0,69	0,38	n.a.
3		5,203	4,087	80,404	98,56	99,37	n.a.
**Total:**			**4,147**	**80,912**	**100,00**	**100,00**	

6-Hydrazineyl-3-(naphthalen-2-yl)-4-(pyridin-4-yl)pyridazine 17

Chromatogram

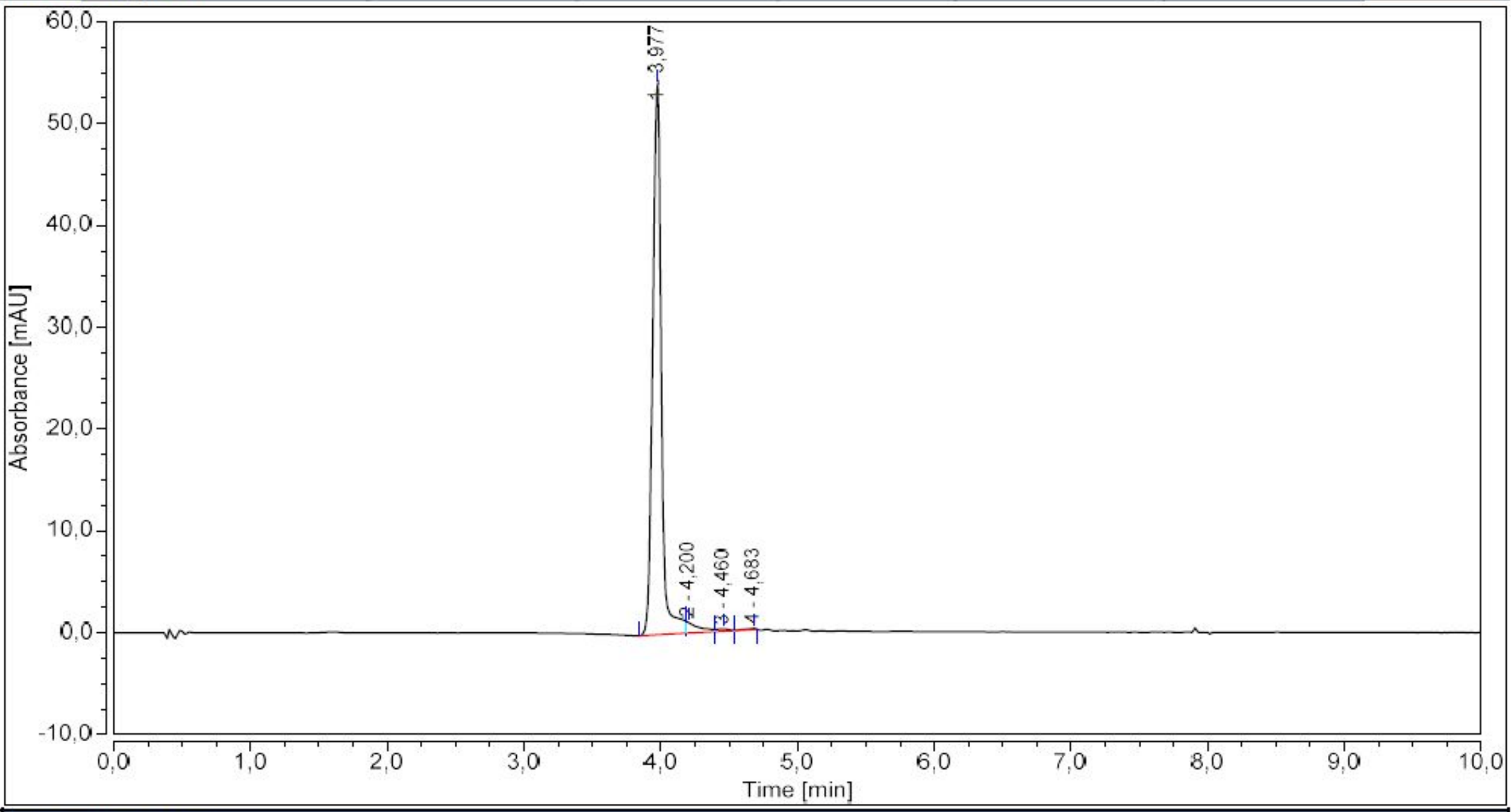

Integration Results

No.	Peak Name	Retention Time min	Area mAU*min	Height mAU	Relative Area %	Relative Height %	Amount n.a.
1		3,977	3,968	54,164	96,33	97,43	n.a.
2		4,200	0,113	1,077	2,74	1,94	n.a.
3		4,460	0,026	0,231	0,63	0,42	n.a.
4		4,683	0,012	0,119	0,30	0,21	n.a.
**Total:**			**4,119**	**55,590**	**100,00**	**100,00**	

6-(2-Methylhydrazineyl)-3-(naphthalen-2-yl)-4-(pyridin-4-yl)pyridazine 18

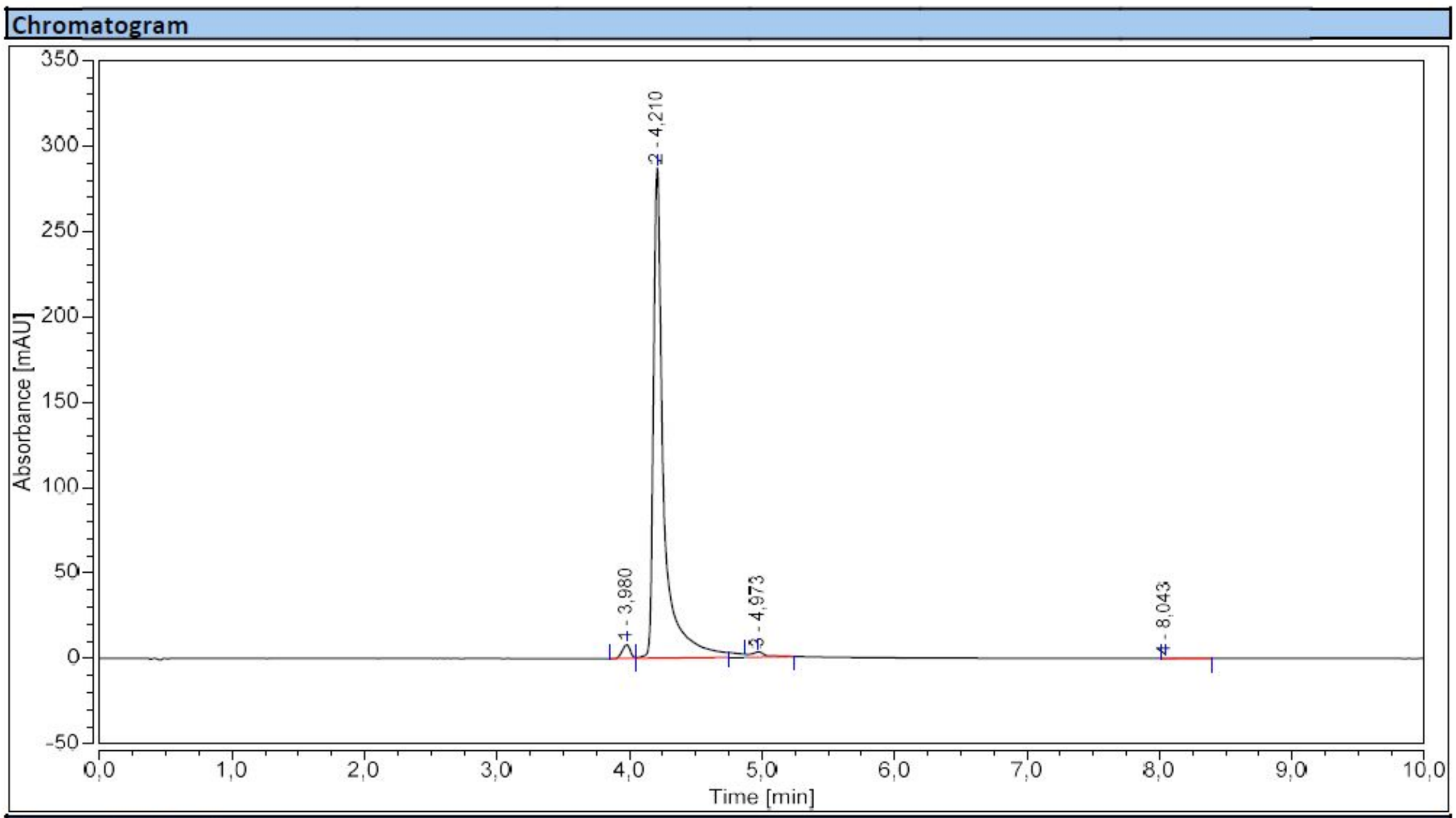

Integration Results

No.	Peak Name	Retention Time min	Area mAU*min	Height mAU	Relative Area %	Relative Height %	Amount n.a.
1		3,980	0,573	8,242	2,13	2,76	n.a.
2		4,210	25,843	287,002	96,23	96,17	n.a.
3		4,973	0,406	3,011	1,51	1,01	n.a.
4		8,043	0,032	0,167	0,12	0,06	n.a.
**Total:**			**26,855**	**298,423**	**100,00**	**100,00**	

3-(Naphthalen-2-yl)-4-(pyridin-4-yl)-6-vinylpyridazine 19

Chromatogram

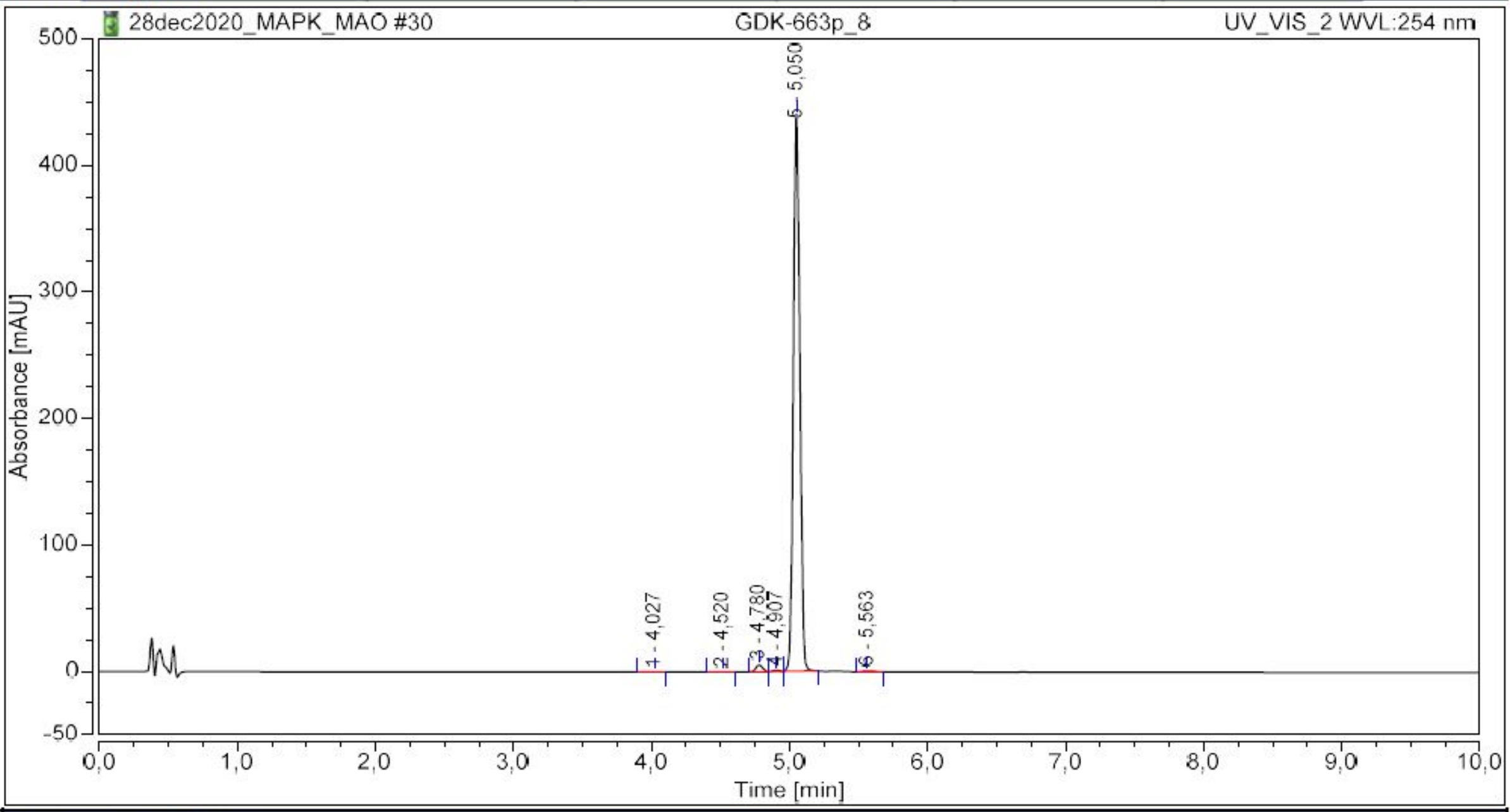

Integration Results

No.	Peak Name	Retention Time min	Area mAU*min	Height mAU	Relative Area %	Relative Height %	Amount n.a.
1		4,027	0,015	0,152	0,06	0,03	n.a.
2		4,520	0,015	0,155	0,06	0,03	n.a.
3		4,780	0,300	5,713	1,24	1,28	n.a.
4		4,907	0,057	1,027	0,24	0,23	n.a.
5		5,050	23,771	440,326	98,13	98,26	n.a.
6		5,563	0,067	0,736	0,28	0,16	n.a.
**Total:**			**24,224**	**448,109**	**100,00**	**100,00**	

***N*,*N*-Dimethyl-2-(6-(naphthalen-2-yl)-5-(pyridin-4-yl)pyridazin-3-yl)ethan-1-amine 20**

Chromatogram

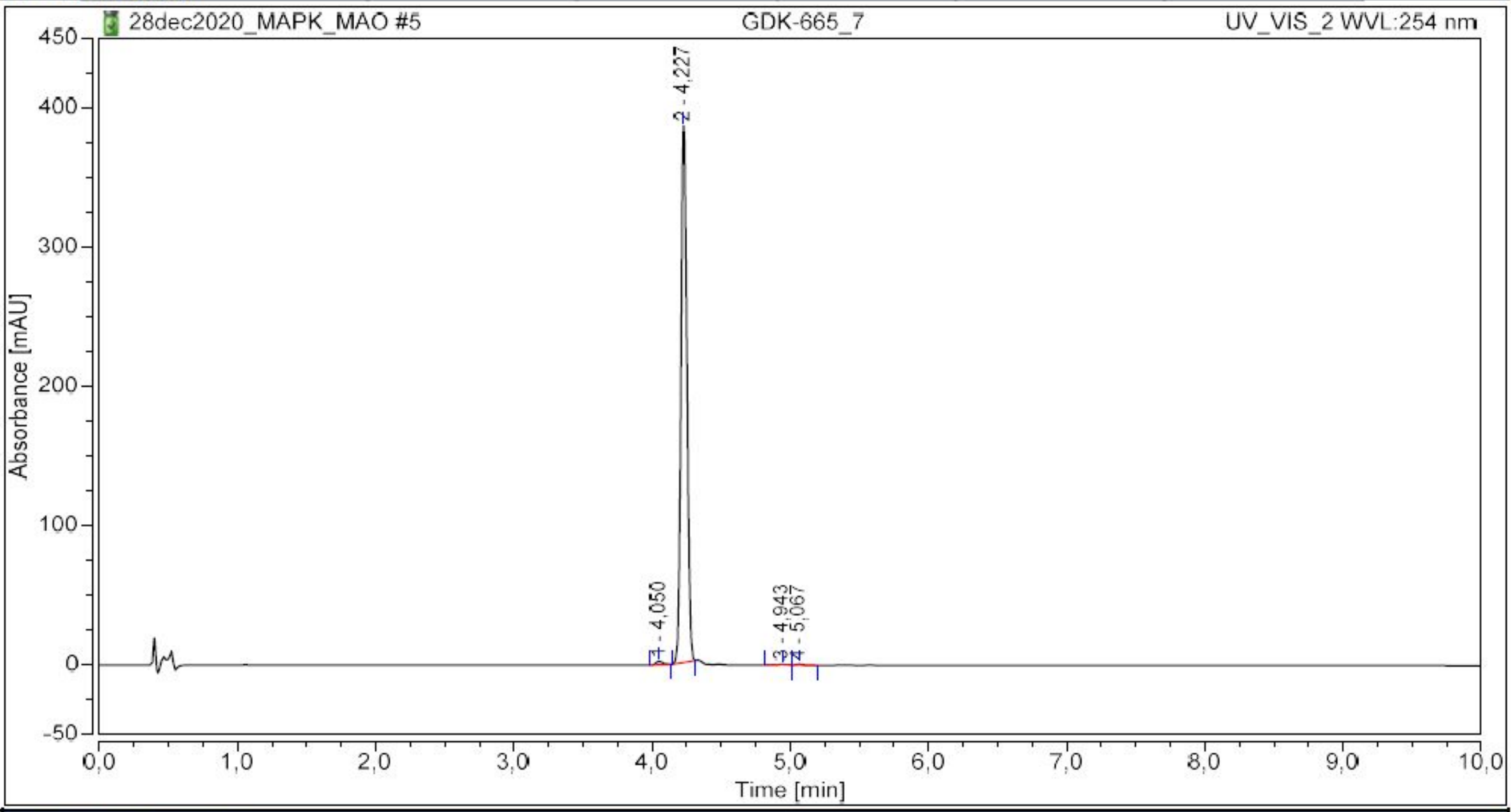

Integration Results

No.	Peak Name	Retention Time min	Area mAU*min	Height mAU	Relative Area %	Relative Height %	Amount n.a.
1		4,050	0,146	2,587	0,74	0,66	n.a.
2		4,227	19,409	385,594	98,79	98,98	n.a.
3		4,943	0,039	0,538	0,20	0,14	n.a.
4		5,067	0,053	0,834	0,27	0,21	n.a.
**Total:**			**19,648**	**389,552**	**100,00**	**100,00**	

***N*[1],*N*[1]-Dimethyl-*N*[2]-(6-(naphthalen-1-yl)-5-(pyridin-4-yl)pyridazin-3-yl)ethane-1,2-diamine 21**

Chromatogram

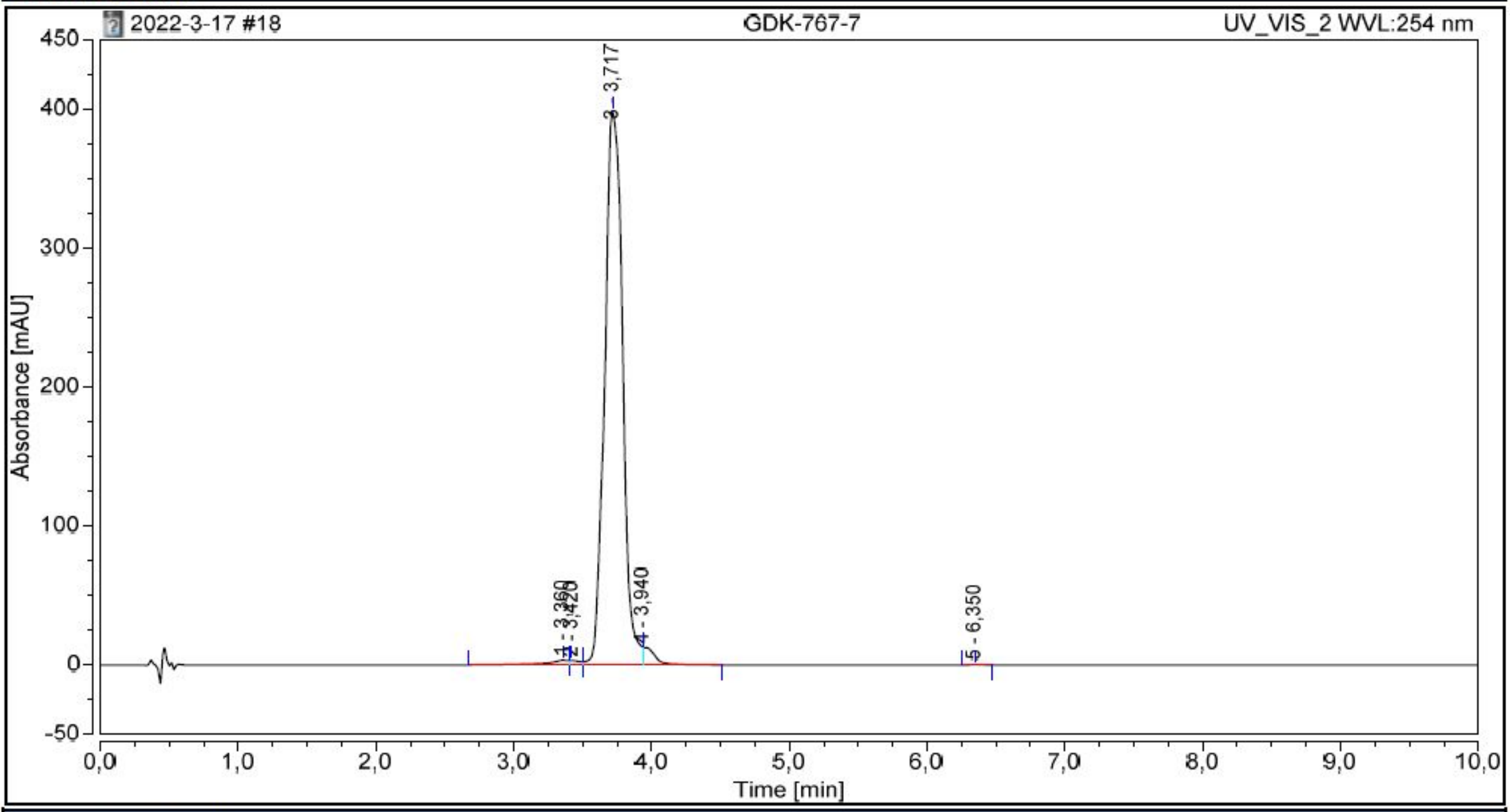

Integration Results

No.	Peak Name	Retention Time min	Area mAU*min	Height mAU	Relative Area %	Relative Height %	Amount n.a.
1		3,360	0,646	3,380	1,04	0,81	n.a.
2		3,420	0,282	3,198	0,45	0,76	n.a.
3		3,717	60,061	398,499	96,43	95,31	n.a.
4		3,940	1,273	12,741	2,04	3,05	n.a.
5		6,350	0,024	0,297	0,04	0,07	n.a.
**Total:**			**62,286**	**418,115**	**100,00**	**100,00**	

***N*,*N*-Dimethyl-6-(naphthalen-1-yl)-5-(pyridin-4-yl)pyridazin-3-amine 22**

Chromatogram

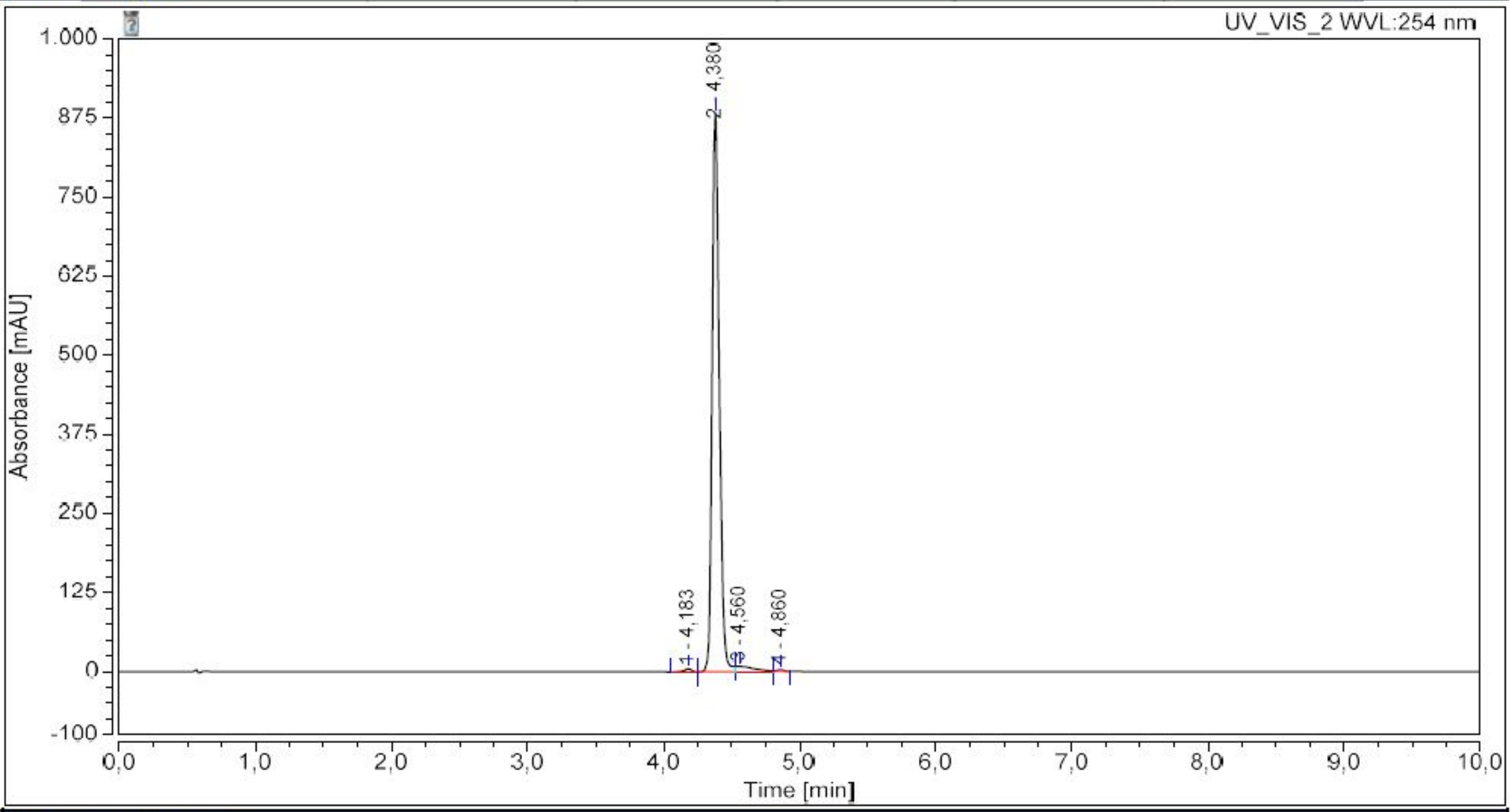

Integration Results

No.	Peak Name	Retention Time min	Area mAU*min	Height mAU	Relative Area %	Relative Height %	Amount n.a.
1		4,183	0,287	4,945	0,52	0,55	n.a.
2		4,380	53,779	883,793	96,79	98,19	n.a.
3		4,560	1,343	8,383	2,42	0,93	n.a.
4		4,860	0,153	2,917	0,28	0,32	n.a.
**Total:**			**55,561**	**900,039**	**100,00**	**100,00**	
